# Chiral Ligands Based on Binaphthyl Scaffolds for Pd-Catalyzed Enantioselective C–H Activation/Cycloaddition Reactions

**DOI:** 10.1021/jacs.2c09479

**Published:** 2022-11-15

**Authors:** José Manuel González, Xandro Vidal, Manuel Angel Ortuño, José Luis Mascareñas, Moisés Gulías

**Affiliations:** Centro Singular de Investigación en Química Biolóxica e Materiais Moleculares (CIQUS) and Departamento de Química Orgánica, Universidade de Santiago de Compostela, 15782 Santiago de Compostela, Spain

## Abstract

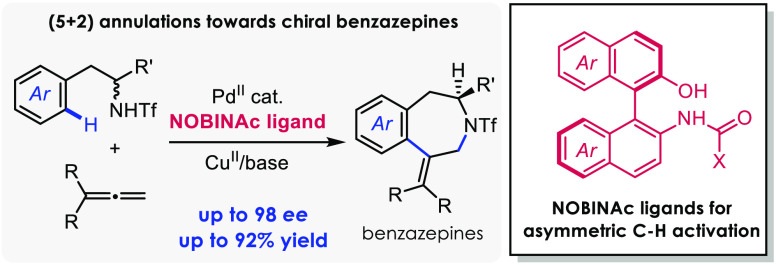

We report the first examples of the use of a new class of ligands (NOBINAc) for performing asymmetric C–H activations using palladium catalysts. These ligands combine the axial chirality of binaphthyl scaffolds with the bifunctional and bidentate coordination properties of mono-N-protected amino acids (MPAAs), which are well-known to favor Pd-promoted C–H activations via concerted metalation–deprotonation mechanisms. We demonstrate that our new ligands enable substantially higher enantioselectivities than MPAAs in the assembly of 2-benzazepines through formal (5 + 2) cycloadditions between homobenzyltriflamides or *o*-methylbenzyltriflamides and allenes.

Transition-metal-catalyzed C–H functionalization reactions have emerged as one of the more powerful tools in the field of organic synthesis.^1^ A major ongoing challenge in the area is the development of enantioselective versions, especially for reactions in which the asymmetry is created in the C–H activation step.^2^ Despite significant progress, the number of such asymmetric reactions is still small, and in many cases the resulting enantioselectivities are far from optimal.^3^ A major breakthrough in the field was the discovery by Yu and co-workers of mono-N-protected amino acids (MPAAs) as efficient chiral ligands to promote a broad variety of Pd-catalyzed enantioselective C–H functionalizations.^4^ Mechanistic studies have established that these ligands bind the metal in a bidentate manner, with the *N*-acyl moiety acting as an internal base to drive the C–H activation step (concerted metalation–deprotonation (CMD) mechanism). The metal chelation leads to a relatively rigid transition state, which allows efficient transfer of asymmetric information from the chiral center of the amino acid to the resulting palladacycle intermediate.^5^

Relying on these chiral ligands, we have recently reported a Pd-catalyzed desymmetrizing cycloaddition between α-diarylmethyltriflamides and allenes to give valuable tetrahydroisoquinolines via enantioselective C–H activation/cycloaddition processes.^6^ The best conditions to perform this transformation involved the use of 2,6-difluorobenzoylleucine as the ligand, which allowed enantioselectivities of up to 95% *ee*.

Unfortunately, homologous α-dibenzylmethyltriflamides, which have an extra methylene carbon between the stereogenic center and the aromatic ring and therefore provide appealing benzazepines in their annulation to allenes, led to very poor enantioselectivities (barely 14% *ee*).^7^ After an intense screening of other MPAAs and reaction conditions, the best results were obtained with Boc-valine, which in the best of the cases gave a yet modest 76% *ee*. This poor asymmetric efficiency might be related to the formation of relatively flexible six-membered palladacycles in the C-H activation step (Scheme 1).

**Scheme 1:**
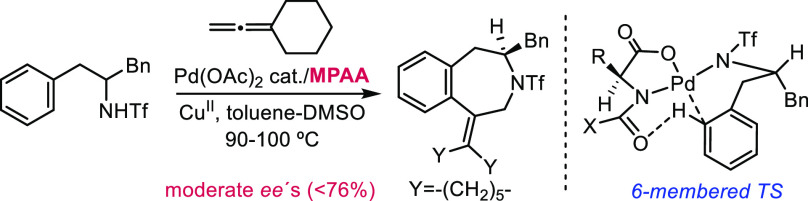
Preliminary Studies on the Synthesis of Benzazepines through a Formal (5 + 2) Annulation

With this state of the art, we reasoned that ligands featuring axial instead of point chirality might allow for more efficient transmission of chiral information. These ligands with atropoisomeric chirality are well-established in the field of asymmetric catalysis, but curiously, they have essentially never been used as bidentate anionic ligands in palladium-mediated C–H functionalization processes.^8^

Herein, we demonstrate that acylated versions of NOBIN (NOBINAc) are excellent ligands for asymmetric Pd-catalyzed C–H activation/annulation processes, clearly outperforming standard MPAAs. Specifically, we report their use to achieve highly enantioselective (5 + 2) annulations between homobenzyltriflamides or *o*-methylbenzyltriflamides and allenes. These reactions allow the construction of a variety of enantioenriched 2-benzazepines using either desymmetrization or kinetic resolution strategies and in reactions that involve activation of either sp^2^ or sp^3^ C–H bonds.^9^

Atropoisomeric bidentate ligands such as BINAP have been widely used as privileged scaffolds in many metal-catalyzed asymmetric reactions.^10^ The restricted rotation around the aryl–aryl bond generates a rigid asymmetric environment that can be efficiently sensed by substrates when coordinated to the metal center.

Inspired by these structures and considering the dianionic nature of MPAA ligands and the key role of the amide moiety as an internal base for the C–H activation (CMD mechanism),^4b,5^ we reasoned that acylated NOBIN derivatives such as **L** (Figure 1a) might be effective ligands in Pd-catalyzed asymmetric C–H activations. Preliminary DFT calculations confirmed that this class of ligands can provide unstrained palladacycles like those obtained using MPAAs, with similar bond distances between the metal and the O and N atoms. The metal geometry is also rather similar (square-planar), although with a higher bite angle (Figure 1b,c). More importantly, the chiral environment resulting from the complexation of NOBINAc is different, which may have consequences in the asymmetric transfer process.

**Figure 1:**
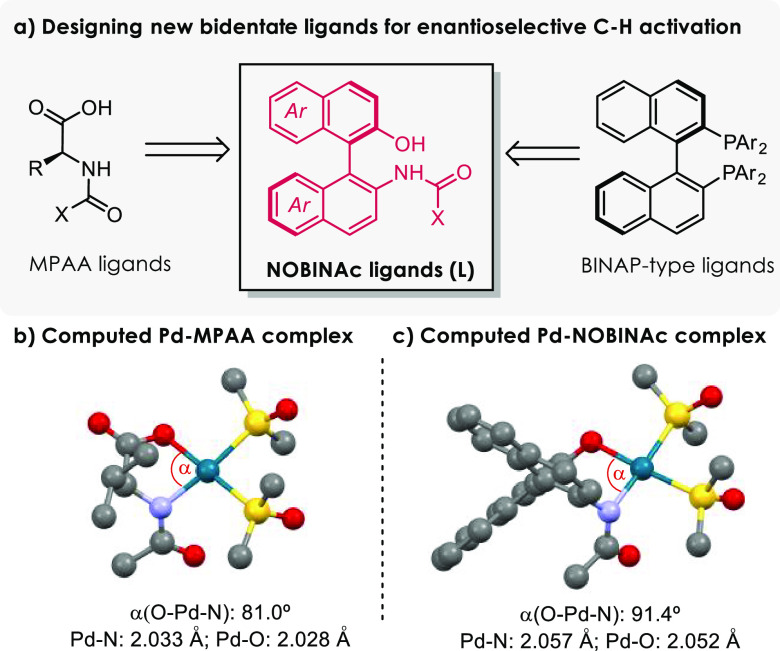
(a) Design of NOBINAc ligands for asymmetric Pd-catalyzed activation. (b, c) DFT-optimized structures for qualitative comparison between [LPd(DMSO)_2_] complexes (L = MPAA and NOBINAc).

As indicated above, our study on asymmetric annulations to form benzazepines started with the use of 2,6-difluorobenzoylleucine as the ligand. The reaction between triflyl-protected homobenzylamine **1a** and commercially available vinylidenecyclohexane (**2a**) using conditions similar to those described for benzylamides^6^ gave a good yield (72%), but the product was isolated with only 14% *ee*. After intensive screening with different MPAAs, we found that the best conditions involved the use of Boc-valine, which produced the cycloadduct in 85% yield but with a still modest 76% *ee* (Scheme 1; see the Supporting Information for the complete screening). Other ligands sporadically used in asymmetric C–H activation with palladium, such APAO, APAQ or *p*-GluOH, gave lower yields and *ee*’s (Scheme 2).^11^

**Scheme 2:**
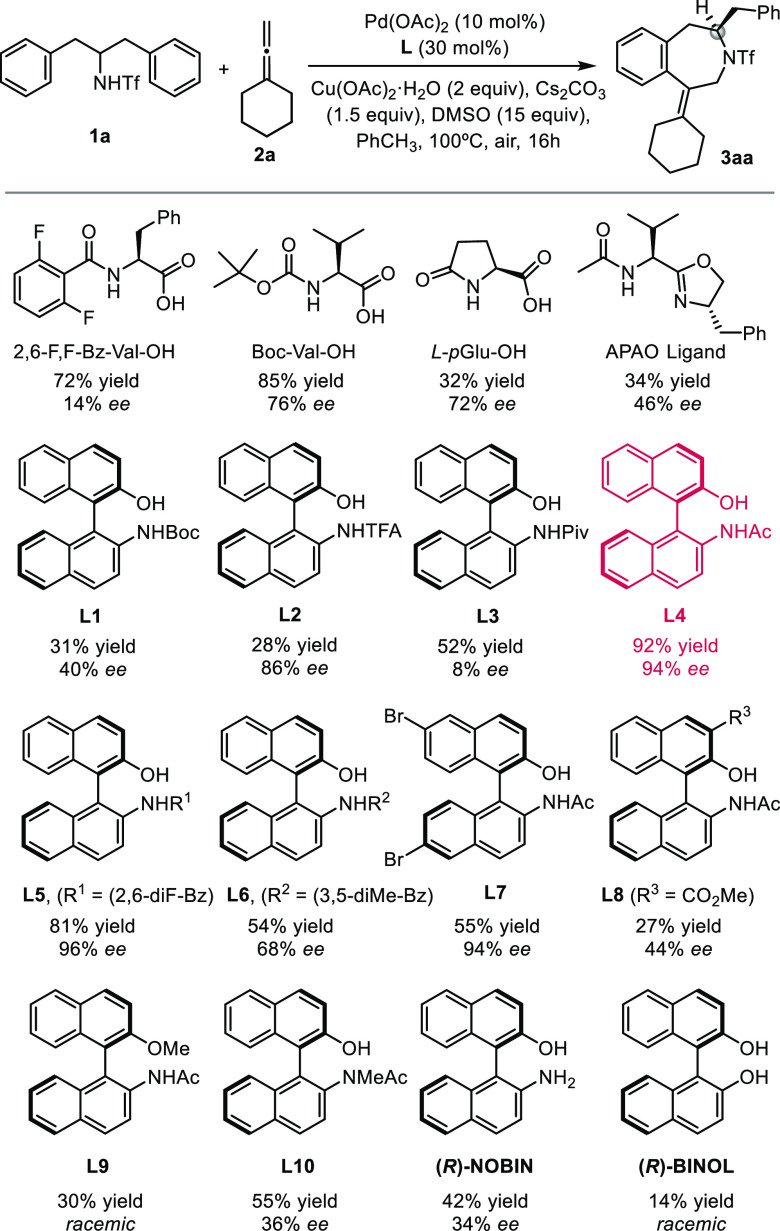
Screening of Ligands Reaction conditions: **1a** (0.1 mmol), **2a** (0.2 mmol), Pd(OAc)_2_ (10 mol %), Ligand **L** (30 mol %), Cu(OAc)_2_·H_2_O (2 equiv), Cs_2_CO_3_ (1.5 equiv), DMSO (15 equiv), PhCH_3_ (1.0 mL), air, 100 °C, 16 h. Isolated yields are reported.

Remarkably, the *N*-Boc-protected (*R*)-NOBIN derivative **L1** was a valid ligand for the reaction, but the product was obtained in a low 31% yield with 40% *ee*. While this initial enantioselectivity was poor, the experiment validated the use of this type of ligand with the binaphthyl scaffold and a phenol handle instead of the carboxylic acid of MPAAs. Using the trifluoromethylacetyl NOBIN derivative increased the enantioselectivty to a promising 86% *ee*, although the product was obtained in only 28% yield. Gratifyingly, with the *N*-acetyl derivative **L4** [(***R***)-**Ac-NOBIN**] the reaction took place in an excellent 92% yield with 94% *ee*. We could even increase the enantiomeric excess to 96% *ee* using 2,6-benzoyl analogue **L5**, but at the cost of a slight decrease in the reaction yield to 81%, while 3,5-benzoyl analogue **L6** was clearly less effective. Other *N*-acetyl NOBIN derivatives with substituents on the naphthyl skeleton, such **L7** and **L8**, gave worse results.

Importantly, a control experiment using the methyl ether derivative **L9** led to lower conversion and a racemic product, a result similar to that obtained when the reaction was carried out in the absence of ligands (40% yield after 16 h), while ligand **L10** with the methylated amino group led to a 55% yield with 36% *ee*. When the free amine NOBIN was used as the ligand, the reaction was also low-yielding (34%), although it could induce some enantioselectivity (42% *ee*). Not surprisingly, when binaphthol was used instead of NOBINAc, the reaction was very inefficient (14% yield) and furnished the product in racemic form. All of these results support the requirement of a dianionic palladium ligand with an acetamide group capable of promoting the CMD process. The cation of the base also plays a role in the reaction, since the yields and enantioselectivities decreased when K_2_CO_3_ (82% yield, 86% *ee*) or especially Li_2_CO_3_ (38% yield, 6% *ee*) was used instead of the cesium salt. Interestingly, a comparison of reaction rates between reactions with and without ligand showed that the reaction with ligand is about 2 times faster (see the Supporting Information).

With the optimal conditions in hand, we tested the scope of the annulation. Gratifyingly, benzazepine products **3ba**–**3ea** containing halogens at the *ortho*, *meta*, and *para* positions were assembled in excellent yields (76–92%) with enantioselectivities of up to 97% *ee* (Scheme 3). Other types of substituents are also tolerated, as illustrated for the trifluoromethyl (**3fa**, 98% *ee*), methoxy (**3ga**, 94% *ee*)*,* and methyl (**3ha**, 92% *ee*) derivatives. A homonaphthylamide precursor was also tested and gave the expected product **3ia** in 92% yield with 84% *ee*.

**Scheme 3:**
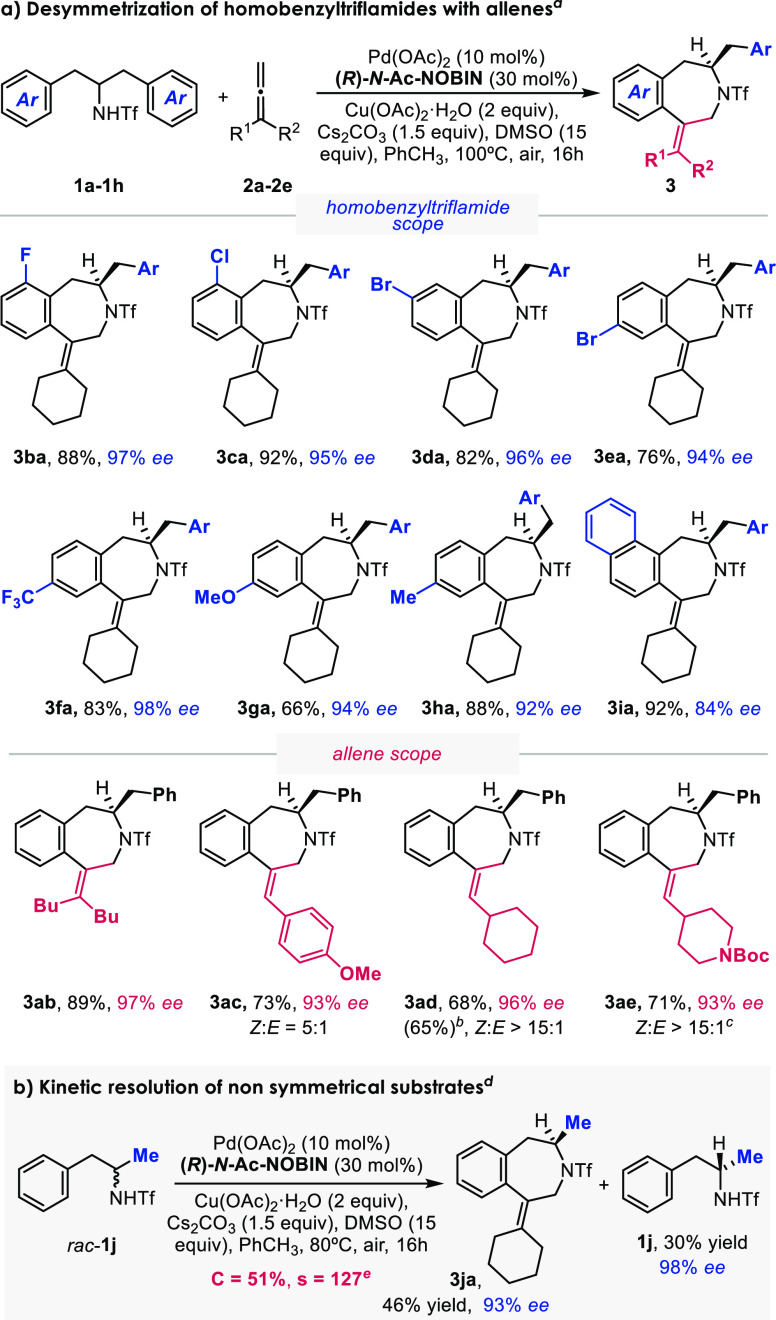
Scope of the Asymmetric (5 + 2) Annulation Conditions: **1** (0.1 mmol), **2** (0.2 mmol), Pd(OAc)_2_ (10 mol %), ligand (30 mol %), Cu(OAc)_2_·H_2_O (2 equiv), Cs_2_CO_3_ (1.5 equiv), DMSO (15 equiv), PhCH_3_ (1 mL), air, 100 °C, 16 h. The reaction was run at a 0.5 mmol scale of **1a**. The reaction time was 48 h. The reaction was run at 80 °C with 0.5 mL of PhCH_3_. The conversion (*C*) and selectivity (*s*) were calculated as *C* = *ee*^SM^/(*ee*^SM^ + *ee*^PR^) and *s* = ln[(1 – *C*)(1 – *ee*^SM^)]/ln[(1 – *C*)(1 + *ee*^SM^)], respectively, where *ee*^SM^ is the *ee* of recovered starting material **1** and *ee*^PR^ is the *ee* of product **3**.

The reaction is also quite general with respect to the allene component. A nonadiene, as an example of other 1,1-disubstituted allenes, gave the expected benzazepine adduct **3ab** in 89% yield with 97% *ee*. Monosubstituted allenes also provided good results, as exemplified for the synthesis of products **3ac**, **3ad**, and **3ae**, which were obtained with high enantioselectivities (93–96% *ee*), good *E:Z* diastereoselectivity, and complete regioselectivity. Compound **3ad** was crystallized, which allowed us to assign the absolute configuration of the product as *R*.

Importantly, the annulation can be extended to nonsymmetric precursors, providing very efficient kinetic resolutions. When we tested the reaction with α-methylphenethylamide **1j** and allene **2a**, the corresponding benzazepine **3ja** was isolated in 46% yield with 93% *ee*, and the chiral homobenzylamides were recovered in 30% yield with 98% *ee*, which translates to a selectivity factor of 127.

The benzazepine cycloadducts can be easily manipulated thanks to the presence of the exocyclic double bond. For instance, product **3ad** can be easily hydrogenated to give the saturated product **4** with complete *trans* diastereoselectivity in 80% yield (Scheme 4). This product can be deprotected using Red-Al without deterioration of the enantioselectivity. Product **3ad** can also be selectively oxidized in one step to the corresponding ketone using ruthenium trichloride catalyst and periodate, again without affecting the *ee*.

**Scheme 4:**
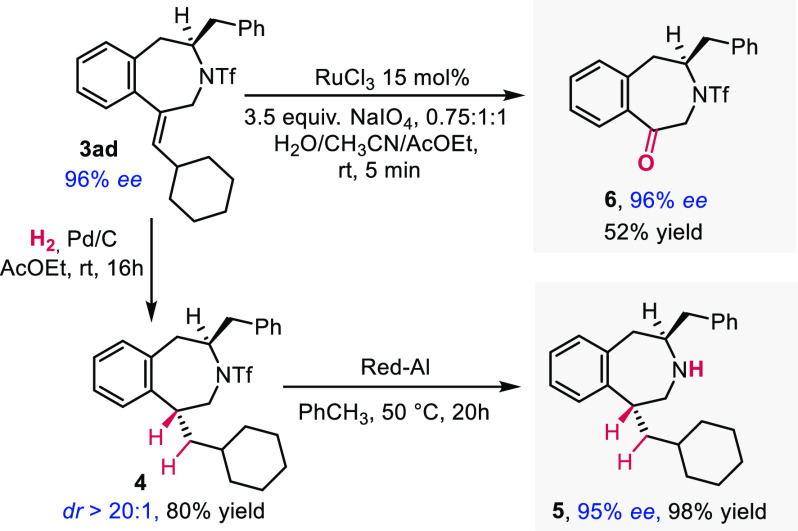
Derivatization of the Benzazepine Products

We recently reported that benzazepines can also be assembled by annulation of *o*-methylbenzylamides with allenes in a reaction that involves the activation of C(sp^3^)–H bonds.^12^ Unfortunately, the asymmetric version using MPAA-type ligands led to low enantioselectivities (less than 79% *ee* in the best of the cases, with a selectivity factor of 13). Remarkably, under the standard conditions with NOBINAc ligand **L4**, the enantioselectivities rose to 95% *ee* and 90% *ee* for the product **8aa** and the starting material **7a** (Scheme 5), and the selectivity factor increased to 121. The reactions are also effective for other substrates, and again, the obtained products exhibited excellent enantioselectivities (Scheme 5, bottom). Overall, the above results confirm the NOBINAc structures as excellent ligands for the above asymmetric annulations involving a C–H activation and Pd(II)/Pd(0) catalytic cycles.

**Scheme 5:**
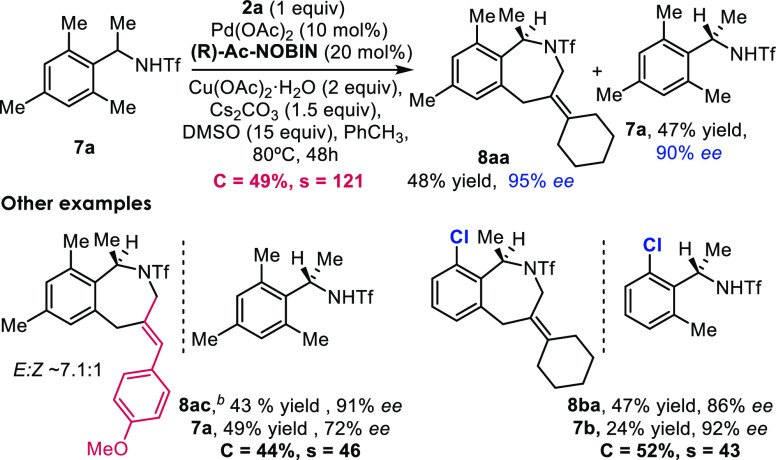
Kinetic Resolution of *o*-Methylbenzyltriflamides Reaction conditions: *rac*-**1a** (0.1 mmol), **2a** (0.1 mmol), Pd(OAc)_2_ (10 mol %), **Ac-NOBIN** (30 mol %), Cu(OAc)_2_·H_2_O (2 equiv), Cs_2_CO_3_ (1.5 equiv), DMSO (15 equiv), PhCH_3_ (1 mL), air, 80 °C, 48 h. 1.5 equiv of **2c**.

Why is the NOBINAc scaffold so effective in the asymmetric induction? To shed light on this question, we computed the relative Gibbs energies of the C–H bond activation transition states using DFT, with ligand **L4** and homobenzylamide **1a**. We used the M06 density functional^13^ as implemented in Gaussian 16.^14^ We explored several conformations, but only the most stable ones are reported and discussed herein.^15,16^

We considered two main topologies for the six-membered transition state structures,^5a^ depending on whether the coplanar *ortho* C–H bond (to be activated) points downward (**D**) or upward (**U**) with respect to the Pd coordination plane (see the Supporting Information). Remarkably, the rigid framework of NOBINAc favors structures **D**, as the alternative forms **U** exhibit strong distortions of the Pd square-planar geometry (N–O–N–C dihedral angle for **TS-US** = 24.9°; Figure 2a). This is in clear contrast to the results obtained using Ac-Val-OH, where lower distortions are found in both types of transition state topologies **D** and **U** (dihedral angles = 0–13°; see the Supporting Information). Indeed, with the amino acid ligand the lowest-energy transition states for each isomer are **TS-DR** and **TS-US**, and the Gibbs energy difference is 4.8 kcal/mol. However, in the case of NOBINAc, the most stable transition states leading to the *R* and *S* enantiomers are **TS-DR** and **TS-DS**, respectively (Figure 2b), with **TS-DR** clearly preferred by 8.2 kcal/mol. While this number suggests that complete enantioselection should be obtained, it is very likely that there could be some ligand-free reaction, and especially some background reaction in which NOBINAc acts as monodentate ligand.^5b^ These processes might contribute to partial erosion of the enantioselectivity. Furthermore, it is important to note that these calculations do not simulate the full experimental scenario, and therefore, the energetic values should be taken carefully, although they are very useful for comparative purposes.

**Figure 2:**
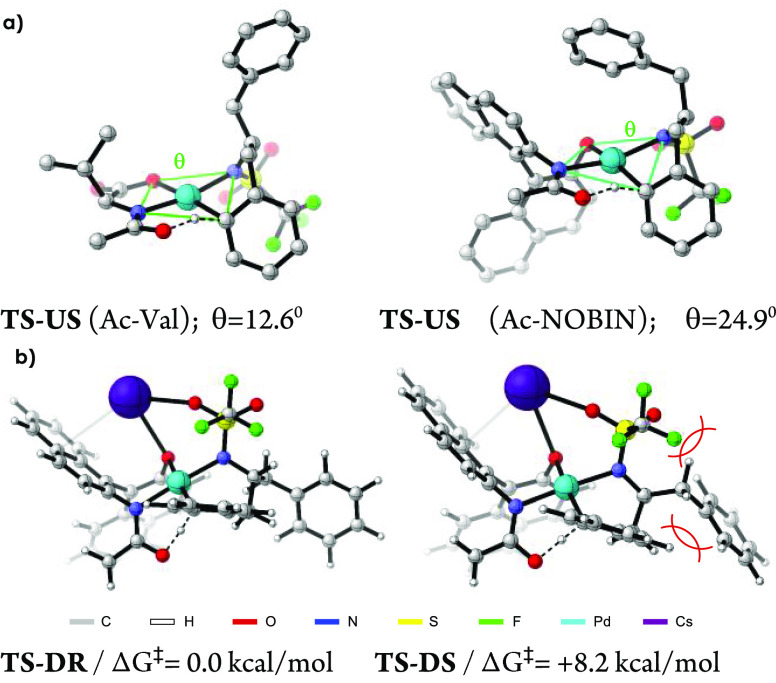
(a) DFT-optimized structures and dihedral angles of **TS**-**US** for acetyl-Val and acetyl-NOBIN. Hydrogens and Cs atoms have been omitted for clarity. (b) DFT-optimized structures and relative Gibbs energies of the two more stable conformations of transition states for the key C–H bond activation using NOBINAc ligands.

Importantly, the calculated TSs allow us to infer the reasons behind the differences in energy between **TS-DR** and **TS-DS**, namely, clear steric clashes of the nonreacting Bn substituent with the other benzyl group and with the triflyl group (Figure 2b).

In conclusion, we have discovered a new class of ligands (NOBINAc) for performing palladium-catalyzed enantioselective annulations involving C–H activations. The use of these ligands allows the assembly of a variety of enantioenriched benzazepine products by reaction of very simple starting benzylamide precursors with allenes. The much-better asymmetric induction obtained with this class of ligands over that with classic amino acids originates from the destabilization of **U** transition state structures due to the introduction of further strain in the Pd square-planar geometries. Our examples represent the first application of metal-catalyzed C–H activation chemistry in the enantioselective construction of seven-membered rings through (5 + 2) annulations. Further developments with this class of activating ligands are currently underway.

## Supporting Information Available

Experimental details, characterization data for all new compounds, and computational details (PDF)
