# Supplementary material for: Chiral Ligands Based on Binaphthyl Scaffolds for Pd-Catalyzed Enantioselective C–H Activation/Cycloaddition Reactions
Source: J Am Chem Soc. 2022 Nov 15;144(47):21437–42. doi: 10.1021/jacs.2c09479 (PMC9716525; doi:10.1021/jacs.2c09479)

Supporting Information

Chiral Ligands Based on Binaphthyl Scaffolds for Pd-Catalyzed Enantioselective C–H Activation/Cycloaddition Reactions

José Manuel González, Xandro Vidal, Manuel Ángel Ortuño, José Luis Mascareñas*, Moisés Gulías*

e-mail: joseluis.mascarenas@usc.es moises.gulias@usc.es

Centro Singular de Investigación en Química Biológica y Materiales Moleculares (CIQUS) and Departamento de Química Orgánica, Universidad de Santiago de Compostela, 15782 Santiago de Compostela, Spain.

Table of Contents

1. General experimental information 3

2. Synthesis of chiral ligands 4

2.1. Synthesis of NOBIN type chiral ligands 4

3. Synthesis of starting materials and allenes 11

3.1. Synthesis of symmetrical homobenzyltriflamides 11

3.2. Synthesis of non-symmetrical homobenzyltriflamides 14

3.3. Synthesis of α-substituted *ortho*-methyl benzyltriflamides 15

3.4. Synthesis of allenes 16

4. Desymmetrization and kinetic resolution of homobenzyltriflamides through an enantioselective Pd-catalyzed (5+2) annulation with allenes 17

4.1. Chiral ligand screening 17

4.2. Chiral ligand loading 19

4.3. Comparison of the reaction with and without ligand 20

4.4. Base Screening 21

4.5. Desymmetrization of homobenzyltriflamides; reaction scope 22

4.6. Kinetic resolution of homobenzyltriflamides 41

5. Synthetic manipulations of the enantioenriched cycloadducts 44

5.1. Hydrogenation of the exocyclic double bond and deprotection of the *N*-triflyl group 44

5.2. Oxidative cleavage of the exocyclic double bond 47

6. Kinetic resolution of *ortho*-methyl benzyltriflamides 49

7. Computational data 59

7.1. Computational details 59

7.2. Additional schemes and structural information 59

8. References 63

9. NMR Spectra 65

1. General experimental information

Reactions were conducted in dry solvents under Argon unless otherwise stated. Dry solvents were obtained from Across Organics, Extra Dry over Molecular Sieves, and used without further purification. $Pd(OAc)_2$ (98%) [3375-31-1] was obtained from Strem. All other chemicals were purchased from Sigma-Aldrich, Acros Organics, Alfa Aesar, Fluorochem, TCI Chemicals or Abcr and they were used as received. All palladium-catalyzed reactions were carried without precautions to elude moisture or oxygen.

The abbreviation "rt" refers to reactions carried out at a temperature between 21-25°C. Reaction mixtures were stirred using Teflon-coated magnetic stir bars. Thin layer chromatography (TLC) was carried out on pre-coated silica gel F_{254} plates with visualization under UV light or by dipping the plate into solutions of *p*-anisaldehyde, ninhydrin, phosphomolybdic acid or potassium permanganate solutions followed by heating. Column chromatography was performed on silica gel (40-60 µm) unless otherwise stated.

NMR data was collected on Varian Mercury 300 MHz or Bruker AVIII 500 MHz spectrometers. Chemical shifts are given in ppm (δ) and are referenced to the residual $CDCl_3$ solvent peak at 7.26 ppm (^{1}H-NMR) and 77.16 ppm (^{13}C-NMR). Conventional one-dimensional (1D) ^{1}H-NMR, ^{19}F-NMR, ^{13}C{^{1}H}-NMR, Distortionless Enhancement by Polarization Transfer Spectra (DEPT) and two-dimensional (2D) ^{1}H-^{1}H Correlation Spectroscopy (COSY), ^{1}H-^{1}H Nuclear Overhauser Effect Spectroscopy (NOESY), ^{1}H-^{13}C heteronuclear single quantum coherence (HSQC),^{1}H-^{13}C Heteronuclear Multiple-Bond Correlation Spectroscopy (HMBC) experiments were recorded at room temperature under routine conditions. NMR data was analysed using MestReNova NMR data processing software (http://mestrelab.com/). High Resolution Mass Spectra (HRMS) were performed at the CACTUS facility of the University of Santiago de Compostela on a Bruker micrOTOF spectrometer.

Enantiomeric ratios (*er*) were determined on an Agilent HPLC 1100 Series or on a Jasco SFC 4000 series using commercially available chiral columns. All racemic products were prepared under the same procedure than the chiral products although with the employment of a racemic mono-protected amino acid as ligand.

X-ray crystallographic analysis of compounds **3ad**, **4** and **8aa** was performed at the CACTUS facility of the University of Santiago de Compostela and the absolute stereochemistry of all compounds was assigned by analogy to **3ad** and **8aa**.

2. Synthesis of chiral ligands

All mono-protected amino acids (MPAA), acetyl-protected aminomethyl oxazoline (APAO) and acetyl-protected aminoethyl quinoline (APAQ) ligands were purchased from commercial sources or synthesized according to literature procedures.

2.1. Synthesis of NOBIN type chiral ligands

Procedure for the synthesis of NOBIN derivatives via Smiles reaction followed by NH_2 protection

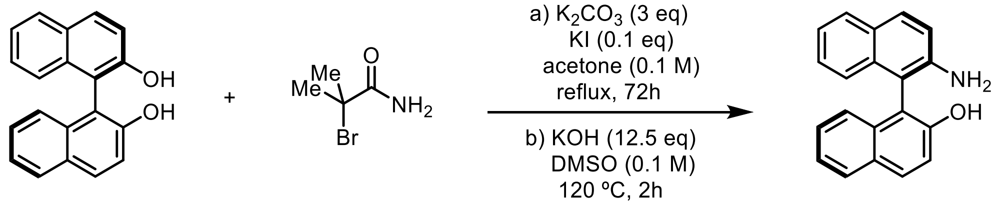

(*R*)-BINOL **(*R*)-NOBIN**

Following a previously reported procedure,[1] (*R*)-BINOL (1.00 g, 3.49 mmol), 2-bromo-2-methylpropanamide (1.74 g, 3 eq), K_2CO_3 (1.45 g, 3 eq) and KI (58 mg, 0.1 eq) were added to a flame-dried round botton flask under argon atmosphere. Then, dry acetone (35 mL) was injected to the flask via syringe. The orange solution was heated at reflux for 72 hours. After that, the solution was filtered through Celite© and eluted with ethyl acetate. The solvent was removed under reduced pressure, and the resulting crude was redissolved in DMSO (35 mL). Then, KOH (12.5 eq) was added to the solution. The resulting black solution was heated at 120°C for two hours. After that, the reaction was allowed to warm at rt, and water (175 mL) was added to the solution. The reaction was extracted with ethyl acetate. The combined organic phases were dried over anhydrous sodium sulfate and evaporated in vacuo. The residue was purified by column chromatography over silica gel (hexanes : ethyl acetate; 80:20), affording **(*R*)-NOBIN** as a pale yellow solid, which was further recrystalized in EtOH to afford the pure product as a white solid (550 mg, 55% yield).

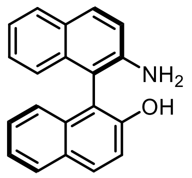

^{1}H NMR (300 MHz, $CDCl_3$) δ, ppm: 8.0 – 7.8 (m, 4H), 7.4 – 7.1 (m, 7H), 7.0 (d, *J* = 9.2 Hz, 1H), 5.1 (s, 1H), 3.7 (s, 2H). Data in agreement with those found in the literature.[1]

(*R*)-NOBIN

(*R*)-2'-amino-6,6'-dibromo-[1,1'-binaphthalen]-2-ol

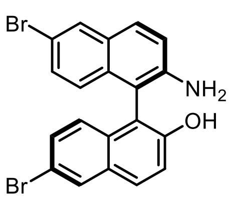

Obtained as a white solid (99 mg, 40% yield). **^{1}H NMR** (300 MHz, $CDCl_3$) δ, ppm: 8.0 (s, 1H), 7.9 (s, 1H), 7.8 (dd, *J* = 9.0, 4.7 Hz, 1H), 7.7 (d, *J* = 8.9 Hz, 1H), 7.4 – 7.2 (m, 3H), 7.1 (d, *J* = 8.8 Hz, 1H), 7.0 – 6.9 (m, 1H), 6.9 (d, *J* = 9.0 Hz, 1H), 5.3 (brs, 2H). Data in agreement with those found in the literature.[1]

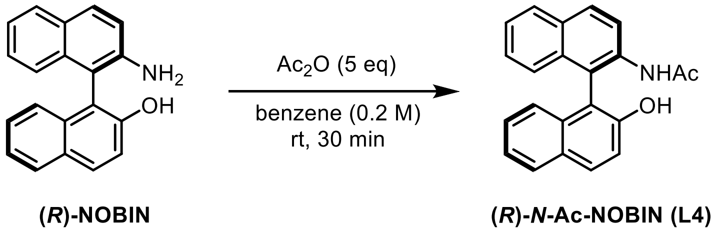

Following a previously reported procedure,[2] to a solution of (*R*)-NOBIN (150 mg, 0.53 mmol) in benzene (2.6 mL) was added acetic anhydride (0.25 mL, 5 eq) at room temperature. The reaction was stirred for 30 minutes. After complete conversion of the starting material, the solvent was evaporated under reduced pressure and the residue was purified by column chromatography over silica gel (hexanes : ethyl acetate; 60:40), affording **(*R*)-*N*-(2'-hydroxy-[1,1'-binaphthalen]-2-yl)acetamide (L4)** as a white solid (129 mg, 75% yield). **^{1}H NMR** (500 MHz, $CDCl_3$) δ, ppm: 8.54 (d, *J* = 9.0 Hz, 1H), 8.04 (d, *J* = 9.1 Hz, 1H), 7.98 (d, *J* = 8.9 Hz, 1H), 7.92 (t, *J* = 8.4 Hz, 2H), 7.48 – 7.41 (m, 1H), 7.42 – 7.34 (m, 2H), 7.33 – 7.24 (m, 2H), 7.15 (d, *J* = 8.5 Hz, 1H), 7.01 (d, *J* = 8.4 Hz, 1H), 6.91 (brs, 1H), 1.83 (s, 3H). **^{13}C NMR** (126 MHz, $CDCl_3$) δ, ppm: 169.1 (C), 152.1 (C), 135.9 (C), 133.2 (C), 132.9 (C), 131.5 (C), 131.3 (CH), 130.3 (CH), 129.5 (C), 128.6 (CH), 128.4 (CH), 127.6 (CH), 127.4 (CH), 125.7 (CH), 125.4 (CH), 124.3 (CH), 124.2 (CH), 121.4 (CH), 118.3 (C), 118.1 (CH), 113.1 (C), 24.7 (CH_3). **HRMS** (APCI, $[M+H]^+$) *m/z* calculated for $C_{22}H_{18}NO_2$: 328.1332; found 328.1343. **IR** (cm^{-1}): 3381, 3139, 1647, 1594, 1497, 1428, 1341, 1267, 819, 745, 504. **Rf** = 0.33 (hexane:AcOEt 50:50). **MP** = 209.1-210.5^{o}C.

(*R*)-tert-butyl (2'-hydroxy-[1,1'-binaphthalen]-2-yl)carbamate (L1)

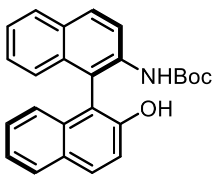

L1

Reaction performed with di-tert-butyl decarbonate (1 eq) instead of acetic anhydride. **L1** was obtained as a white solid (53 mg, 79% yield). **^{1}H NMR** (500 MHz, $CDCl_3$) δ, ppm: 8.50 (d, *J* = 9.1 Hz, 1H), 8.03 (d, *J* = 9.2 Hz, 1H), 7.98 (d, *J* = 8.8 Hz, 1H), 7.91 (dd, *J* = 8.1, 4.6 Hz, 2H), 7.43 – 7.35 (m, 3H), 7.31 – 7.23 (m, 2H), 7.10 – 7.06 (m, 1H), 7.05 – 7.01 (m, 1H), 6.25 (s, 1H), 5.02 (brs, 1H), 1.39 (s, 9H). **^{13}C NMR** (126 MHz, $CDCl_3$) δ, ppm: 153.2 (C), 152.1 (C), 136.6 (C), 133.4 (C), 133.1 (C), 131.2 (CH), 130.8 (C), 130.4 (CH), 129.5 (C), 128.5 (CH), 128.4 (CH), 127.4 (CH), 127.4 (CH), 125.14 (CH), 125.12 (CH), 124.5 (CH), 124.0 (CH), 120.1 (CH), 118.0 (CH), 116.3 (C), 113.2 (C), 81.1 (C), 28.3 (CH_3). **HRMS** (APCI, $[M+H]^+$) *m/z* calculated for $C_{25}H_{24}NO_3$: 386.1756.; found 386.1748. **Rf** = 0.30 (hexane:AcOEt 50:50). **MP** = 193.7-195.1^{o}C.

(*R*)-2,2,2-trifluoro-N-(2'-hydroxy-[1,1'-binaphthalen]-2-yl)acetamide (L2)

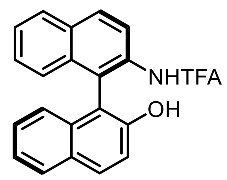

L2

Reaction performed with trifluoroacetic anhydride (1 eq) instead of acetic anhydride**. L2** was obtained as a white solid (47 mg, 70% yield). **^{1}H NMR** (500 MHz, $CDCl_3$) δ, ppm: 8.51 (d, *J* = 9.0 Hz, 1H), 8.09 (d, *J* = 9.0 Hz, 1H), 7.98 (dd, *J* = 13.5, 8.6 Hz, 2H), 7.91 (d, *J* = 8.2 Hz, 1H), 7.82 (brs, 1H), 7.51 (t, *J* = 7.5 Hz, 1H), 7.43 – 7.32 (m, 3H), 7.29 (t, *J* = 7.7 Hz, 2H), 6.98 (d, *J* = 8.4 Hz, 1H), 5.80 (brs, 1H). **^{19}F NMR** (282 MHz, $CDCl_3$) δ, ppm: -76.37. **^{13}C NMR** (126 MHz, $CDCl_3$) δ, ppm: 155.1 (q, *J* = 37.3 Hz, C), 152.2 (C), 132.9 (C), 132.8 (C), 132.7 (C), 132.1 (C), 131.9 (CH), 130.5 (CH), 129.4 (C), 128.7 (CH), 128.6 (CH), 127.9 (CH), 127.8 (CH), 126.5 (CH), 125.9 (CH), 124.3 (CH), 123.9 (CH), 121.3 (C), 120.2 (CH), 118.1 (CH), 115.5 (q, *J* = 288.9 Hz, C), 111.8 (C). **HRMS** (APCI, $[M+H]^+$) *m/z* calculated for $C_{22}H_{15}F_3NO_2$: 382.1049; found 382.1061. **Rf** = 0.48 (hexane:AcOEt 50:50). **MP** = 92.5-94.1°C.

(*R*)-N-(2'-hydroxy-[1,1'-binaphthalen]-2-yl)pivalamide (L3)

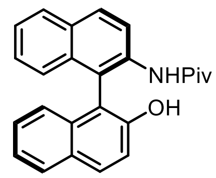

L3

Reaction performed with trimethylacetyl chloride (3 eq) instead of acetic anhydride. **L3** was obtained as a white solid (18 mg, 55% yield). **^{1}H NMR** (500 MHz, $CDCl_3$) δ, ppm: 8.59 (d, *J* = 9.0 Hz, 1H), 8.03 (d, *J* = 9.0 Hz, 1H), 7.95 (d, *J* = 8.9 Hz, 1H), 7.91 (d, *J* = 8.2 Hz, 1H), 7.87 (d, *J* = 8.1 Hz, 1H), 7.5 – 7.4 (m, 1H), 7.37 (d, *J* = 8.9 Hz, 1H), 7.36 – 7.21 (m, 4H), 7.20 (brs, 1H), 7.02 (d, *J* = 8.4 Hz, 1H), 5.09 (brs, 1H), 0.77 (s, 9H). **^{13}C NMR** (126 MHz, $CDCl_3$) δ, ppm: 177.1 (C), 152.1 (C), 136.2 (C), 133.0 (C), 132.8 (C), 131.3 (C), 131.2 (CH), 130.3 (CH), 129.4 (C), 128.6 (CH), 128.5 (CH), 127.7 (CH), 127.5 (CH), 125.5 (CH), 125.20 (CH), 124.18 (CH), 124.2 (CH), 121.1 (CH), 118.02 (CH), 117.98 (C), 113.1 (C), 39.7 (C), 27.1 (CH_3). **HRMS** (APCI, $[M+H]^+$) *m/z* calculated for $C_{25}H_{24}NO_2$: 370.1802; found 370.1810. **Rf** = 0.37 (hexane:AcOEt 50:50). **MP** = 205.1-206.5°C.

(*R*)-2,6-difluoro-N-(2'-hydroxy-[1,1'-binaphthalen]-2-yl)benzamide (L5)

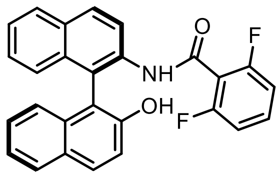

L5

Reaction performed with 2,6-difluorobenzoyl chloride (1 eq) instead of acetic anhydride. **L5** was obtained as a white solid (39 mg, 52% yield). **^{1}H NMR** (500 MHz, $CDCl_3$) δ, ppm: 8.67 (d, *J* = 9.0 Hz, 1H), 8.04 (d, *J* = 9.1 Hz, 1H), 7.88 (d, *J* = 8.7 Hz, 2H), 7.80 (d, *J* = 8.1 Hz, 1H), 7.48 – 7.36 (m, 2H), 7.33 – 7.09 (m, 6H), 6.95 (d, *J* = 8.4 Hz, 1H), 6.71 (t, *J* = 8.5 Hz, 1H), 5.05 (s, 1H). **^{19}F NMR** (282 MHz, $CDCl_3$) δ, ppm: -111.77. **^{13}C NMR** (126 MHz, $CDCl_3$) δ, ppm: 167.9 (C), 161.2 (d, *J* = 6.6 Hz, C), 159.2 (d, *J* = 6.2 Hz, C), 158.9 (C), 152.3 (C), 135.5 (C), 133.3 (C), 133.0 (C), 132.3 (t, *J* = 10.5 Hz, CH), 131.8 (C), 131.4 (CH), 130.5 (CH), 129.4 (C), 128.48 (CH), 128.46 (CH), 127.6 (CH), 126.0 (CH), 125.6 (CH), 124.3 (CH), 124.1 (CH), 121.5 (CH), 118.1 (CH), 113.8 (t, *J* = 18.2 Hz, C), 112.7 (C), 112.3 (d, *J* = 4.3 Hz, CH), 112.2 (d, *J* = 4.1 Hz, CH). **HRMS** (APCI, $[M+H]^+$) *m/z* calculated for $C_{27}H_{18}F_2NO_2$: 426.1300; found 426.1301. **Rf** = 0.38 (hexane:AcOEt 50:50). **MP** = 243.1-244.7°C.

(*R*)-N-(2'-hydroxy-[1,1'-binaphthalen]-2-yl)-3,5-dimethylbenzamide (L6)

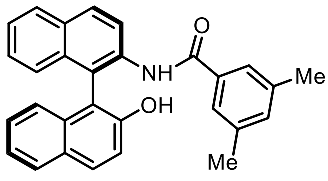

L6

Reaction performed with 3,5-dimethylbenzoyl chloride (1 eq) instead of acetic anhydride. **L6** was obtained as a white solid (14 mg, 39% yield). **^{1}H NMR** (500 MHz, $CDCl_3$) δ, ppm: 8.90 (d, *J* = 9.0 Hz, 1H), 8.12 (d, *J* = 9.1 Hz, 1H), 8.02 (d, *J* = 8.9 Hz, 1H), 7.96 (t, *J* = 8.1 Hz, 2H), 7.73 (s, 1H), 7.50 – 7.45 (m, 2H), 7.45 – 7.30 (m, 5H), 7.17 (d, *J* = 8.4 Hz, 1H), 6.98 (s, 1H), 6.71 (s, 2H), 5.14 (brs, 1H), 2.13 (s, 6H). **^{13}C NMR** (126 MHz, $CDCl_3$) δ, ppm: 165.9 (C), 152.3 (C), 138.5 (C), 136.4 (C), 134.7 (C), 133.4 (CH), 133.0 (C), 132.8 (C), 131.4 (CH), 131.3 (C), 130.7 (CH), 129.6 (C), 128.65 (CH), 128.63 (CH), 128.0 (CH), 127.6 (CH), 125.6 (CH), 125.2 (CH), 124.7 (CH), 124.4 (CH), 120.4 (CH), 118.2 (CH), 117.3 (C), 113.0 (C), 21.2 (CH_3). **HRMS** (APCI, $[M+H]^+$) *m/z* calculated for $C_{29}H_{24}NO_2$: 418.1802; found 418.1809. **Rf** = 0.37 (hexane:AcOEt 50:50). **MP** = 272.6-274.2^{o}C.

(*R*)-N-(6,6'-dibromo-2'-hydroxy-[1,1'-binaphthalen]-2-yl)acetamide (L7)

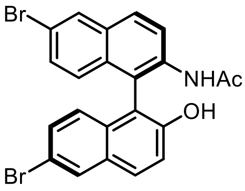

L7

L7 was obtained as a white solid (99 mg, 92% yield). **^{1}H NMR** (500 MHz, DMSO) δ, ppm: 9.80 (s, 1H), 8.62 (s, 1H), 8.23 (d, *J* = 2.1 Hz, 1H), 8.16 (d, *J* = 2.1 Hz, 1H), 8.08 (d, *J* = 9.1 Hz, 1H), 7.95 (dd, *J* = 12.7, 9.0 Hz, 2H), 7.43 – 7.36 (m, 2H), 7.30 (dd, *J* = 9.0, 2.1 Hz, 1H), 6.90 (d, *J* = 9.1 Hz, 1H), 6.73 (d, *J* = 9.0 Hz, 1H), 1.75 (s, 3H). **^{13}C NMR** (126 MHz, DMSO) δ, ppm: 168.7 (C), 153.9 (C), 135.8 (C), 132.3 (C), 132.0 (C), 131.2 (C), 129.8 (CH), 129.6 (CH), 129.4 (C), 129.2 (CH), 129.1 (CH), 129.0 (CH), 127.8 (CH), 126.8 (CH), 126.1 (CH), 125.2 (CH), 124.0 (C), 119.8 (CH), 118.0 (C), 115.4 (C), 113.6 (C), 23.3 (CH_3). **HRMS** (APCI, $[M+H]^+$) *m/z* calculated for $C_{22}H_{16}Br_2NO_2$: 483.9542; found 483.9547. **Rf** = 0.38 (hexane:AcOEt 50:50). **MP** = 178.7-180.1^{o}C.

(*R*)-methyl 2'-acetamido-2-hydroxy-[1,1'-binaphthalene]-3-carboxylate (L8)

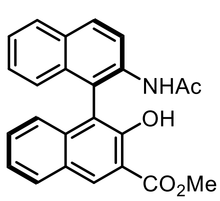

L8

L8 was obtained as a white solid (43.8 mg, 81% yield**). ^{1}H NMR** (300 MHz, $CDCl_3$) δ, ppm: 10.82 (s, 1H), 8.75 (s, 1H), 8.50 (d, *J* = 8.9 Hz, 1H), 8.01 (d, *J* = 9.0 Hz, 1H), 7.93 (t, *J* = 8.9 Hz, 2H), 7.38 (m, 3H), 7.22 (m, 1H), 7.07 (m, 3H), 4.08 (s, 3H), 1.82 (s, 3H). **^{13}C NMR** (75 MHz, $CDCl_3$) δ, ppm: 170.5 (C), 168.5 (C), 154.1 (C), 137.0 (C), 134.7 (C), 133.8 (CH), 132.8 (C), 131.3 (C), 130.3 (CH), 129.9 (CH), 129.2 (CH), 128.3 (CH), 127.4 (C), 126.6 (CH), 125.6 (CH), 125.0 (CH), 124.8 (CH), 124.7 (CH), 121.6 (CH), 120.9 (C), 116.4 (C), 114.3 (C), 53.0 (CH_3), 24.6 (CH_3). **HRMS** (APCI, $[M+H]^+$) *m/z* calculated for $C_{24}H_{20}NO_4$: 386.1387; found: 386.1387. **Rf** = 0.31 (hexane:AcOEt 40:60). **MP** = 109.1-110.4^{o}C.

Synthesis of L9 via OH methylation

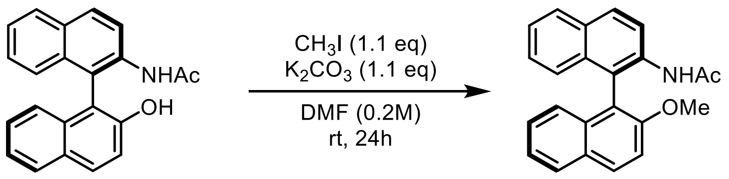

L4 **L9**

In a flame-dried round botton flask, (*R*)-*N*-(2'-hydroxy-[1,1'-binaphthalen]-2-yl)acetamide (20 mg, 0.06 mmol) and potassium carbonate (9.3 mg, 1.1 eq) were added under Ar atmosphere. After that, DMF (0.6 mL) was injected to the flask. The solution was stirred during 20 minutes at rt, getting progressively yellow likely due to phenoxide formation. After that, CH_3I (4 µL, 1.1 eq) was added and the reaction was stirred for 24 hours until complete conversion. Then, the reaction was quenched with water and extracted with ethyl acetate. The combined organic phases were dried over anhydrous sodium sulfate and evaporated in vacuo. The residue was purified by column chromatography over silica gel (hexanes :ethyl acetate; 60:40), affording **(*R*)- N-(2'-methoxy-[1,1'-binaphthalen]-2-yl)acetamide (L8)** as a white solid (19 mg, 89% yield). **^{1}H NMR** (500 MHz, $CDCl_3$) δ, ppm: 8.48 (d, *J* = 8.9 Hz, 1H), 7.98 (d, *J* = 9.1 Hz, 1H), 7.90 (d, *J* = 9.0 Hz, 1H), 7.82 (t, *J* = 8.2 Hz, 2H), 7.41 (d, *J* = 9.1 Hz, 1H), 7.29 (t, *J* = 7.3 Hz, 2H), 7.21 – 7.09 (m, 2H), 7.03 – 6.92 (m, 2H), 6.81 (brs, 1H), 3.70 (s, 3H), 1.73 (s, 3H). **^{13}C NMR** (126 MHz, $CDCl_3$) δ, ppm: 168.4 (C), 155.3 (C), 134.6 (C), 133.7 (C), 133.1 (C), 131.09 (C), 131.05 (CH), 129.4 (C), 128.8 (CH), 128.3 (CH), 128.2 (CH), 127.5 (CH), 126.4 (CH), 125.8 (CH), 125.0 (CH), 124.8 (CH), 124.4 (CH), 121.5 (C), 121.0 (CH), 117.3 (C), 113.8 (CH), 56.8 (CH_3), 24.7 (CH_3). **HRMS** (APCI, $[M+H]^+$) *m/z* calculated for $C_{23}H_{20}NO_2$: 342.1489; found 342.1487. **Rf** = 0.68 (hexane:AcOEt 50:50). **MP** = 191.3-193.2^{o}C.

Synthesis of L10

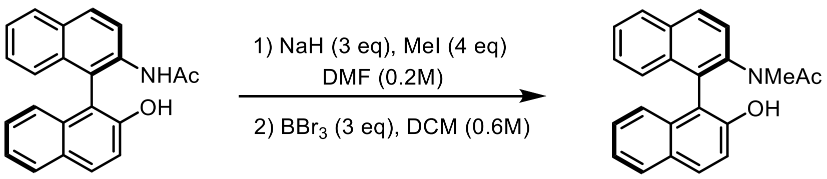

L4 **L10**

In a flame-dried Schlenk flask were added **L4** (98.2 mg, 0.3 mmol, 1 eq) and anhydrous DMF (1.5 mL, 0.2M) under Ar. NaH (36 mg, 60% in mineral oil, 0.9 mmol, 3 eq) was then added at 0°C and stirred for 30 min. MeI (75 µL, 1.2 mmol, 4 eq) was then added at 0°C. The reaction was then warmed up to room temperature and stirred for 6 hours. The reaction was quenched by adding water and extracted with EtOAc. The combined organic extracts were washed with water and brine, dried with Na_2SO_4, filtrated, and concentrated in vacuo to give the methylated product as a white solid (106 mg, 99%), which was used in the next synthetic step without further purification.

In a flame-dried Schlenk flask were added the methylated product previously obtained (71.1 mg, 0.2 mmol, 1 eq) and anhydrous DCM (0.33 mL, 0.6M) under Ar, and the solution was cooled to -78°C. A solution of BBr_3 in DCM (0.6 mL, 1.0 M, 0.6 mmol, 3 eq) was added dropwise at −78°C. After stirring the reaction for another 1 hour at −78°C, the reaction was slowly warmed up to room temperature and stirred for additional 12 hours. Water was slowly added to quench the reaction and the mixture was extracted with DCM. The combined organic extracts were washed with water and brine, dried with Na_2SO_4, filtrated, and concentrated in vacuo. The obtained residue was purified by flash column chromatography (silica gel, hexane:EtOAc 60:40 to 30:70) to finally obtain **L10** as a white solid.

***N*-(2'-hydroxy-[1,1'-binaphthalen]-2-yl)-*N*-methylacetamide (L10)**

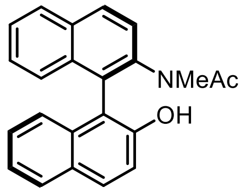

L10

L10 was obtained as a white solid (20.6 mg, 30% yield). Rotamers were observed. **^{1}H NMR** (400 MHz, $C_2D_2Cl_4$, 353K) δ, ppm: 6.86 (d, *J* = 8.6 Hz, 1H), 6.79 – 6.73 (m, 1H), 6.66 (t, *J* = 9.8 Hz, 2H), 6.38 – 6.27 (m, 1H), 6.22 (d, *J* = 8.6 Hz, 1H), 6.17 – 6.05 (m, 4H), 5.99 (t, *J* = 7.7 Hz, 1H), 5.73 (d, *J* = 8.6 Hz, 1H), 1.72 (s, 3H), 0.78 (s, 3H). **^{13}C NMR** (100 MHz, $C_2D_2Cl_4$, 353K) δ, ppm: 172.7 (C), 170.6 (C), 153.0 (C), 141.3 (C), 133.6 (CH), 133.0 (CH), 130.3 (CH), 129.8 (CH), 128.0 (CH), 127.9 (CH), 126.9 (CH), 126.5 (CH), 126.2 (CH), 124.4 (CH), 123.2 (CH), 119.9 (CH), 117.9 (C), 116.9 (C), 60.1 (CH_3), 13.8 (CH_3). **HRMS** (APCI, $[M+H]^+$) *m/z* calculated for $C_{23}H_{20}NO_2$: 342.1489; found: 342.1492. **Rf** = 0.52 (hexane:AcOEt 40:60). **MP** = 169.1-171.9°C.

Synthesis of L11

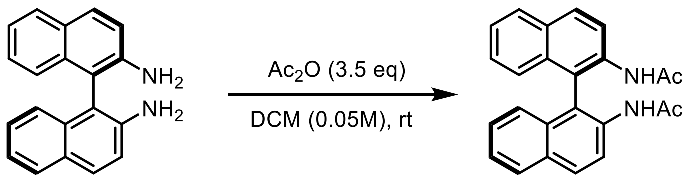

(*R*)-BINAM L11

Following a procedure previously reported in the literature,[3] **L11** was obtained as a white solid (91 mg, 99% yield). **^{1}H NMR** (300 MHz, $CDCl_3$) δ, ppm: 8.35 (d, *J* = 8.5 Hz, 2H), 8.05 (d, *J* = 9.0 Hz, 2H), 7.95 (d, *J* = 8.2 Hz, 2H), 7.46 (t, *J* = 7.5 Hz, 2H), 7.28 (td, *J* = 1.2, 6.8 Hz, 2H), 7.03 (d, *J* = 8.4 Hz, 2H), 6.92 (brs, 2H), 1.84 (s, 6H). Data in agreement with with those found in the literature.[3]

3. Synthesis of starting materials and allenes

3.1. Synthesis of symmetrical homobenzyltriflamides

General procedure for the preparation of symmetrical homobenzyltriflamides (1a-1i)

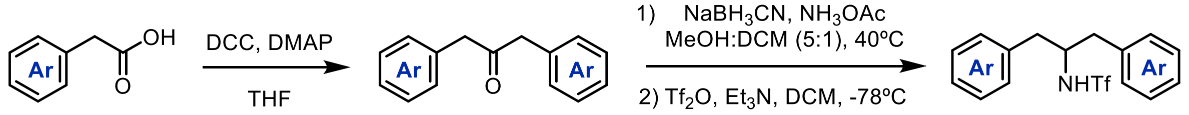

Following literature procedures,[4] the corresponding phenylacetic acid (1 eq) was added to a stirred solution of DCC (1 eq) and DMAP (0.3 eq) in THF (0.3M) under argon atmosphere at room temperature and the reaction was stirred for 1h. Then, the mixture was filtered through a Celite® plug and washed with Et_2O. After evaporation of volatiles under vacuum, the crude was purified by flash column chromatography (silica gel, hexane/EtOAc 90:10 to 70:30) to obtain the symmetric ketone.

To a solution of the obtained ketone (1 eq) in MeOH/DCM (0.15M, v/v = 5/1), $NaBH_3CN$ (1 eq) and NH_4OAc (10 eq) were added. The reaction flask was stirred at 40ºC overnight. Aqueous NaOH solution (10%) was added into the reaction mixture, which was then extracted with DCM. The combined organic layers were washed with aqueous NaCl solution, and then dried over Na_2SO_4. The organic solvent was concentrated by rotary evaporation to afford the corresponding free amine which was used in the next synthetic step without further purification.

The obtained amine (1 eq) was dissolved in dry DCM (0.5M), and the reaction mixture was cooled to -78ºC. Triethylamine (1 eq) was added and the reaction mixture was stirred at -78ºC for 5 min. Trifluoromethanesulfonic anhydride (1.05 eq) was then added dropwise and the reaction mixture was stirred at -78ºC for 30 min, and 2 hours at room temperature before quenching with water. The organic layer was separated, and the aqueous layer was extracted with DCM. The combined organics were washed with brine, dried over Na_2SO_4, filtered, and then concentrated in vacuo. The obtained trifluoromethanesulfonamide was purified by flash column chromatography (silica gel, hexane:Et_2O 95:5 to 70:30).

***N*-(1,3-diphenylpropan-2-yl)-1,1,1-trifluoromethanesulfonamide (1a)**

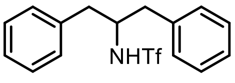

1a

1a was obtained as a white solid upon freezing (1.2 g, 38% yield). **^{1}H NMR** (300 MHz, $CDCl_3$) δ, ppm: 7.42 – 7.26 (m, 6H), 7.25 – 7.16 (m, 4H), 4.80 (brs, 1H), 4.14 – 4.01 (m, 1H), 2.99 – 2.78 (m, 4H). **^{19}F NMR** (282 MHz, $CDCl_3$) δ, ppm: -77.65 (s). **^{13}C NMR** (75 MHz, $CDCl_3$) δ, ppm: 136.1 (C), 129.7 (CH), 128.9 (CH), 127.4 (CH), 119.4 (q, J = 320.9 Hz, C), 58.6 (CH), 41.2 (CH_2). **HRMS** (APCI, $[M-H]^-$) *m/z* calculated for $C_{16}H_{15}F_3NO_2S$: 342.0781; found 342.0786. **IR** (cm^{-1}): 3287, 3030, 1451, 1430, 1370, 1357, 1190, 1142, 1048, 695, 608, 489. **Rf** = 0.44 (hexane:Et_2O 60:40). **MP** = 64.3-65.7ºC.

_N_-(1,3-bis(2-fluorophenyl)propan-2-yl)-1,1,1-trifluoromethanesulfonamide (1b)

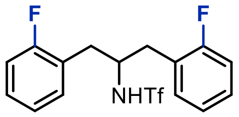

1b

1b was obtained as a white solid upon freezing (460 mg, 40% yield). **^{1}H NMR** (300 MHz, $CDCl_3$) δ, ppm: 7.35 – 7.02 (m, 8H), 4.89 (brs, 1H), 4.15 – 3.99 (m, 1H) 3.07 – 2.80 (m, 4H). **^{19}F NMR** (282 MHz, $CDCl_3$) δ, ppm: -78.27 (s), 117.40 (s). **^{13}C NMR** (75 MHz, $CDCl_3$) δ, ppm: 161.5 (d, J = 244.7 Hz, C), 131.9 (d, J = 4.4 Hz, CH), 129.4 (d, J = 8.3 Hz, CH), 124.6 (d, J = 3.6 Hz, CH), 123.4 (d, J = 15.6 Hz, C), 119.3 (q, J = 320.7 Hz, C), 115.73 (d, J = 22.2 Hz, CH), 57.8 (CH), 35.3 (CH_2). **HRMS** (APCI, $[M-H]^-$) m/z calculated for $C_{16}H_{13}F_5NO_2S$: 378.0593; found 378.0589. **Rf** = 0.49 (hexane:Et_2O 50:50). **MP** = 92.4-93.5^{o}C.

_N_-(1,3-bis(2-chlorophenyl)propan-2-yl)-1,1,1-trifluoromethanesulfonamide (1c)

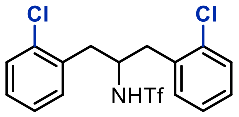

1c

1c was obtained as a white solid upon freezing (480 mg, 40% yield). **^{1}H NMR** (300 MHz, $CDCl_3$) δ, ppm: 7.43 – 7.34 (m, 2H), 7.28 – 7.18 (m, 6H), 5.13 (brs, 1H), 4.30 – 4.14 (m, 1H), 3.19 – 2.92 (m, 4H). **^{19}F NMR** (282 MHz, $CDCl_3$) δ, ppm: -78.23 (s). **^{13}C NMR** (75 MHz, $CDCl_3$) δ, ppm: 134.5 (C), 134.4 (C), 131.9 (CH), 130.0 (CH), 128.1 (CH), 125.6 (CH), 119.2 (q, J = 321.1 Hz, C), 57.6 (CH), 39.7 (CH_2). **HRMS** (APCI, $[M-H]^-$) m/z calculated for $C_{16}H_{13}F_3Cl_2NO_2S$: 410.0002; found 410.0014. **Rf** = 0.59 (hexane:Et_2O 60:40). **MP** = 126.1-127.0^{o}C.

_N_-(1,3-bis(3-bromophenyl)propan-2-yl)-1,1,1-trifluoromethanesulfonamide (1d)

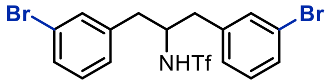

1d

1d was obtained as a yellow solid upon freezing (380 mg, 47% yield). **^{1}H NMR** (300 MHz, $CDCl_3$) δ, ppm: 7.49 – 7.42 (m, 2H), 7.36 – 7.32 (m, 2H), 7.28 – 7.20 (m, 2H), 7.16 – 7.09 (m, 2H), 4.84 (d, J = 7.8 Hz, 1H), 4.11 – 3.93 (m, 1H), 2.96 – 2.74 (m, 4H). **^{19}F NMR** (282 MHz, $CDCl_3$) δ, ppm: -77.82 (s). **^{13}C NMR** (75 MHz, $CDCl_3$) δ, ppm: 138.2 (C), 132.7 (CH), 130.7 (CH), 130.5 (CH), 128.2 (CH), 123.0 (C), 119.3 (q, J = 320.8 Hz, C), 58.4 (CH), 41.1 (CH_2). **HRMS** (APCI, $[M-H]^-$) m/z calculated for $C_{16}H_{13}F_3Br_2NO_2S$: 497.8991; found 497.8991. **Rf** = 0.42 (hexane:Et_2O 60:40). **MP** = 95.1-96.9^{o}C.

_N_-(1,3-bis(4-bromophenyl)propan-2-yl)-1,1,1-trifluoromethanesulfonamide (1e)

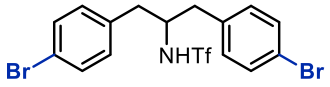

1e

1e was obtained as a yellowish solid upon freezing (450 mg, 35% yield). **^{1}H NMR** (300 MHz, $CDCl_3$) δ, ppm: 7.47 (d, J = 7.9 Hz, 4H), 7.04 (d, J = 7.9 Hz, 4H), 4.70 (brs, 1H), 4.07 – 3.90 (m, 1H), 2.93 – 2.67 (m, 4H). **^{19}F NMR** (282 MHz, $CDCl_3$) δ, ppm: -78.78 (s). **^{13}C NMR** (75 MHz, $CDCl_3$) δ, ppm: 134.9 (C), 132.2 (CH), 131.3 (CH), 121.5 (C), 119.3 (q, J = 321.0 Hz, C), 58.2 (CH), 40.7 (CH_2). **HRMS** (APCI, $[M-H]^-$) m/z calculated for $C_{16}H_{13}Br_2F_3NO_2S$: 497.8991; found 497.9002. **Rf** = 0.44 (hexane:Et_2O 60:40). **MP** = 127.6-128.9^{o}C.

***N*-(1,3-bis(4-(trifluoromethyl)phenyl)propan-2-yl)-1,1,1-trifluoromethanesulfonamide (1f)**

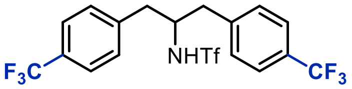

1f

1f was obtained as a yellowish solid upon freezing (460 mg, 48% yield). **^{1}H NMR** (300 MHz, $CDCl_3$) δ, ppm: 7.63 (d, *J* = 8.0 Hz, 4H), 7.31 (d, *J* = 7.9 Hz, 4H), 4.88 (brs, 1H), 4.16 – 4.02 (m, 1H), 3.06 – 2.84 (m, 4H). **^{19}F NMR** (282 MHz, $CDCl_3$) δ, ppm: -62.67 (s), -77.92 (s). **^{13}C NMR** (75 MHz, $CDCl_3$) δ, ppm: 140.0 (C), 130.0 (q, *J* = 33.62 Hz, C), 130.0 (CH), 126.0 (q, *J* = 3.8 Hz, CH), 124.1 (q, *J* = 260.0 Hz, C), 119.3 (q, *J* = 320.5 Hz, C), 58.3 (CH), 41.4 (CH_2). **HRMS** (APCI, $[M-H]^-$) *m/z* calculated for $C_{18}H_{13}F_9NO_2S$: 478.0529; found 478.0528. **Rf** = 0.55 (hexane:Et_2O 40:60). **MP** = 84.3-85.9^{o}C.

***N*-(1,3-bis(4-methoxyphenyl)propan-2-yl)-1,1,1-trifluoromethanesulfonamide (1g)**

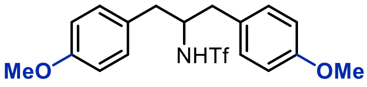

1g

1g was obtained as an orange oil (600 mg, 30% yield). **^{1}H NMR** (300 MHz, $CDCl_3$) δ, ppm: 7.10 (d, *J* = 8.6 Hz, 4H), 6.88 (d, *J* = 8.7 Hz, 4H), 4.68 (d, *J* = 8.8 Hz, 1H), 3.81 (s, 6H), 2.91 – 2.71 (m, 4H). **^{19}F NMR** (282 MHz, $CDCl_3$) δ, ppm: -77.84 (s). **^{13}C NMR** (75 MHz, $CDCl_3$) δ, ppm: 158.9 (C), 130.8 (CH), 128.1 (C), 119.5 (q, *J* = 320.7 Hz, C), 114.5 (CH), 58.6 (CH), 55.4 (CH_3), 40.0 (CH_2). **HRMS** (APCI, $[M-H]^-$) *m/z* calculated for $C_{18}H_{19}F_3NO_4S$: 402.0992; found 402.1002. **Rf** = 0.51 (hexane:Et_2O 40:60).

***N*-(1,3-di-p-tolylpropan-2-yl)-1,1,1-trifluoromethanesulfonamide (1h)**

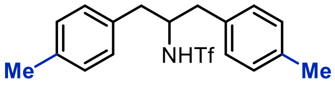

1h

1h was obtained as a yellowish solid (250 mg, 56% yield). **^{1}H NMR** (300 MHz, $CDCl_3$) δ, ppm: 7.17 (d, *J* = 7.8 Hz, 4H), 7.08 (d, *J* = 8.1 Hz, 4H), 4.04 (p, *J* = 6.3 Hz, 1H), 2.93 – 2.71 (m, 4H), 2.36 (s, 6H). **^{19}F NMR** (282 MHz, $CDCl_3$) δ, ppm: -77.84 (s). **^{13}C NMR** (75 MHz, $CDCl_3$) δ, ppm: 137.0 (C), 133.0 (C), 129.6 (CH), 119.5 (q, *J* = 321.0 Hz, C), 58.3 (CH), 40.4 (CH_2), 21.2 (CH_3). **HRMS** (APCI, $[M-H]^-$) *m/z* calculated for $C_{18}H_{19}F_3NO_2S$: 370.1094; found 370.1096. **Rf** = 0.49 (hexane:Et_2O 60:40). **MP** = 86.5-88.4^{o}C.

***N*-(1,3-di(naphthalen-1-yl)propan-2-yl)-1,1,1-trifluoromethanesulfonamide (1i)**

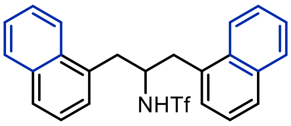

1i

1i was obtained as a yellow solid upon freezing (430 mg, 33% yield). **^{1}H NMR** (300 MHz, $CDCl_3$) δ, ppm: 7.95 – 7.90 (m, 1H), 7.86 (d, *J* = 8.2 Hz, 1H), 7.59 (d, *J* = 8.5 Hz, 1H) 7.54 – 7.44 (m, 3H), 7.41 – 7.32 (m, 2H) 5.16 (brs, 1H), 4.50 – 4.32 (m, 1H), 3.53 – 3.25 (m, 4H). **^{19}F NMR** (282 MHz, $CDCl_3$) δ, ppm: -78.09 (s). **^{13}C NMR** (75 MHz, $CDCl_3$) δ, ppm: 134.2 (C), 132.8 (C), 131.9 (C), 129.1 (CH), 128.3 (CH), 128.3 (CH), 126.6 (CH), 126.0 (CH), 125.4 (CH), 123.2 (CH), 119.0 (q, *J* = 321.1 Hz, C), 57.8 (CH), 39.7 (CH_2). **HRMS** (APCI, $[M-H]^-$) *m/z* calculated for $C_{24}H_{19}F_3NO_2S$: 442.1094; found 442.1097. **Rf** = 0.48 (hexane:Et_2O 40:60). **MP** = 127.7-128.9^{o}C.

3.2. Synthesis of non-symmetrical homobenzyltriflamides

Procedure for the synthesis of homobenzyltriflamide *rac*-1j

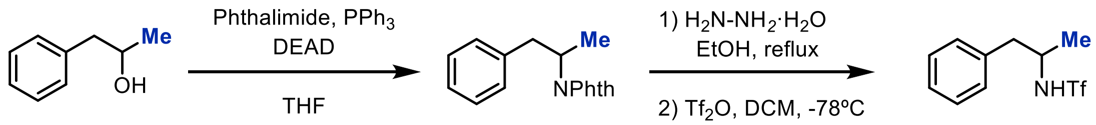

rac-**1j**

To a solution of 1-phenylpropan-2-ol (1 eq), triphenylphosphine (1.5 eq), and phthalimide (1 eq) anhydrous THF (0.25M) was added dropwise DEAD (40% in toluene, 1.5 eq) at 0°C. . After stirring over night at room temperature, the solvent was removed in vacuum. The crude reaction mixture was purified by flash column chromatography (silica gel, hexane:EtOAc 95:5) to obtain the phthalimide.

To a solution of the obtained phthalimide (1 eq) in EtOH (0.1 M) was added hydrazine hydrate (6 eq) dropwise and the resulting mixture was stirred at reflux until the starting material had been completely consumed as judged by TLC analysis (around 2h). The formed pasty precipitate was then filtered through a plug of celite, washed with abundant Et_2O and the solvent was evaporated under reduced pressure. The obtained amine was used in the next synthetic step without further purification.

The crude amine (1 equiv) was dissolved in dry DCM (0.5 M), and the reaction mixture was cooled to -78°C. Triethylamine (1 eq) was added and the reaction mixture was stirred -78°C for 5 min. Trifluoromethanesulfonic anhydride (1.05 eq) was then added dropwise and the reaction mixture was stirred at -78°C for 2 hours, before quenching with water. The organic layer was separated, and the aqueous layer was extracted with DCM. The combined organics were washed with brine, dried over Na_2SO_4, filtered, and then concentrated in vacuo. The obtained trifluoromethanesulfonamide was purified by flash column chromatography (silica gel, hexane:Et_2O 95:5 to 80:20).

1,1,1-trifluoro-*N*-(1-phenylpropan-2-yl)methanesulfonamide (*rac*-1j)

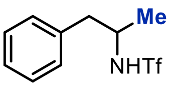

rac-**1j**

rac-**1j** was obtained as a white solid upon freezing (1.2 g, 90% yield). **^{1}H NMR** (300 MHz, $CDCl_3$) δ, ppm: 7.39 – 7.24 (m, 3H), 7.22 – 7.16 (m, 2H), 4.85 (brs, 1H), 3.99 – 3.87 (m, 1H), 2.96 – 2.75 (m, 2H), 1.27 (d, *J* = 6.6 Hz, 3H). **^{19}F NMR** (282 MHz, $CDCl_3$) δ, ppm: -77.70. **^{13}C NMR** (75 MHz, $CDCl_3$) δ, ppm: 136.1 (C), 129.7 (CH), 128.9 (CH), 127.3 (CH), 119.7 (q, *J* = 320.9 Hz, C), 53.3 (CH), 43.9 (CH_2), 21.5 (CH_3). **HRMS** (APCI, $[M-H]^-$) *m/z* calculated for $C_{10}H_{11}F_3NO_2S$: 266.0468; found 266.0453. **Rf** = 0.48 (hexane:Et_2O 60:40). **MP** = 62.3-64.2^{o}C.

3.3. Synthesis of α-substituted *ortho*-methyl benzyltriflamides

General procedure for the preparation α-substituted *ortho*-methyl benzyltriflamides *rac*-7a and *rac*-7b

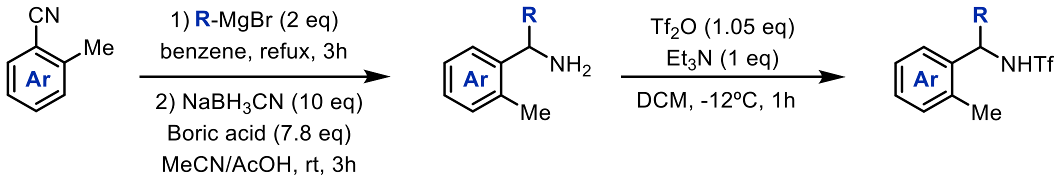

Following a reported procedure,[5] to a solution of the Grignard reagent (2 eq) in benzene (1.5 M) under Ar was added the nitrile (1 eq) at room temperature, with the resulting mixture being refluxed for 3h. After that, the reaction was cooled to 0°C and quenched with the careful addition of NH_4Cl (sat). The aqueous layer was extracted with DCM. The combined organic phases were washed with aqueous brine and dried over Na_2SO_4. The crude was purified by column chromatography (hexanes:ethyl acetate 60:40) affording the corresponding bench-stable imine, which was used directly in the next step of the synthesis.

The imine synthesized in the previous step (1 eq) was dissolved, under Ar atmosphere, in acetonitrile (1.5M). To this stirred solution was added sodium cyanoborohydride (10 eq) dissolved in acetonitrile (1.5M), followed by boric acid (7.8 eq). A mixture of glacial acetic acid (3.5 eq) and acetonitrile (1:1) was slowly added to the reaction mixture, followed by another dose of the same mixture after 2h. After 1h, the reaction was cooled to 0°C and carefully quenched with an aqueous solution of NaOH (60% wt). The aqueous layer was extracted with $CHCl_3$. The combined organic phases were washed with $NaHCO_3$ (sat.) and dried over Na_2SO_4. The crude was used for the next step without further purification.

To a solution of the corresponding *o*-methylbenzylamine (1 eq) in dichloromethane (0.5M) under argon atmosphere was added triethylamine (1 eq) at -12℃. After the solution was stirred 5 minutes at that temperature, trifluoromethanesulfonic anhydride (1.05 eq) was added dropwise. The reaction was stirred for 1h at that temperature before being quenched with water. The organic layer was separated, and the aqueous layer extracted with dichloromethane. The combined organic phases were washed with brine and then dried over sodium sulfate. Evaporation and column chromatography on silica gel (hexanes : ethyl acetate; 95:5) afforded the corresponding *ortho*-methyl benzyltriflamide.

1,1,1-trifluoro-*N*-(1-mesitylethyl)methanesulfonamide (*rac*-7a)

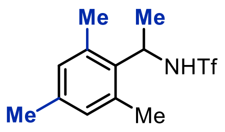

rac-**7a**

rac-**7a** was obtained as a white solid (354 mg, 46% yield over two steps). **^{1}H NMR** (300 MHz, $CDCl_3$) δ, ppm: 6.85 (s, 2H), 5.44 – 5.32 (m, 1H), 5.32 – 5.19 (m, 1H), 2.39 (s, 6H), 2.26 (s, 3H), 1.63 (d, *J* = 6.5 Hz, 3H). **^{19}F NMR** (282 MHz, $CDCl_3$) δ, ppm: -77.88 (s). **^{13}C NMR** (75 MHz, $CDCl_3$) δ, ppm: 137.6 (C), 134.9 (C), 134.2 (C), 130.7 (CH), 119.6 (d, *J* = 321.1 Hz), 51.6 (CH), 21.5 (CH_3), 20.9 (CH_3), 20.7 (CH_3). **HRMS** (APCI, $[M]^+$) *m/z* calculated for $C_{12}H_{16}F_3NO_2S$: 295.0848, found 295.0850. **IR** (cm^{-1}): 3326, 2970, 1428, 1389, 1372, 1348, 1230, 1180, 1146, 1077, 1008, 982, 848, 620. **Rf** = 0.51 (hexane:Et_2O 60:40). **MP** = 72.8-73.9^{o}C.

***N*-(1-(2-chloro-6-methylphenyl)ethyl)-1,1,1-trifluoromethanesulfonamide (*rac*-7b)**

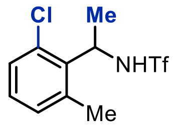

rac-**7b**

rac-**7b** was obtained as a white solid (343 mg, 32% yield over two steps). **^{1}H NMR** (500 MHz, $CDCl_3$) δ, ppm: 7.18 – 7.15 (m, 1H), 7.10 – 7.01 (m, 2H), 6.18 (brs, 1H), 5.17 (brs, 1H), 2.35 (s, 3H), 1.63 (d, *J* = 7.2 Hz, 3H). **^{19}F NMR** (282 MHz, $CDCl_3$) δ, ppm: -78.2 (s). **^{13}C NMR** (126 MHz, $CDCl_3$) δ, ppm: 137.5 (C), 136.1 (C), 132.5 (C), 130.0 (CH), 129.4 (CH), 129.1 (CH), 119.6 (q, *J* = 320.9 Hz, C), 51.3 (CH), 20.8 (CH_3), 20.4 (CH_3). **HRMS** (APCI, $[M-H]^-$) *m/z* calculated for $C_{10}H_{10}ClF_3NO_2S$: 300.0078; found 300.0082. **Rf** = 0.55 (hexane:Et_2O 60:40). **MP** = 68.7-70.1^{o}C.

3.4. Synthesis of allenes

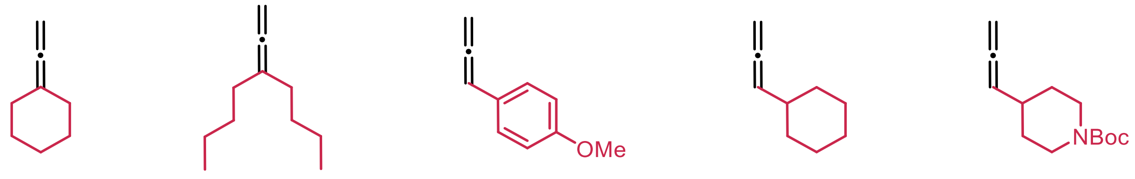

2a **2b** **2c** **2d** **2e**

Allene **2a** (vinylidenecyclohexane) was purchased from Sigma-Aldrich or prepared following a procedure reported in the literature.[6a] Allene **2d** (propa-1,2-dien-1-ylcyclohexane) were purchased from Sigma-Aldrich. Allene **2b** (5-vinylidenenonane),[6a] allene **2c** (1-methoxy-4-(propa-1,2-dien-1-yl)benzene),[6b] and allene **2e** (tert-butyl 4-(propa-1,2-dien-1-yl)piperidine-1-carboxylate),[6c] were prepared according to procedures described in the literature. Spectral data recorded agreed with the previously reported.

4. Desymmetrization and kinetic resolution of homobenzyltriflamides through an enantioselective Pd-catalyzed (5+2) annulation with allenes

4.1. Chiral ligand screening

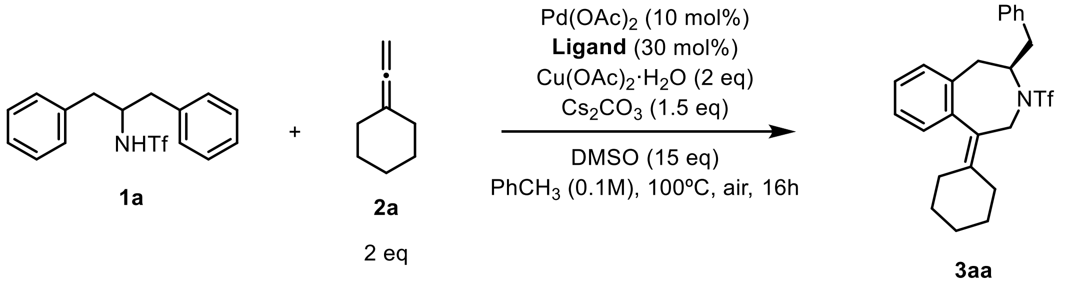

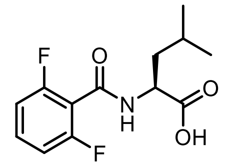

2,6-F,F-Bz-Leu-OH
72% yield
14% *ee*

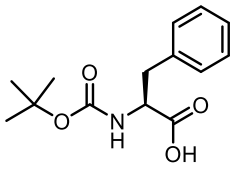

Boc-Phe-OH
88% yield
52% *ee*

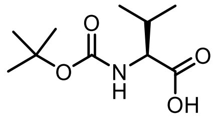

Boc-Val-OH
85% yield
76% *ee*

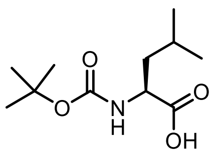

Boc-Leu-OH
86% yield
60% *ee*

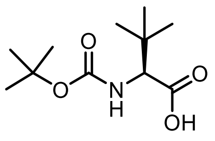

Boc-tLe-OH
67% yield
74% *ee*

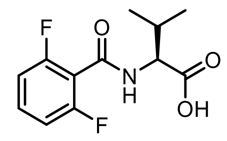

2,6-F,F-Bz-Val-OH
85% yield
6% *ee*

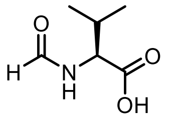

Formyl-Val-OH
23% yield
60% *ee*

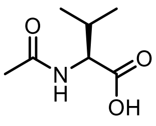

Ac-Val-OH
78% yield
42% *ee*

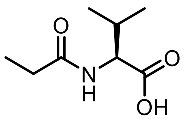

Pr-Val-OH
80% yield
42% *ee*

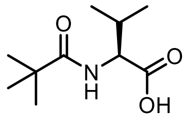

Piv-Val-OH
26% yield
4% *ee*

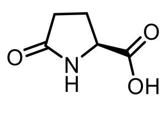

L-pGlu-OH
32% yield
72% *ee*

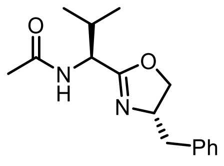

APAO Ligand
34% yield
46% *ee*

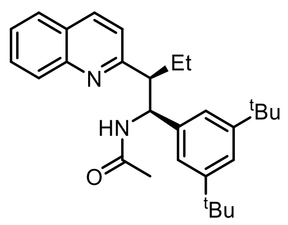

APAQ Ligand
41% yield
12% *ee*

Table S1. Screening of MPAA, APAO and APAQ chiral ligands

Reaction conditions: **1a** (0.1 mmol), **2a** (0.2 mmol), $Pd(OAc)_2$ (10 mol%), ligand (30 mol%), $Cu(OAc)_2 \cdot H_2O$ (2 eq), Cs_2CO_3 (1.5 eq), DMSO (15 eq), $PhCH_3$ (1 mL), air, 100°C, 16h. Isolated yields. Enantiomeric excess (*ee*) was determined by chiral HPLC analysis of the isolated pure product.

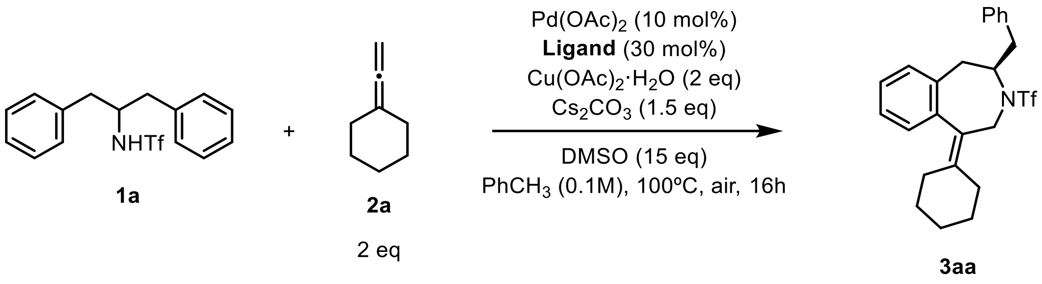

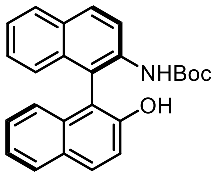

L1
31% yield
40% *ee*

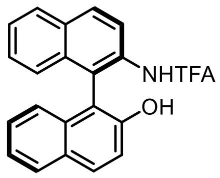

L2
28% yield
86% *ee*

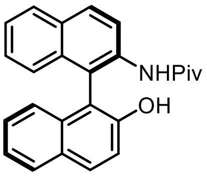

L3
52% yield
8% *ee*

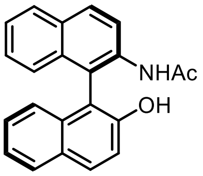

L4
92% yield
94% *ee*

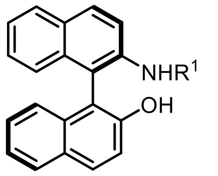

L5, (R^1 = (2,6-F,F)Bz)
81% yield
96% *ee*

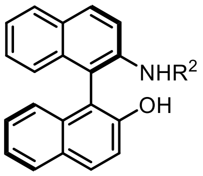

L6, (R^2 = (3,5-Me,Me)Bz)
54% yield
68% *ee*

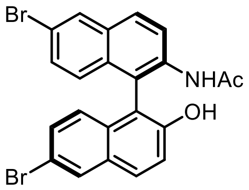

L7
55% yield
94% *ee*

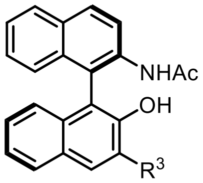

L8, (R^3 = CO_2Me)
27% yield
44% *ee*

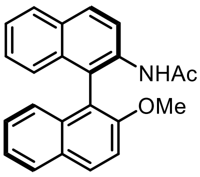

L9
30% yield
racemic

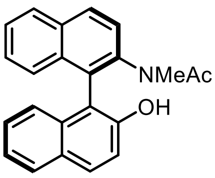

L10
55% yield
36% *ee*

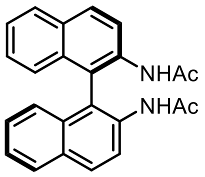

L11
36% yield
10% *ee*

No Ligand
40% yield
racemic

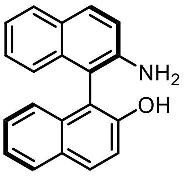

(*R*)-NOBIN
34% yield
42% *ee*

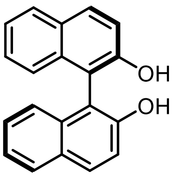

(*R*)-BINOL
14% yield
racemic

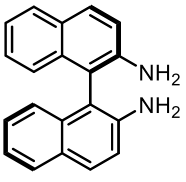

(*R*)-BINAM
20% yield
40% *ee*

Table S2. Screening of NOBIN-type chiral ligands

Reaction conditions: **1a** (0.1 mmol), **2a** (0.2 mmol), $Pd(OAc)_2$ (10 mol%), ligand (30 mol%), $Cu(OAc)_2 \cdot H_2O$ (2 eq), Cs_2CO_3 (1.5 eq), DMSO (15 eq), $PhCH_3$ (1 mL), air, 100°C, 16h. Isolated yields. Enantiomeric excess (*ee*) was determined by chiral HPLC analysis of the isolated pure product.

4.2. Chiral ligand loading

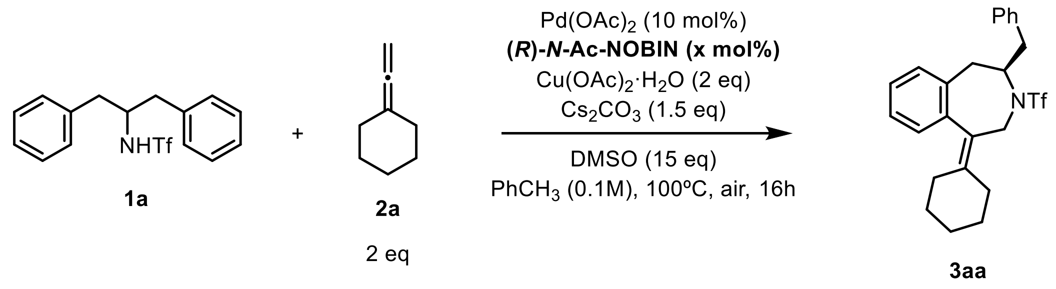

Entry	mol% Ligand	Yield (%)	*ee* (%)
**1**	10	66	88
**2**	15	72	90
**3**	20	80	90
**4**	30	92	94

Table S3. Screening chiral ligand loading

Reaction conditions: **1a** (0.1 mmol), **2a** (0.2 mmol), $Pd(OAc)_2$ (10 mol%), (*R*)-*N*-Ac-NOBIN (x mol%), $Cu(OAc)_2 \cdot H_2O$ (2 eq), Cs_2CO_3 (1.5 eq), DMSO (15 eq), $PhCH_3$ (1 mL), air, 100°C, 16h. Isolated yields. Enantiomeric excess (*ee*) was determined by chiral HPLC analysis of the isolated pure product.

4.3. Comparison of the reaction with and without ligand

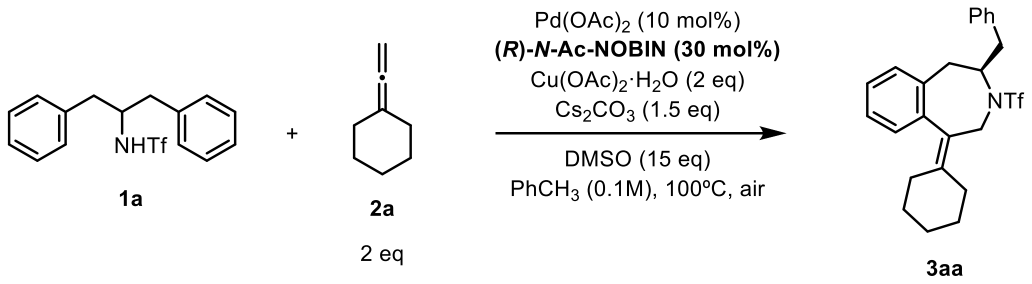

Two reactions were set using two separated Schlenk tubes under air atmosphere. Homobenzyltriflamide **1a** (0.2 mmol, 1 eq), $Pd(OAc)_2$ (4.5 mg, 10 mol%), $Cu(OAc)_2 \cdot H_2O$ (80 mg, 2 eq) and Cs_2CO_3 (97.8 mg, 1.5 eq) were added in each Schlenk. Then, in the first Schlenk, ligand **L4** ((*R*)-*N*-Ac-NOBIN) (19.6 mg, 30 mol%) was added, and in the second Schlenk no ligand was added. Then, toluene (2 mL, 0.1M), DMSO (213 µL, 15 eq) and allene **2a** (43.3 mg, 0.4 mmol, 2 eq) were added in both tubes. The tubes were sealed with a rubber septum and the reaction mixtures were stirred at 100°C. Aliquots of 250 µL of the reactions were taken at 5, 10, 20 and 40 min, filtered through a pad of silica gel and florisil eluting with Et_2O. The volatiles were evaporated, and the crude residues were analysed by **^{19}F NMR** spectrometry. The fractional conversion of product (int.P/(int.P+int.SM)) was plotted as a function of time.

Time (min)	Fractional Conversion (^{19}F-NMR) with ligand L4	Fractional Conversion (^{19}F-NMR) without ligand
**5**	0,1099	0,0490
**10**	0,1302	0,0608
**20**	0,1912	0,0944
**40**	0,2439	0,1205

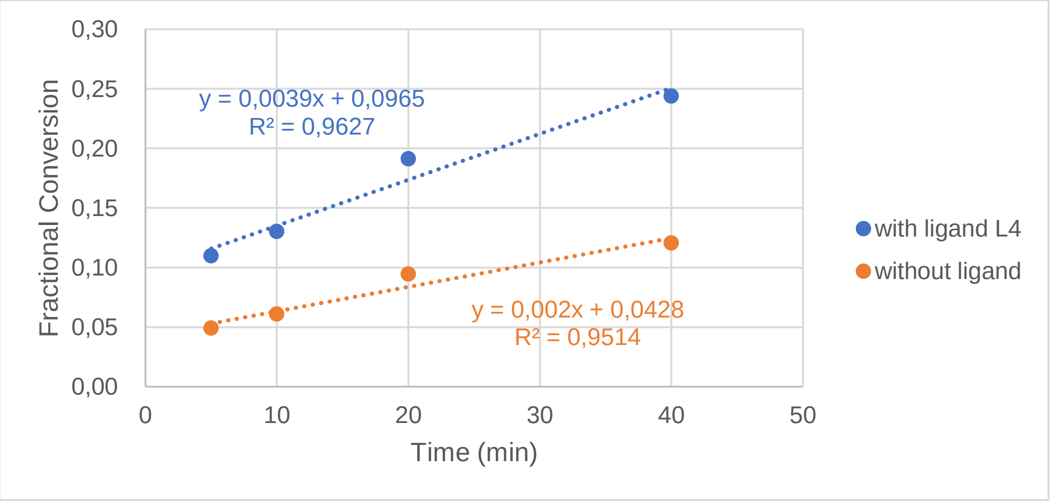

4.4. Base Screening

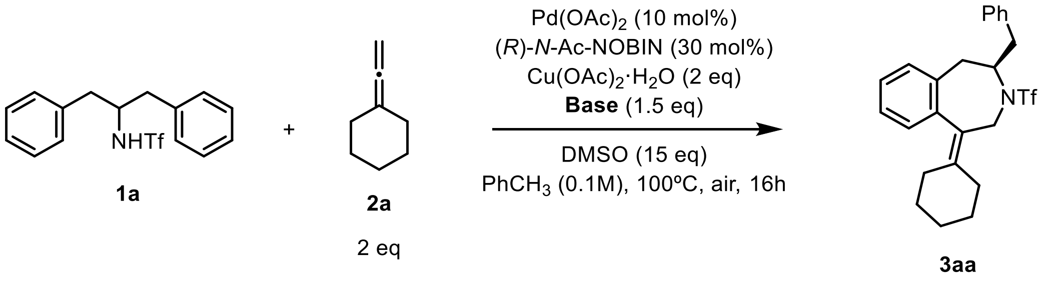

Entry	Base	Yield (%)	*ee* (%)
**1**	Cs_2CO_3	92	94
**2**	K_2CO_3	82	86
**3**	Na_2CO_3	63	70
**4**	Li_2CO_3	38	6

Table S3. Base Screening

Reaction conditions: **1a** (0.1 mmol), **2a** (0.2 mmol), $Pd(OAc)_2$ (10 mol%), (*R*)-*N*-Ac-NOBIN (30 mol%), $Cu(OAc)_2{\cdot}H_2O$ (2 eq), Base (1.5 eq), DMSO (15 eq), $PhCH_3$ (1 mL), air, 100°C, 16h. Isolated yields. Enantiomeric excess (*ee*) was determined by chiral HPLC analysis of the isolated pure product.

4.5. Desymmetrization of homobenzyltriflamides; reaction scope

General procedure for the desymmetrizing (5+2) annulation via Pd(II)-catalyzed C(sp^2)–H functionalization with allenes

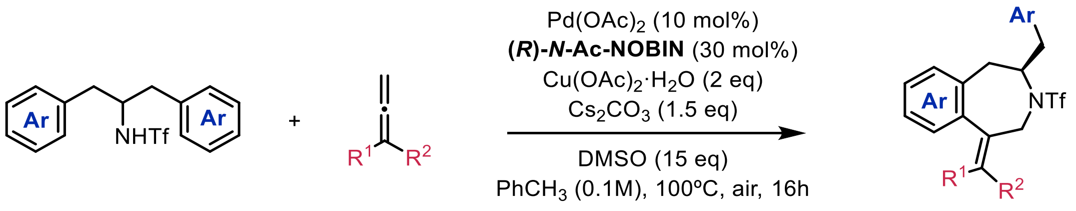

$Pd(OAc)_2$ (2.2 mg, 10 mol%), ligand (*R*)-*N*-Ac-NOBIN (9.8 mg, 30 mol%), $Cu(OAc)_2{\cdot}H_2O$ (40 mg, 2 eq), Cs_2CO_3 (48.9 mg, 1.5 eq) and the corresponding homobenzyltriflamide (0.1 mmol, 1 eq) were weighed in air and placed in a Schlenk tube with a magnetic stir bar. Then, toluene (1 mL, 0.1M), DMSO (15 eq) and the corresponding allene (0.2 mmol, 2 eq) were added. The tube was sealed with a rubber septum and the reaction mixture was stirred 16 hours at 100ºC. Then, the resulting mixture was cooled to room temperature and filtered through a pad of silica gel and florisil, eluting with Et_2O. Evaporation and column chromatography (silica gel, hexane:Et_2O 99:1 to 90:10) afforded the desired products.

(*R*)-4-benzyl-1-cyclohexylidene-3-((trifluoromethyl)sulfonyl)-2,3,4,5-tetrahydro-1*H*-benzo[*d*]azepine (3aa)

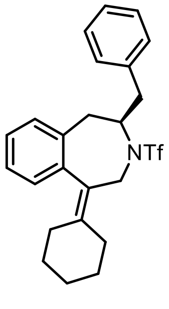

3aa

3aa was obtained as a white solid (41.5 mg, 92%). **^{1}H NMR** (500 MHz, $CDCl_3$) δ, ppm: 7.31 – 7.01 (m, 8H), 6.93 (d, *J* = 7.2 Hz, 1H), 4.48 (d, *J* = 16.3 Hz, 1H), 4.25 – 4.02 (m, 2H), 3.14 – 3.00 (m, 1H), 2.91 (t, *J* = 11.6 Hz, 1H), 2.69 – 2.48 (m, 2H), 2.22 – 2.08 (m, 3H), 2.03 – 1.94 (m, 1H), 1.67 – 1.31 (m, 6H). **^{19}F NMR** (470 MHz, $CDCl_3$) δ, ppm: -77.45 (s). **^{13}C NMR** (126 MHz, $CDCl_3$) δ, ppm: 145.2 (C), 142.7 (C), 138.0 (C), 136.9 (C), 136.1 (C), 130.9 (CH), 129.5 (CH), 128.9 (CH), 128.1 (CH), 127.7 (CH), 127.2 (CH), 126.9 (CH), 120.2 (q, *J* = 324.8 Hz, C), 61.0 (CH_2), 57.3 (CH), 45.7 (CH_2), 37.2 (CH_2), 32.5 (CH_2), 30.2 (CH_2), 28.6 (CH_2), 27.9 (CH_2), 26.7 (CH_2). **HRMS** (APCI, $[M+H]^+$) *m/z* calculated for $C_{24}H_{27}F_3NO_2S$: 450.1709; found 450.1708. **IR** (cm^{-1}): 2927, 2852, 1449, 1394, 1379, 1224, 1179, 1146, 1130, 767, 737, 703, 581. **Rf** = 0.68 (hexane:Et_2O 80:20). **MP** = 105.6-107.2°C.

Enantioselectivity of the cycloadduct product was determined by chiral SFC analysis on Phenomenex i-Cellulose-5 at 40°C (CO_2 : MeOH = 97:3, 1 mL/min, λ=210 nm).

Racemic sample (*rac*-**3aa**)

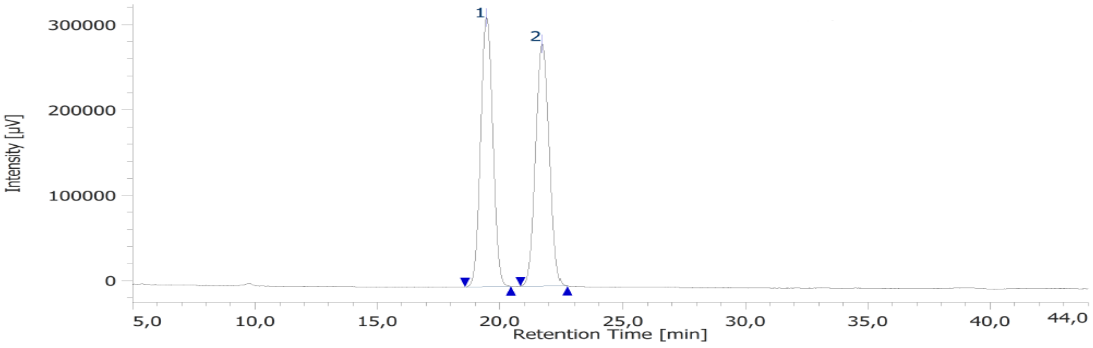

#	Peak Name	CH	tR [min]	Area [µV·sec]	Height [µV]	Area%	Height%	Quantity	NTP	Resolution	Symmetry Factor	Warning
1	Unknown	5	19,460	10947631	315393	49,978	52,618	N/A	7038	2,317	1,039	
2	Unknown	5	21,730	10957222	284004	50,022	47,382	N/A	7029	N/A	1,041	

Asymmetric sample (**3aa**, 96.8:3.2 *e.r.*, 94% *ee*)

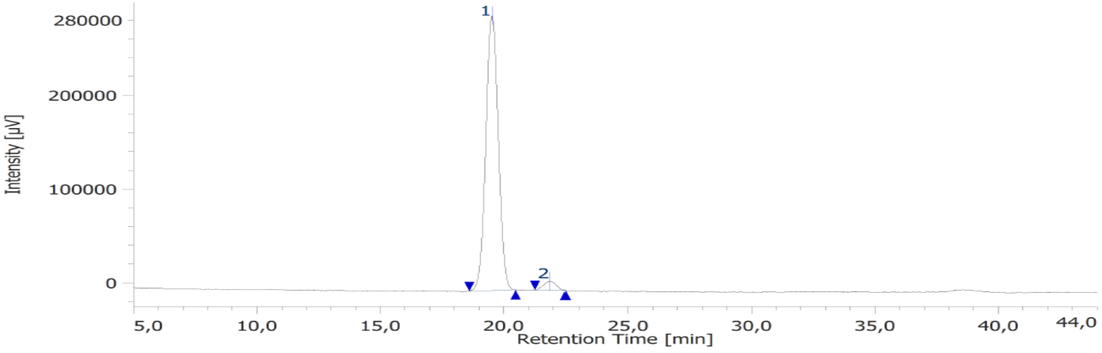

#	Peak Name	CH	tR [min]	Area [µV·sec]	Height [µV]	Area%	Height%	Quantity	NTP	Resolution	Symmetry Factor	Warning
1	Unknown	5	19,517	10317497	292684	96,810	96,842	N/A	6961	2,470	1,024	
2	Unknown	5	21,867	340011	9543	3,190	3,158	N/A	8097	N/A	1,011	

(*R*)-1-cyclohexylidene-6-fluoro-4-(2-fluorobenzyl)-3-((trifluoromethyl)sulfonyl)-2,3,4,5-tetrahydro-1*H*-benzo[*d*]azepine (3ba)

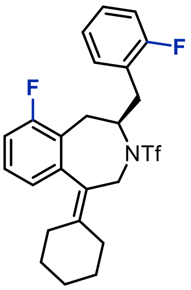

3ba

3ba was obtained as a white solid (42.8 mg, 88%). **^{1}H NMR** (300 MHz, $CDCl_3$) δ, ppm: 7.26 – 7.16 (m, 2H), 7.16 – 6.97 (m, 3H), 6.92 – 6.81 (m, 2H), 4.47 (d, *J* = 17.7 Hz, 1H), 4.32 – 4.04 (m, 2H), 3.17 – 2.93 (m, 3H), 2.40 (t, *J* = 12.8 Hz, 1H), 2.24 – 2.05 (m, 2H), 2.04 – 1.92 (m, 1H), 1.67 – 1.43 (m, 6H). **^{19}F NMR** (470 MHz, $CDCl_3$) δ, ppm: -77.53 (s), -177.49 (s), -121.47 (s). **^{13}C NMR** (126 MHz, $CDCl_3$) δ, ppm: 161.7 (d, *J* = 191.0 Hz, C), 159.7 (d, *J* = 190.8 Hz, C) 146.3 (C), 145.2 (C), 140.6 (C), 139.3 (C), 131.7 (CH), 129.2 (d, J = 8.2 Hz, CH), 127.9 (d, *J* = 8.7 Hz, CH), 126.5 (d, *J* = 2.5 Hz, CH), 124.6 (d, *J* = 3.5 Hz, CH), 123.6 (d, *J* = 15.9 Hz, C), 123.0 (d, *J* = 17.0 Hz, C), 119.3 (q, *J* = 321.6 Hz, C), 115.7 (d, *J* = 22.3 Hz, CH), 114.1 (d, *J* = 22.6 Hz, CH), 59.0 (CH), 56.3 (CH_2), 45.5 (CH_2), 32.6 (CH_2), 30.2 (CH_2), 28.5 (CH_2), 27.9 (CH_2), 26.6 (CH_2). **HRMS** (APCI, $[M+H]^+$) *m/z* calculated for $C_{24}H_{25}F_5NO_2S$: 486.1521; found 486.1537. **Rf** = 0.60 (hexane:Et_2O 80:20). **MP** = 44.6-46.1^{o}C.

Enantioselectivity of the cycloadduct product was determined by chiral SFC analysis on Phenomenex Cellulose-1 at 40°C (CO_2 : MeOH = 97:3, 1 mL/min, λ=210 nm).

Racemic sample (*rac*-**3ba**)

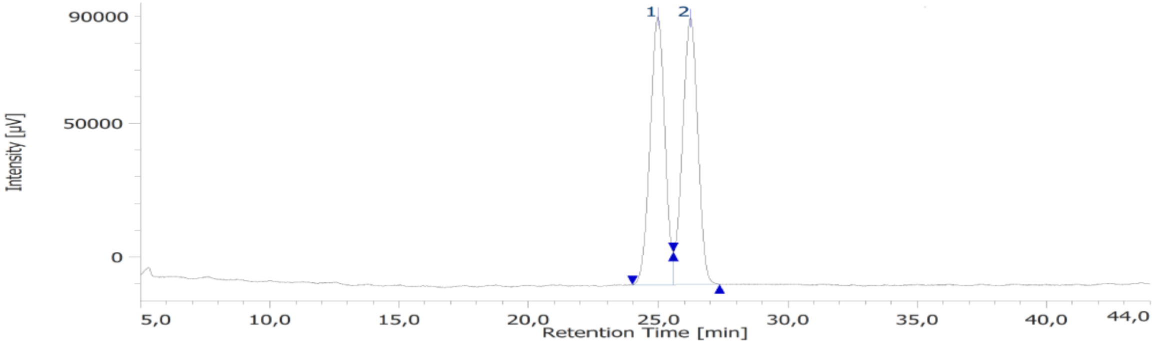

#	Peak Name	CH	tR [min]	Area [µV·sec]	Height [µV]	Area%	Height%	Quantity	NTP	Resolution	Symmetry Factor	Warning
1	Unknown	5	24.957	3991052	100263	49.303	50.178	N/A	8856	1.172	N/A	
2	Unknown	5	26.220	4103945	99552	50.697	49.822	N/A	9086	N/A	N/A	

Asymmetric sample (**3ba**, 98.6:1.4 *e.r.*, 97% *ee*)

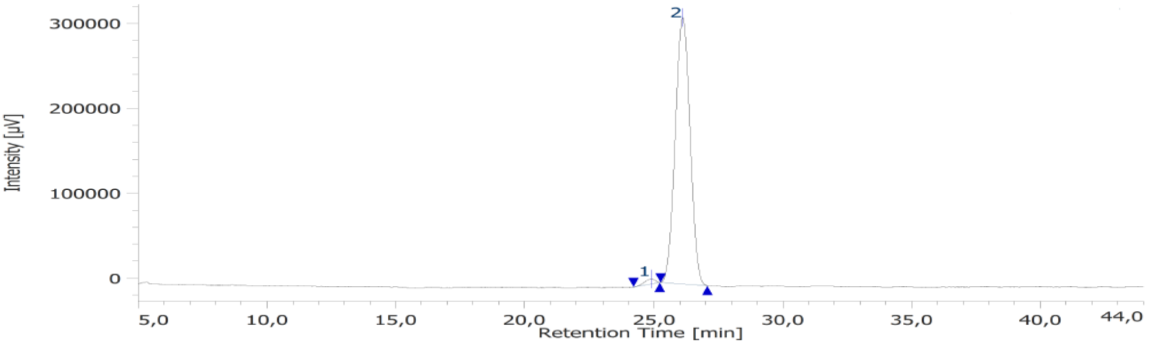

#	Peak Name	CH	tR [min]	Area [µV·sec]	Height [µV]	Area%	Height%	Quantity	NTP	Resolution	Symmetry Factor	Warning
1	Unknown	5	24.877	177822	6061	1.394	1.897	N/A	14626	1.289	0.786	
2	Unknown	5	26.100	12576969	313394	98.606	98.103	N/A	9331	N/A	1.004	

(*R*)-6-chloro-4-(2-chlorobenzyl)-1-cyclohexylidene-3-((trifluoromethyl)sulfonyl)-2,3,4,5-tetrahydro-1*H*-benzo[*d*]azepine (3ca)

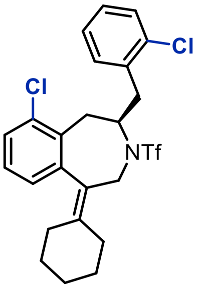

3ca

3ca was obtained as a white solid (47.8 mg, 92%). **^{1}H NMR** (500 MHz, $CDCl_3$) δ, ppm: 7.47 – 7.41 (m, 1H), 7.37 – 7.23 (m, 4H), 7.17 (t, *J* = 7.8 Hz, 1H), 7.05 (d, *J* = 7.5 Hz, 1H), 4.58 (d, *J* = 17.8 Hz, 1H), 4.47 – 4.32 (m, 2H), 3.39 – 3.16 (m, 3H), 2.67 (t, *J* = 12.9 Hz, 1H), 2.36 – 2.14 (m, 3H), 2.14 – 2.03 (m, 1H), 1.79 – 1.46 (m, 6H). **^{19}F NMR** (470 MHz, $CDCl_3$) δ, ppm: -77.44 (s). **^{13}C NMR** (126 MHz, $CDCl_3$) δ, ppm: 146.2 (C), 144.8 (C), 140.2 (C), 138.9 (C), 134.6 (C), 134.5 (C), 133.0 (C), 131.5 (CH), 130.0 (CH), 129.5 (CH), 128.8 (CH), 128.3 (CH), 127.8 (CH), 127.3 (CH), 119.2 (q, *J* = 319.6 Hz, C), 66.0 (CH_2), 64.3 (CH), 45.6 (CH_2), 33.6 (CH_2), 32.6 (CH_2), 30.2 (CH_2), 28.5 (CH_2), 27.9 (CH_2), 26.6 (CH_2). **HRMS** (APCI, $[M+H]^+$) *m/z* calculated for $C_{24}H_{25}Cl_2F_3NO_2S$: 518.0930; found 518.0917. **Rf** = 0.59 (hexane:Et_2O 80:20). **MP** = 115.6-117.1^{o}C.

Enantioselectivity of the cycloadduct product was determined by chiral SFC analysis on Phenomenex Cellulose-1 at 40°C (CO_2 : MeOH = 96:4, 1 mL/min, λ=220 nm).

Racemic sample (*rac*-**3ca**)

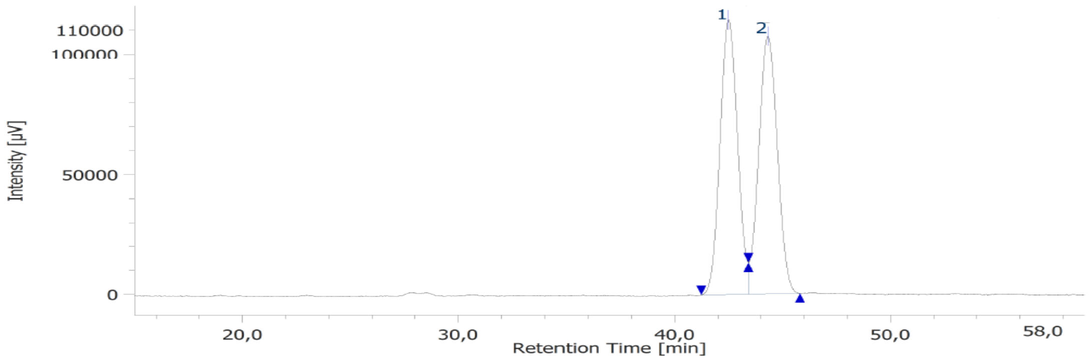

#	Peak Name	CH	tR [min]	Area [µV·sec]	Height [µV]	Area%	Height%	Quantity	NTP	Resolution	Symmetry Factor	Warning
1	Unknown	11	42,487	6459286	114123	50,070	51,553	N/A	12541	1,166	N/A	
2	Unknown	11	44,307	6441335	107249	49,930	48,447	N/A	12093	N/A	N/A	

Asymmetric sample (**3ca**, 97.7:2.3 *e.r.*, 95% *ee*)

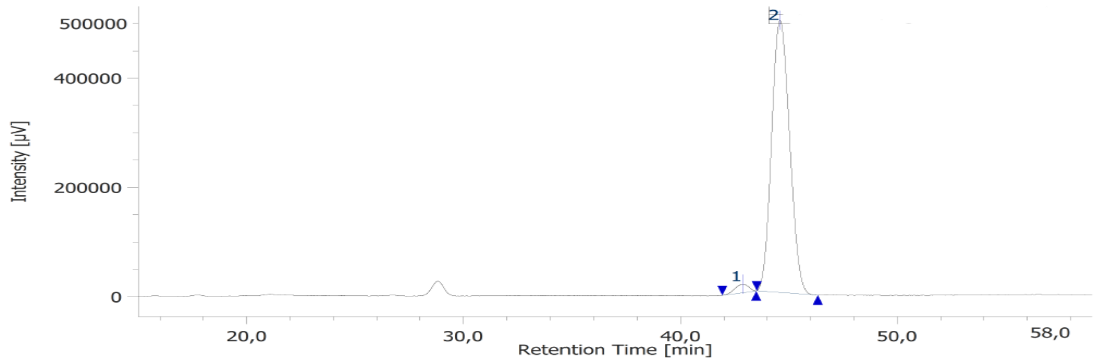

#	Peak Name	CH	tR [min]	Area [µV·sec]	Height [µV]	Area%	Height%	Quantity	NTP	Resolution	Symmetry Factor	Warning
1	Unknown	10	42,853	693115	15458	2,345	3,010	N/A	18501	1,225	0,859	
2	Unknown	10	44,577	28869210	498046	97,655	96,990	N/A	13041	N/A	1,114	

(*R*)-7-bromo-4-(3-bromobenzyl)-1-cyclohexylidene-3-((trifluoromethyl)sulfonyl)-2,3,4,5-tetrahydro-1*H*-benzo[*d*]azepine (3da)

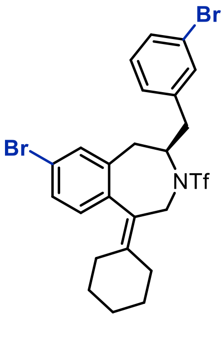

3da

3da was obtained as a white solid (49.8 mg, 82%). **^{1}H NMR** (500 MHz, $CDCl_3$) δ, ppm: 7.47 – 7.26 (m, 3H), 7.22 – 7.09 (m, 3H), 1.68 – 1.45 (m, 1H), 4.62 – 4.27 (m, 1H), 4.22 – 3.97 (m, 2H), 3.06 (d, *J* = 13.5 Hz, 1H), 2.87 (d, *J* = 13.3 Hz, 1H), 2.65 – 2.49 (m, 2H), 2.23 – 2.15 (m, 2H), 2.15 – 2.06 (m, 1H), 2.01 – 1.92 (m, 1H), 1.68 – 1.45 (m, 6H). **^{19}F NMR** (470 MHz, $CDCl_3$) δ, ppm: -77.3 (s). **^{13}C NMR** (126 MHz, $CDCl_3$) δ, ppm: 138.9 (C), 138.9 (C), 136.9 (C), 134.5 (C), 132.5 (CH), 132.4 (CH), 131.7 (CH), 131.0 (CH), 130.6 (CH), 130.6 (CH), 128.1 (CH), 123.0 (C), 121.3 (C), 119.3 (q, *J* = 313.9 Hz, C), 66.0 (CH_2), 60.5 (CH), 45.4 (CH_2) , 32.5 (CH_2), 32.6 (CH_2), 30.3 (CH_2), 28.5 (CH_2), 27.8 (CH_2), 27.3 (CH_2), 26.6 (CH_2). **HRMS** (APCI, $[M+H]^+$) *m/z* calculated for $C_{24}H_{25}Br_2F_3NO_2S$: 605.9919; found 605.9919. **Rf** = 0.61 (hexane:Et_2O 80:20). **MP** = 60.6-63.1°C.

Enantioselectivity of the cycloadduct product was determined by chiral HPLC analysis on Chiralpak IB at rt (Hexane : *i*PrOH = 99:1, 0.5 mL/min, λ=254 nm).

Racemic sample (*rac*-**3da**)

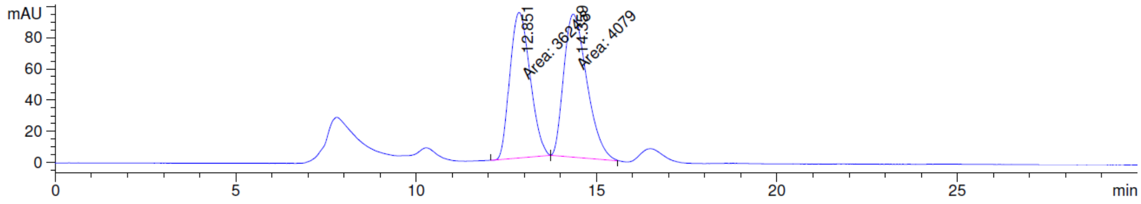

Peak #	RetTime [min]	Type	Width [min]	Area [mAU*s]	Height [mAU]	Area %
1	12.851	MM	0.6499	3624.80103	92.96255	47.0521
2	14.359	MM	0.7435	4078.99878	91.44033	52.9479

Asymmetric sample (**3da**, 98.0:2.0 *e.r.*, 96% *ee*)

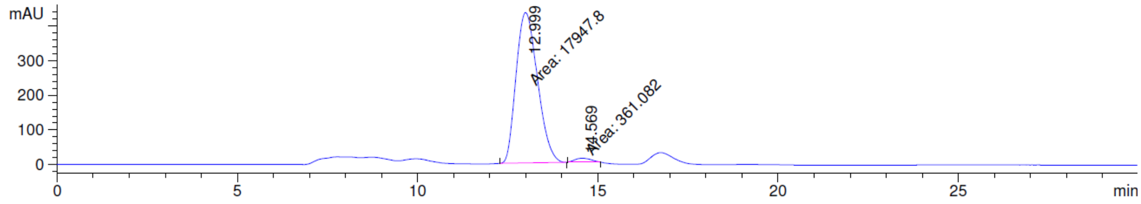

Peak #	RetTime [min]	Type	Width [min]	Area [mAU*s]	Height [mAU]	Area %
1	12.999	MM	0.6903	1.79478e4	433.34088	98.0278
2	14.569	MM	0.5208	361.08160	11.55587	1.9722

(*R*)-8-bromo-4-(4-bromobenzyl)-1-cyclohexylidene-3-((trifluoromethyl)sulfonyl)-2,3,4,5-tetrahydro-1*H*-benzo[*d*]azepine (3ea)

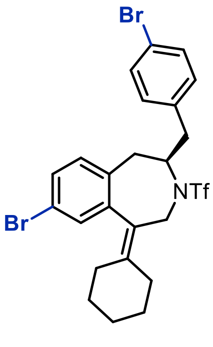

3ea

3ea was obtained as a white solid (46.2 mg, 76%). **^{1}H NMR** (300 MHz, $CDCl_3$) δ, ppm: 7.41 (d, *J* = 8.0 Hz, 2H), 7.25 (dd, *J* = 8.1, 2.1 Hz, 1H), 7.22 – 7.16 (m, 1H), 7.05 (d, *J* = 8.0 Hz, 2H), 6.82 (d, *J* = 8.0 Hz, 1H), 4.58 – 4.37 (m, 1H), 4.13 (d, *J* = 17.7 Hz, 1H), 4.06 – 3.94 (m, 1H), 3.11 – 2.95 (m, 1H), 2.86 (t, *J* = 11.6 Hz, 1H), 2.60 – 2.46 (m, 2H), 2.23 – 2.07 (m, 3H), 2.06 – 1.93 (m, 1H), 1.71 – 1.44 (m, 6H). **^{19}F NMR** (282 MHz, $CDCl_3$) δ, ppm: -77.34 (s). **^{13}C NMR** (126 MHz, $CDCl_3$) δ, ppm: 146.7 (C), 144.7 (C), 140.0 (C), 135.6 (C), 134.8 (C), 133.5 (CH), 132.2 (CH), 131.1 (CH), 130.7 (CH), 129.7 (CH), 121.3 (C), 120.6 (C), 119.2 (q, *J* = 325.7 Hz, C), 66.0 (CH_2), 60.6 (CH), 45.5 (CH_2) , 33.6 (CH_2), 32.5 (CH_2), 30.2 (CH_2), 28.5 (CH_2), 27.9 (CH_2), 26.6 (CH_2). **HRMS** (APCI, $[M+H]^+$) *m/z* calculated for $C_{24}H_{25}Br_2F_3NO_2S$: 605.9919; found 605.9926. **Rf** = 0.59 (hexane:Et_2O 80:20). **MP** = 55.3-57.1^{o}C.

Enantioselectivity of the cycloadduct product was determined by chiral SFC analysis on Phenomenex Cellulose-1 at 40°C (CO_2 : MeOH = 92:8, 1 mL/min, λ=254 nm).

Racemic sample (*rac*-**3ea**)

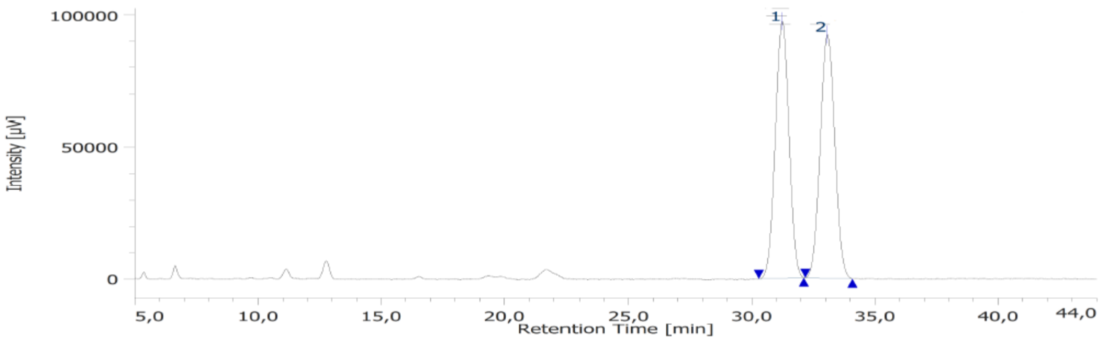

#	Peak Name	CH	tR [min]	Area [µV·sec]	Height [µV]	Area%	Height%	Quantity	NTP	Resolution	Symmetry Factor	Warning
1	Unknown	10	31,227	3756980	97035	49,742	51,311	N/A	14556	1,725	1,000	
2	Unknown	10	33,063	3795911	92075	50,258	48,689	N/A	14470	N/A	1,027	

Asymmetric sample (**3ea**, 97.0:3.0 *e.r.*, 94% *ee*)

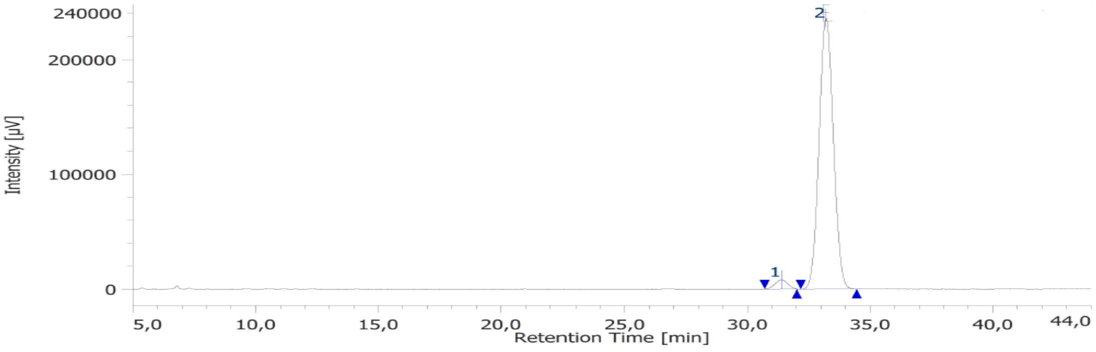

#	Peak Name	CH	tR [min]	Area [µV·sec]	Height [µV]	Area%	Height%	Quantity	NTP	Resolution	Symmetry Factor	Warning
1	Unknown	10	31,377	290069	8112	2,952	3,332	N/A	16583	1,777	0,982	
2	Unknown	10	33,197	9536710	235327	97,048	96,668	N/A	15136	N/A	1,047	

(*R*)-1-cyclohexylidene-8-(trifluoromethyl)-4-(4-(trifluoromethyl)benzyl)-3-((trifluoromethyl)sulfonyl)-2,3,4,5-tetrahydro-1*H*-benzo[*d*]azepine (3fa)

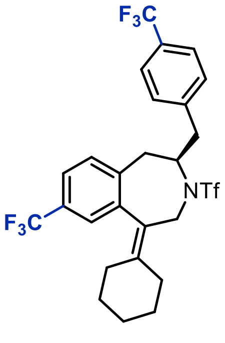

3fa

3fa was obtained as a white solid (48.5 mg, 83%). **^{1}H NMR** (500 MHz, $CDCl_3$) δ, ppm: 7.63 (d, *J* = 7.9 Hz, 2H), 7.46 (d, *J* = 7.9 Hz, 1H), 7.39 (d, *J* = 8.4 Hz, 3H), 7.15 (d, *J* = 7.9 Hz, 1H), 4.73 – 4.51 (m, 1H), 4.31 – 4.05 (m, 2H), 3.28 – 3.14 (m, 1H), 3.08 (t, *J* = 11.4 Hz, 1H), 2.81 – 2.72 (m, 1H), 2.82 – 2.72 (m, 1H), 2.72 – 2.65 (m, 1H), 2.31 – 2.23 (m, 2H), 2.20 – 2.12 (m, 1H), 2.08 – 2.00 (m, 1H), 1.77 – 1.69 (m, 2H), 1.68 – 1.56 (m, 4H). **^{19}F NMR** (282 MHz, $CDCl_3$) δ, ppm: -62.46 (s), -62.54 (s), -77.46 (s). **^{13}C NMR** (126 MHz, $CDCl_3$) δ, ppm: 143.4 (C), 140.6 (C), 140.2 (C), 139.6 (C), 138.7 (C), 129.9 (q, *J* = 32.5 Hz, C), 129.9 (CH), 129.6 (q, *J* = 32.4 Hz, C) 128.6, (CH), 127.8 (d, *J* = 3.4 Hz, CH), 126.0 (d, *J* = 3.6 Hz, CH), 124.6 (d, *J* = 3.5 Hz, CH), 124.2 (q, *J* = 272.0 Hz, C) 120.1 (q, *J* = 319.5 Hz, C), 119.1 (q, *J* = 328.6 Hz, C), 60.4 (CH_2), 57.0 (CH), 45.5 (CH_2), 37.0 (CH_2), 32.5 (CH_2), 30.5 (CH_2), 30.3 (CH_2), 28.5 (CH_2), 27.9 (CH_2), 26.5 (CH_2). **HRMS** (APCI, $[M+H]^+$) *m/z* calculated for $C_{26}H_{23}F_9NO_2S$: 584.1300; found 584.1297. **Rf** = 0.51 (hexane:Et_2O 70:30). **MP** = 69.1-70.6^{o}C.
Enantioselectivity of the cycloadduct product was determined by chiral SFC analysis on Phenomenex Cellulose-2 at 40°C (CO_2 : MeOH = 97:3, 1 mL/min, λ=254 nm).

Racemic sample (*rac*-**3fa**)

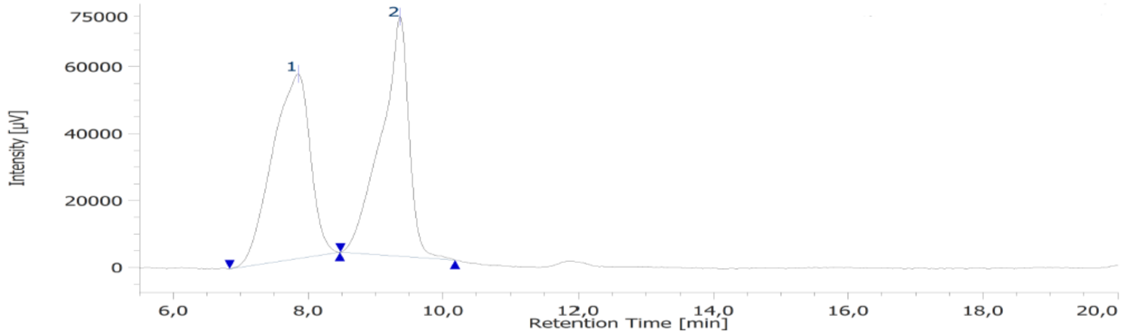

#	Peak Name	CH	tR [min]	Area [µV·sec]	Height [µV]	Area%	Height%	Quantity	NTP	Resolution	Symmetry Factor	Warning
1	Unknown	11	7.850	2159682	55200	51.219	43.545	N/A	853	1.699	0.791	
2	Unknown	11	9.367	2056855	71564	48.781	56.455	N/A	2750	N/A	0.727	

Asymmetric sample (**3fa**, 99.0:1.0 *e.r.*, 98% *ee*)

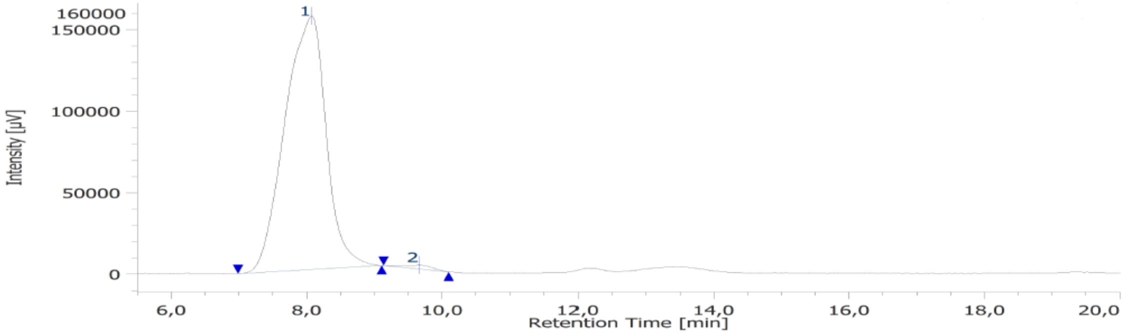

#	Peak Name	CH	tR [min]	Area [µV·sec]	Height [µV]	Area%	Height%	Quantity	NTP	Resolution	Symmetry Factor	Warning
1	Unknown	11	8.073	6584571	155425	98.953	98.445	N/A	806	1.692	0.886	
2	Unknown	11	9.663	69672	2455	1.047	1.555	N/A	2680	N/A	0.886	

(*R*)-1-cyclohexylidene-8-methoxy-4-(4-methoxybenzyl)-3-((trifluoromethyl)sulfonyl)-2,3,4,5-tetrahydro-1*H*-benzo[*d*]azepine (3ga)

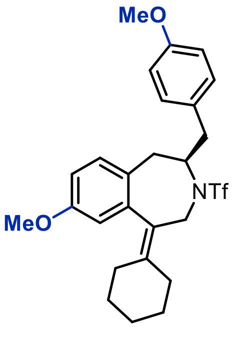

3ga

3ga was obtained as a yellowish solid (33.5 mg, 66%, using **L4** as ligand; 25.6 mg, 50%, using **L5** as ligand). **^{1}H NMR** (500 MHz, $CDCl_3$) δ, ppm: 7.16 (d, *J* = 8.0 Hz, 2H), 6.96 – 6.81 (m, 3H), 6.77 – 6.61 (m, 2H), 4.63 – 4.46 (m, 1H), 4.20 (d, *J* = 17.7 Hz, 1H), 4.14 – 4.01 (m, 1H), 3.82 (s, 3H), 3.77 (s, 3H), 3.06 (t, *J* = 11.8 Hz, 1H), 2.91 (t, *J* = 11.7 Hz, 1H), 2.69 – 2.53 (m, 2H2.29 – 2.18 (m, 3H), 2.15 – 2.03 (m, 1H), 1.72 – 1.54 (m, 6H). **^{19}F NMR** (470 MHz, $CDCl_3$) δ, ppm: -77.40 (s). **^{13}C NMR** (126 MHz, $CDCl_3$) δ, ppm: 158.8 (C), 158.4 (C), 145.3 (C), 143.8 (C), 139.1 (C), 138.2 (C), 132.8 (C), 130.5 (CH), 128.9 (CH), 119.5 (q, *J* = 311.3 Hz, C), 116.7 (CH), 114.3 (CH), 112.8 (CH), 60.9 (CH_2), 58,1 (CH) 55.4 (CH_3), 45.6 (CH_2), 36.3 (CH_2), 32.5 (CH_2), 30.2 (CH_2), 28.6 (CH_2), 27.9 (CH_2), 26.7 (CH_2). **HRMS** (APCI, $[M+H]^+$) *m/z* calculated for $C_{26}H_{31}F_3NO_4S$: 510.1920; found 510.1919. **Rf** = 0.49 (hexane:Et_2O 70:30). **MP** = 97.1-98.6^{o}C.

Enantioselectivity of the cycloadduct product was determined by chiral HPLC analysis on Chiralpak IF3 at rt (Hexane : *i*PrOH = 99:1, 0.5 mL/min, λ=254 nm).

Racemic sample (*rac*-**3ga**)

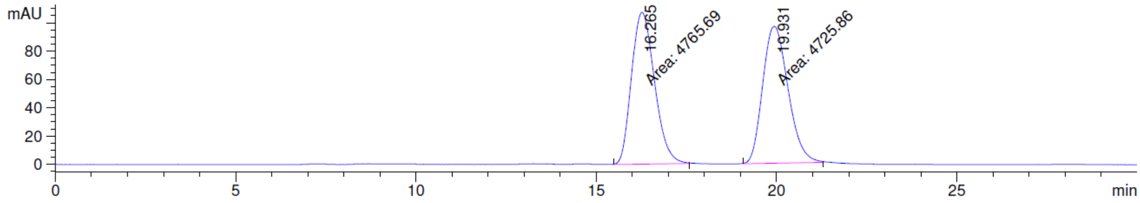

Peak #	RetTime [min]	Type	Width [min]	Area [mAU*s]	Height [mAU]	Area %
1	16.265	MM	0.7414	4765.68799	107.13641	50.2098
2	19.931	MM	0.8144	4725.85986	96.70866	49.7902

Asymmetric sample using ligand **L4** (**3ga**, 97.2:2.8 *e.r.*, 94% *ee*)

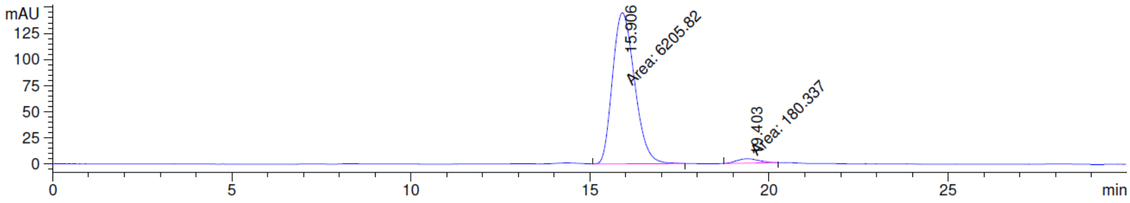

Peak #	RetTime [min]	Type	Width [min]	Area [mAU*s]	Height [mAU]	Area %
1	15.906	MM	0.7142	6205.81934	144.81584	97.1761
2	19.403	MM	0.7256	180.33653	4.14231	2.8239

Asymmetric sample using ligand **L5** (**3ga**, 97.0:3.0 *e.r.*, 94% *ee*)

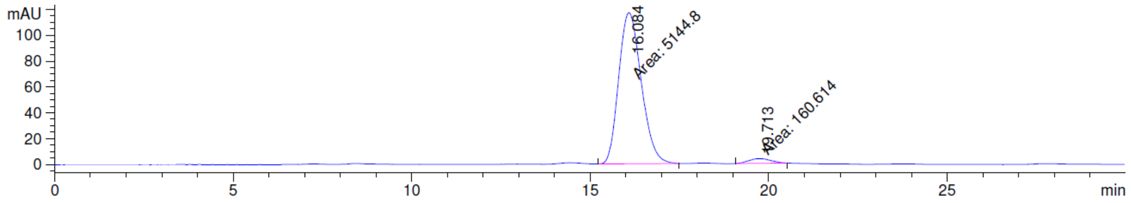

Peak #	RetTime [min]	Type	Width [min]	Area [mAU*s]	Height [mAU]	Area %
1	16.084	MM	0.7348	5144.79932	116.70015	96.9726
2	19.713	MM	0.7257	160.61375	3.68847	3.0274

(*R*)-1-cyclohexylidene-8-methyl-4-(4-methylbenzyl)-3-((trifluoromethyl)sulfonyl)-2,3,4,5-tetrahydro-1*H*-benzo[*d*]azepine (3ha)

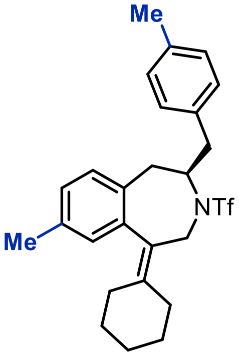

3ha

3ha was obtained as a white solid (41.9 mg, 88%). **^{1}H NMR** (500 MHz, $CDCl_3$) δ, ppm: 7.19 – 7.10 (m, 4H), 7.05 – 6.87 (m, 3H), 4.54 (d, *J* = 17.8 Hz, 1H), 4.22 (d, *J* = 17.6 Hz, 1H), 4.17 – 4.08 (m, 1H), 3.10 (t, *J* = 11.6 Hz, 1H), 2.95 (t, *J* = 10.8 Hz, 1H), 2.71 – 2.55 (m, 2H), 2.37 (s, 3H), 2.32 (s, 3H), 2.28 – 2.17 (m, 3H), 2.14 – 2.06 (m, 1H), 1.74 – 1.54 (m, 6H). **^{19}F NMR** (470 MHz, $CDCl_3$) δ, ppm: -77.42 (s). **^{13}C NMR** (126 MHz, $CDCl_3$) δ, ppm: 144.9 (C), 142.5 (C), 137.8 (C), 136.8 (C), 136.2 (C), 133.8 (C), 133.1 (C), 131.4 (CH), 129.6 (CH), 129.4 (CH), 128.3 (CH), 127.9 (CH), 120.2 (q, *J* =319.8 Hz, C), 60.9 (CH_2), 57.9 (CH), 45.7 (CH_2), 36.7 (CH_2), 32.5 (CH_2), 30.2 (CH_2), 28.6 (CH_2), 27.9 (CH_2), 26.7 (CH_2), 21.3 (CH_3), 21.2 (CH_3). **HRMS** (APCI, $[M+H]^+$) *m/z* calculated for $C_{26}H_{31}F_3NO_2S$: 478.2022; found 478.2025. **Rf** = 0.71 (hexane:Et_2O 80:20). **MP** = 85.2-86.9^{o}C.

Enantioselectivity of the cycloadduct product was determined by chiral SFC analysis on Phenomenex i-Cellulose-5 at 40°C (CO_2 : MeOH = 97:3, 1 mL/min, λ=220 nm).

Racemic sample (*rac*-**3ha**)

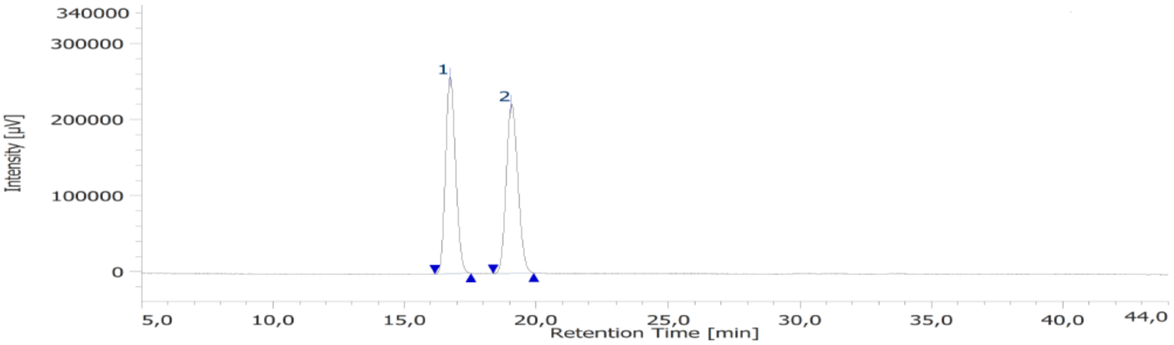

#	Peak Name	CH	tR [min]	Area [µV·sec]	Height [µV]	Area%	Height%	Quantity	NTP	Resolution	Symmetry Factor	Warning
1	Unknown	10	16,730	6869561	258309	50,041	53,759	N/A	8964	3,071	1,159	
2	Unknown	10	19,070	6858419	222188	49,959	46,241	N/A	8625	N/A	1,130	

Asymmetric sample (**3ha**, 95.9:4.1 *e.r.*, 92% *ee*)

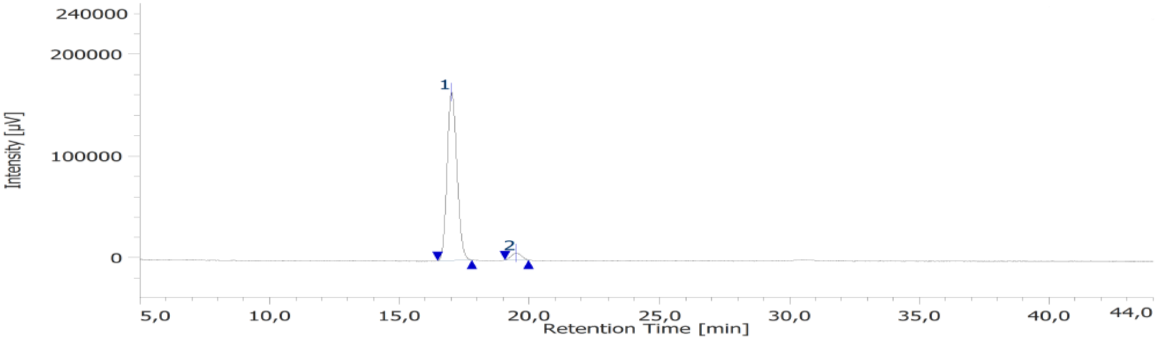

#	Peak Name	CH	tR [min]	Area [µV·sec]	Height [µV]	Area%	Height%	Quantity	NTP	Resolution	Symmetry Factor	Warning
1	Unknown	10	17,013	4354733	165242	95,933	96,037	N/A	9489	3,468	1,131	
2	Unknown	10	19,510	184607	6819	4,067	3,963	N/A	10967	N/A	1,009	

(*R*)-5-cyclohexylidene-2-(naphthalen-1-ylmethyl)-3-((trifluoromethyl)sulfonyl)-2,3,4,5-tetrahydro-1*H*-naphtho[1,2-*d*]azepine (3ia)

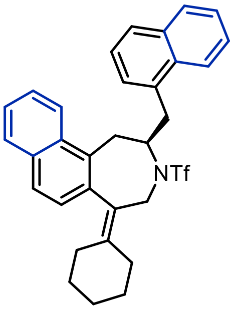

3ia

3ia was obtained as a white solid (50.1 mg, 92%). **^{1}H NMR** (500 MHz, $CDCl_3$) δ, ppm: 7.89 – 7.83 (m, 1H), 7.82 – 7.76 (m, 1H), 7.73 – 7.67 (m, 1H), 7.62 (d, *J* = 8.4 Hz, 1H), 7.53 – 7.45 (m, 2H), 7.43 – 7.39 (m, 2H), 7.27 (t, *J* = 7.4 Hz, 1H), 7.22 – 7.12 (m, 3H), 4.71 – 4.50 (m, 1H), 4.42 (d, *J* = 17.6 Hz, 2H), 3.49 – 3.41 (m, 1H), 3.22 (dd, *J* = 14.5, 5.3 Hz, 1H), 2.79 (dd, *J* = 14.5, 12.2 Hz, 1H), 2.38 – 2.22 (m, 1H), 2.22 – 2.14 (m, 1H), 2.09 – 1.97 (m, 1H), 1.73 – 1.50 (m, 6H), 1.47 – 1.42 (m, 1H), 1.40 – 1.35 (m, 1H), 1.26 – 1.11 (m, 1H). **^{19}F NMR** (470 MHz, $CDCl_3$) δ, ppm: -77.45 (s). **^{13}C NMR** (126 MHz, $CDCl_3$) δ, ppm: 138.4 (C), 135.6 (C), 134.8 (C), 134.7 (C), 134.2 (C), 133.1 (C), 133.1 (C), 132.3 (C), 130.9 (C), 129.0 (CH), 128.8 (CH), 128.6 (CH), 128.2 (CH), 128.0 (CH), 127.8 (CH), 126.8 (CH), 126.3 (CH), 126.0 (CH), 125.4 (CH), 125.3 (CH), 125.3 C(H), 122.0 (CH), 119.1 (q *J* = 336.6 Hz, C), 58.9 (CH), 45.1 (CH_2), 32.8 (CH_2), 31.7 (CH_2), 30.2 (CH_2), 28.5 (CH_2), 28.0 (CH_2), 26.7 (CH_2), 22.8 (CH_2). **HRMS** (APCI, $[M+H]^+$) *m/z* calculated for $C_{32}H_{31}F_3NO_2S$: 550.2022; found 550.2032. **Rf** = 0.57 (hexane:Et_2O 80:20). **MP** = 146.3-148.2^{o}C.

Enantioselectivity of the cycloadduct product was determined by chiral HPLC analysis on Chiralpak IB at rt (Hexane : *i*PrOH = 99:1, 0.5 mL/min, λ=254 nm).

Racemic sample (*rac*-**3ia**)

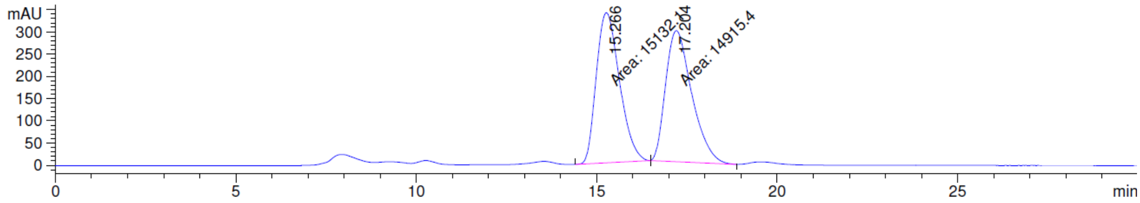

Peak #	RetTime [min]	Type	Width [min]	Area [mAU*s]	Height [mAU]	Area %
1	15.266	MM	0.7477	1.51321e4	337.31384	50.3607
2	17.204	MM	0.8444	1.49154e4	294.41104	49.6393

Asymmetric sample (**3ia**, 92.1:7.9 *e.r.*, 84% *ee*)

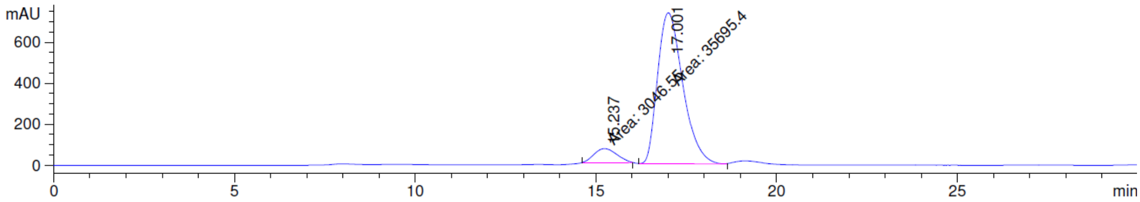

Peak #	RetTime [min]	Type	Width [min]	Area [mAU*s]	Height [mAU]	Area %
1	15.237	MM	0.7339	3046.54834	69.18185	7.8637
2	17.001	MM	0.8090	3.56954e4	735.37329	92.1363

(*R*)-4-benzyl-1-(nonan-5-ylidene)-3-((trifluoromethyl)sulfonyl)-2,3,4,5-tetrahydro-1*H*-benzo[*d*]azepine (3ab)

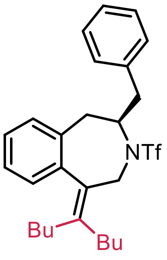

3ab

3ab was obtained as a yellow oil (44.2 mg, 89%). **^{1}H NMR** (500 MHz, $CDCl_3$) δ, ppm: 7.31 – 7.25 (m, 1H), 7.25 – 7.06 (m, 6H), 6.93 (d, *J* = 7.2 Hz, 1H), 4.46 (d, *J* = 16.2 Hz, 1H), 4.19 – 4.03 (m, 2H), 3.16 – 3.00 (m, 1H), 2.99 – 2.83 (m, 1H), 2.69 – 2.47 (m, 2H), 2.13 – 1.94 (m, 3H), 1.94 – 1.77 (m, 1H), 1.44 – 1.30 (m, 4H), 1.27 – 1.16 (m, 2H), 1.15 – 1.01 (m, 2H), 0.93 (t, *J* = 6.6 Hz, 3H), 0.70 (t, *J* = 7.3 Hz, 3H). **^{19}F NMR** (470 MHz, $CDCl_3$) δ, ppm: -77.48 (s). **^{13}C NMR** (126 MHz, $CDCl_3$) δ, ppm: 140.3 (C), 139.0 (C), 138.3 (C), 136.8 (C), 135.9 (C), 130.5 (CH), 129.5 (CH), 129.0 (CH), 128.0 (CH), 127.8 (CH), 127.2 (CH), 126.9 (CH), 119.30 (q, *J* = 333.4 Hz, C), 60.8 (CH), 45.7 (CH_2), 37.2 (CH_2), 32.8 (CH_2), 30.8 (CH_2), 30.7 (CH_2), 30.1 (CH_2), 23.2 (CH_2), 22.8 (CH_2), 14.2 (CH_3), 13.9 (CH_3). **HRMS** (APCI, $[M+H]^+$) *m/z* calculated for $C_{27}H_{35}F_3NO_2S$: 494.2335; found 494.2344. **Rf** = 0.78 (hexane:Et_2O 80:20).

Enantioselectivity of the cycloadduct product was determined by chiral HPLC analysis on Chiralpak IA3 at rt (Hexane, 0.2 mL/min, λ=220 nm).

Racemic sample (*rac*-**3ab**)

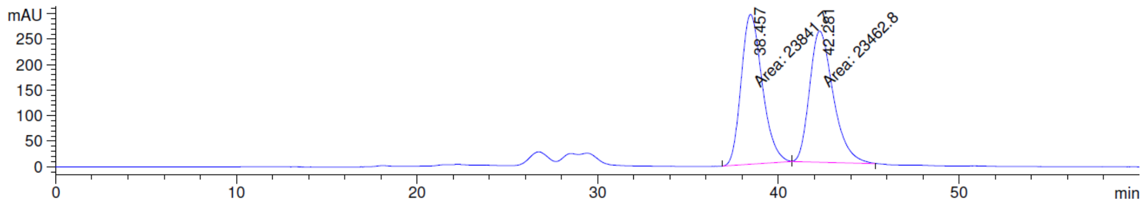

Peak #	RetTime [min]	Type	Width [min]	Area [mAU*s]	Height [mAU]	Area %
1	38.457	MM	1.3553	2.38417e4	293.19177	50.4004
2	42.281	MM	1.5227	2.34628e4	256.81506	49.5996

Asymmetric sample (**3ab**, 98.6:1.4 *e.r.*, 97% *ee*)

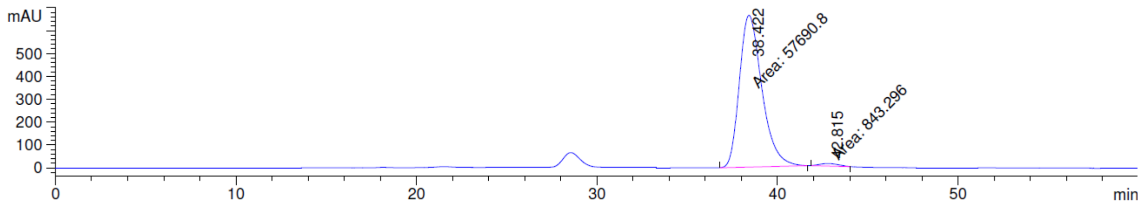

Peak #	RetTime [min]	Type	Width [min]	Area [mAU*s]	Height [mAU]	Area %
1	38.422	MM	1.4482	5.76908e4	663.95721	98.5593
2	42.815	MM	1.1916	843.29602	11.79545	1.4407

(*R*,*Z*)-4-benzyl-1-(4-methoxybenzylidene)-3-((trifluoromethyl)sulfonyl)-2,3,4,5-tetrahydro-1*H*-benzo[*d*]azepine (3ac) + (*R*,*E*)-4-benzyl-1-(4-methoxybenzylidene)-3-((trifluoromethyl)sulfonyl)-2,3,4,5-tetrahydro-1*H*-benzo[*d*]azepine (3ac')

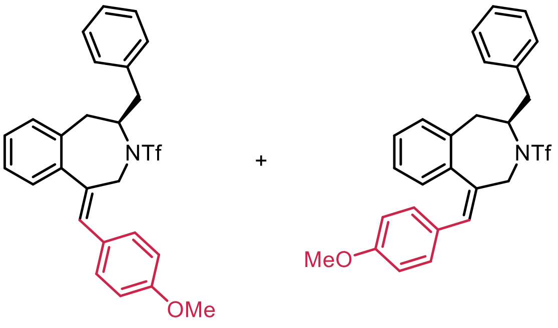

3ac and **3ac'** were obtained as a white solid (35.6 mg, 73%) and as an inseparable mixture of diastereoisomers (*Z*:*E* = 5:1). **^{1}H NMR** (500 MHz, $CDCl_3$) δ, ppm: 7.37 (d, *J* = 7.4 Hz, 0.2H), 7.31 – 7.24 (m, 2.6H), 7.24 – 7.18 (m, 1.8H) 7.18 – 7.08 (m, 3.8H), 7.07 – 6.98 (m, 3H), 6.92 – 6.86 (m, 0.6H), 6.83 (d, *J* = 8.8 Hz, 2H), 6.62 – 6.56 (m, 2.6H), 6.54 (d, *J* = 2.5 Hz, 0.2H), 4.91 – 4.79 (m, 1H), 4.62 (d, *J* = 15.9 Hz, 1H), 4.45 – 4.24 (m, 1.2H), 4.22 – 4.12 (m, 0.2H), 4.09 – 3.93 (m, 1H), 3.78 (s, 0.6H), 3.66 (s, 3H), 3.11 (d, *J* = 12.9 Hz, 0.2H), 3.02 – 2.74 (m, 4.4H), 2.63 (dd, *J* = 14.3, 5.2 Hz, 0.2H). **^{19}F NMR** (282 MHz, $CDCl_3$) δ, ppm: -77.09 (s), -77.13 (s). **^{13}C NMR** (126 MHz, $CDCl_3$) δ, ppm: 159.0 (C), 140.6 (C), 136.9 (C), 136.6 (C), 135.9 (C), 135.3 (C), 134.9 (C), 134.2 (C), 130.8 (CH), 130.6 (CH), 130.0 (CH), 129.6 (CH), 129.4 (CH), 129.0 (CH), 128.9 (CH), 128.5 (CH), 128.4 (CH), 128.2 (CH), 128.0 (CH), 127.6 (CH), 127.3 (CH), 127.2 (CH), 125.7 (CH), 119.6 (q, *J* = 321.3 Hz, C), 114.3 (CH), 113.7 (CH), 58.9 (CH), 55.5 (CH_3), 55.3 (CH_3), 50.4 (CH_2), 45.8 (CH_2), 37.3 (CH_2). **HRMS** (APCI, $[M+H]^+$) *m/z* calculated for $C_{26}H_{25}F_3NO_3S$, 488.1502; found 488.1492. **Rf** = 0.51 (hexane:Et_2O 70:30). **MP** = 52.6-58.9°C.

The stereochemistry of the exocyclic double bond in **3ac** was assigned based on the observed nOe between H_a (4.62 ppm) and H_b (6.8 ppm).

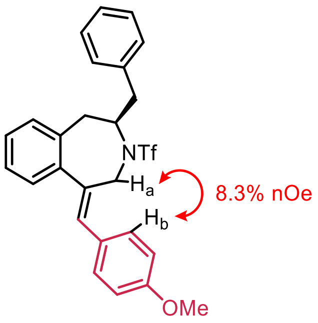

Enantioselectivity of the mixture of diastereoisomers was determined by chiral HPLC analysis on Chiralpak IA3 at rt (Hexane : *i*PrOH = 99:1, 0.5 mL/min, λ=220 nm)

Racemic sample (*rac*-**3ac**)

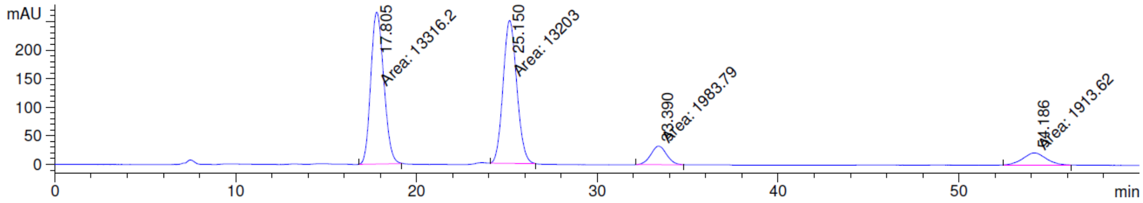

Peak #	RetTime [min]	Type	Width [min]	Area [mAU*s]	Height [mAU]	Area %
1	17.805	MM	0.8349	1.33162e4	265.82874	43.7793
2	25.150	MM	0.8796	1.32030e4	250.17213	43.4073
3	33.390	MM	1.0165	1983.78601	32.52759	6.5220
4	54.186	MM	1.4948	1913.62415	21.33637	6.2914

Asymmetric sample (**3ac**, 96.7:3.3 *e.r.*, 93% *ee*; **3ac'**, 95.5:4.5 *e.r.*, 91% *ee*)

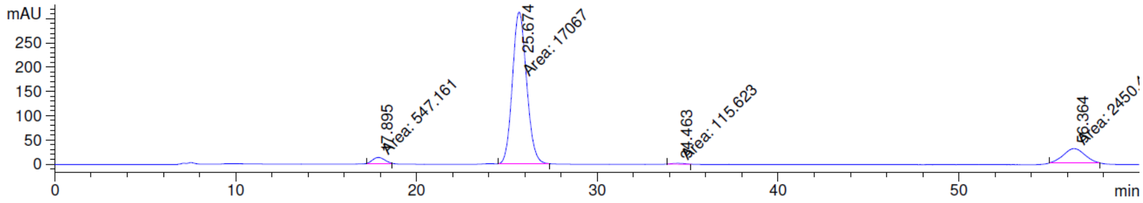

Peak #	RetTime [min]	Type	Width [min]	Area [mAU*s]	Height [mAU]	Area %
1	17.895	MM	0.7531	547.16119	12.10986	2.7114
2	25.674	MM	0.9084	1.70670e4	313.13165	84.5729
3	34.463	MM	0.8819	115.62344	2.18518	0.5730
4	56.364	MM	1.3649	2450.43506	29.92263	12.1427

(*R*,*Z*)-4-benzyl-1-(cyclohexylmethylene)-3-((trifluoromethyl)sulfonyl)-2,3,4,5-tetrahydro-1*H*-benzo[*d*]azepine (3ad)

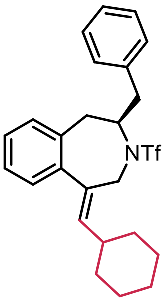

3ad

3ad was obtained as a white solid (31.5 mg, 68%, using **L4** as ligand; 24.4 mg, 53%, using **L5** as ligand) and as a single diastereoisomer (*Z*:*E* > 15:1). **^{1}H NMR** (500 MHz, $CDCl_3$) δ, ppm: 7.31 – 7.25 (m, 2H), 7.25 – 7.12 (m, 5H), 7.10 (td, *J* = 7.3, 1.6 Hz, 1H), 6.85 (dd, *J* = 7.5, 1.3 Hz, 1H), 5.44 (d, *J* = 9.8 Hz, 1H), 4.62 (d, *J* = 18.1 Hz, 1H), 4.22 – 4.11 (m, 1H), 4.06 (d, J = 18.3 Hz, 1H), 3.07 (d, *J* = 12.5 Hz, 1H), 2.91 (t, *J* = 11.5 Hz, 1H), 2.76 – 2.60 (m, 2H), 2.21 – 2.08 (m, 1H), 1.76 – 1.53 (m, 5H), 1.33 – 1.21 (m, 2H), 1.21 – 1.00 (m, 4H). **^{19}F NMR** (470 MHz, $CDCl_3$) δ, ppm: -77.07 (s). **^{13}C NMR** (126 MHz, $CDCl_3$) δ, ppm: 140.3 (C), 137.1 (CH), 136.8 (C), 134.8 (C), 134.3 (C), 129.6 (CH), 129.0 (CH), 128.8 (CH), 128.6 (CH), 128.0 (CH), 127.9 (CH), 127.2 (CH), 119.3 (q, *J* = 326.0 Hz, C), 61.6 (CH), 44.3 (CH_2), 37.2 (CH), 32.8 (CH_2), 32.5 (CH_2), 26.1 (CH_2), 26.0 (CH_2), 25.8 (CH_2). **HRMS** (APCI, $[M+H]^+$) *m/z* calculated for $C_{25}H_{29}F_3NO_2S$, 464.1866; found 464.1871. **Rf** = 0.64 (hexane:Et_2O 80:20). **MP** = 121.1-122.9^{o}C.

The stereochemistry of the exocyclic double bond was assigned based on the observed nOe between H_a (5.44 ppm) and H_b (7.30 ppm) and the absence of nOe between H_a (5.44 ppm) and H_c or H_d (4.62 ppm, 4.06 ppm).

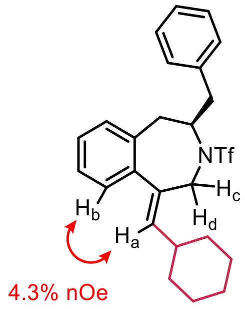

Absolute configuration of the cycloadduct **3ad** was determined as *R* by X-ray crystallography. The structure was deposited in the Cambridge Structural Database; Deposition Number: 2204297.

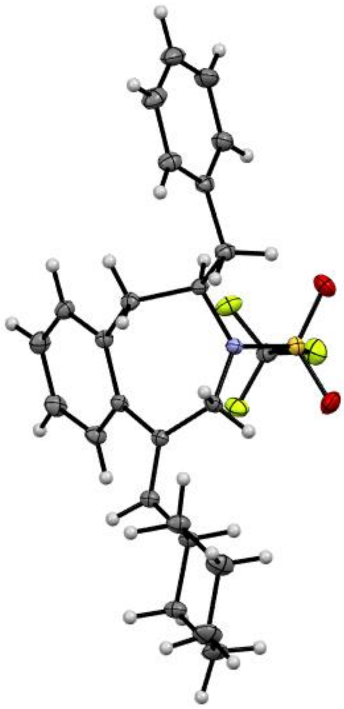

Enantioselectivity of the cycloadduct product was determined by chiral HPLC analysis on Chiralpak IB at rt (Hexane : *i*PrOH = 99:1, 0.5 mL/min, λ=254 nm).

Racemic sample (*rac*-**3ad**)

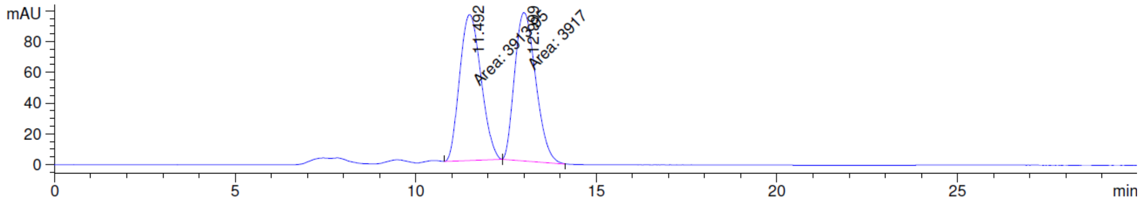

Peak #	RetTime [min]	Type	Width [min]	Area [mAU*s]	Height [mAU]	Area %
1	11.492	MM	0.6876	3913.94556	94.87476	49.9805
2	12.999	MM	0.6768	3917.00171	96.46179	50.0195

Asymmetric sample using ligand **L4** (**3ad**, 97.8:2.2 *e.r.*, 96% *ee*)

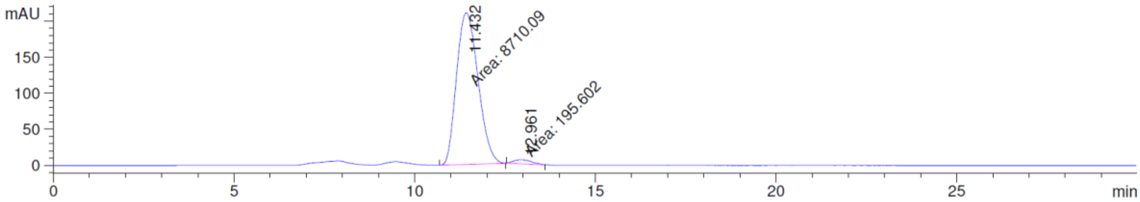

Peak #	RetTime [min]	Type	Width [min]	Area [mAU*s]	Height [mAU]	Area %
1	11.432	MM	0.6893	8710.09082	210.60458	97.8036
2	12.961	MM	0.5814	195.60178	5.60746	2.1964

Asymmetric sample using ligand **L5** (**3ad**, 98.1:1.9 *e.r.*, 96% *ee*)

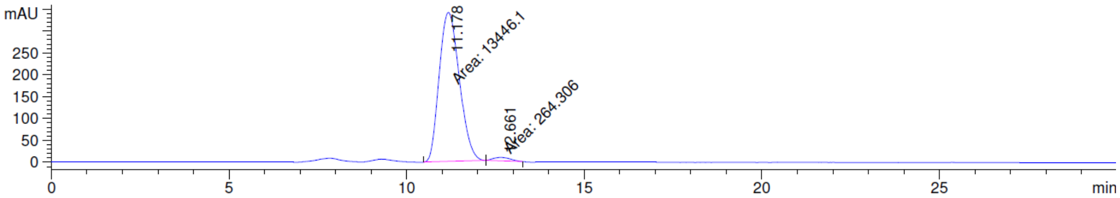

Peak #	RetTime [min]	Type	Width [min]	Area [mAU*s]	Height [mAU]	Area %
1	11.178	MM	0.6588	1.34461e4	340.15125	98.0722
2	12.661	MM	0.5434	264.30643	8.10693	1.9278

tert-butyl (_R_,_Z_)-4-((4-benzyl-3-((trifluoromethyl)sulfonyl)-2,3,4,5-tetrahydro-1_H_-benzo[_d_]azepin-1-ylidene)methyl)piperidine-1-carboxylate (3ae)

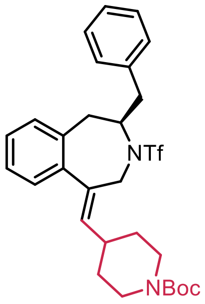

3ae

3ae was obtained as a white solid (40.1 mg, 71%) and as a single diastereoisomer (*Z*:*E* > 15:1) after 48h of reaction. **^{1}H NMR** (500 MHz, $CDCl_3$) δ, ppm: 7.40 – 7.16 (m, 8H), 6.98 – 6.91 (m, 1H), 5.50 (d, *J* = 9.6 Hz, 1H), 4.69 (d, *J* = 17.9 Hz, 1H), 4.33 – 4.01 (m, 4H), 3.21 – 3.06 (m, 1H), 3.06 – 2.93 (m, 1H), 2.89 – 2.69 (m, 4H), 2.47 – 2.31 (m, 1H), 1.79 – 1.67 (m, 1H), 1.66 – 1.54 (m, 1H), 1.48 (s, 9H), 1.40 – 1.24 (m, 2H). **^{19}F NMR** (470 MHz, $CDCl_3$) δ, ppm: -77.05 (s). **^{13}C NMR** (189 MHz, $CDCl_3$) δ, ppm: 155.0 (C), 136.6 (C), 136.4 (C) 134.7 (CH), 134.3 (C), 129.6 (C), 129.0 (CH), 128.9 (CH), 128.3 (CH), 127.9 (CH), 127.3 (CH), 119.62 (q, *J* = 324.6 Hz, C), 79.7 (C), 66.0 (CH_2), 58.5 (CH), 44.2 (CH_2), 43.4 (CH_2), 41.1 (CH_2), 37.3 (CH_2), 35.3 (CH), 31.5 (CH_2), 31.4 (CH_2), 28.6 (CH_3). **HRMS** (APCI, [M-H]$^-$) *m/z* calculated for $C_{29}H_{34}F_3N_2O_4S$, 563.2197; found 563.2180. **Rf** = 0.47 (hexane:Et_2O 70:30). **MP** = 92.3-94.1^{o}C.

The stereochemistry of the exocyclic double bond was assigned based on the observed nOe between H_a (5.50 ppm) and H_b (7.26 ppm) and the absence of nOe between H_a (5.50 ppm) and H_c or H_d (4.69 ppm, 4.11 ppm).

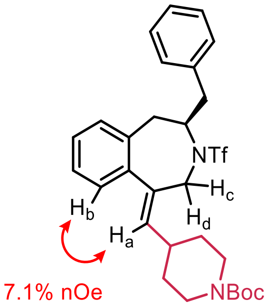

Enantioselectivity of the cycloadduct product was determined by chiral HPLC analysis on Chiralpak IA3 at rt (Hexane : *i*PrOH = 98:2, 0.5 mL/min, λ=254 nm).

Racemic sample (*rac*-**3ae**)

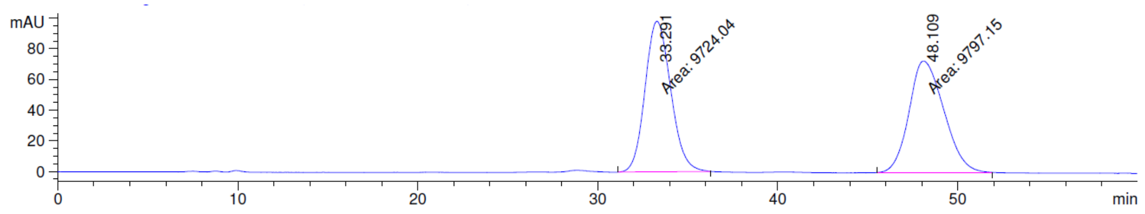

Peak #	RetTime [min]	Type	Width [min]	Area [mAU*s]	Height [mAU]	Area %
1	33.291	MM	1.6535	9724.03711	98.01407	49.8127
2	48.109	MM	2.2478	9797.14844	72.64175	50.1873

Asymmetric sample (**3ae**, 96.7:3.3 *e.r.*, 93% *ee*)

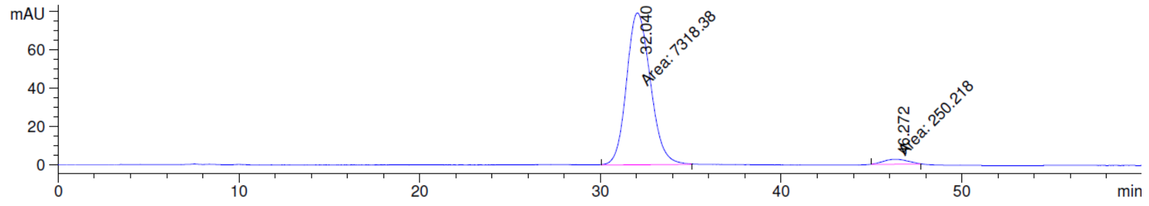

Peak #	RetTime [min]	Type	Width [min]	Area [mAU*s]	Height [mAU]	Area %
1	32.040	MM	1.5362	7318.38477	79.39784	96.6940
2	46.272	MM	1.5733	250.21828	2.65059	3.3060

4.6. Kinetic resolution of homobenzyltriflamides

General procedure for the kinetic resolution of homobenzyltriflamides via (5+2) annulation through a Pd(II)-catalyzed C(sp^2)–H functionalization with allenes

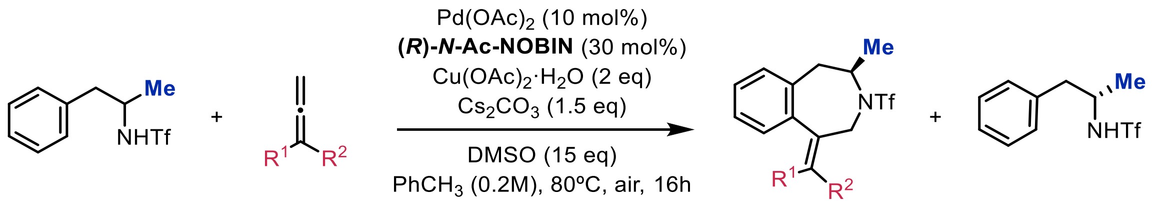

$Pd(OAc)_2$ (2.2 mg, 10 mol%), ligand (*R*)-*N*-Ac-NOBIN (9.8 mg, 30 mol%), $Cu(OAc)_2{\cdot}H_2O$ (40 mg, 2 eq), Cs_2CO_3 (48.9 mg, 1.5 eq) and the corresponding homobenzyltriflamide (0.1 mmol, 1 eq) were weighed in air and placed in a Schlenk tube with a magnetic stir bar. Then, toluene (0.5 mL, 0.2M), DMSO (15 eq) and the corresponding allene (0.2 mmol, 2 eq) were added. The tube was sealed with a rubber septum and the reaction mixture was stirred 16 hours at 80°C. Then, the resulting mixture was cooled to room temperature and filtered through a pad of silica gel and florisil, eluting with Et_2O. Evaporation and column chromatography (silica gel, hexane:Et_2O 99:1 to 80:20) afforded the desired product and the enantioenriched remaining starting material.

(*R*)-1-cyclohexylidene-4-methyl-3-((trifluoromethyl)sulfonyl)-2,3,4,5-tetrahydro-1*H*-benzo[*d*]azepine (3ja)

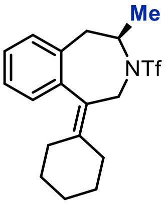

3ja

3ja was obtained as a white solid (17.1 mg, 46%). **^{1}H NMR** (500 MHz, $CDCl_3$) δ, ppm: 7.20 – 7.12 (m, 2H), 7.11 – 7.03 (m, 2H), 4.52 – 4.32 (m, 1H), 4.21 – 3.98 (m, 2H), 2.72 – 2.57 (m, 2H), 2.23 – 2.07 (m, 4H), 2.02 – 1.92 (m, 1H), 1.67 – 1.44 (m, 7H), 1.39 – 1.27 (m, 3H). **^{19}F NMR** (470 MHz, $CDCl_3$) δ, ppm: -77.68 (s). **^{13}C NMR** (126 MHz, $CDCl_3$) δ, ppm: 145.1 (C), 142.5 (C), 137.8 (C), 136.4 (C), 130.9 (CH), 128.0 (CH), 127.7 (CH), 126.9 (CH), 120.2 (q, *J* = 326.6 Hz, C), 51.7 (CH), 44.5 (CH_2), 40.1(CH_2), 32.5 (CH_2), 30.2 (CH_2), 28.6 (CH_2), 27.9 (CH_2), 26.7 (CH_2). **HRMS** (APCI, $[M+H]^+$) *m/z* calculated for $C_{18}H_{23}F_3NO_2S$: 374.1396; found 374.1396. **Rf** = 0.67 (hexane:Et_2O 80:20). **MP** = 65.2-67.1^{o}C.

Enantioselectivity of the cycloadduct product was determined by chiral HPLC analysis on Chiralpak IB at rt (Hexane, 0.2 mL/min, λ=220 nm).

Racemic sample (*rac*-**3ja**)

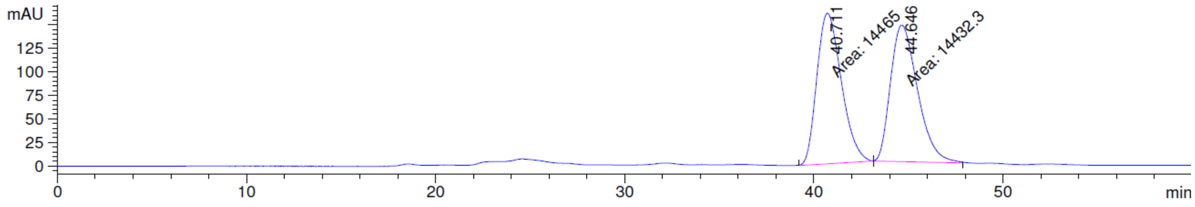

Peak #	RetTime [min]	Type	Width [min]	Area [mAU*s]	Height [mAU]	Area %
1	40.711	MM	1.5043	1.44650e4	160.26340	50.0567
2	44.646	MM	1.6568	1.44323e4	145.17799	49.9433

Asymmetric sample (**3ja**, 96.7:3.3 *e.r.*, 93% *ee*)

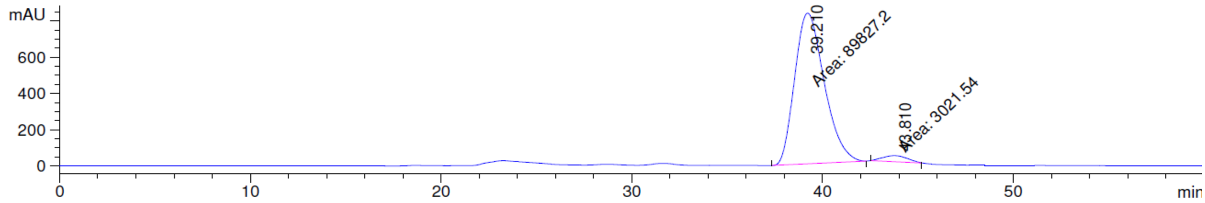

Peak #	RetTime [min]	Type	Width [min]	Area [mAU*s]	Height [mAU]	Area %
1	39.210	MM	1.8001	8.98272e4	831.70490	96.7457
2	43.810	MM	1.4935	3021.54395	33.71909	3.2543

Enantioselectivity of the remaining starting material was determined by chiral HPLC analysis on Chiralpak IB at rt (Hexane : *i*PrOH = 98:2, 0.5 mL/min, λ=210 nm).

Racemic sample (*rac*-**1j**)

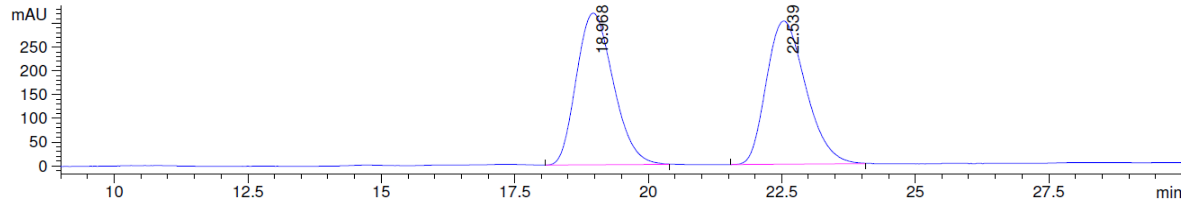

Peak #	RetTime [min]	Type	Width [min]	Area [mAU*s]	Height [mAU]	Area %
1	18.968	PB	0.7393	1.53365e4	318.79587	49.8362
2	22.539	BB	0.7809	1.54374e4	300.70810	50.1638

Asymmetric sample (**1j**, 98.9:1.1 *e.r.*, 98% *ee*)

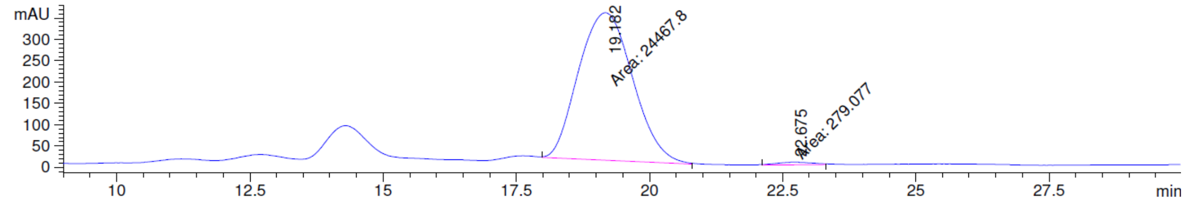

Peak #	RetTime [min]	Type	Width [min]	Area [mAU*s]	Height [mAU]	Area %
1	19.182	MM	1.1791	2.44678e4	345.85205	98.8723
2	22.675	MM	0.7723	279.07660	6.02254	1.1277

5. Synthetic manipulations of the enantioenriched cycloadducts

5.1. Hydrogenation of the exocyclic double bond and deprotection of the *N*-triflyl group

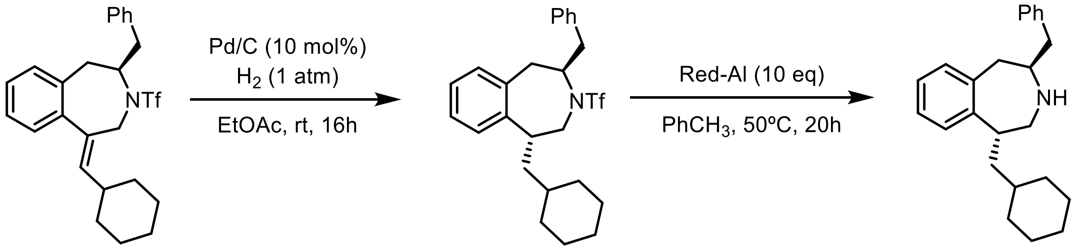

3ad 96% *ee* **4** *d.r.*>20:1 **5** 95% *ee*

According to the literature,[7] a nitrogen flushed Schlenk was charged with compound **3ad** (139.1 mg, 0.3 mmol), Pd/C (63.9 mg, 0.03 mmol) and ethyl acetate (3 mL, 0.1 M). A H_2 balloon was bubbled, and the resulting suspension was stirred at rt for 16h, until TLC indicates full conversion of starting material, under H_2 atmosphere. The mixture was filtered through Celite®, the filtrate was concentrated, and the obtained residue was purified by column chromatography on silica gel (0.5% Et_2O/hexanes) to afford **4** as a white solid (110.1 mg, 80% yield).

Following a method reported in the literature,[7a,8] to a stirred solution of the previously obtained **4** (40 mg, 0.086 mmol) in toluene (0.9 mL, 0.1 M) at 0°C and under argon was added sodium bis(2-methoxyethoxy)-aluminum hydride (60 wt. % in toluene, 0.26 mL, 0.86 mmol, 10 eq). The resulting mixture was stirred for 20h at 50°C. After completion, it was cooled to 0°C and quenched by addition of 5% aq. NH_4Cl (5 mL). This mixture was extracted with dichloromethane, dried over Na_2SO_4 and concentrated under vacuum. The obtained residue was purified by column chromatography on silica gel (DCM to DCM/MeOH/Et_3N 98:1:1) to afford the amine **5** as a yellowish oil (28 mg, 98% yield).

(1*R*,4*R*)-4-benzyl-1-(cyclohexylmethyl)-3-((trifluoromethyl)sulfonyl)-2,3,4,5-tetrahydro-1*H*-benzo[*d*]azepine (4)

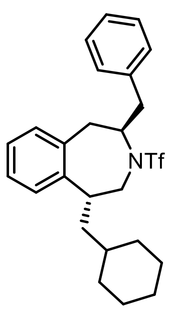

4

4 was obtained as a white solid (111.5 mg, 80%) a single diastereoisomer (*d.r.* > 20:1). **^{1}H NMR** (500 MHz, $CDCl_3$) δ, ppm: 7.28 – 7.20 (m, 2H), 7.20 – 7.08 (m, 3H), 7.08 – 6.93 (m, 3H), 6.86 – 6.78 (m, 1H), 4.54 – 4.34 (m, 1H), 4.07 – 3.79 (m, 1H), 3.56 (dd, J = 14.6, 2.6 Hz, 1H), 3.37 – 3.10 (m, 1H), 3.08 – 2.87 (m, 1H), 2.85 – 2.47 (m, 3H), 1.78 (d, J = 12.7 Hz, 1H), 1.71 – 1.44 (m, 6H), 1.21 – 0.98 (m, 4H), 0.90 – 0.77 (m, 2H). **^{19}F NMR** (470 MHz, $CDCl_3$) δ, ppm: -75.77 (s). **^{13}C NMR** (126 MHz, $CDCl_3$) δ, ppm: 136.9 (C), 135.1 (C), 133.2 (C), 129.5 (CH), 129.4 (CH), 128.8 (CH), 127.6 (CH), 127.3 (CH), 127.2 (CH), 127.0 (CH), 120.27 (q, J = 312.6 Hz, C), 58.1 (CH), 47.8 (CH_2), 35.0 (CH), 34.6 (CH), 34.1 (CH_2), 34.0 (CH_2), 33.2 (CH_2), 26.7 (CH_2), 26.3 (CH_2), 26.3 (CH_2). **HRMS** (APCI, $[M+H]^+$) m/z calculated for $C_{25}H_{31}F_3NO_2S$: 466.2022; found 466.2039. **Rf** = 0.75 (hexane:Et_2O 95:5). **MP** = 110.8-112.2°C.

Absolute configuration of the cycloadduct **4** was determined by X-ray crystallography. The structure was deposited in the Cambridge Structural Database; Deposition Number: 2204513.

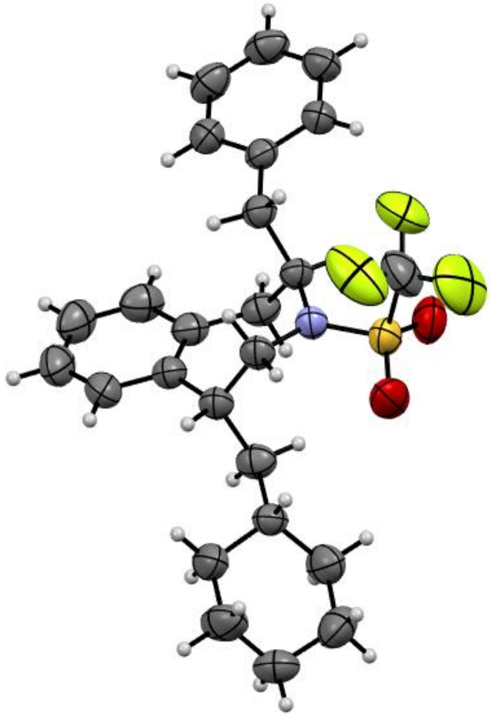

(1*R*,4*R*)-4-benzyl-1-(cyclohexylmethyl)-2,3,4,5-tetrahydro-1*H*-benzo[*d*]azepine (5)

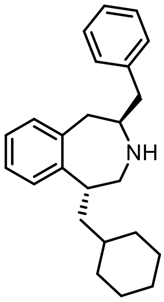

5

5 was obtained as a yellow oil (28.1 mg, 98%). **^{1}H NMR** (500 MHz, $CDCl_3$) δ, ppm: 7.24 – 7.19 (m, 2H), 7.17 – 7.08 (m, 4H), 7.07 – 7.01 (m, 2H), 6.97 – 6.93 (m, 1H), 3.12 – 2.99 (m, 2H), 2.99 – 2.91 (m, 1H), 2.91 – 2.81 (m, 2H), 2.74 – 2.65 (m, 2H), 2.44 (dd, *J* = 12.9, 7.9 Hz, 1H), 1.82 – 1.49 (m, 6H), 1.43 – 1.34 (m, 1H), 1.34 – 1.21 (m, 1H), 1.21 – 1.02 (m, 4H), 0.94 – 0.74 (m, 2H). **^{13}C NMR** (126 MHz, $CDCl_3$) δ, ppm: 145.0 (C), 139.9 (C), 139.3 (C), 130.1 (CH), 129.4 (CH), 129.4 (CH), 128.7 (CH), 126.5 (CH), 126.5 (CH), 126.0 (CH), 58.3 (CH), 42.7 (CH_2), 42.1 (CH), 39.9 (CH_2), 39.0 (CH_2), 35.1 (CH), 34.2 (CH_2), 33.8 (CH_2), 26.8 (CH_2), 26.5 (CH_2). **HRMS** (APCI, $[M+H]^+$) *m/z* calculated for $C_{24}H_{32}N$: 334.2529; found 334.2533. **IR** (cm^{-1}): 3402, 2930, 2871, 1193, 1155, 834, 783, 735, 715, 622, 551. **Rf** = 0.36 (DCM:MeOH 95:5).

Enantiomeric excess of the free amine **5** was determined by chiral HPLC analysis on Chiralpak IA3 at rt (Hexane : *i*PrOH = 90:10, 1 mL/min, λ=220 nm).

Racemic sample (*rac*-**5**)

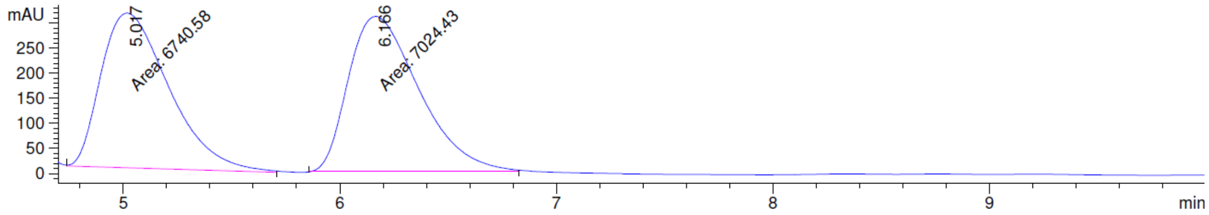

Peak #	RetTime [min]	Type	Width [min]	Area [mAU*s]	Height [mAU]	Area %
1	5.017	MM	0.3638	6740.58008	308.80704	48.9689
2	6.166	MM	0.3783	7024.43115	309.50696	51.0311

Asymmetric sample (**5**, 97.7:2.3 *e.r.*, 95% *ee*)

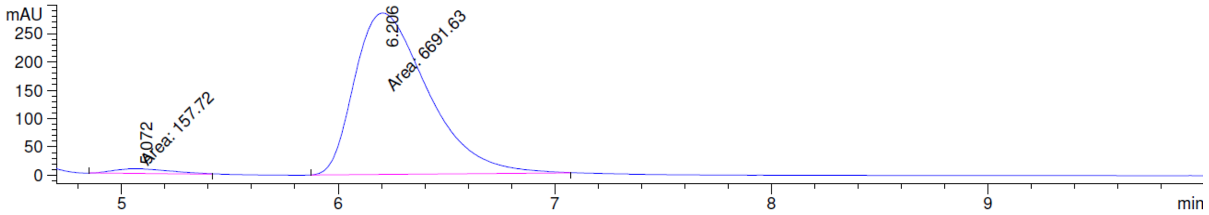

Peak #	RetTime [min]	Type	Width [min]	Area [mAU*s]	Height [mAU]	Area %
1	5.072	MM	0.3124	157.71997	8.41350	2.3027
2	6.206	MM	0.3919	6691.62891	284.59036	97.6973

5.2. Oxidative cleavage of the exocyclic double bond

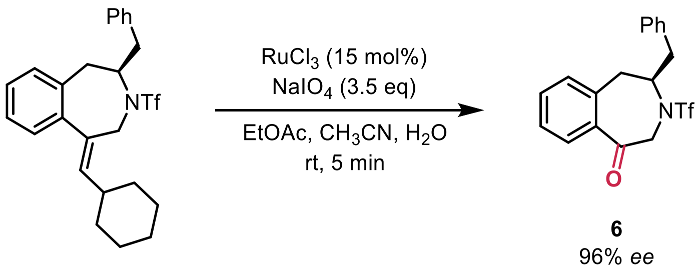

3ad 96% *ee* → **6** 96% *ee*

According to the literature,[7a,9] a solution of $NaIO_4$ (74.9 mg, 0.35 mmol, 3.5 eq) in water (0.6 mL, 0.17 M) was added to a solution of $RuCl_3$ (3.1 mg, 0.015 mmol, 15 mol%) in MeCN (0.8 mL, 0.13 M). This mixture was stirred 5 minutes and then a solution of the compound **3ad** (46.4 mg, 0.1 mmol) in EtOAc (0.8 mL, 0.13 M) was added. The mixture was stirred for 5 minutes until TLC indicates complete consumption of the starting material. $MgSO_4$ was then added, and the resulting heterogeneous mixture was filtered through Celite® eluting with EtOAc. The resulting organic phase was concentrated in vacuo and the crude residue was purified by column chromatography on silica gel (5 to 20% Et_2O/hexanes) to yield the ketone **6** as a white solid (20.2 mg, 52%).

(*R*)-4-benzyl-3-((trifluoromethyl)sulfonyl)-2,3,4,5-tetrahydro-1*H*-benzo[*d*]azepin-1-one (6)

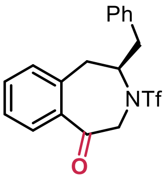

6

6 was obtained as a white solid (20.2 mg, 52%). **^{1}H NMR** (500 MHz, $CDCl_3$) δ, ppm: 7.66 (d, *J* = 7.7 Hz, 1H), 7.41 (td, *J* = 7.5, 1.4 Hz, 1H), 7.35 – 7.29 (m, 3H), 7.26 (t, *J* = 7.2 Hz, 1H), 7.24 – 7.17 (m, 2H), 7.04 (d, *J* = 7.5 Hz, 1H), 4.46 – 4.13 (m, 2H), 3.81 (d, *J* = 19.4 Hz, 1H), 3.15 – 3.06 (m, 1H), 3.06 – 2.96 (m, 1H), 2.96 – 2.87 (m, 1H), 2.81 (dd, *J* = 14.9, 5.1 Hz, 1H). **^{19}F NMR** (470 MHz, $CDCl_3$) δ, ppm: -76.75 (s). **^{13}C NMR** (126 MHz, $CDCl_3$) δ, ppm: 200.3 (C), 136.5 (C), 136.0 (C), 135.6 (C), 133.9 (CH), 129.7 (CH), 129.5 (CH), 129.2 (CH), 128.5 (CH), 127.7 (CH), 119.32 (q, *J* = 322.7 Hz, C), 66.0 (CH_2) 60.5 (CH), 53.4 (CH_2), 37.4 (CH_2). **HRMS** (APCI, $[M+H]^+$) *m/z* calculated for $C_{18}H_{17}F_3NO_3S$: 384.0876; found 384.0877. **IR** (cm^{-1}): 2925, 2855, 1688, 1602, 1452, 1377, 1187, 1130, 1082, 903, 750, 701, 640, 570, 491. **Rf** = 0.56 (hexane:Et_2O 60:40). **MP** = 119.1-120.5^{o}C.

Enantiomeric excess of the ketone **6** was determined by chiral HPLC analysis on Chiralpak IA3 at rt (Hexane : *i*PrOH = 98:2, 0.5 mL/min, λ=254 nm).

Racemic sample (*rac*-**6**)

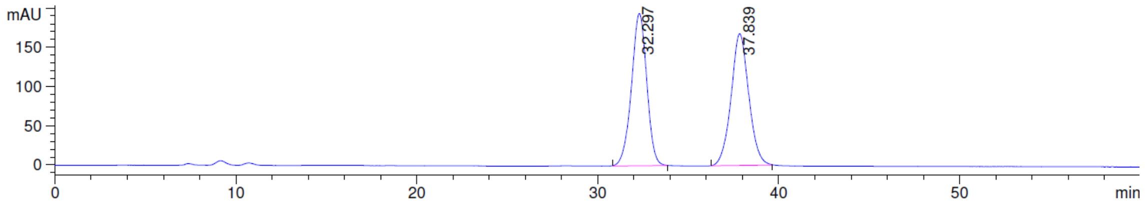

Peak #	RetTime [min]	Type	Width [min]	Area [mAU*s]	Height [mAU]	Area %
1	32.297	BB	0.8942	1.14136e4	194.05838	49.9181
2	37.839	BB	1.0115	1.14511e4	167.93942	50.0819

Asymmetric sample (**6**, 97.8:2.2 *e.r.*, 96% *ee*)

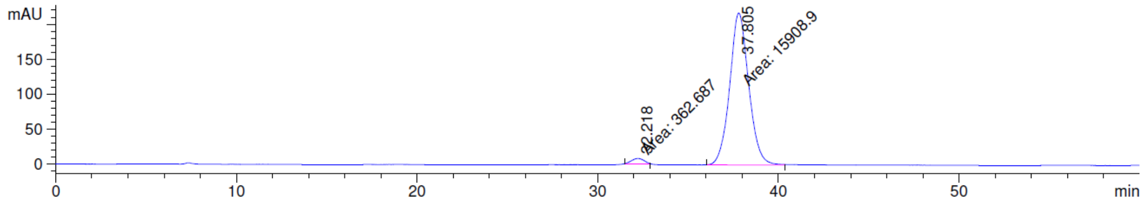

Peak #	RetTime [min]	Type	Width [min]	Area [mAU*s]	Height [mAU]	Area %
1	32.218	MM	0.7832	362.68716	7.71809	2.2290
2	37.805	MM	1.2195	1.59089e4	217.42201	97.7710

6. Kinetic resolution of *ortho*-methyl benzyltriflamides

General procedure for the kinetic resolution of *ortho*-methyl benzyltriflamides via (5+2) annulation through a Pd(II)-catalyzed C(sp^3)–H functionalization with allenes

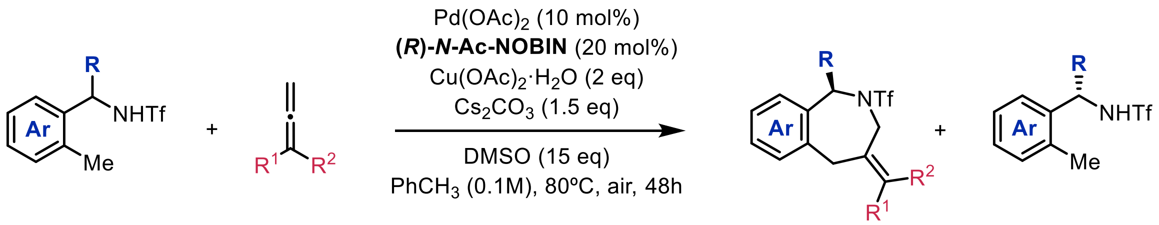

$Pd(OAc)_2$ (2.2 mg, 10 mol%), ligand (*R*)-*N*-Ac-NOBIN (6.5 mg, 20 mol%), $Cu(OAc)_2 \cdot H_2O$ (40 mg, 2 eq), Cs_2CO_3 (48.9 mg, 1.5 eq) and the corresponding *o*-methylbenzyltriflamide (0.1 mmol, 1 eq) were weighed in air and placed in a Schlenk tube with a magnetic stir bar. Then, toluene (1 mL, 0.1M), DMSO (15 eq) and the corresponding allene (0.1 mmol, 1 eq) were added. The tube was sealed with a rubber septum and the reaction mixture was stirred 48 hours at 80°C. Then, the resulting mixture was cooled to room temperature and filtered through a pad of silica gel and florisil, eluting with Et_2O. Evaporation and column chromatography (silica gel, hexane:Et_2O 99:1 to 80:20) afforded the desired product and the enantioenriched remaining starting material.

(*R*)-4-cyclohexylidene-1,7,9-trimethyl-2-((trifluoromethyl)sulfonyl)-2,3,4,5-tetrahydro-1*H*-benzo[*c*]azepine (8aa)

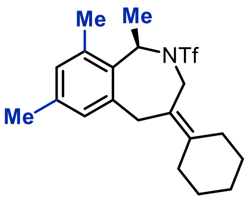

8aa

8aa was obtained as a white solid (19.4 mg, 48% yield). **^{1}H NMR** (500 MHz, $CDCl_3$) δ, ppm: 6.84 (d, *J* = 6.5 Hz, 2H), 5.45 (q, *J* = 7.5, 7.1 Hz, 1H), 4.42 – 4.29 (m, 2H), 3.63 (d, *J* = 15.4 Hz, 1H), 3.53 (d, *J* = 15.4 Hz, 1H), 2.39 – 2.31 (m, 1H), 2.31 – 2.20 (m, 7H), 2.19 – 2.05 (m, 2H), 1.62 – 1.53 (m, 3H), 1.51 – 1.46 (m, 6H). **^{19}F NMR** (282 MHz, $CDCl_3$) δ, ppm: -76.01 (s). **^{13}C NMR** (126 MHz, $CDCl_3$) δ, ppm: 138.2 (C), 138.0 (C), 136.7 (C), 135.3 (C), 134.3 (C), 130.0 (CH), 129.3 (CH), 123.1 (C), 120.3 (q, *J* = 324.1 Hz, C), 55.6 (CH), 47.9 (CH_2), 36.7 (CH_2), 31.1 (CH_2), 30.3 (CH_2), 27.7 (CH_2), 27.6 (CH_2), 26.7 (CH_2), 20.9 (CH_3), 19.5 (CH_3), 19.2 (CH_3). **HRMS** (APCI, $[M+H]^+$) *m/z* calculated for $C_{20}H_{27}F_3NO_2$: 402.1709; found 402.1717. **IR** (cm^{-1}): 2925, 2854, 1716, 1446, 1384, 1225, 1178, 1150, 1097, 1003, 622, 608, 587. **Rf** = 0.68 (hexane:Et_2O 80:20). **MP** = 85.1-86.2°C.

Absolute configuration of the cycloadduct **8aa** was determined as *R* by X-ray crystallography. The structure was deposited in the Cambridge Structural Database; Deposition Number: 2204767.

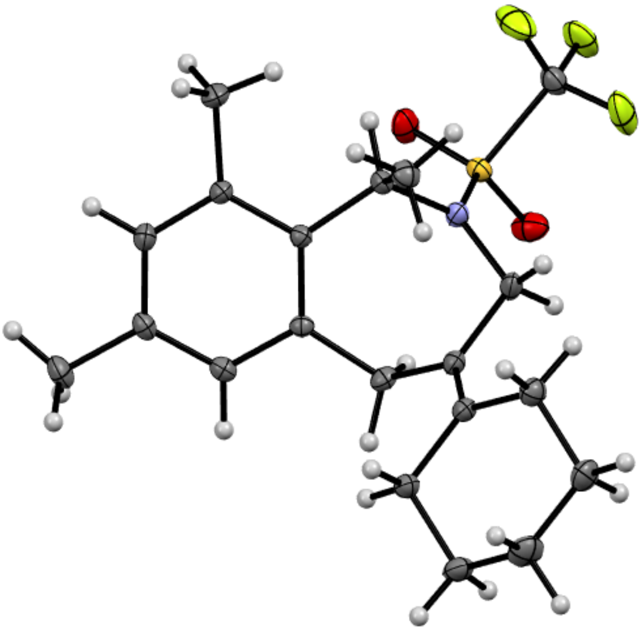

Enantioselectivity of the cycloadduct product was determined by chiral HPLC analysis on Chiralpak IA-3 at rt (Hexane, 0.3 mL/min, λ =220 nm).

Racemic sample (*rac*-**8aa**)

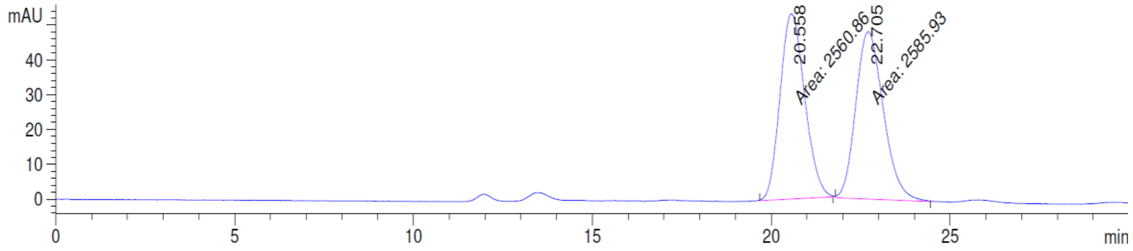

Signal 1: DAD1 A, Sig=220,8 Ref=360,100

Peak #	RetTime [min]	Type	Width [min]	Area [mAU*s]	Height [mAU]	Area %
1	20.558	MM	0.8029	2560.86084	53.16013	49.7565
2	22.705	MM	0.8959	2585.92993	48.10412	50.2435

Totals : 5146.79077 101.26425

Asymmetric sample (**8aa**, 97.7:2.3 *er*, 95% *ee*)

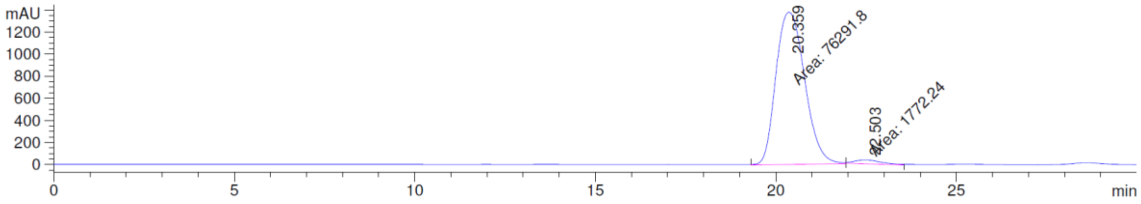

Signal 1: DAD1 A, Sig=220,8 Ref=360,100

Peak #	RetTime [min]	Type	Width [min]	Area [mAU*s]	Height [mAU]	Area %
1	20.359	MM	0.9227	7.62918e4	1378.06604	97.7298
2	22.503	MM	0.8603	1772.23511	34.33171	2.2702

Totals : 7.80640e4 1412.39775

Enantioselectivity of the remaining starting material was determined by chiral SFC analysis on Phenomenex Cellulose-1 at 40°C (CO_2 : MeOH = 98:2, 0.5 mL/min, λ=222 nm).

Racemic sample (*rac*-**7a**)

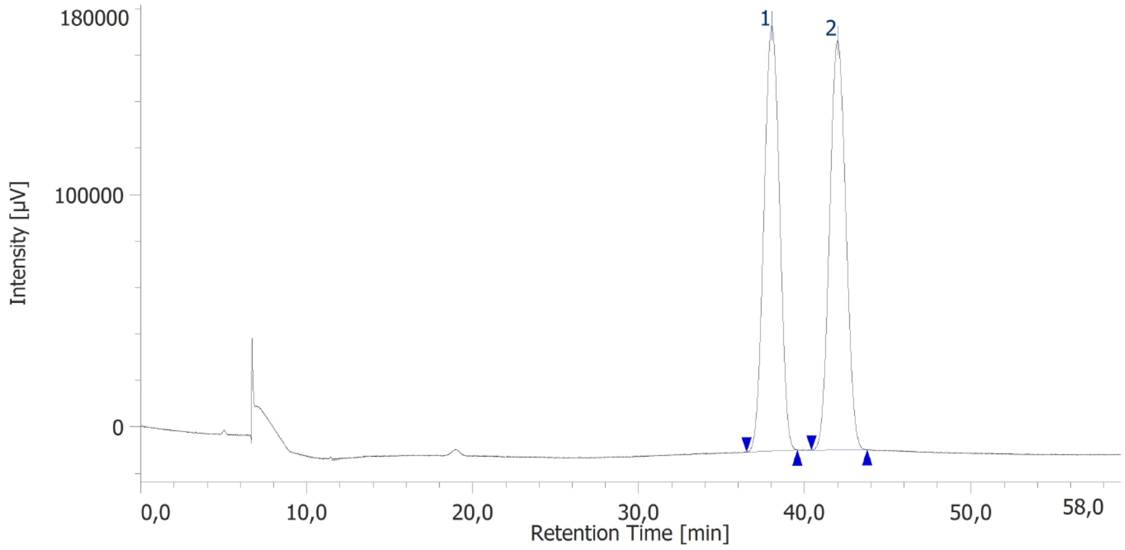

#	Peak Name	CH	tR [min]	Area [μV·sec]	Height [μV]	Area%	Height%	Quantity	NTP	Resolution	Symmetry Factor	Warning
1	Unknown	5	38,023	12004834	182984	50,013	50,932	N/A	7290	2,195	0,998	
2	Unknown	5	41,983	11998750	176285	49,987	49,068	N/A	8358	N/A	1,043	

Asymmetric sample (**7a**, 94.8:5.2 *e.r.*, 90% *ee*)

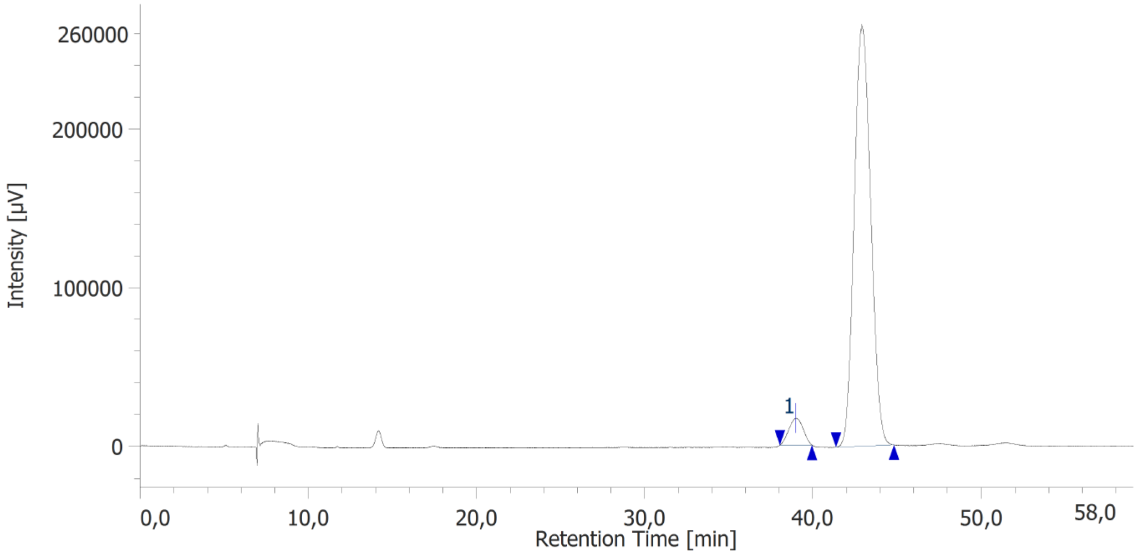

#	Peak Name	CH	tR [min]	Area [μV·sec]	Height [μV]	Area%	Height%	Quantity	NTP	Resolution	Symmetry Factor	Warning
1	Unknown	6	38,987	1003590	17030	5,238	6,042	N/A	8903	2,251	0,998	
2	Unknown	6	42,917	18155295	264825	94,762	93,958	N/A	8630	N/A	1,102	

(*R*)-4-(4-methoxybenzylidene)-1,7,9-trimethyl-2-((trifluoromethyl)sulfonyl)-2,3,4,5-tetrahydro-1*H*-benzo[*c*]azepine (8ac)

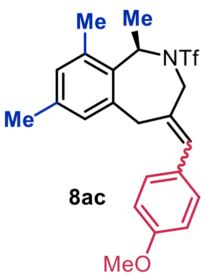

8ac

Reaction performed with 1.5 equiv of allene. **8ac** was obtained as a transparent oil (18.7 mg, 43% yield) and as an inseparable mixture of diastereoisomers (*E*:*Z* = 7.7:1). **^{1}H NMR** (500 MHz, $CDCl_3$) δ, ppm: 7.15 (d, *J* = 8.7 Hz, 2.03H), 7.04 (d, *J* = 8.6 Hz, 0.30H), 6.92 (d, *J* = 12.5 Hz, 2.26H), 6.87 (d, *J* = 8.6 Hz, 2.11H), 6.60 (s, 0.13H), 6.54 (s, 1.00H), 5.55 (s, 0.97H), 4.75 (d, *J* = 14.6 Hz, 0.13H), 4.67 – 4.59 (m, 0.13H), 4.40 (d, *J* = 15.5 Hz, 0.13H), 4.18 (d, *J* = 14.6 Hz, 1.00H), 3.95 (d, *J* = 13.0 Hz, 0.2H), 3.82 (s, 3.46H), 3.80 – 3.66 (m, 2.30H), 3.58 (d, *J* = 15.2 Hz, 0.13H), 2.33 (d, *J* = 9.6 Hz, 6.01H), 2.29 (d, *J* = 7.2 Hz, 0.79H), 1.63 (d, *J* = 7.2 Hz, 3.24H). **^{19}F NMR** (282 MHz, $CDCl_3$) δ, ppm: -76.42 (s). **^{13}C NMR** (126 MHz, $CDCl_3$, only *E* isomer assigned) δ, ppm: 159.0 (C), 137.3 (C), 137.2 (C), 136.1 (C), 134.8 (C), 134.0 (C), 130.3 (CH), 130.0 (CH), 129.7 (CH), 128.9 (C), 128.8 (CH), 120.3 (q, *J* = 324.0 Hz, C), 113.8 (CH), 55.6 (CH), 55.4 (CH_3), 54.1 (CH_2), 37.2 (CH_2), 21.0 (CH_3), 19.6 (CH_3), 19.5 (CH_3). **HRMS** (APCI, $[M+H]^+$) *m/z* calculated for $C_{22}H_{25}F_3NO_3S$: 440.1502; found 440.1513. **Rf** = 0.56 (hexane:Et_2O 70:30). **MP** = 86.3-91.1°C.

Assignment of stereochemistry based on the observed NOE between the H_a (6.54 ppm, 100%) with H_b (4.18 ppm, 4.3%) and between H_c (6.60 ppm) and H_d (3.58 ppm, detected by 2D-NOESY experiments).

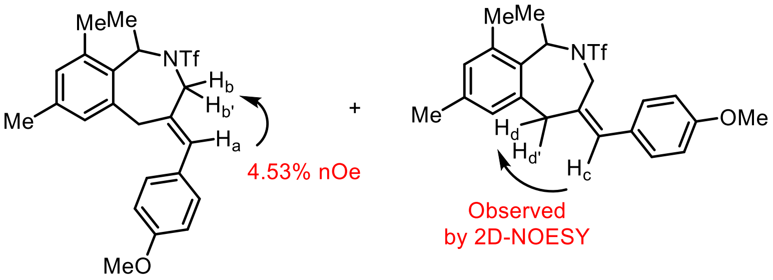

For measurement of the enantiomeric excess of the cycloadduct **8ac** the following transformation has been applied:

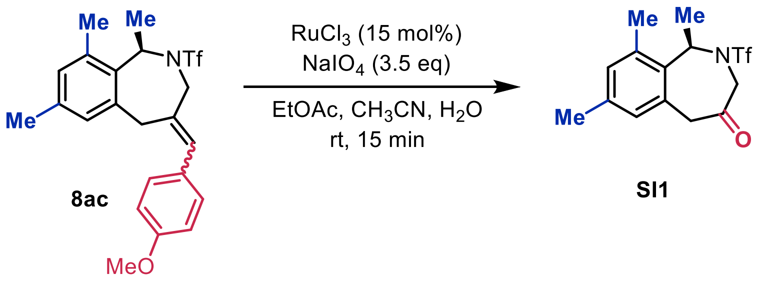

Following a previously reported procedure,[7a,9] a solution of $NaIO_4$ (33.6 mg, 3.5 eq) in water (0.26 mL, 0.17 M) was added to a solution of $RuCl_3$ (1.5 mg, 15 mol%) in MeCN (0.35 mL, 0.13 M). This mixture was stirred 15 minutes and then a solution of the compound **8ac** (19.7 mg, 0.049 mmol) in EtOAc (0.35 mL, 0.13 M) was added. The mixture was stirred for 15 minutes until TLC indicates complete consumption of the starting material. $MgSO_4$ was then added and the resulting heterogeneous mixture was filtered through Celite® and eluted with EtOAc. The resulting organic phase was concentrated in vacuo and the crude residue was purified by column chromatography over silica gel (hexanes : diethyl ether; 95:5-90:10), affording the ketone **SI1** as a white solid (8.5 mg, 56% yield). **^{1}H NMR** (500 MHz, $CDCl_3$) δ, ppm: 6.93 (s, 1H), 6.83 (s, 1H), 5.56 (q, *J* = 7.4 Hz, 1H), 4.57 (d, *J* = 17.3 Hz, 1H), 4.45 (d, *J* = 13.4 Hz, 1H), 3.98 (d, *J* = 17.3 Hz, 1H), 3.63 (d, *J* = 13.4 Hz, 1H), 2.34 (s, 3H), 2.27 (s, 3H), 1.41 (d, *J* = 7.2 Hz, 3H). **^{19}F NMR** (282 MHz, $CDCl_3$) δ, ppm: -76.24 (s). **^{13}C NMR** (126 MHz, $CDCl_3$) δ, ppm: 203.9 (C), 137.6 (C), 134.6 (C), 134.5 (C), 131.4 (CH), 130.7 (CH), 128.9 (C), 120.1 (q, *J* = 323.0 Hz, C), 55.7 (CH), 55.2 (CH_2), 49.6 (CH_2), 20.8 (CH_3), 20.1 (CH_3), 19.5 (CH_3). **HRMS** (APCI, $[M-H]^-$) *m/z* calculated for $C_{14}H_{15}F_3NO_3S$: 334.0730; found 334.0740. **Rf** = 0.43 (hexane:Et_2O 60:40). **MP** = 110.1-111.7^{o}C.

Enantiomeric excess of the ketone **SI1** was determined by chiral HPLC analysis on Chiralpak IB at rt (Hexane : *i*PrOH = 99:1, 0.5 mL/min, λ=220 nm).

Racemic sample (*rac*-**SI1**)

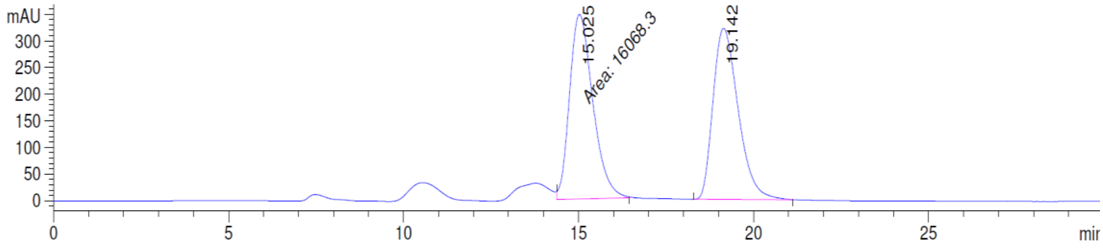

Signal 1: DAD1 A, Sig=220,8 Ref=360,100

Peak #	RetTime [min]	Type	Width [min]	Area [mAU*s]	Height [mAU]	Area %
1	15.003	MM	0.6495	1167.85474	29.96639	4.2536
2	18.679	PB	0.7250	2.62879e4	564.79004	95.7464

Totals : 2.74557e4 594.75643

Asymmetric sample (**SI1**, 95.7:4.3 *er*, 91% *ee*)

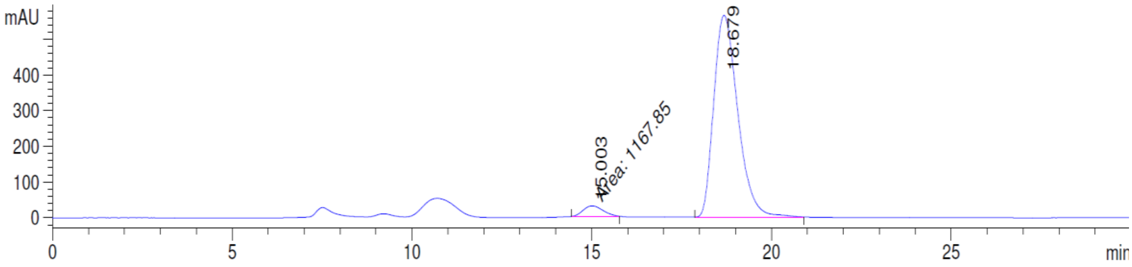

Signal 1: DAD1 A, Sig=220,8 Ref=360,100

Peak #	RetTime [min]	Type	Width [min]	Area [mAU*s]	Height [mAU]	Area %
1	15.003	MM	0.6495	1167.85474	29.96639	4.2536
2	18.679	PB	0.7250	2.62879e4	564.79004	95.7464

Totals : 2.74557e4 594.75643

Enantioselectivity of the remaining starting material was determined by chiral HPLC analysis on Chiralpak IB at rt (Hexane : *i*PrOH = 99:1, 0.5 mL/min, λ=220 nm).

Racemic sample (*rac*-**7a**)

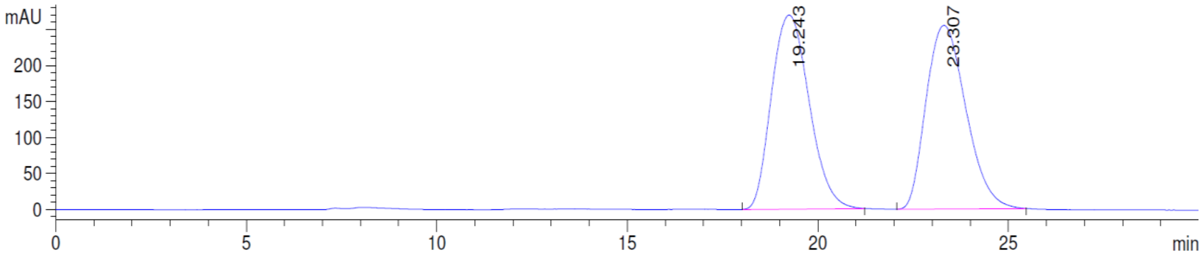

Signal 1: DAD1 A, Sig=220,8 Ref=360,100

Peak #	RetTime [min]	Type	Width [min]	Area [mAU*s]	Height [mAU]	Area %
1	19.243	BB	1.0908	1.85869e4	269.45322	50.0366
2	23.307	BB	1.1314	1.85597e4	254.90724	49.9634

Totals : 3.71466e4 524.36046

Asymmetric sample (**7a**, 86.0:14.0 *er*, 72% *ee*)

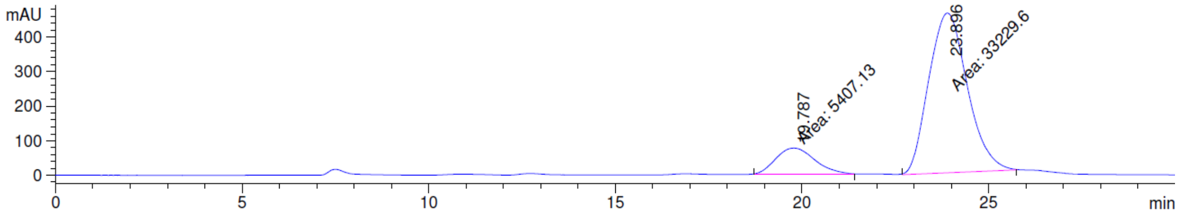

Signal 1: DAD1 A, Sig=220,8 Ref=360,100

Peak #	RetTime [min]	Type	Width [min]	Area [mAU*s]	Height [mAU]	Area %
1	19.787	MM	1.2031	5407.12744	74.90763	13.9948
2	23.896	MM	1.1990	3.32296e4	461.90591	86.0052

Totals : 3.86368e4 536.81355

(*R*)-9-chloro-4-cyclohexylidene-1-methyl-2-((trifluoromethyl)sulfonyl)-2,3,4,5-tetrahydro-1*H*-benzo[*c*]azepine

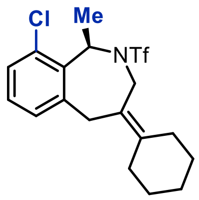

8ba

8ba was obtained as a yellowish solid (19.1 mg, 47% yield). **^{1}H NMR** (500 MHz, $CDCl_3$) δ, ppm: 7.24 – 7.18 (m, 1H), 7.11 – 7.06 (m, 2H), 5.69 (q, *J* = 7.0 Hz, 1H), 4.38 (s, 2H), 3.69 (d, *J* = 15.5 Hz, 1H), 3.58 (d, *J* = 15.4 Hz, 1H), 2.32 – 2.02 (m, 4H), 1.61 – 1.46 (m, 9H). **^{19}F NMR** (282 MHz, $CDCl_3$) δ, ppm: -76.41 (s). **^{13}C NMR** (126 MHz, $CDCl_3$) δ, ppm: 140.3 (C), 138.9 (C), 137.2 (C), 132.5 (C), 129.0 (CH), 128.22 (CH), 128.19 (CH), 122.5 (C), 120.2 (q, *J* = 323.3 Hz, C), 56.3 (CH), 48.0 (CH_2), 36.3 (CH_2), 31.2 (CH_2), 30.2 (CH_2), 27.7 (CH_2), 27.5 (CH_2), 26.6 (CH_2), 18.7 (CH_3). **HRMS** (APCI, $[M+H]^+$) *m/z* calculated for $C_{18}H_{22}ClF_3NO_2S$: 408.1006; found 408.0997. **Rf** = 0.66 (hexane:Et_2O 80:20). **MP** = 89.5-91.2^{o}C.

Enantioselectivity of the cycloadduct product was determined by chiral HPLC analysis on Chiralpak IA3 at rt (Hexane, 0.3 mL/min, λ=220 nm).

Racemic sample (*rac*-**8ba**)

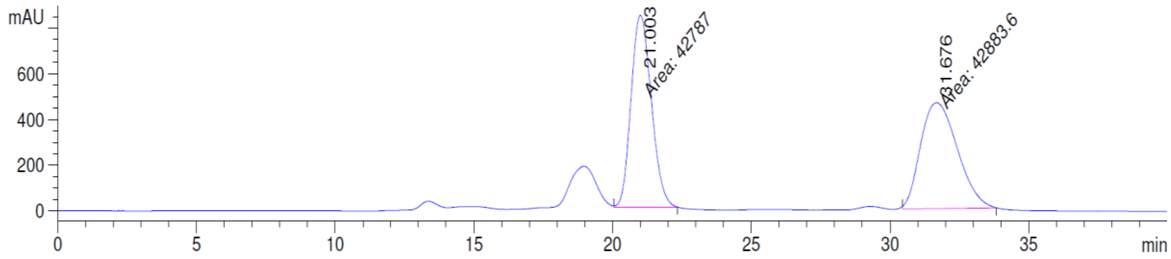

Signal 1: DAD1 A, Sig=220,8 Ref=360,100

Peak #	RetTime [min]	Type	Width [min]	Area [mAU*s]	Height [mAU]	Area %
1	21.003	MM	0.8500	4.27870e4	838.98834	49.9436
2	31.676	MM	1.5419	4.28836e4	463.55023	50.0564

Totals : 8.56706e4 1302.53857

Asymmetric sample (**8ba**, 92.9:7.1 *er*, 86% *ee*)

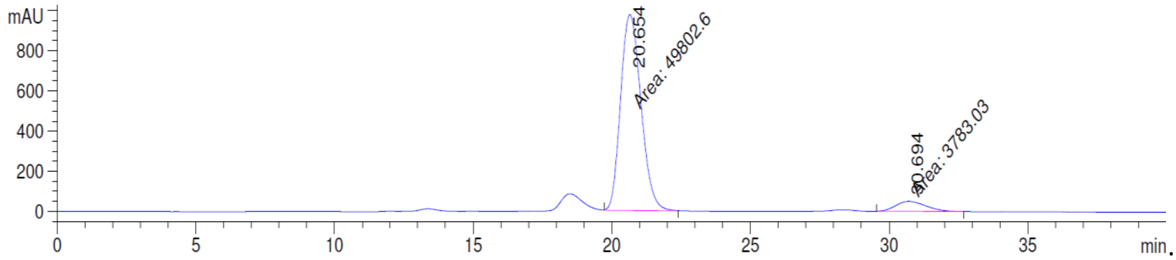

Signal 1: DAD1 A, Sig=220,8 Ref=360,100

Peak #	RetTime [min]	Type	Width [min]	Area [mAU*s]	Height [mAU]	Area %
1	20.654	MM	0.8519	4.98026e4	974.38898	92.9402
2	30.694	MM	1.2615	3783.03369	49.98244	7.0598

Totals : 5.35856e4 1024.37142

Enantioselectivity of the remaining starting material was determined by chiral HPLC analysis on Chiralpak IB at rt (Hexane : *i*PrOH = 99:1, 0.5 mL/min, λ=220 nm)

Racemic sample (*rac*-**7b**)

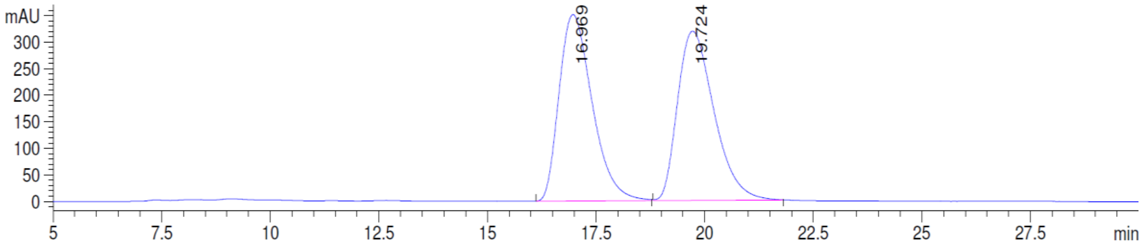

Signal 1: DAD1 A, Sig=220,8 Ref=360,100

Peak #	RetTime [min]	Type	Width [min]	Area [mAU*s]	Height [mAU]	Area %
1	16.969	BB	0.8164	1.86904e4	351.46616	50.0257
2	19.724	BB	0.9004	1.86712e4	319.23615	49.9743

Totals : 3.73617e4 670.70230

Asymmetric sample (**7b**, 95.8:4.2 *er*, 92% *ee*)

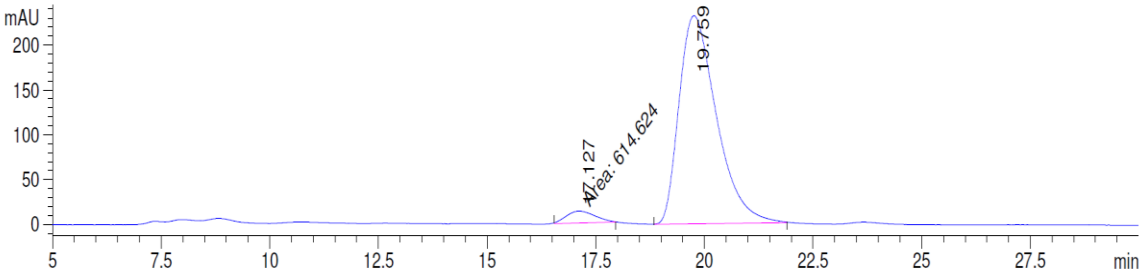

Signal 1: DAD1 A, Sig=220,8 Ref=360,100

Peak #	RetTime [min]	Type	Width [min]	Area [mAU*s]	Height [mAU]	Area %
1	17.127	MM	0.7704	614.62427	13.29672	4.2368
2	19.759	PB	0.9112	1.38920e4	231.80692	95.7632

Totals : 1.45066e4 245.10364

7. Computational data

7.1. Computational details

All calculations were carried out at density functional theory (DFT) level using the M06 density functional[9] in Gaussian 16.[10] The effective core potential LANL2DZ together with its associated double-ζ basis set was used for Pd and Cs; the 6-31+G(d,p) basis set was used O and F; the 6-31G(d,p) basis set was used for S, N, C, and H (BS-1).[11-13] Geometry optimizations were performed in toluene solvent using the continuum SMD model.[14] Analytical frequency calculations were computed to confirm maxima (transition states, one imaginary frequency). Gibbs energies were computed at 373 K and 1 M. All frequencies below 50 cm^{-1} were replaced by 50 cm^{-1} when computing vibrational partition functions[15] with the Goodvibes script.[16] Single point calculations were performed on previous optimized geometries using the larger basis set 6-311+G(d,p) for all non-heavy atoms (BS-2).[17-19] Final Gibbs energies are obtained as follows:

$$\Delta G = \Delta E(BS\text{-}2) + [\Delta G(BS\text{-}1) - \Delta E(BS\text{-}1)]$$

All inputs and outputs are freely available from the open access platform ioChem-BD[20] under the following DOI: 10.19061/iochem-bd-6-155 or the link provided in the reference 16 of the manuscript.

7.2. Additional schemes and structural information

The DFT-optimized transition states structures (TS) for for the formation of R and S enantiomers of Ac-Val were obtained starting with the topologies **D** (downwards) and **U** (upwards) as explained in the main text and reported in ref. 5a. For each one, we computed the *R* and *S* enantiomers, which yields overall 4 initial configurations: DR, DS, UR, US. In DR and DS TSs, the co-planar *ortho* C-H bond (to be activated) points below the Pd coordination plane (downwards). Whereas in US and UR, the *ortho* C-H bond (to be activated) points upwards. DR and UR structures lead to the *R* stereoisomer while DS and US transition states lead to the *S* enantiomer. For each configuration, several conformers were explored by changing the position of Cs^+ and by systematically rotating dihedral angles (*e.g.*, Bn group of the substrate, SO_2CF_3 group, ^{i}Pr of Val). Due to the large number of resulting conformers, only the four most stable ones are reported in the main text and labeled as **TS-DR**, **TS-DS**, **TS-UR**, and **TS-US**. The same procedure was followed for Ac-NOBIN.

Scheme S1. Most stable TS structures for the Ac-Val-based catalyst. Relative Gibbs energies in kcal/mol.

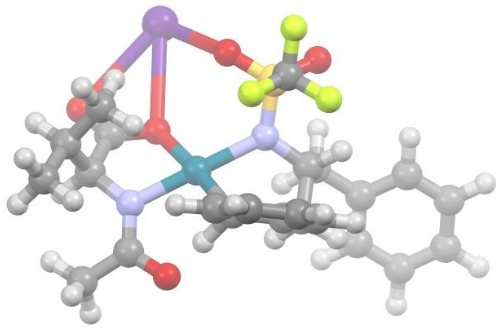

TS-DR (Ac-Val)

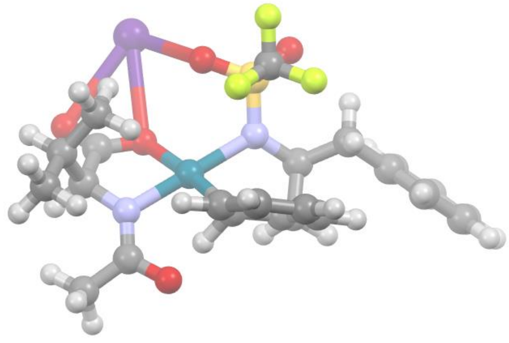

TS-DS (Ac-Val)

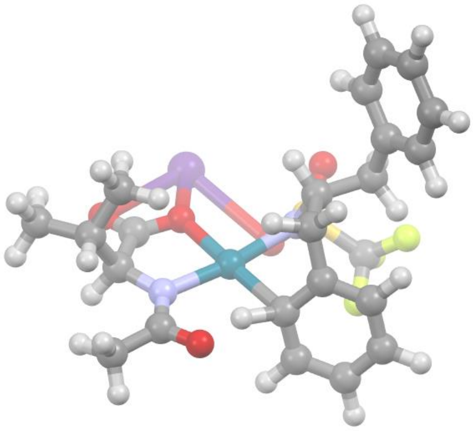

TS-UR (Ac-Val)

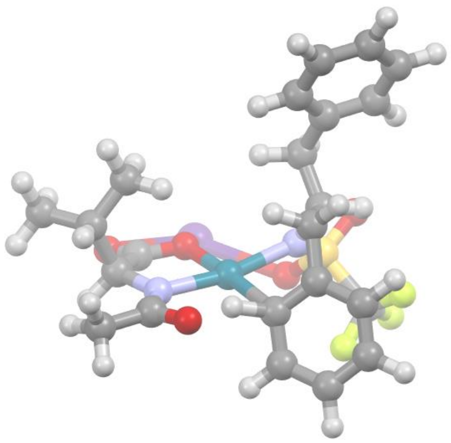

TS-US (Ac-Val)

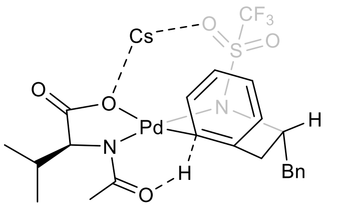

TS-DR 0.0

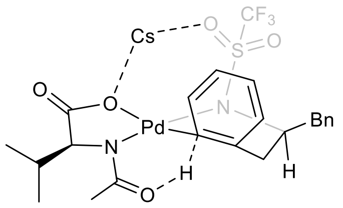

TS-DS 8.7

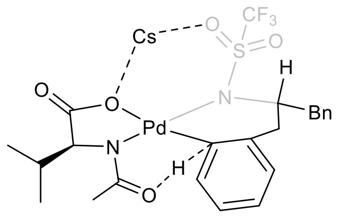

TS-UR 13.7

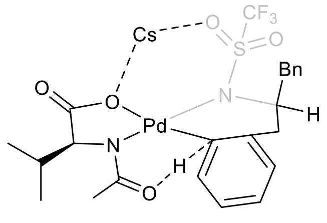

TS-US 4.8

Scheme S2. Most stable TS structures for the Ac-NOBIN-based catalyst. Relative Gibbs energies in kcal/mol.

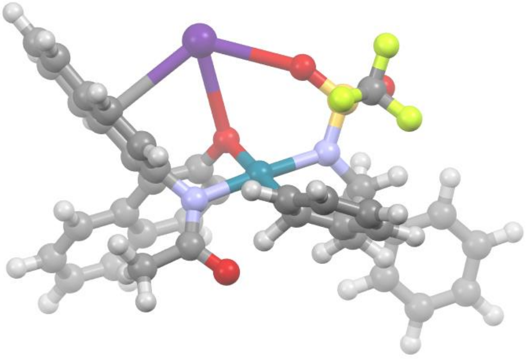

TS-DR (Ac-NOBIN)

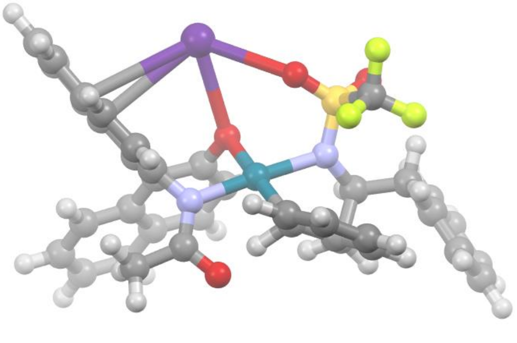

TD-DS (Ac-NOBIN)

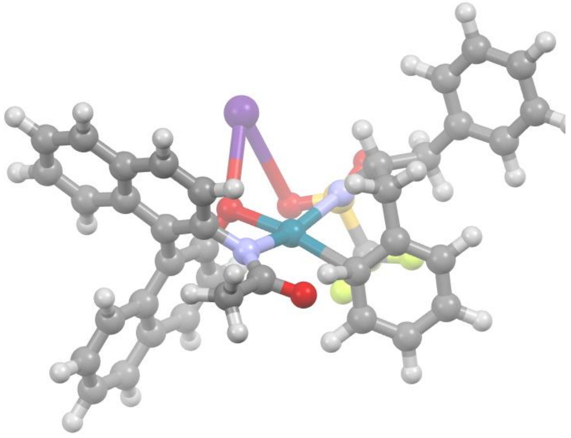

TS-UR (Ac-NOBIN)

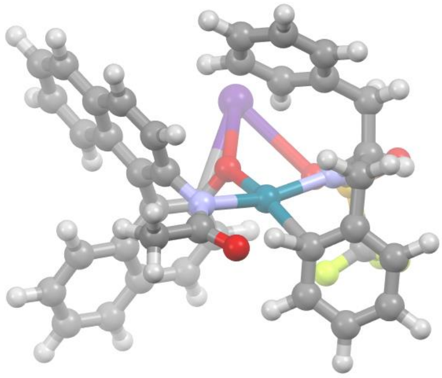

TS-US (Ac-NOBIN)

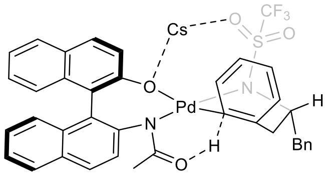

TS-DR 0.0

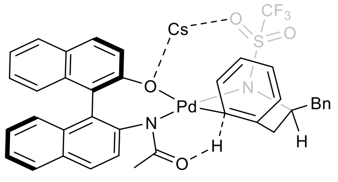

TS-DS 8.2

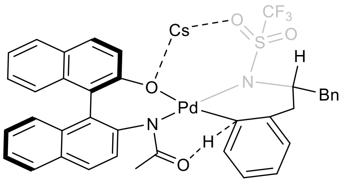

TS-UR 23.9

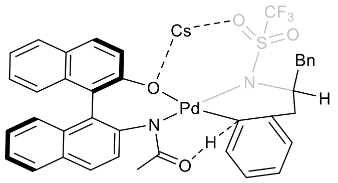

TS-US 12.5

Table S5. Dihedral angle between the atoms forming the square-planar environment (N-O-N-C) for each TS structure. Ideal value = 0 °.

Isomer	φ (NOBIN-Pd) / °	ΔG / kcal·mol^{-1}	φ (Val-Pd) / °	ΔG / kcal·mol^{-1}
**DR**	0.7	0.0	−3.8	0.0
**DS**	4.5	8.2	−0.8	8.7
**UR**	43.7	23.9	7.9	13.7
**US**	24.9	12.5	12.6	4.8

Scheme S3. NCI plots of **TS-DR** for Ac-NOBIN- and Ac-Val-derived ligands.

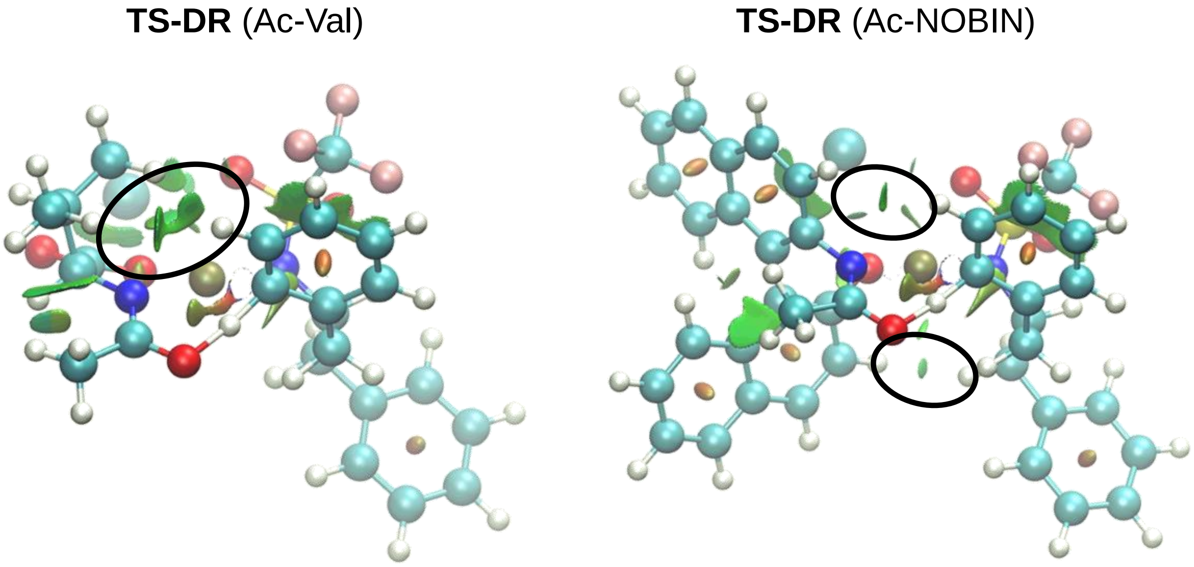

Scheme S3 displays the non-covalent interactions (NCI) plots[22, 23] of **TS-DR** (the most stable TS) for each ligand. The thin green surfaces represent van der Waals interactions. The specific interactions between the ligand and the substrate are highlighted within black circles. On the one hand, for Ac-Val, the Bn group that is being activated clearly interacts with the ^{i}Pr group. On the other hand, for Ac-NOBIN, there exist two interactions between the naphthyl moiety and the substrate, although they seem smaller than those for Ac-Val.

8. References

(1) Chang, X.; Zhang, Q.; Guo, C. *Org. Lett.* **2019**, *21*, 4915–4918.

(2) Belokon, Y. N.; Bespalova, N. B.; Churkina, T. D.; Císařová, I.; Ezernitskaya, M. G.; Harutyunyan, S. R.; Hrdina, R.; Kagan, H. B.; Kočovský, P.; Kochetkov, K. A.; Larionov, O. V.; Lyssenko, K. A.; North, M.; Polášek, M.; Peregudov, A. S.; Prisyazhnyuk, V. V.; Vyskočil, Š. *J. Am. Chem. Soc.* **2003**, *125*, 12860–12871.

(3) Takishima, R.; Nishii, Y.; Miura, M. *Org. Lett*. **2021**, *23*, 4, 1349–1354.

(4) (a) Velasco-Rubio, Á.; Varela, J. A.; Saá, C. *Org. Lett.* **2020**, *22*, 3591–3595. (b) Shi, H.; Herron, A. N.; Shao, Y.; Shao, Q.; Yu, J.-Q. *Nature* **2018**, *558*, 581–586.

(5) S. Levinger, S, R. Nair, A. Hassner, *Beilstein J. Org. Chem.* **2008**, *4*, 1.

(6) a) Kippo, T.; Fukuyama, T.; Ryu, I. *Org. Lett.* **2011**, *13*, 3864–3867. b) Zhao, Z.; Racicot, L.; Murphy, G. K. *Angew. Chem. Int. Ed.* **2017**, *56*, 11620–11623. c) Li, C.; Yang, Z.; Wang, L.; Guo, Y.; Huang, Z.; Ma, S. *Angew. Chem. Int. Ed.* **2020**, *59*, 6278–6283

(7) (a) González, J. M.; Cendón, B.; Mascareñas, J. L.; Gulías, M. *J. Am. Chem. Soc.* **2021**, *143*, 3747–3752. (b) Casanova, N.; Del Río, K. P.; García-Fandiño, R.; Mascareñas, J. L.; Gulías, M. *ACS Catal.* **2016**, *6*, 3349-3353.

(8) Shao, Q.; Wu, Q.-F.; He, J.; Yu, J.-Q. *J. Am. Chem. Soc.* **2018**, *140*, 5322-5325.

(9) Fernández, D. F.; Gulías, M.; Mascareñas, J. L.; López, F. *Angew. Chem. Int. Ed.* **2017**, *56*, 9541-9545.

(10) Zhao, Y.; Truhlar, D. G. *Theor. Chem. Acc.* **2008**, *120*, 215-241.

(11) Gaussian 16, Revision C.01. Frisch, M. J. et al. Gaussian Inc., Wallingford CT, **2016**.

(12) Hay, P. J.; Wadt, W. R. *J. Chem. Phys.* **1985**, *82*, 299-310.

(13) Francl, M. M.; Pietro W. J., Hehre, W. J.; Binkley, J. S.; Gordon, M. S.; DeFrees, D. J.; Pople, J. A. *J. Chem. Phys.* **1982**, *77*, 3654-3665.

(14) Hariharan, P. C.; Pople, J. A. *Theor. Chim. Acta* **1973**, *28*, 213-222.

(15) Marenich, A. V.; Cramer, C. J.; Truhlar, D. G. *J. Phys. Chem. B* **2009**, *113*, 6378-6396.

(16) Grimme, S. *Chem. Eur. J.* **2012**, *18*, 9955-9964.

(17) Luchini, G.; Alegre-Requena, J. V.; Funes-Ardoiz, I.; Paton, R. S. *F1000Research* **2020**, *9*, 1-14.

(18) Krishnan, R.; Binkley, J. S.; Seeger, R.; Pople, J. A. *J. Chem. Phys.* **1980**, *72*, 650-654.

(19) McLean, A. D.; Chandler, G. S. *J. Chem. Phys.* **1980**, *72*, 5639-5648.

(20) Clark, T.; Chandrasekhar, J.; Spitznagel, G. W.; Schleyer, P. V. R. *J. Comput. Chem.* **1983**, *4*, 294-301.

(21) Álvarez-Moreno, M.; De Graaf, C.; López, N.; Maseras, F.; Poblet, J. M.; Bo, C. *J. Chem. Inf. Model.* **2015**, *55*, 95-103.

(22) Johnson, E. R.; Keinan, S.; Mori-Sánchez, P.; Contreras-García, J.; Cohen, A. J.; Yang, W. *J. Am. Chem. Soc.* **2010**, *132*, 6498-6506.

(23) Boto, R. A.; Peccati, F.; Laplaza, R.; Quan, C.; Carbone, A.; Piquemal, J.-P.; Maday, Y.; Contreras-García, J. *J. Chem. Theory Comput.* **2020**, *16*, 4150-4158.

9. NMR Spectra

^{1}H NMR (500 MHz, CDCl$_3$)

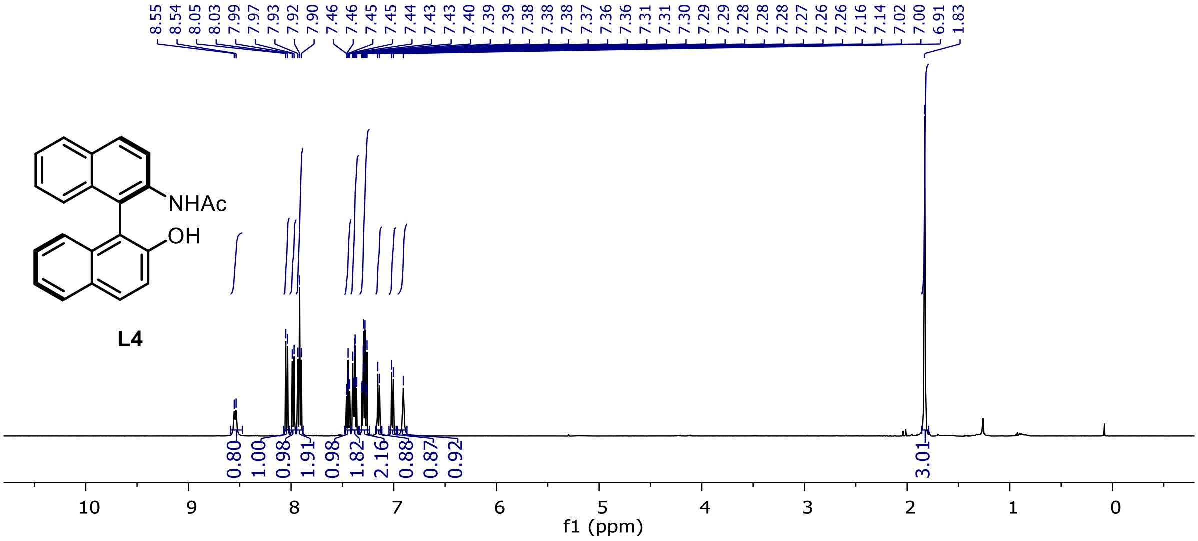

DEPT-135

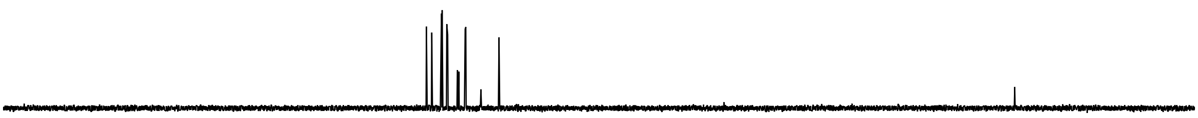

^{13}C NMR (126 MHz, CDCl$_3$)

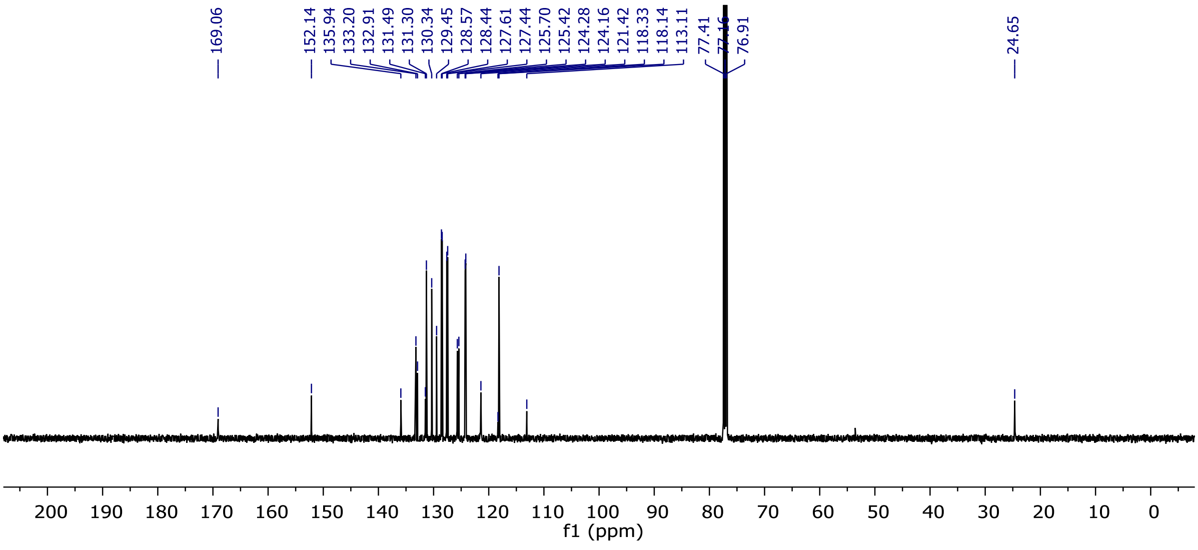

^{1}H NMR (500 MHz, $CDCl_3$)

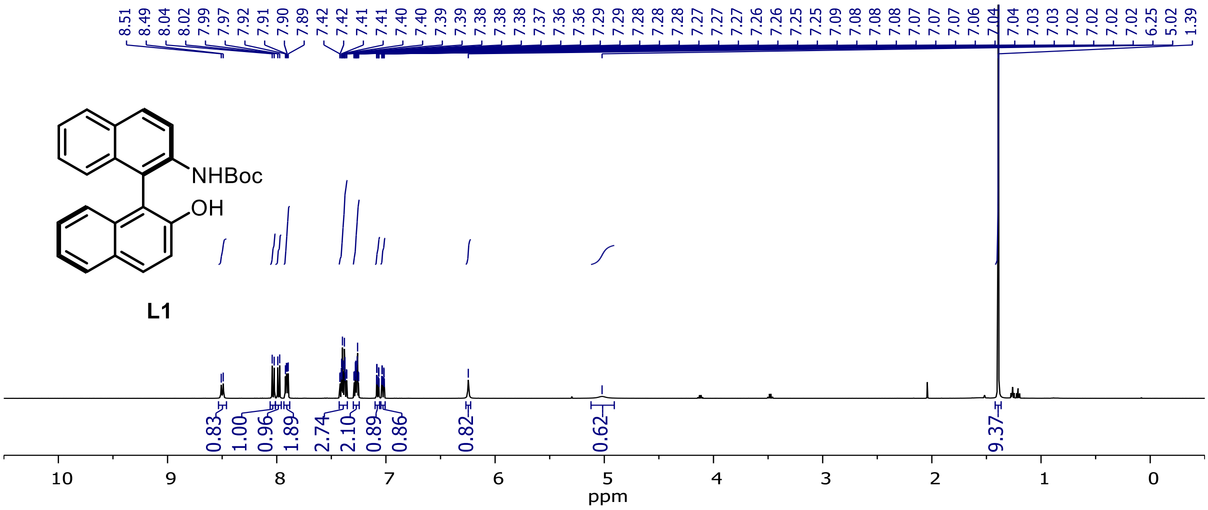

DEPT-135

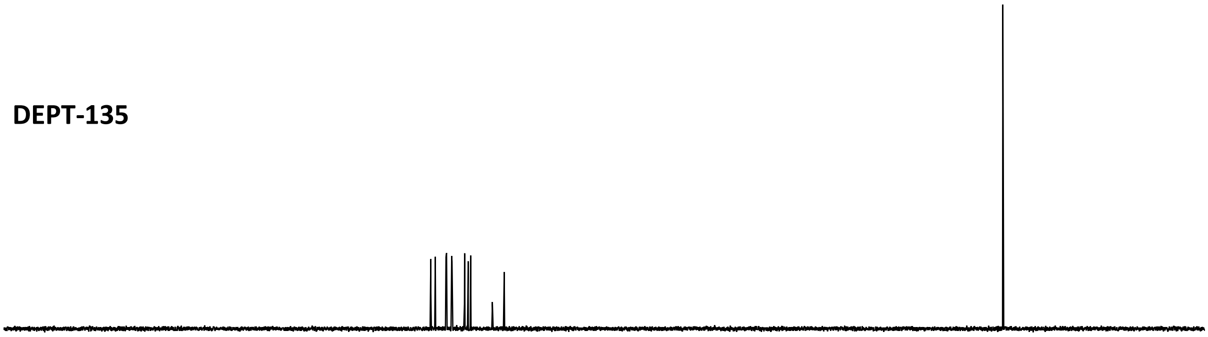

^{13}C NMR (126 MHz, $CDCl_3$)

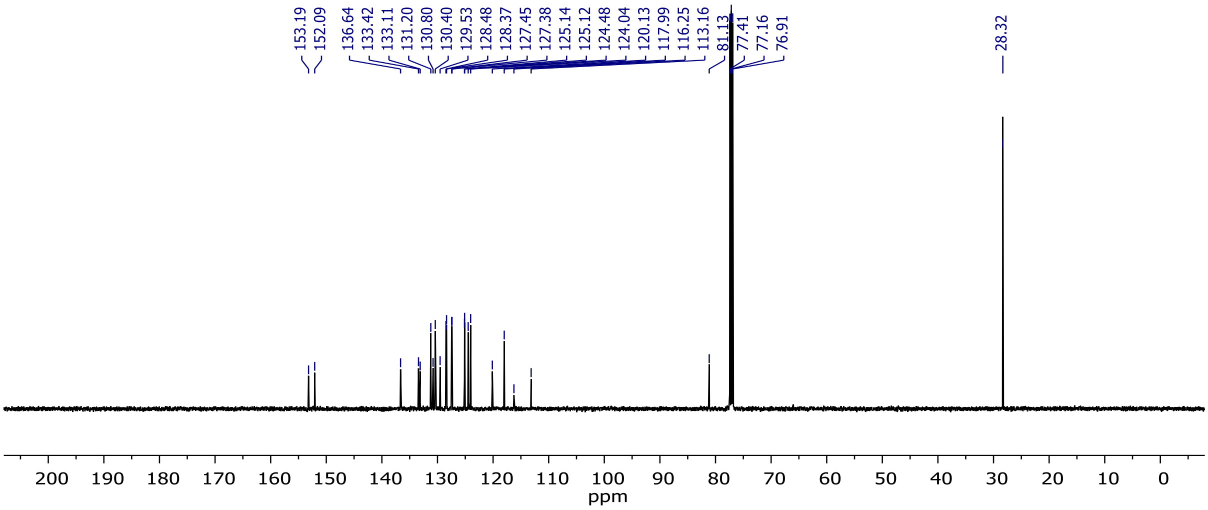

^{1}H NMR (500 MHz, CDCl$_3$)

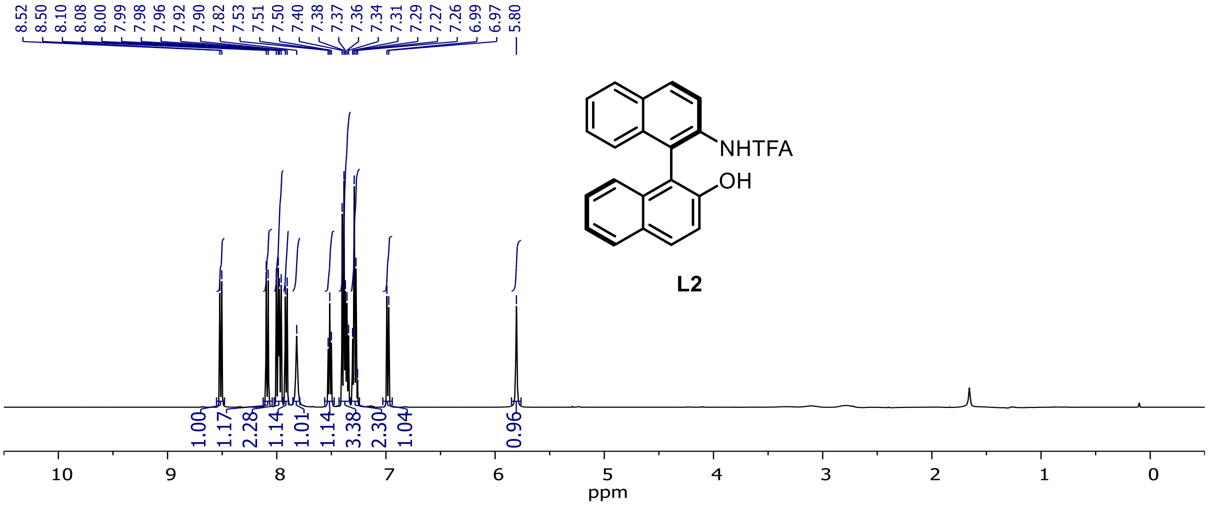

DEPT-135

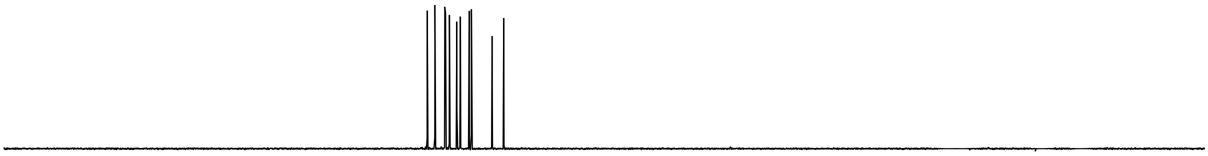

^{13}C NMR (126 MHz, CDCl$_3$)

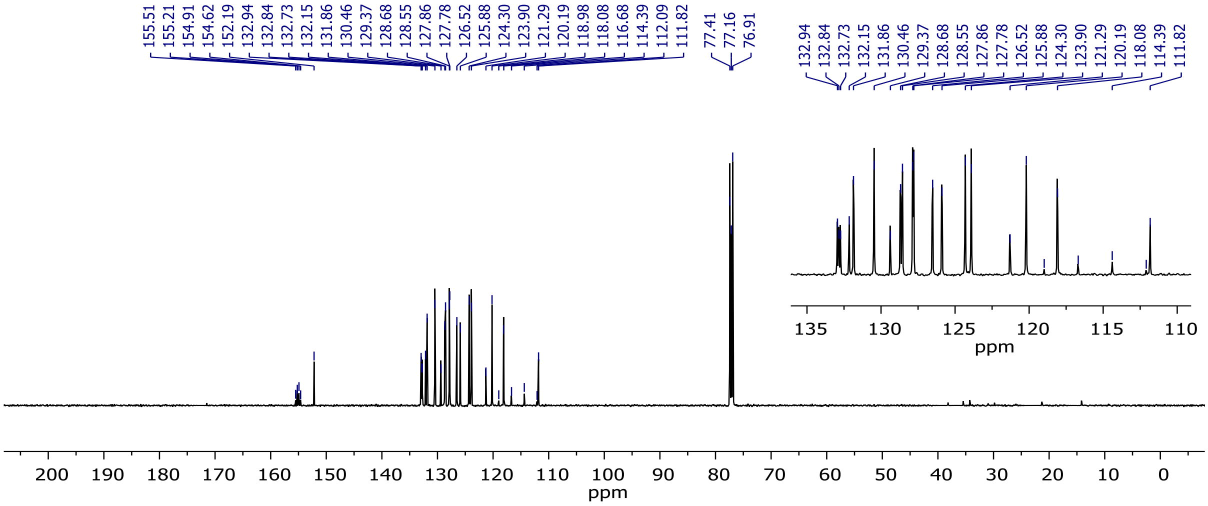

^{1}H NMR (500 MHz, $CDCl_3$)

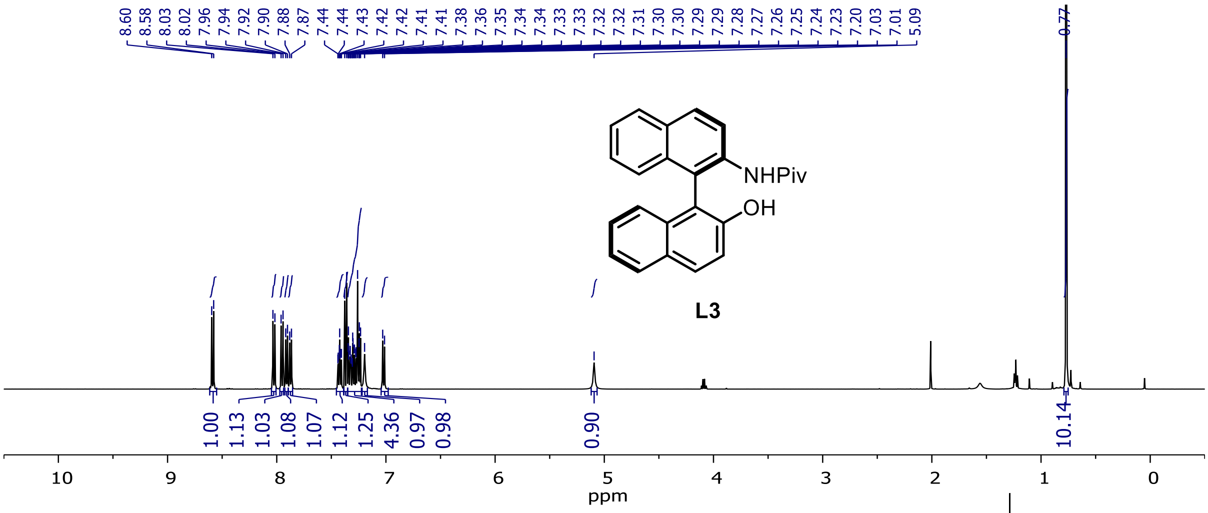

DEPT-135

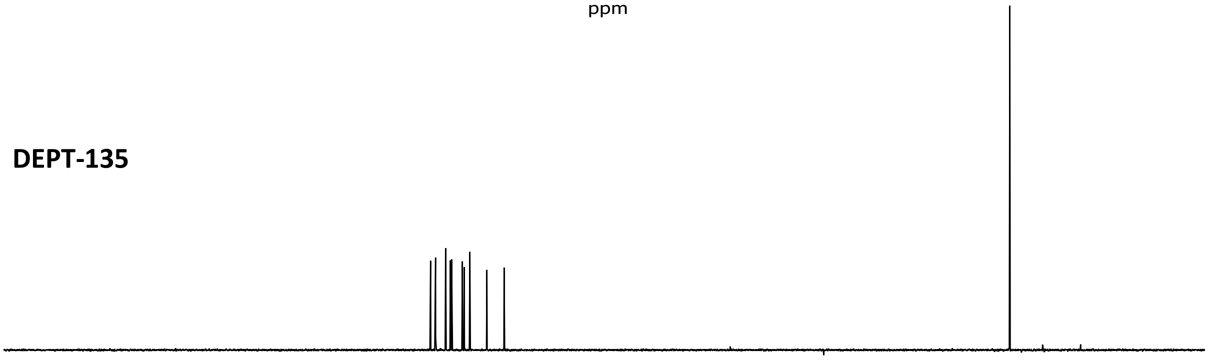

^{13}C NMR (126 MHz, $CDCl_3$)

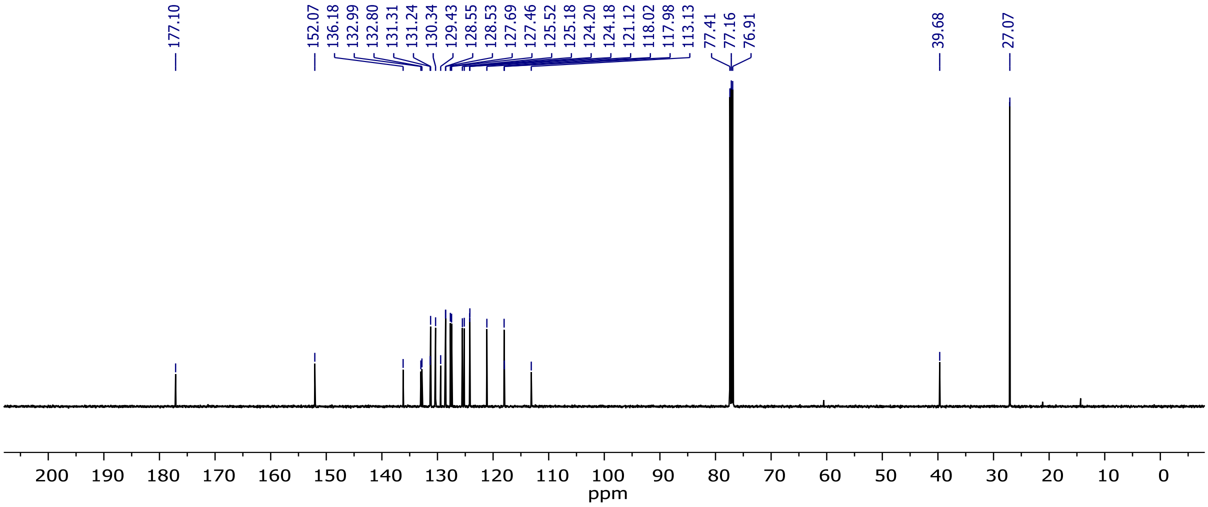

^{1}H NMR (500 MHz, $CDCl_3$)

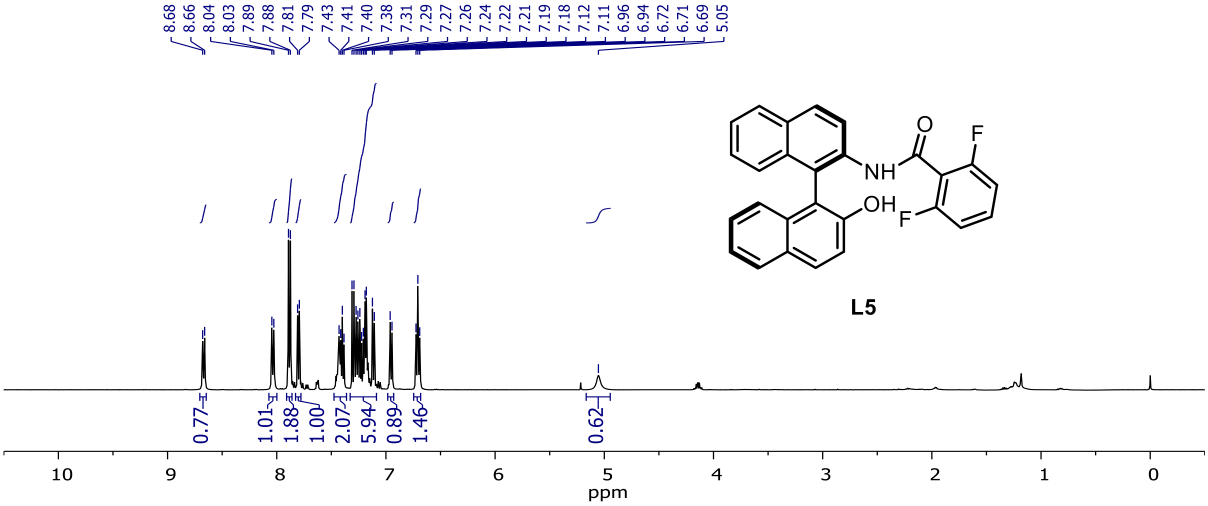

DEPT-135

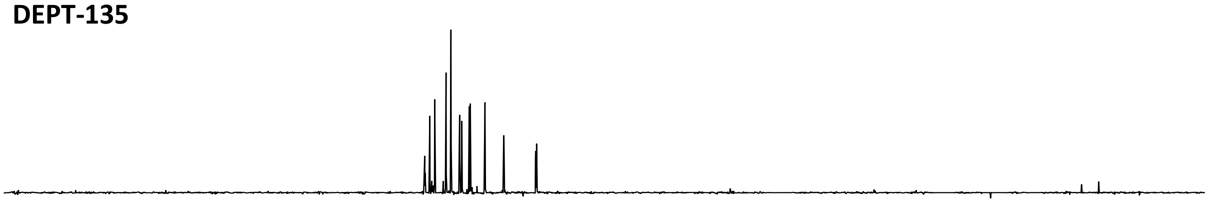

^{13}C NMR (126 MHz, $CDCl_3$)

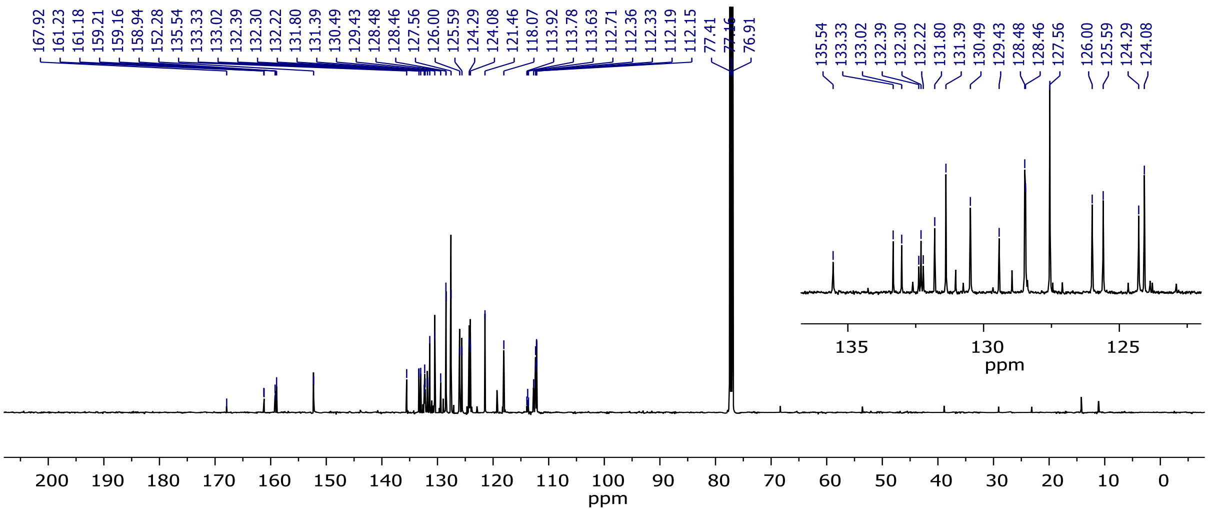

^{1}H NMR (500 MHz, $CDCl_3$)

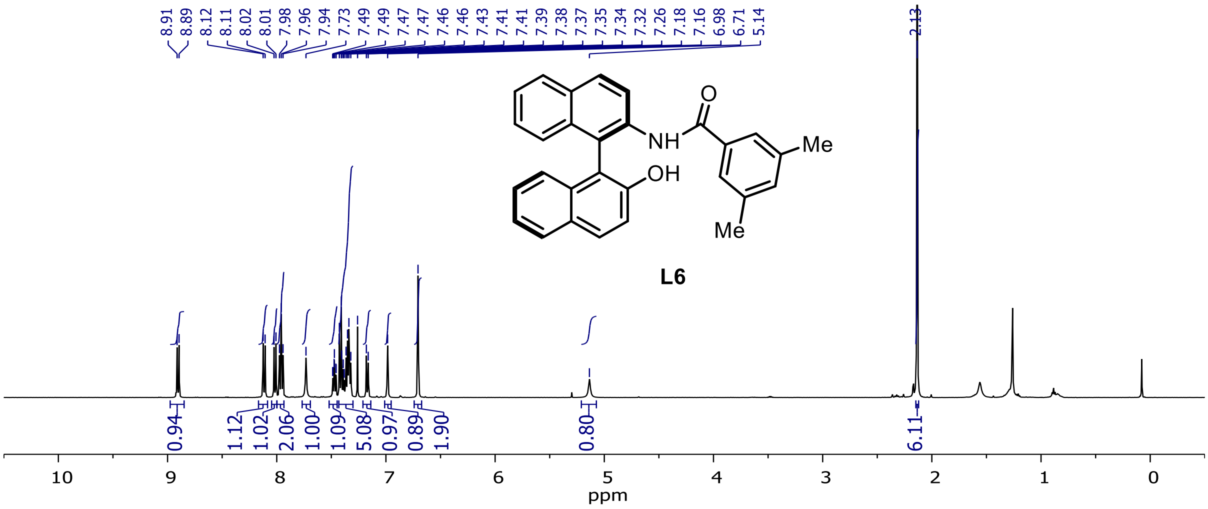

DEPT-135

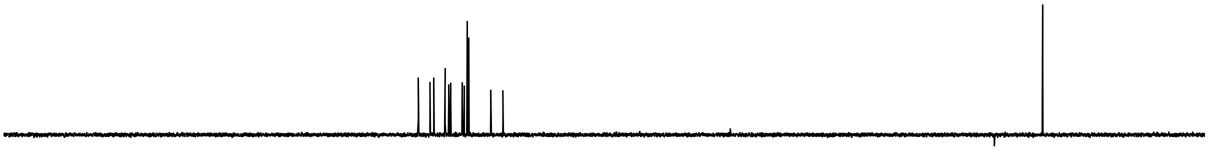

^{13}C NMR (126 MHz, $CDCl_3$)

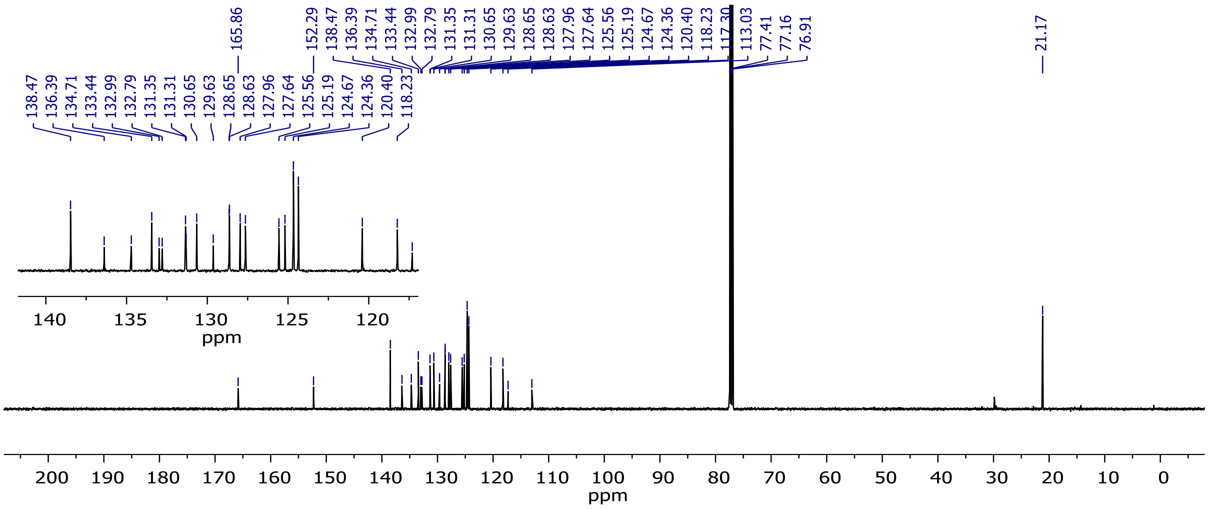

^{1}H NMR (500 MHz, $CDCl_3$)

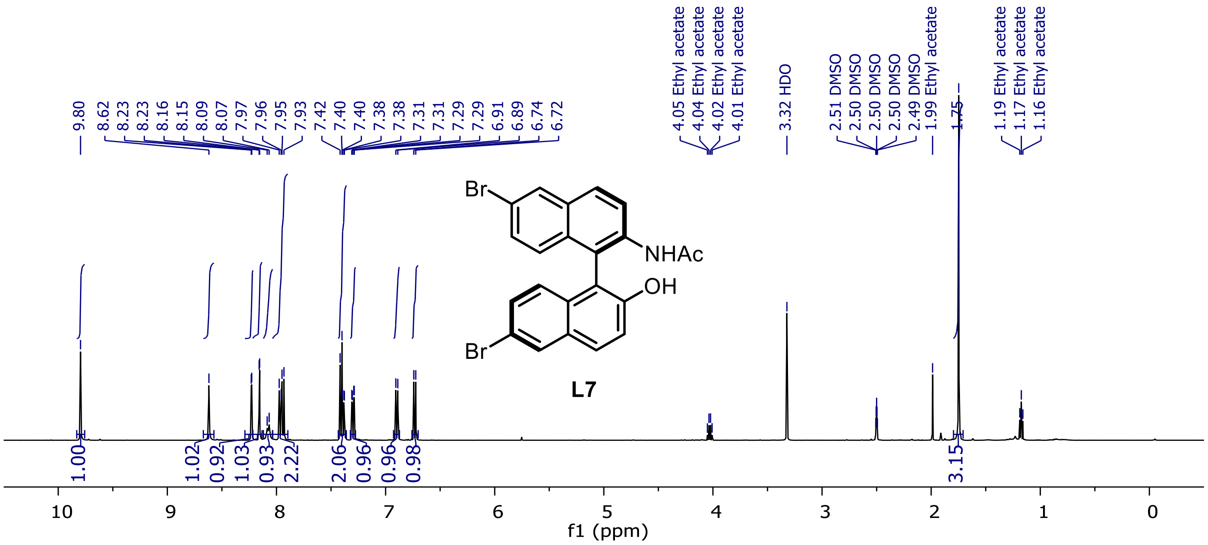

DEPT-135

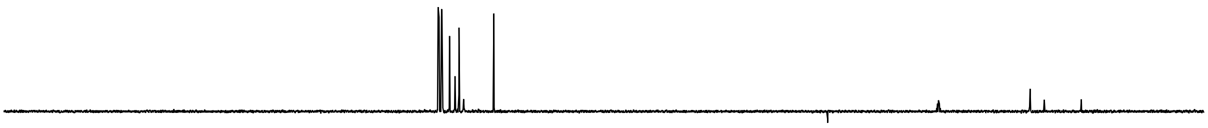

^{13}C NMR (126 MHz, $CDCl_3$)

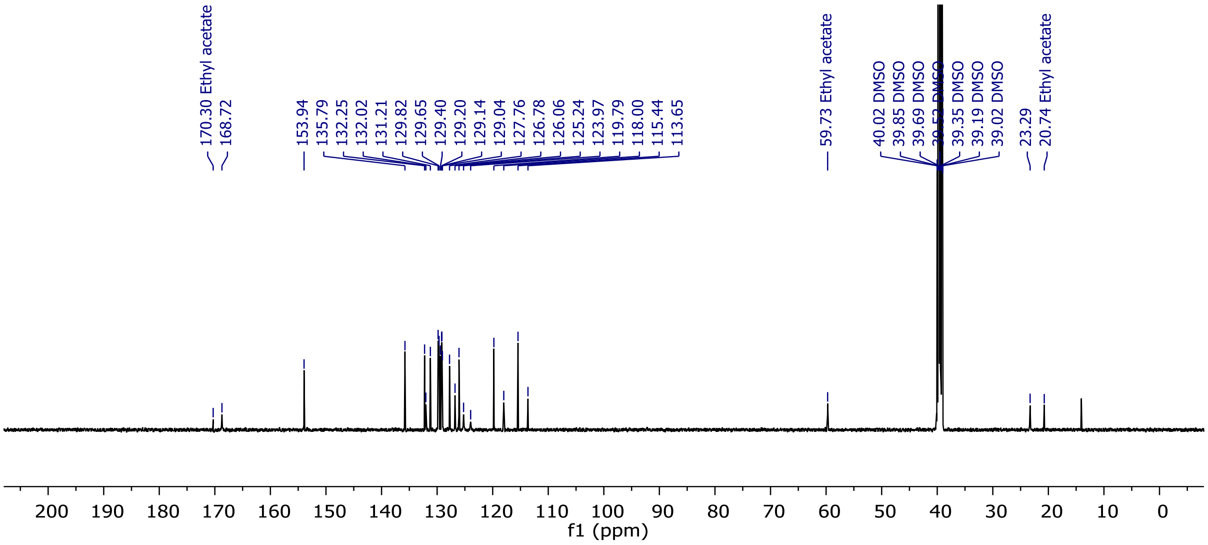

^{1}H NMR (500 MHz, $CDCl_3$)

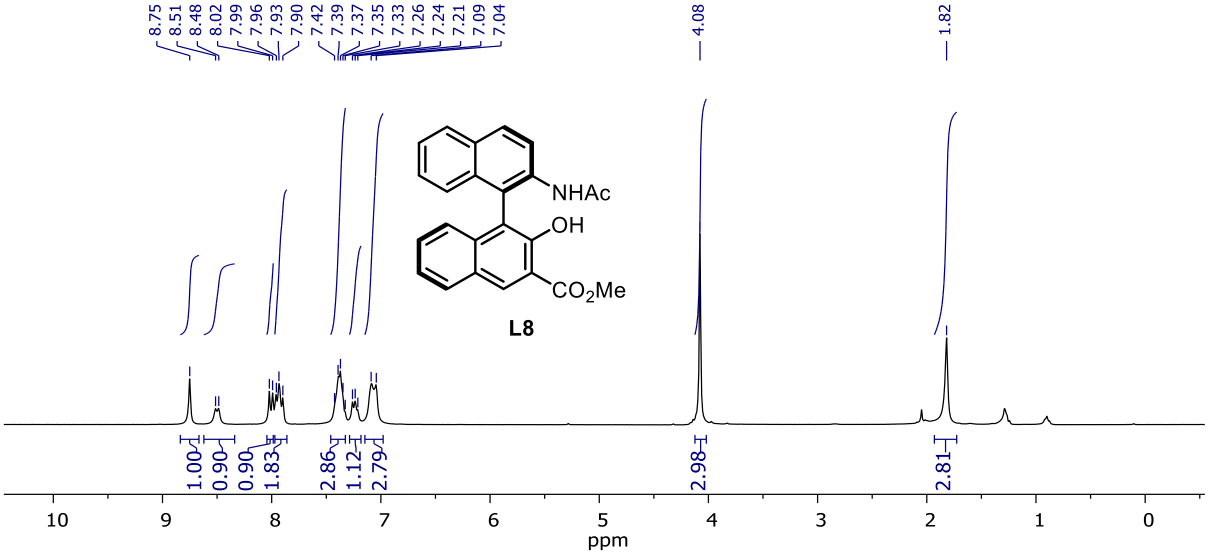

DEPT-135

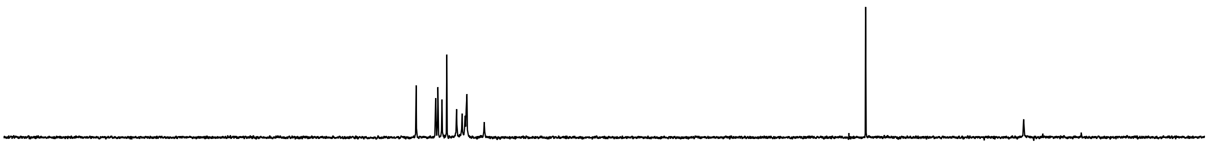

^{13}C NMR (126 MHz, $CDCl_3$)

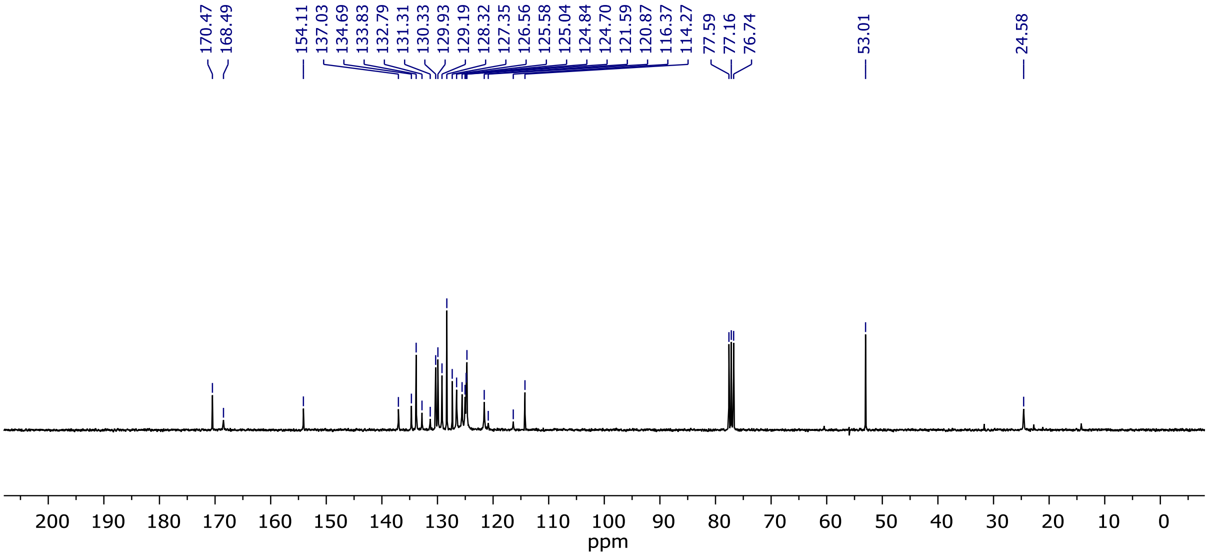

^{1}H NMR (500 MHz, CDCl$_3$)

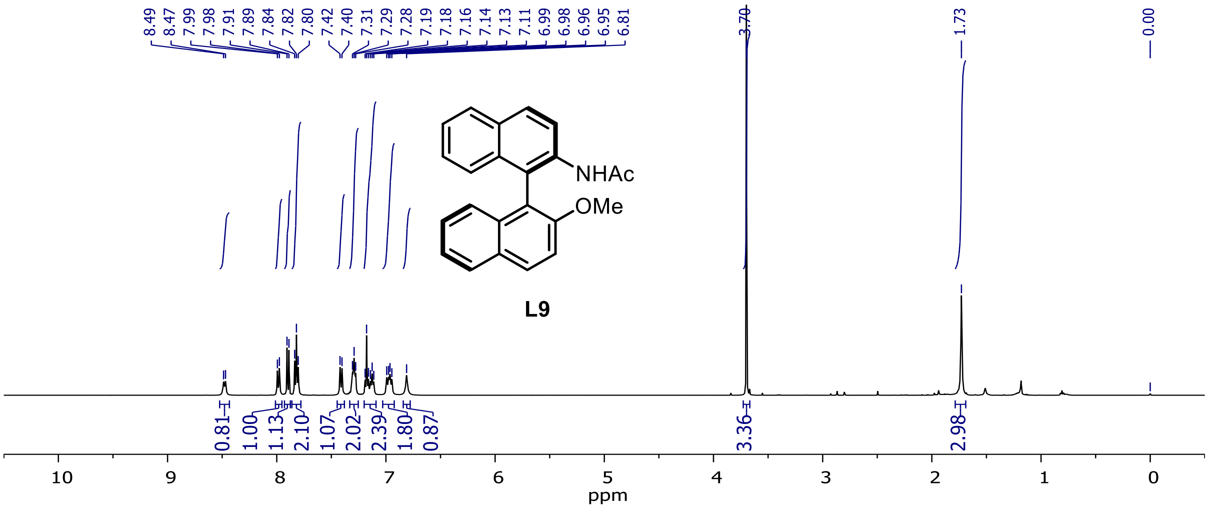

DEPT-135

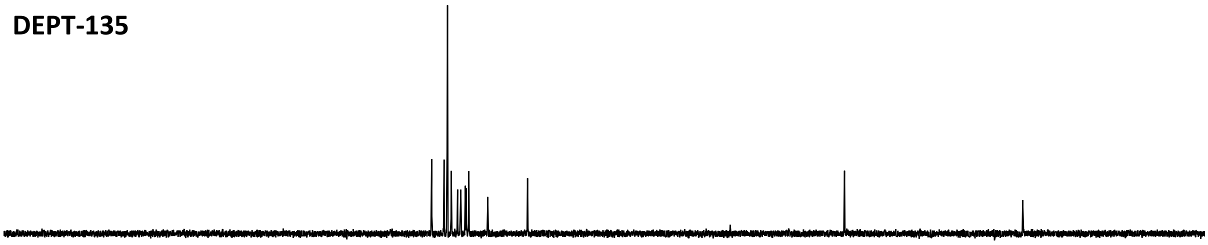

^{13}C NMR (126 MHz, CDCl$_3$)

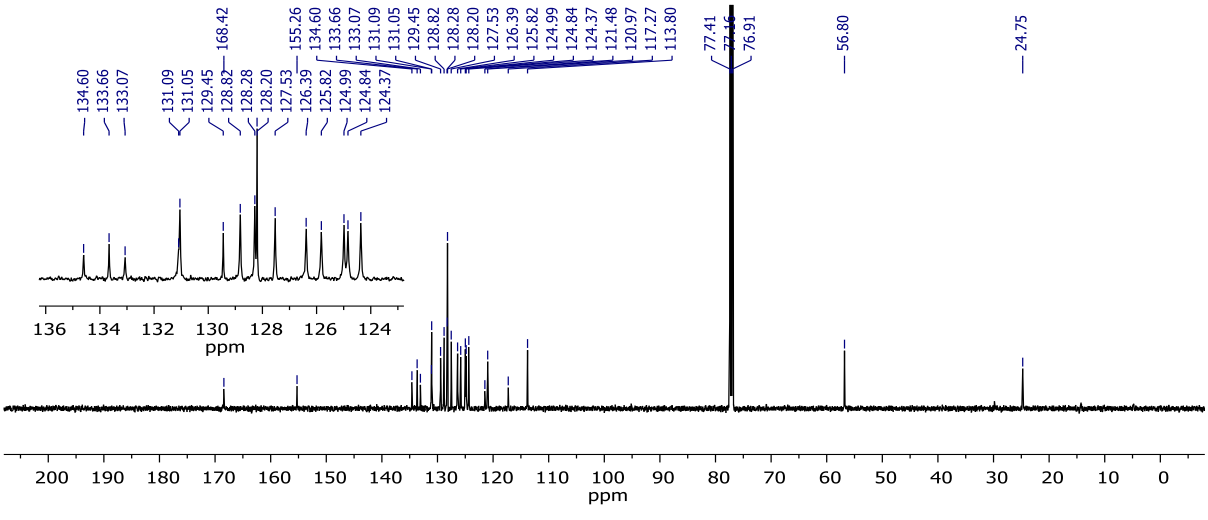

^{1}H NMR (400 MHz, $C_2D_2Cl_4$, 298K)

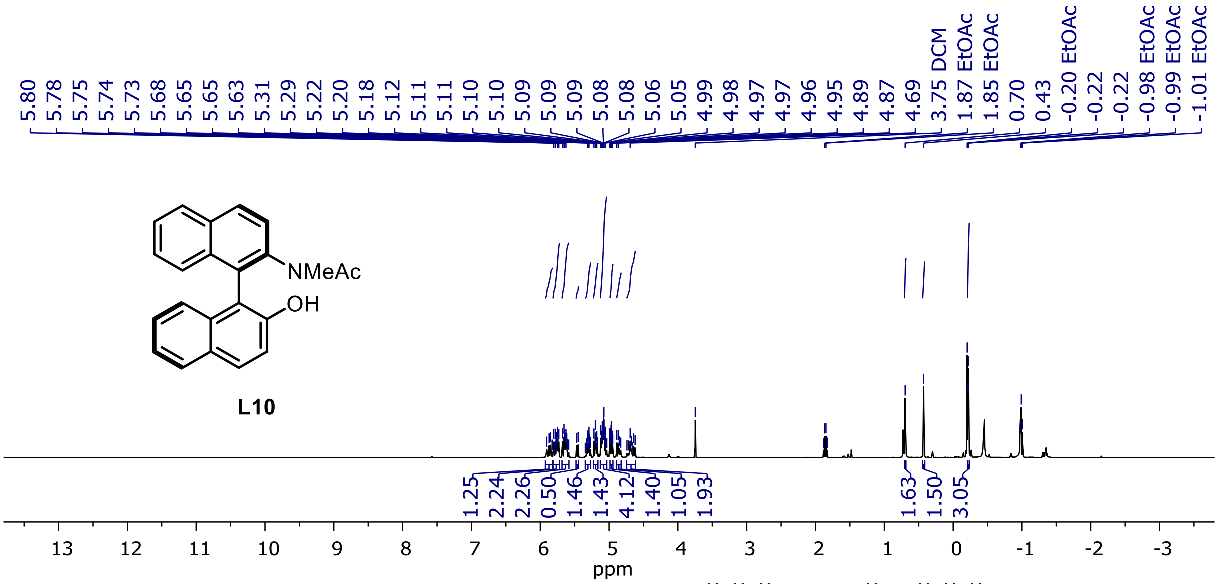

^{1}H NMR (400 MHz, $C_2D_2Cl_4$, 353K)

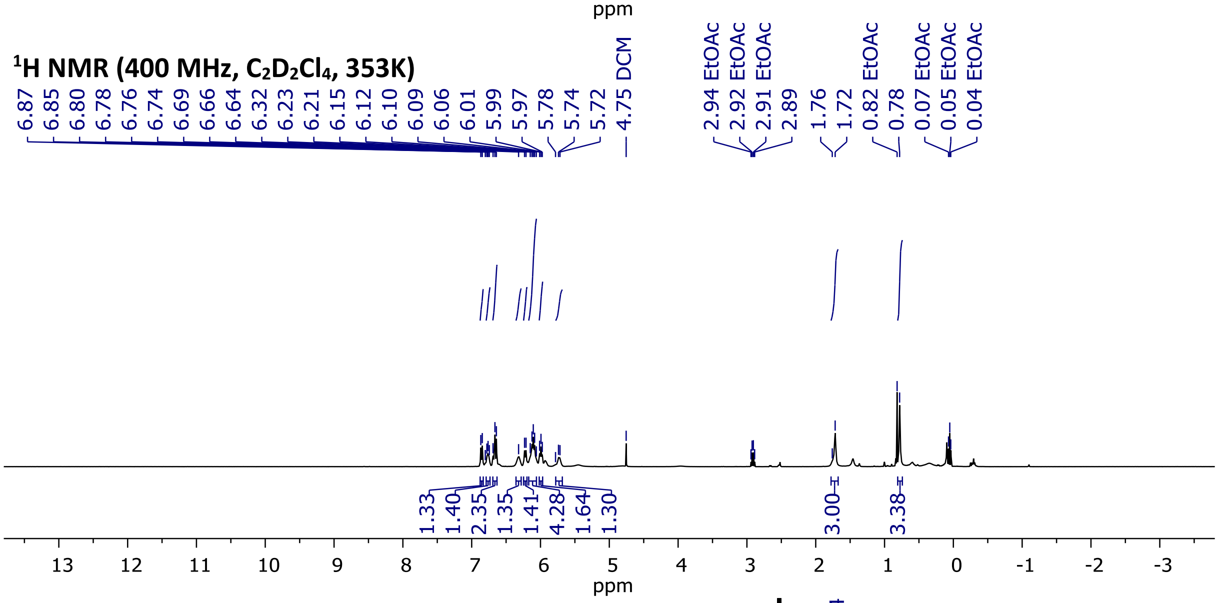

^{13}C NMR (100 MHz, $C_2D_2Cl_4$, 353K)

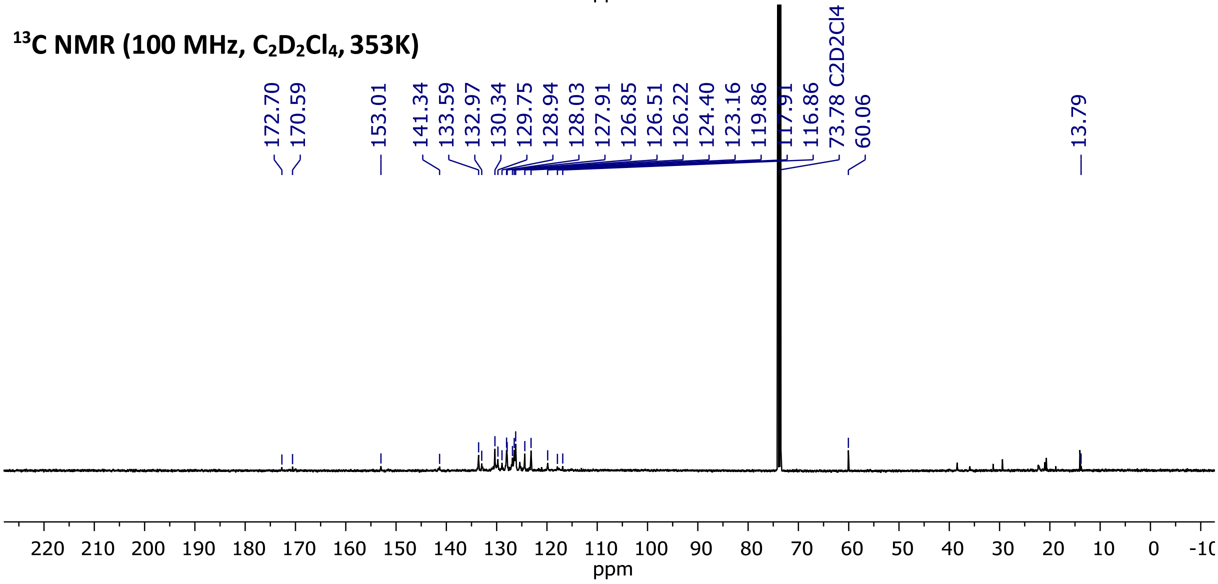

¹H NMR (300 MHz, CDCl₃)

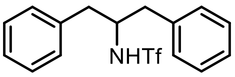

1a

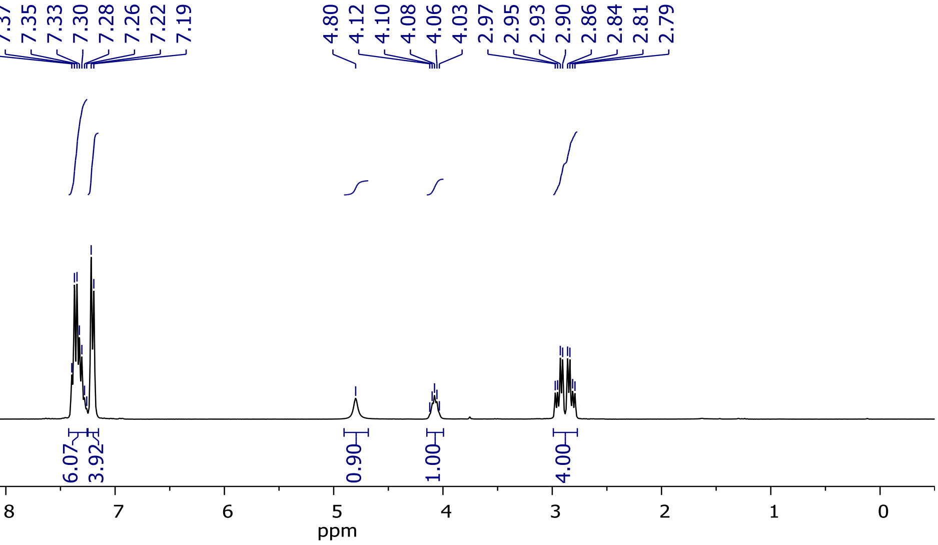

DEPT-135

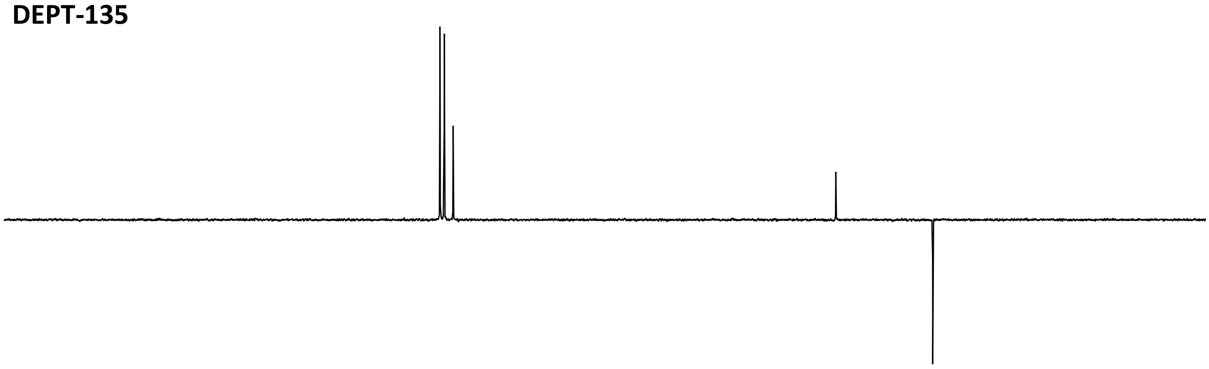

¹³C NMR (75 MHz, CDCl₃)

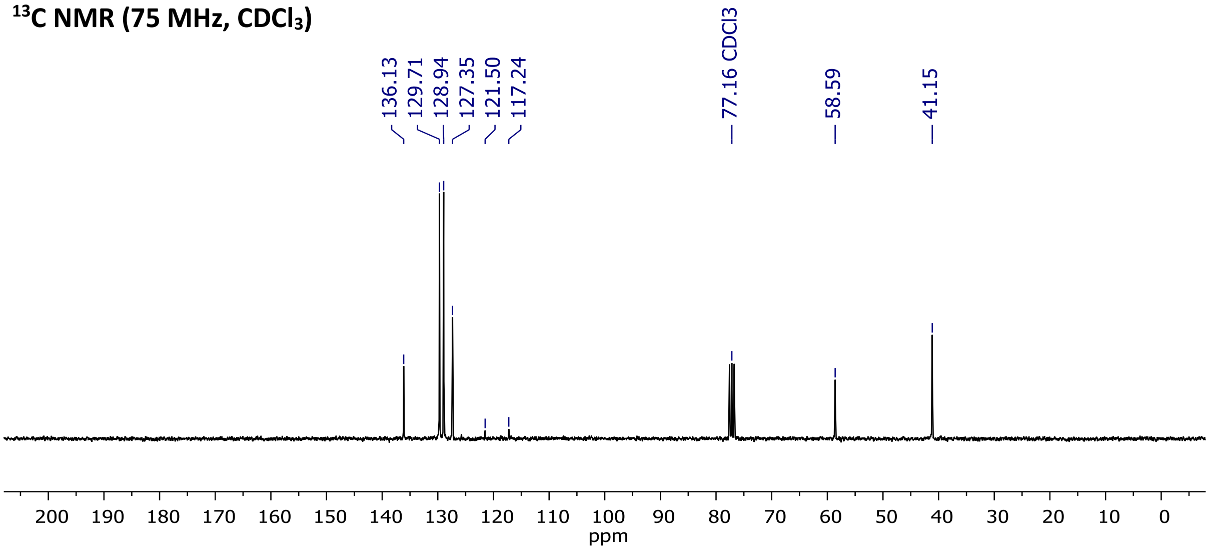

^{1}H NMR (300 MHz, CDCl$_3$)

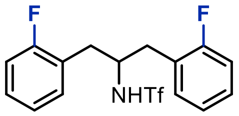

1b

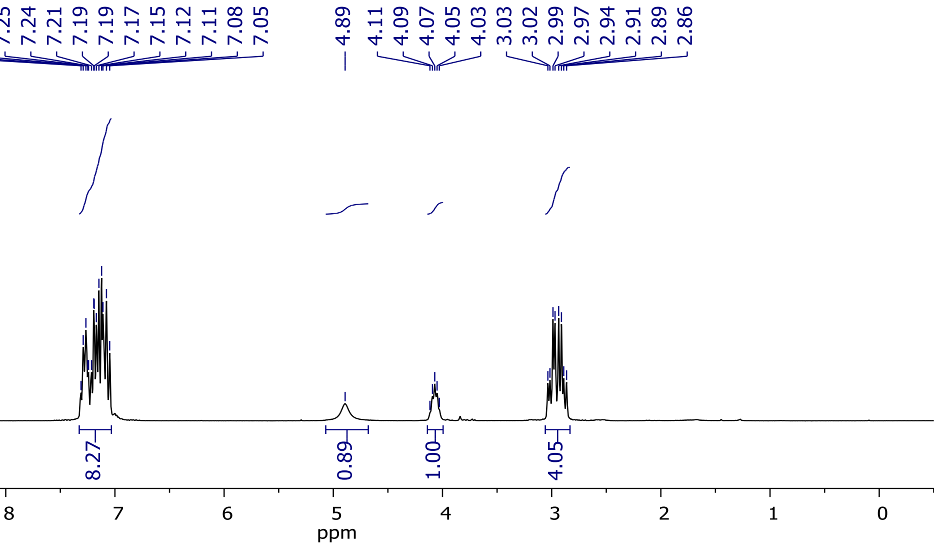

DEPT-135

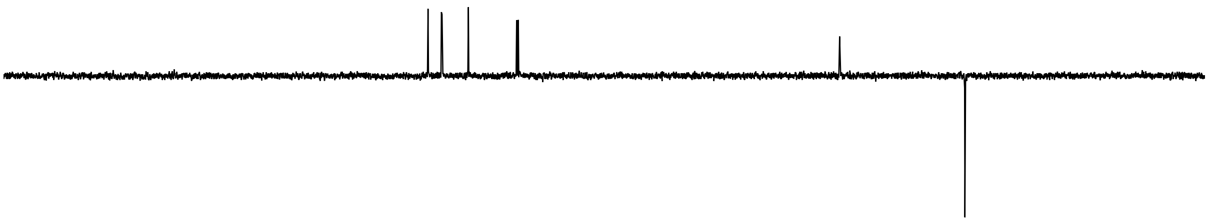

^{13}C NMR (75 MHz, CDCl$_3$)

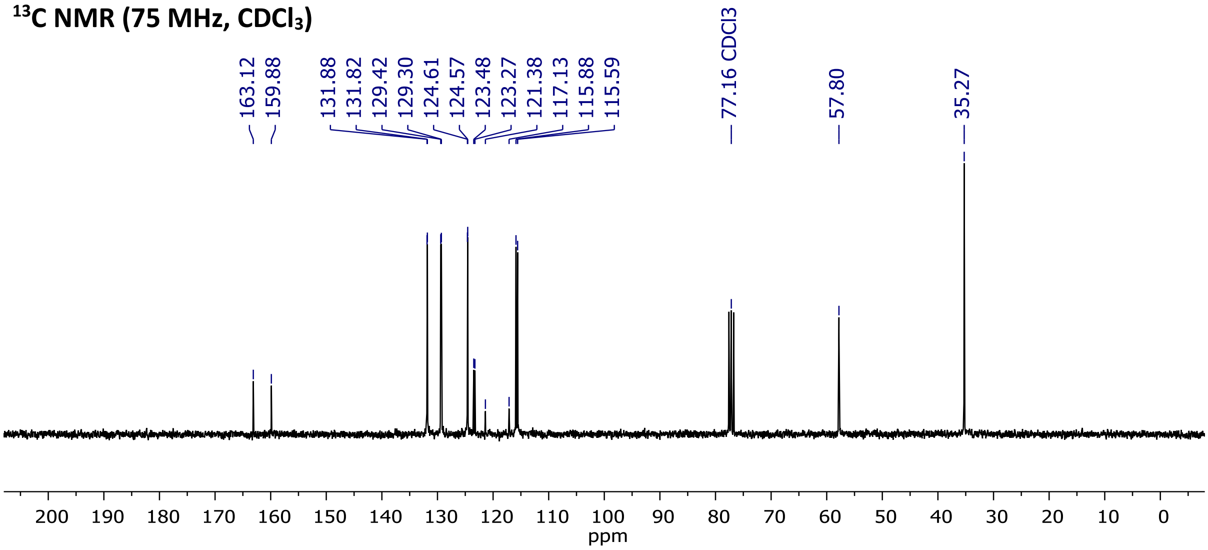

^{1}H NMR (300 MHz, $CDCl_3$)

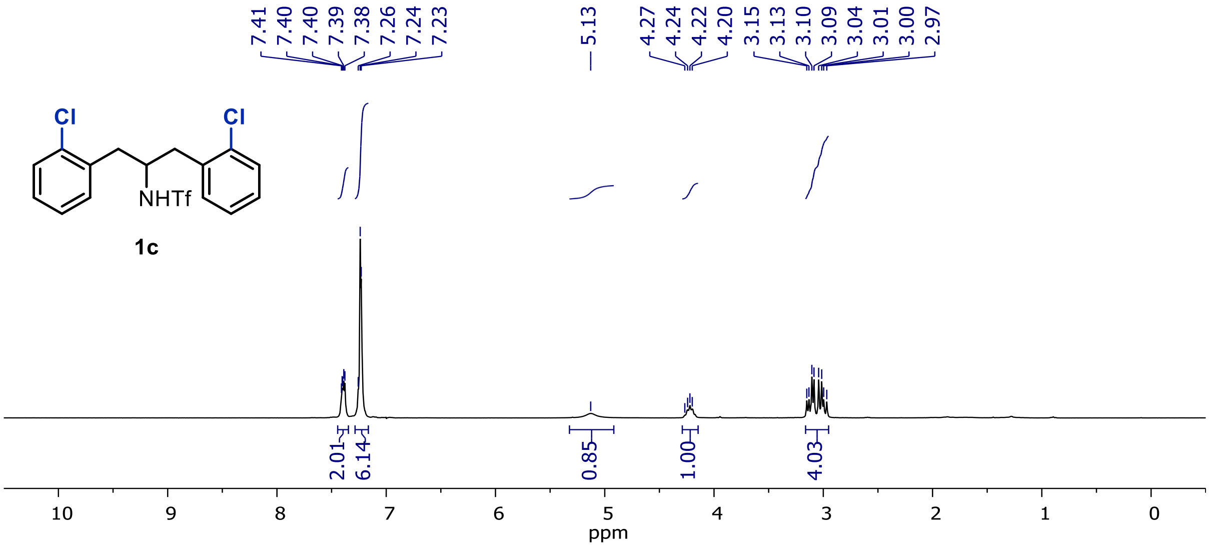

DEPT-135

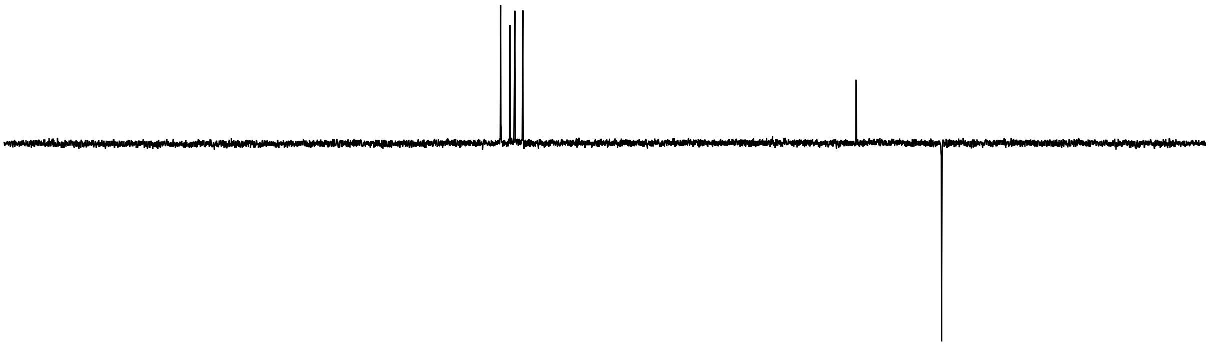

^{13}C NMR (75 MHz, $CDCl_3$)

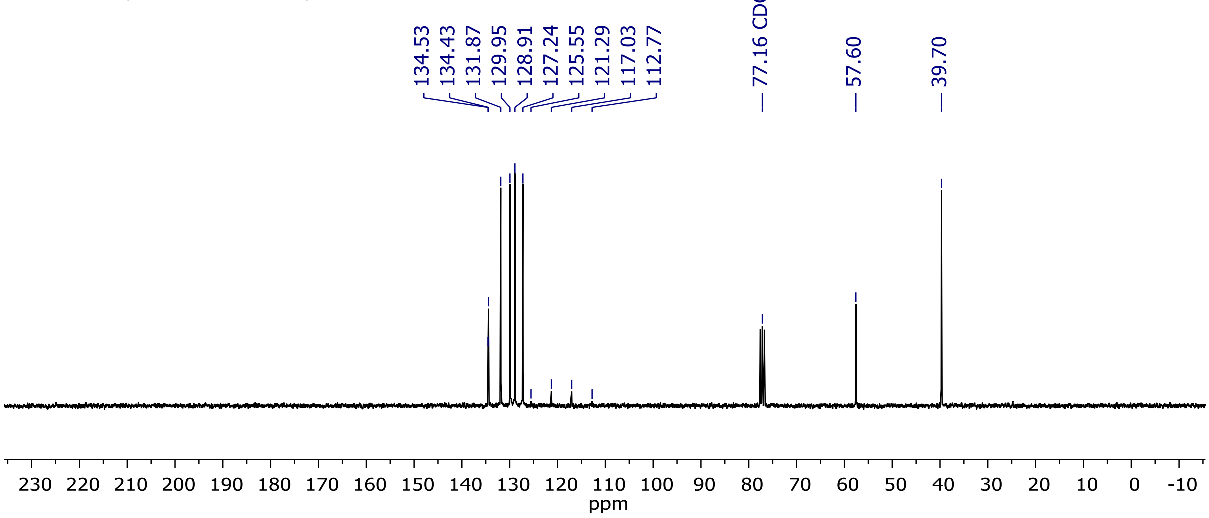

^{1}H NMR (300 MHz, $CDCl_3$)

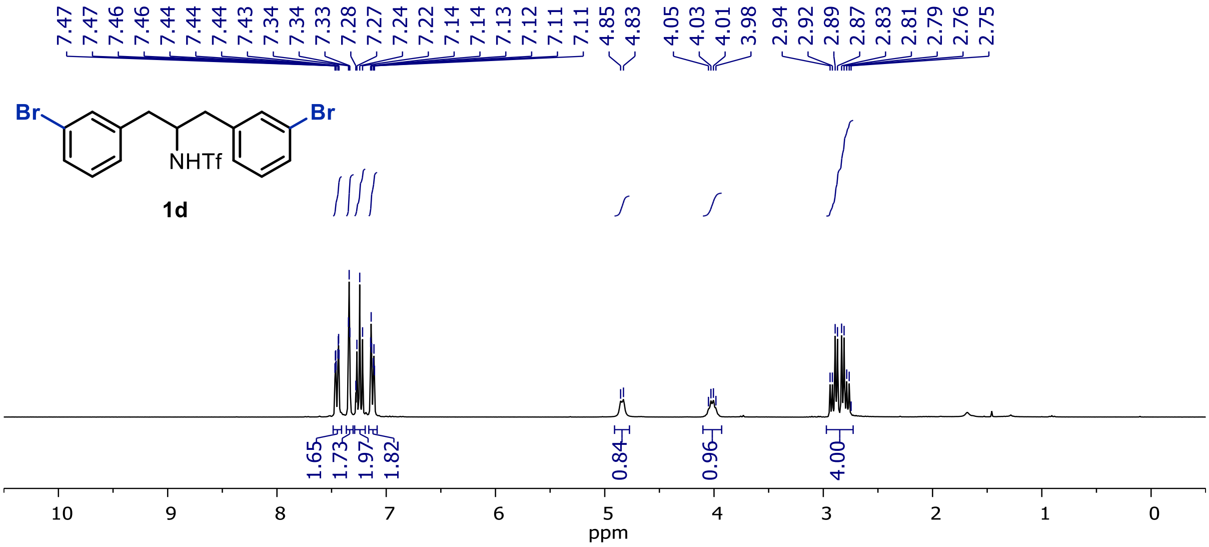

1d

DEPT-135

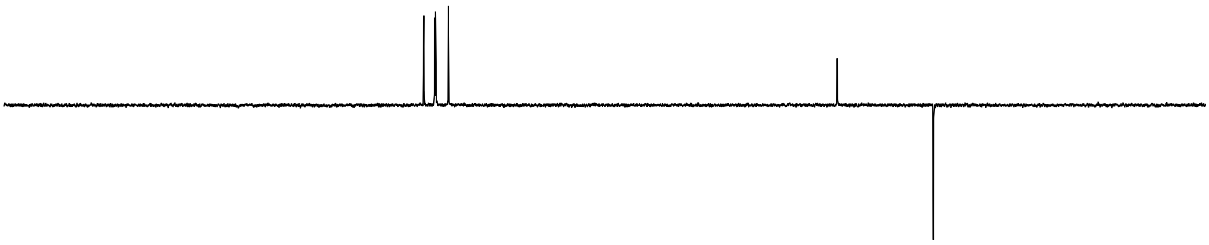

^{13}C NMR (75 MHz, $CDCl_3$)

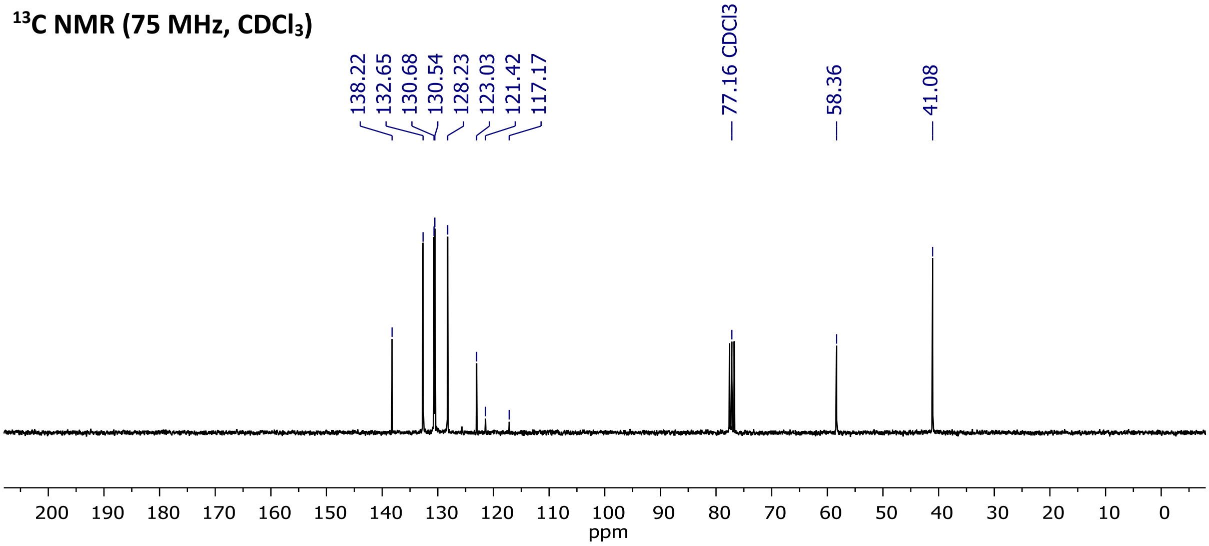

^{1}H NMR (300 MHz, $CDCl_3$)

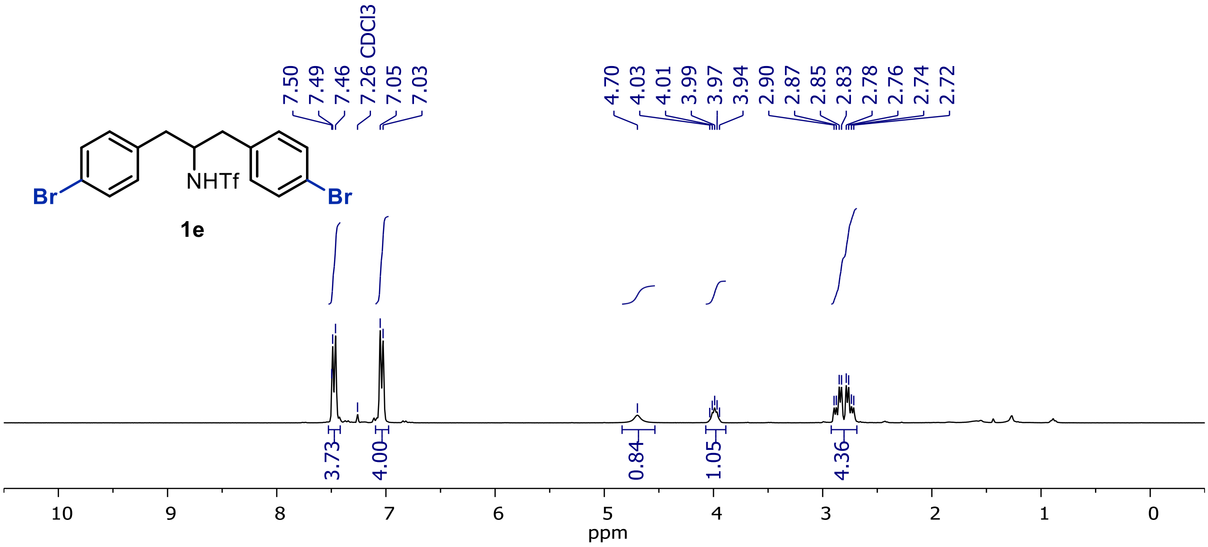

DEPT-135

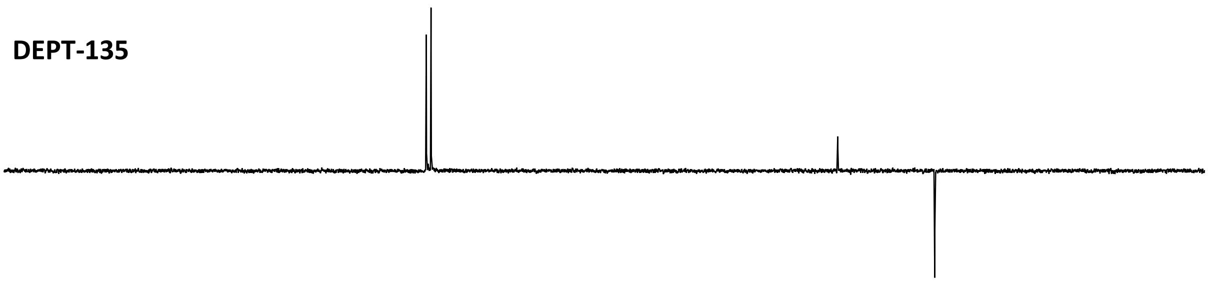

^{13}C NMR (75 MHz, $CDCl_3$)

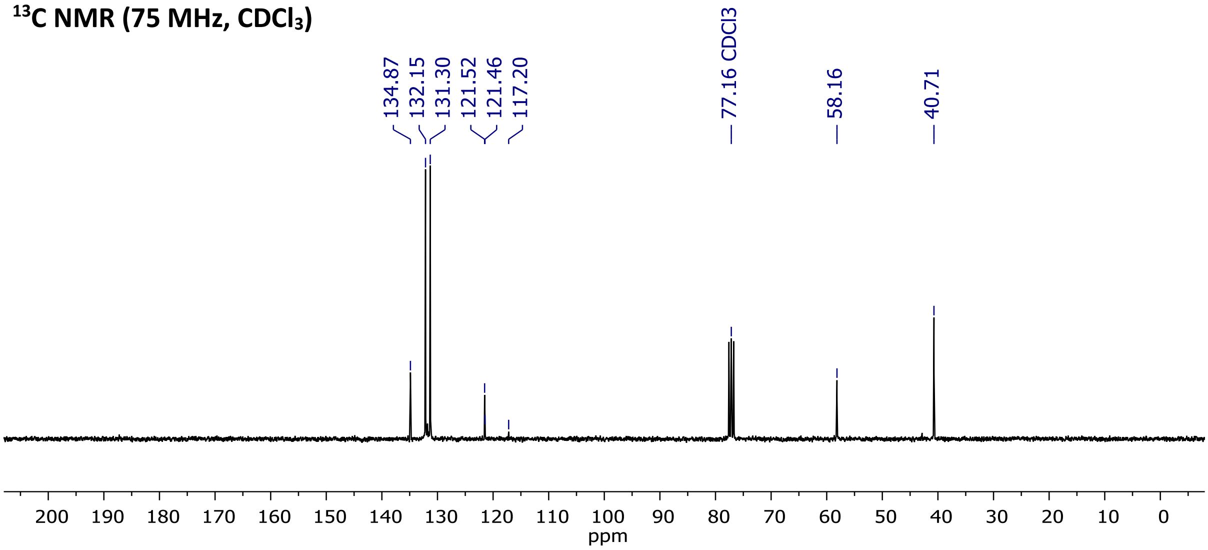

^{1}H NMR (300 MHz, CDCl$_3$)

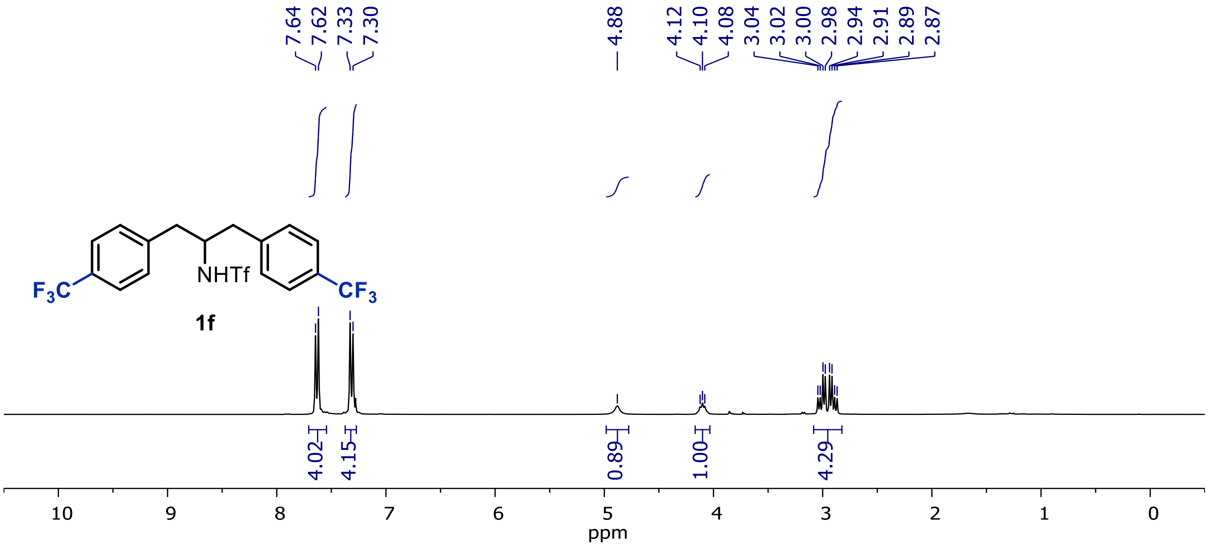

DEPT-135

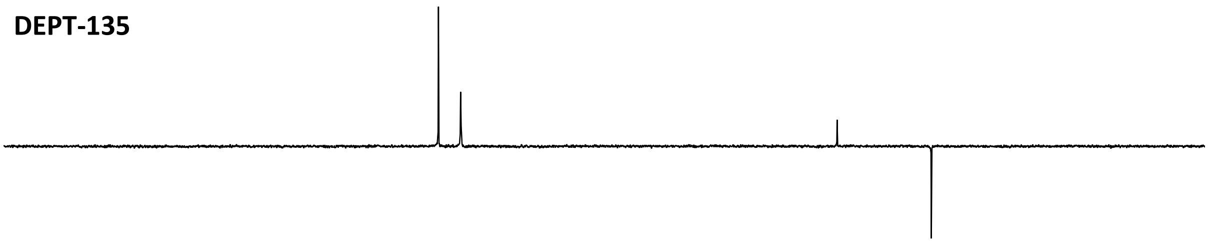

^{13}C NMR (75 MHz, CDCl$_3$)

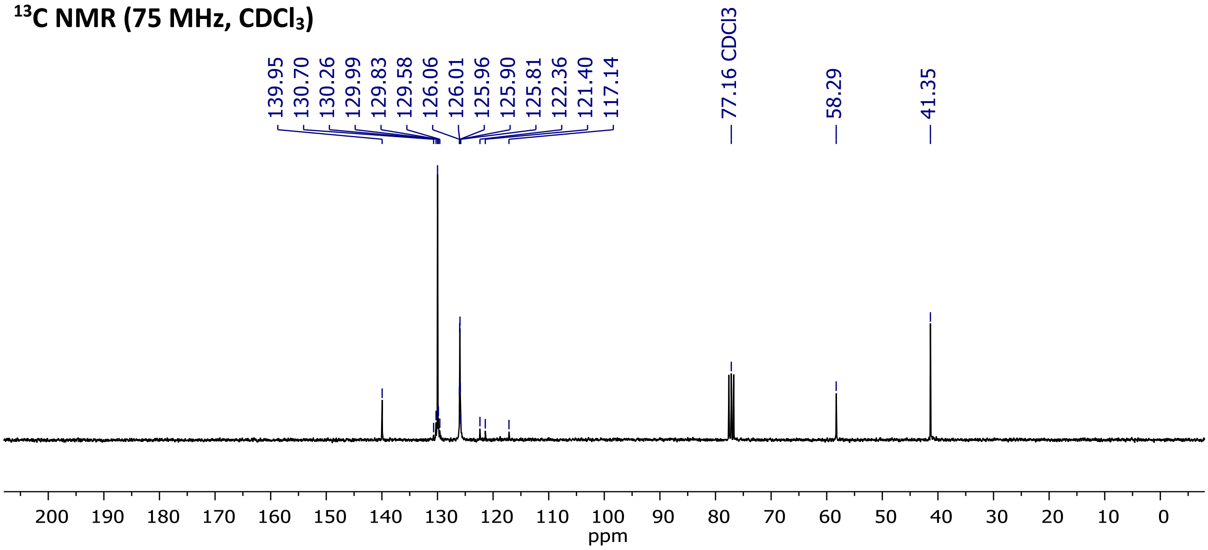

^{1}H NMR (300 MHz, $CDCl_3$)

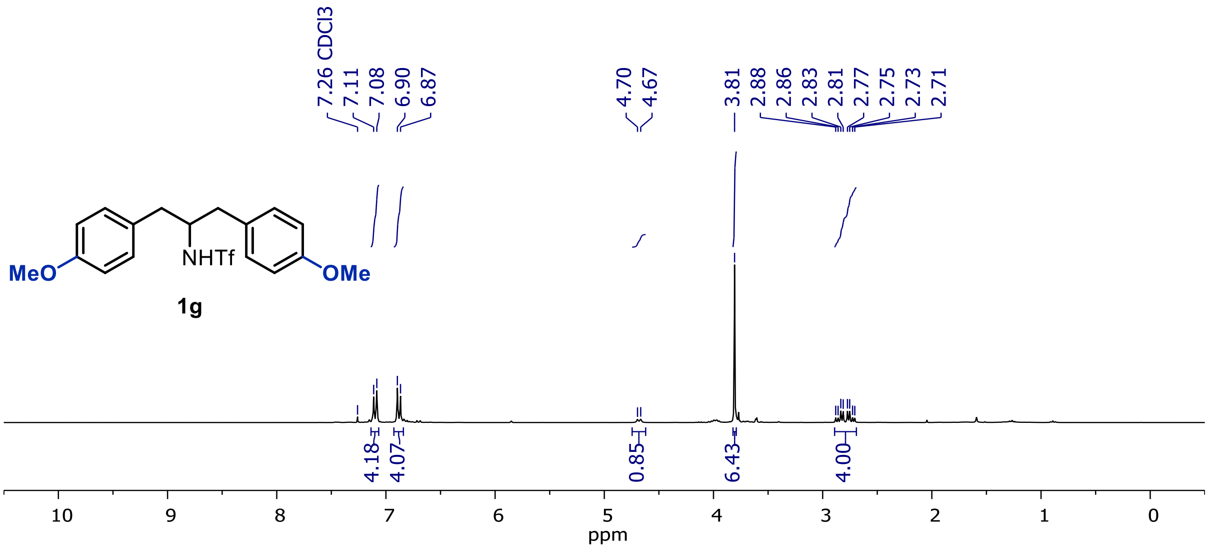

DEPT-135

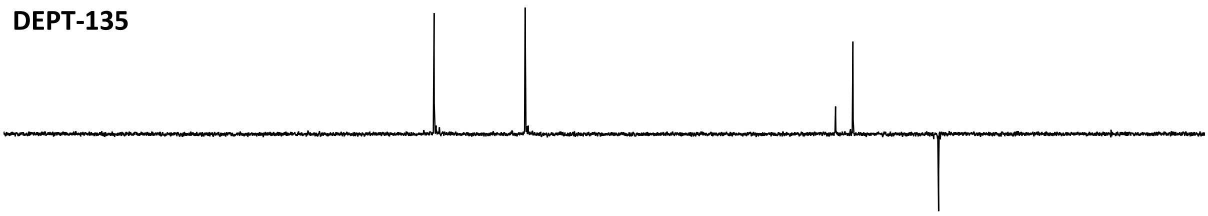

^{13}C NMR (75 MHz, $CDCl_3$)

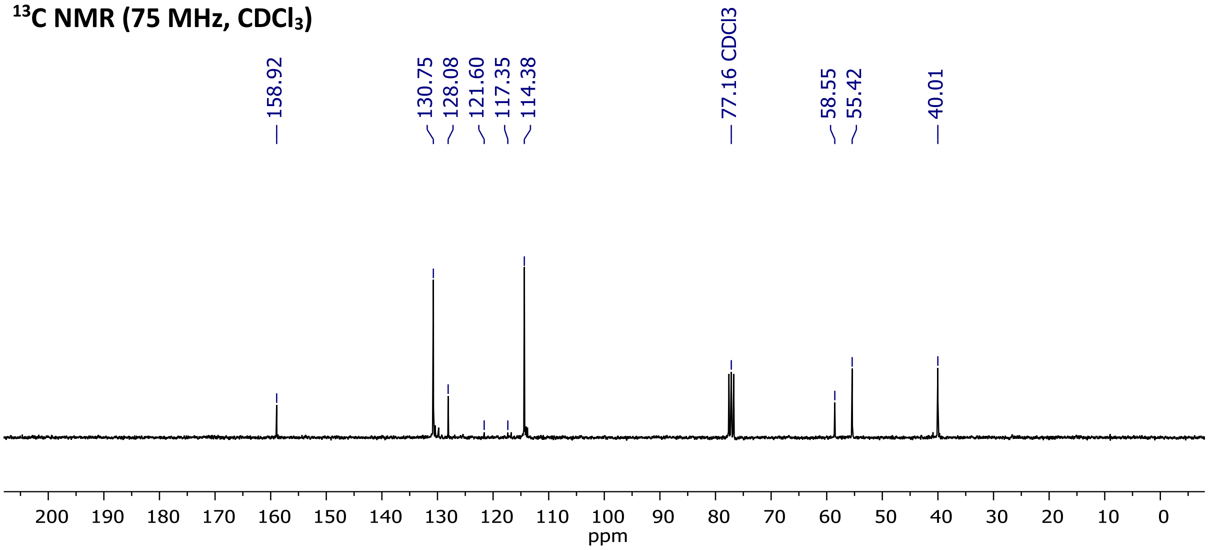

^{1}H NMR (300 MHz, $CDCl_3$)

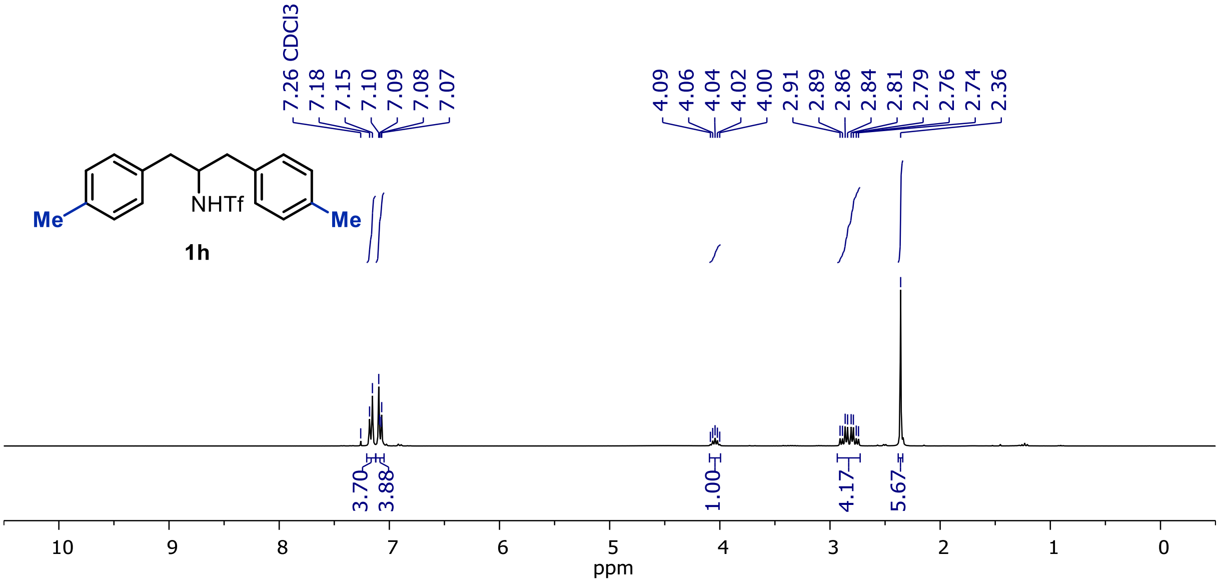

DEPT-135

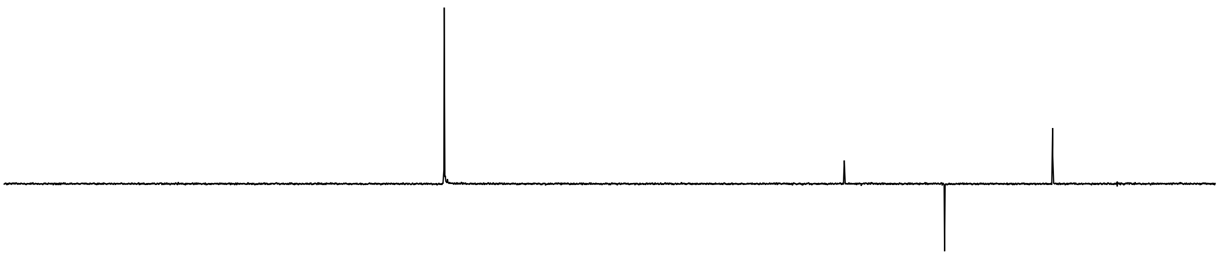

^{13}C NMR (75 MHz, $CDCl_3$)

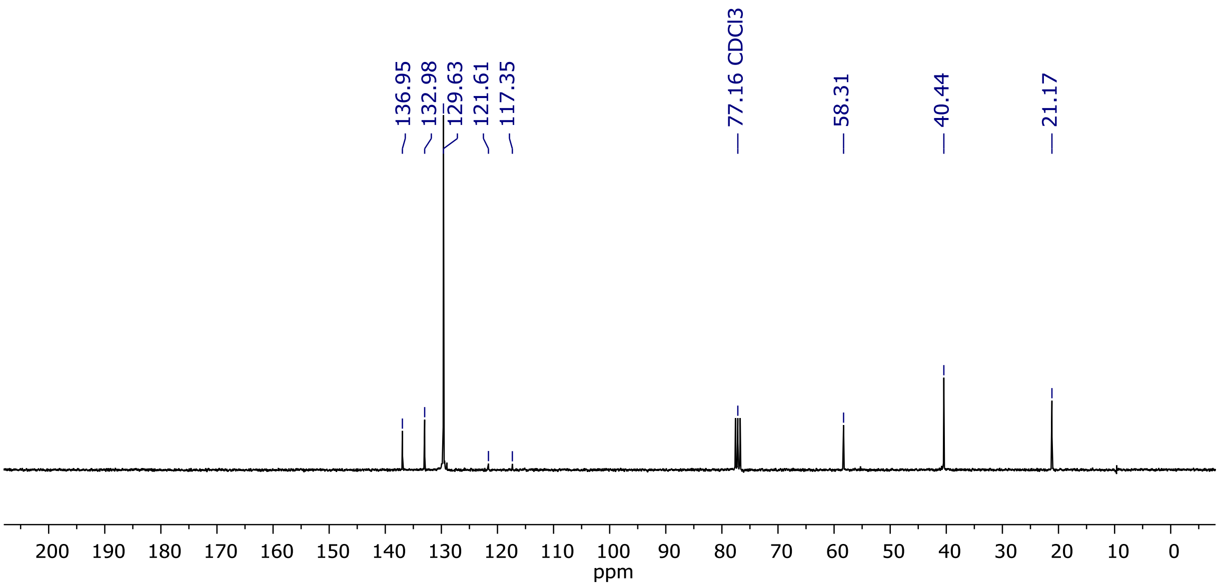

^{1}H NMR (300 MHz, $CDCl_3$)

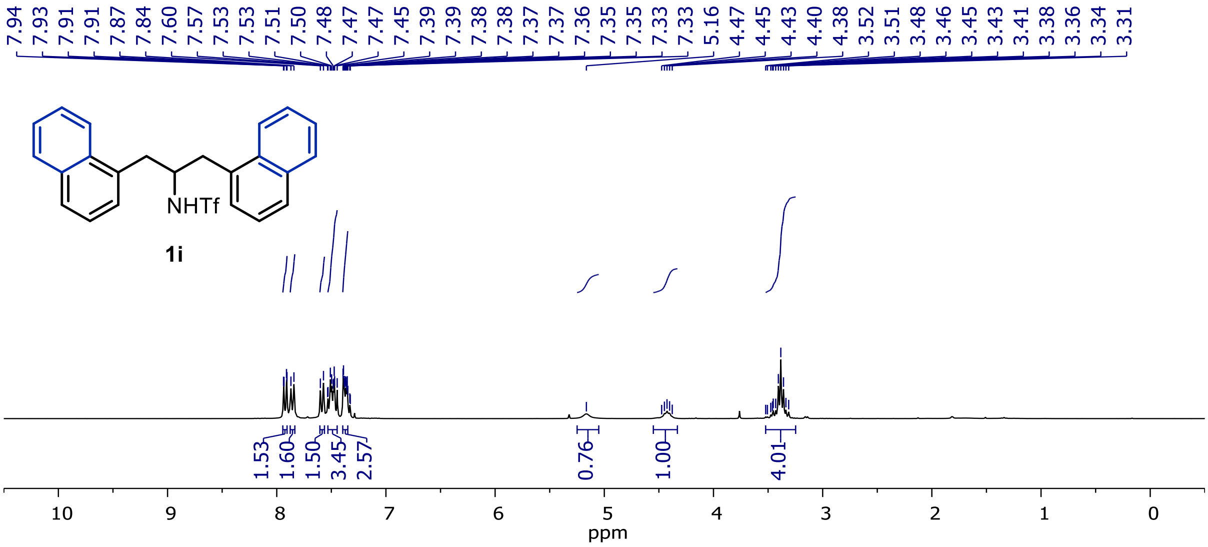

DEPT-135

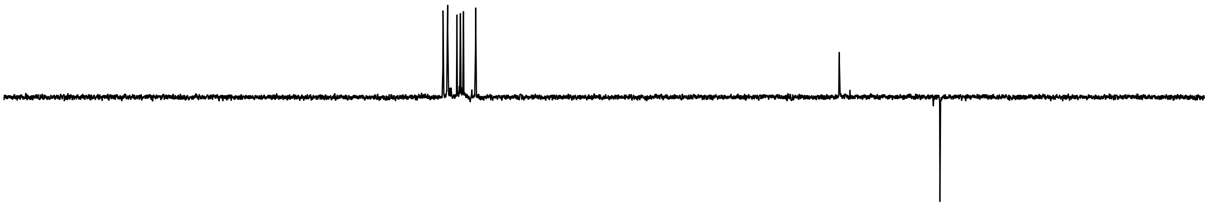

^{13}C NMR (75 MHz, $CDCl_3$)

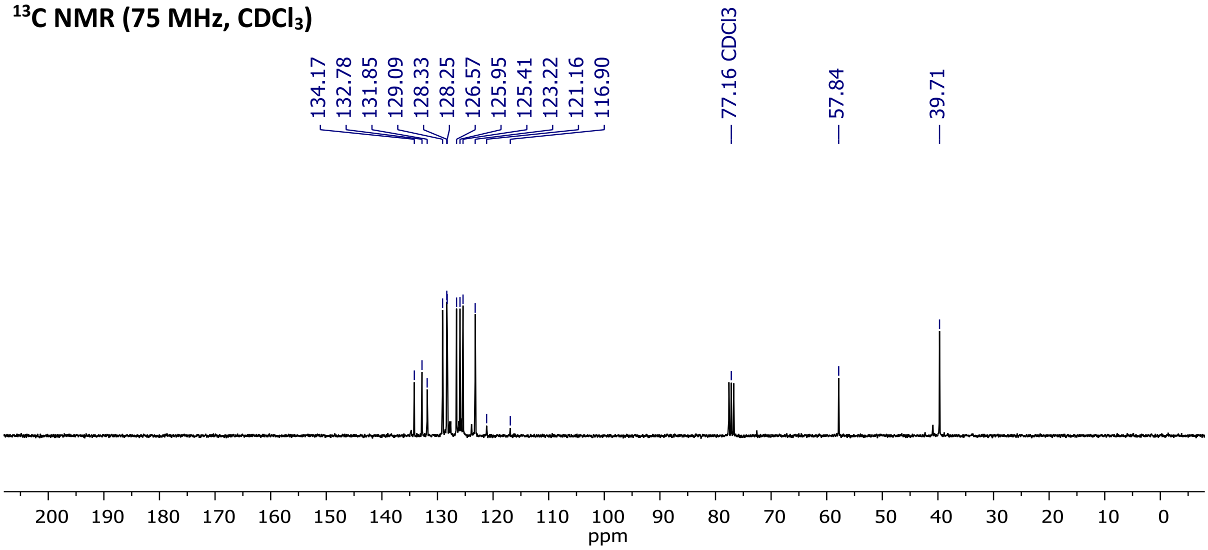

^{1}H NMR (300 MHz, $CDCl_3$)

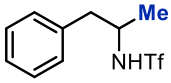

rac-**1j**

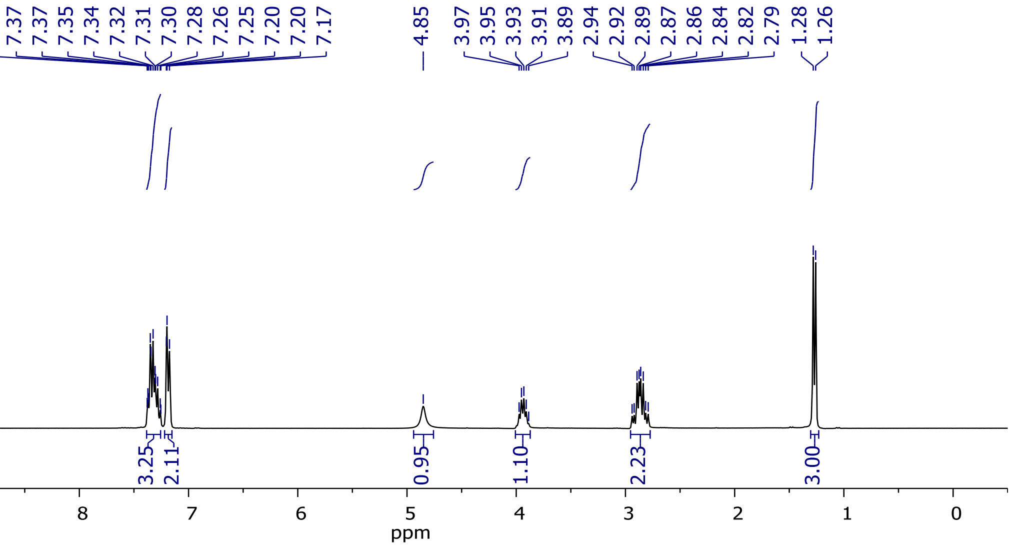

DEPT-135

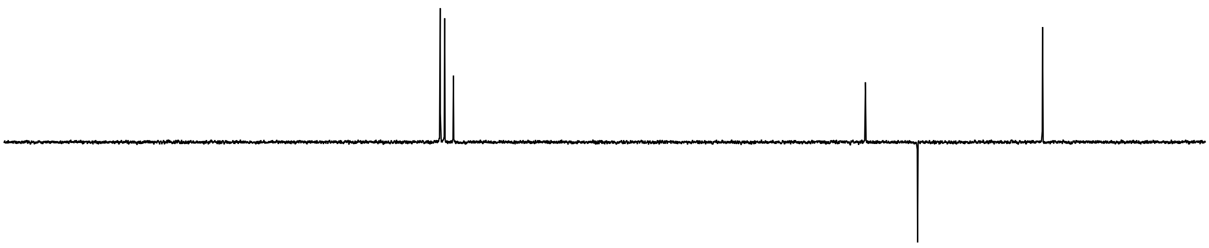

^{13}C NMR (75 MHz, $CDCl_3$)

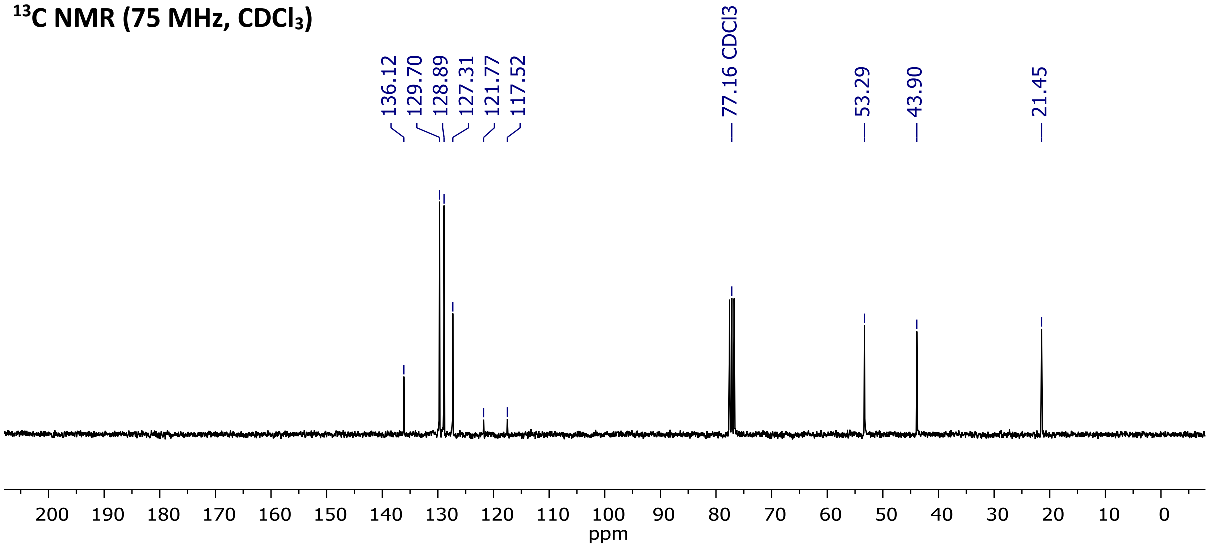

^{1}H NMR (300 MHz, $CDCl_3$)

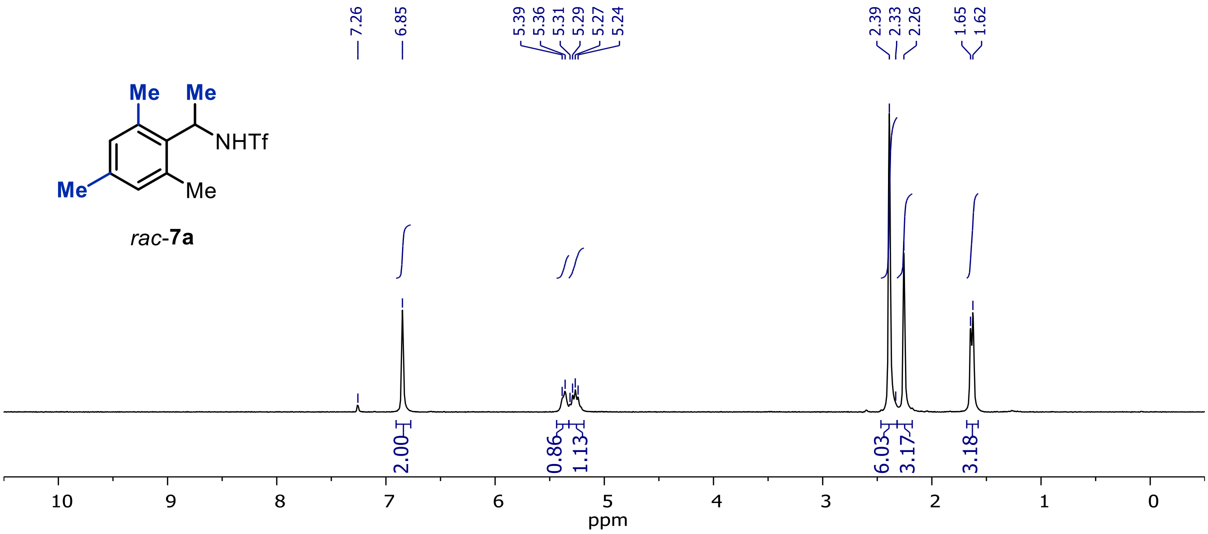

rac-**7a**

DEPT-135

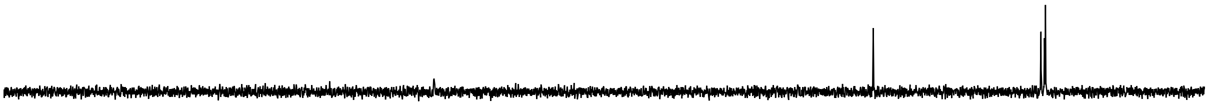

^{13}C NMR (75 MHz, $CDCl_3$)

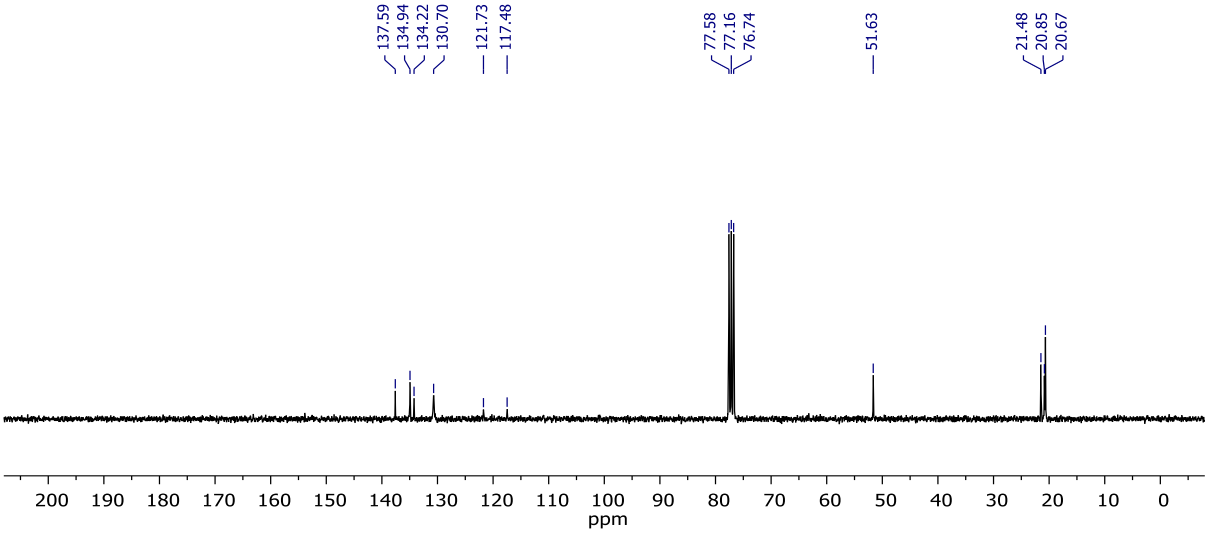

^{1}H NMR (500 MHz, CDCl$_3$)

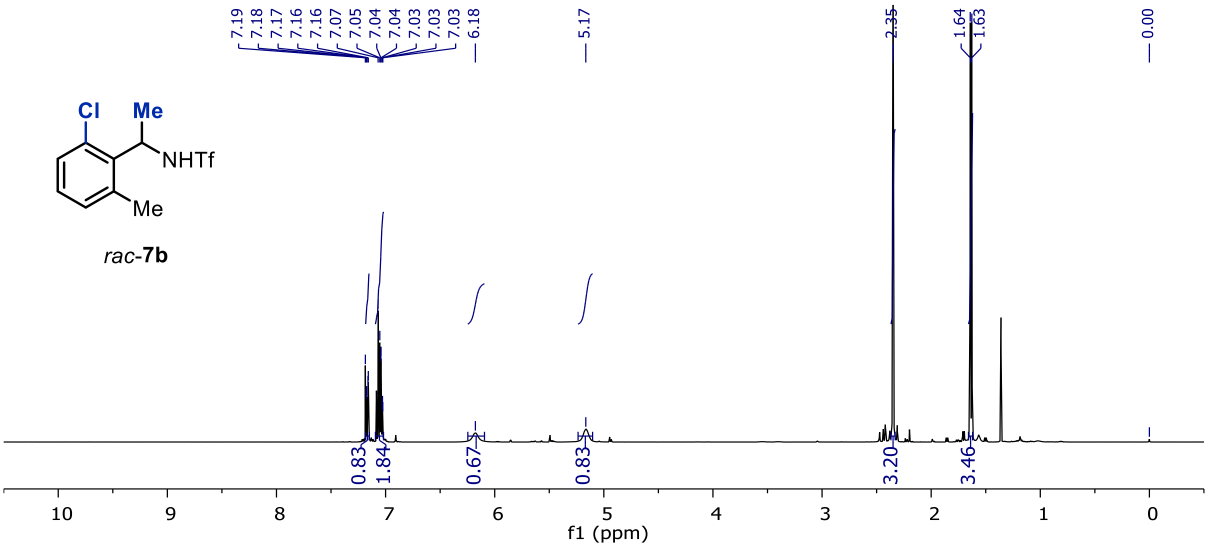

DEPT-135

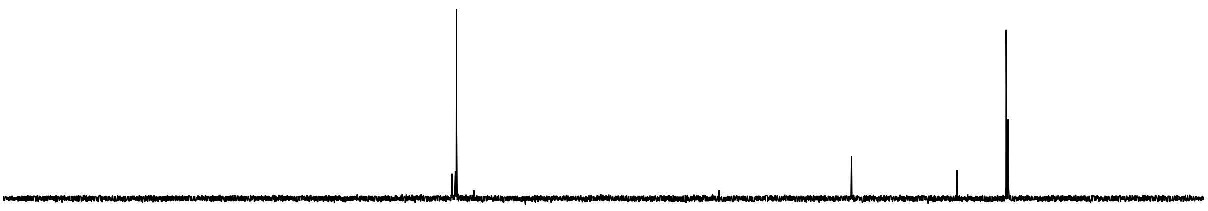

^{13}C NMR (126 MHz, CDCl$_3$)

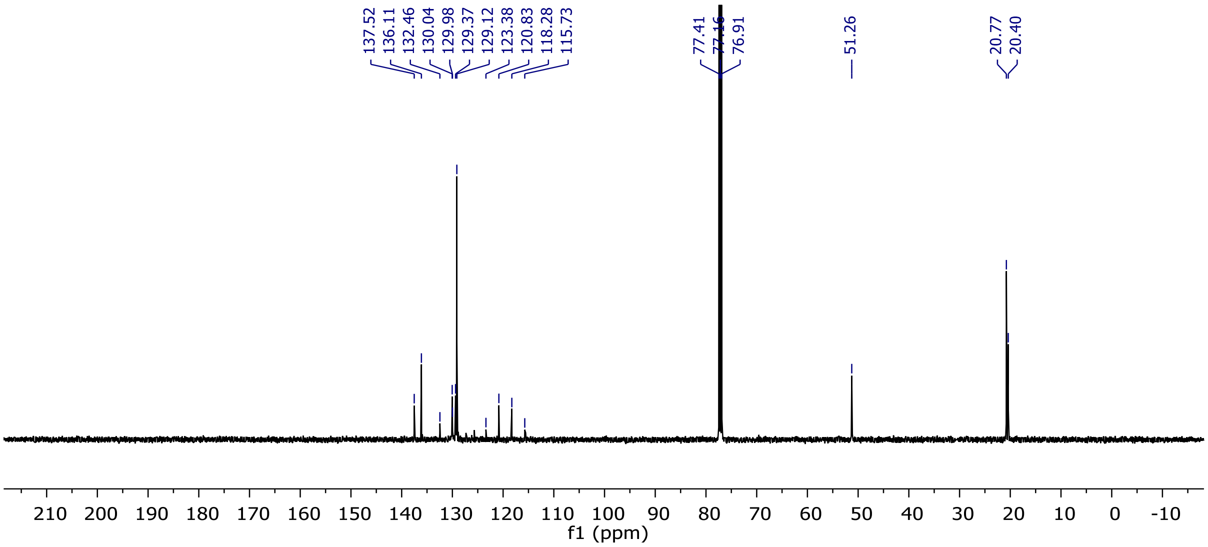

^{1}H NMR (500 MHz, $CDCl_3$)

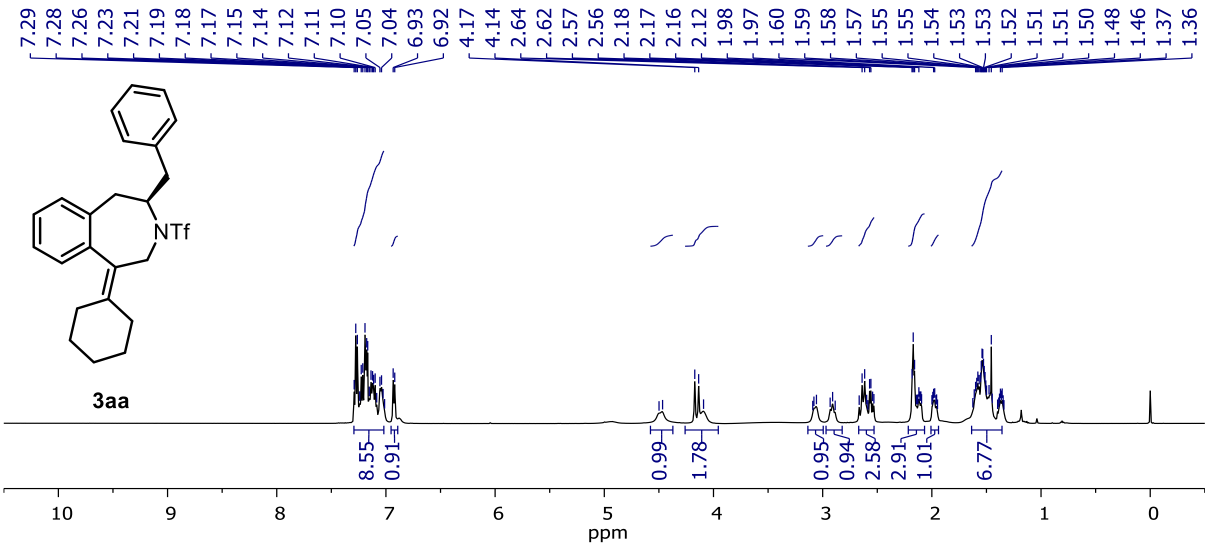

DEPT-135

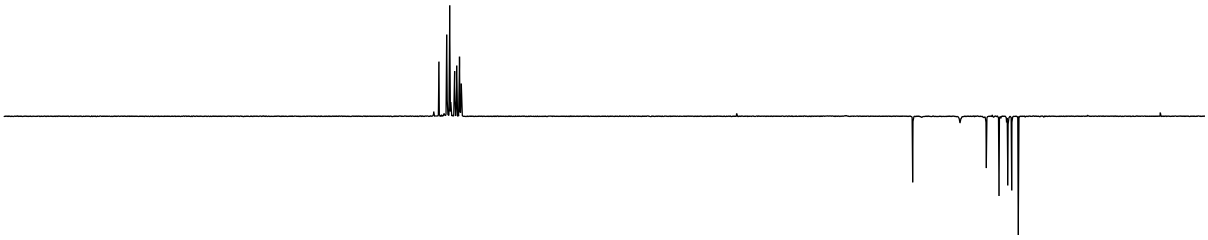

^{13}C NMR (126 MHz, $CDCl_3$)

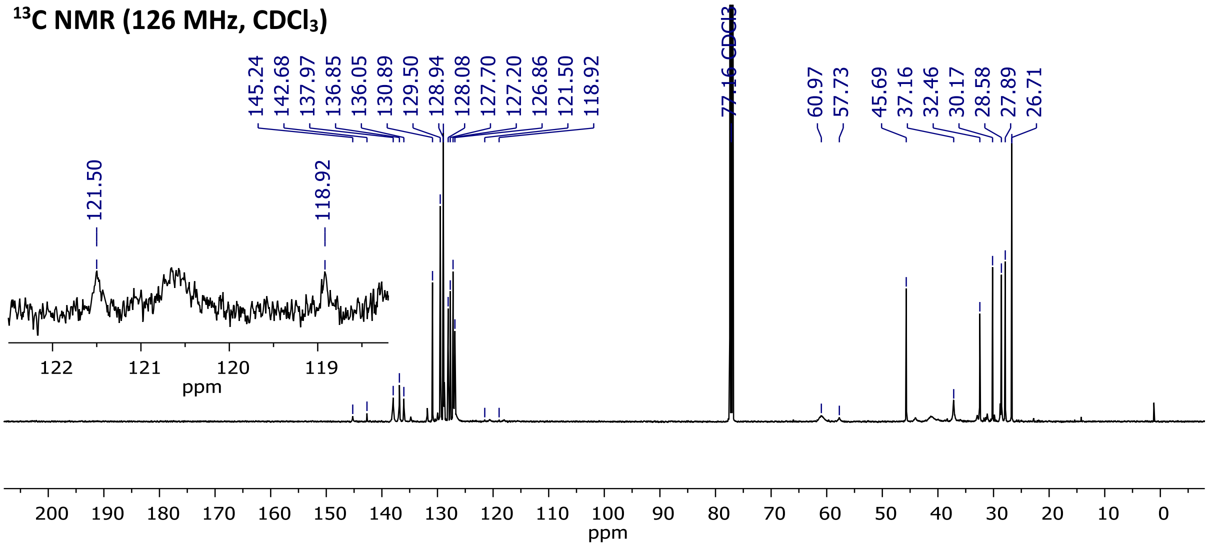

^{1}H NMR (500 MHz, $CDCl_3$)

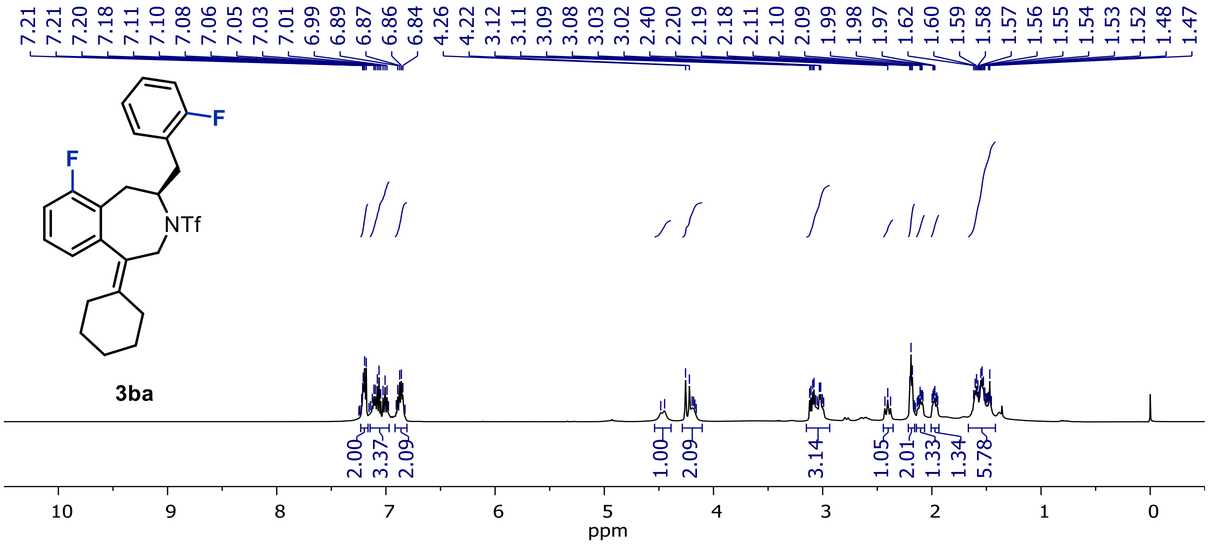

DEPT-135

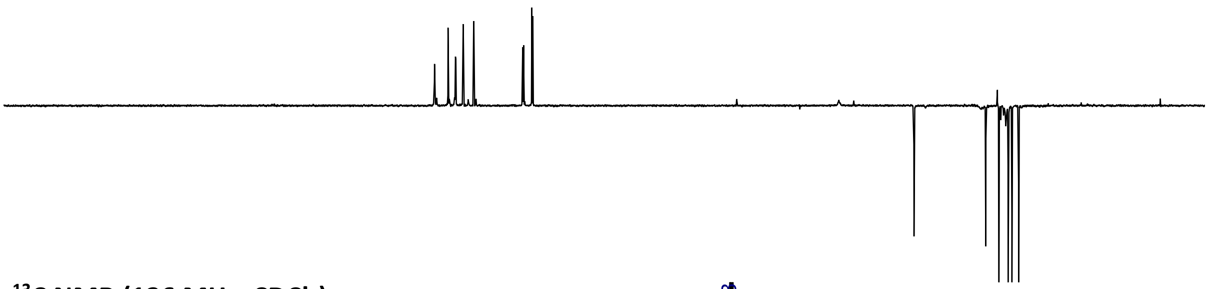

^{13}C NMR (126 MHz, $CDCl_3$)

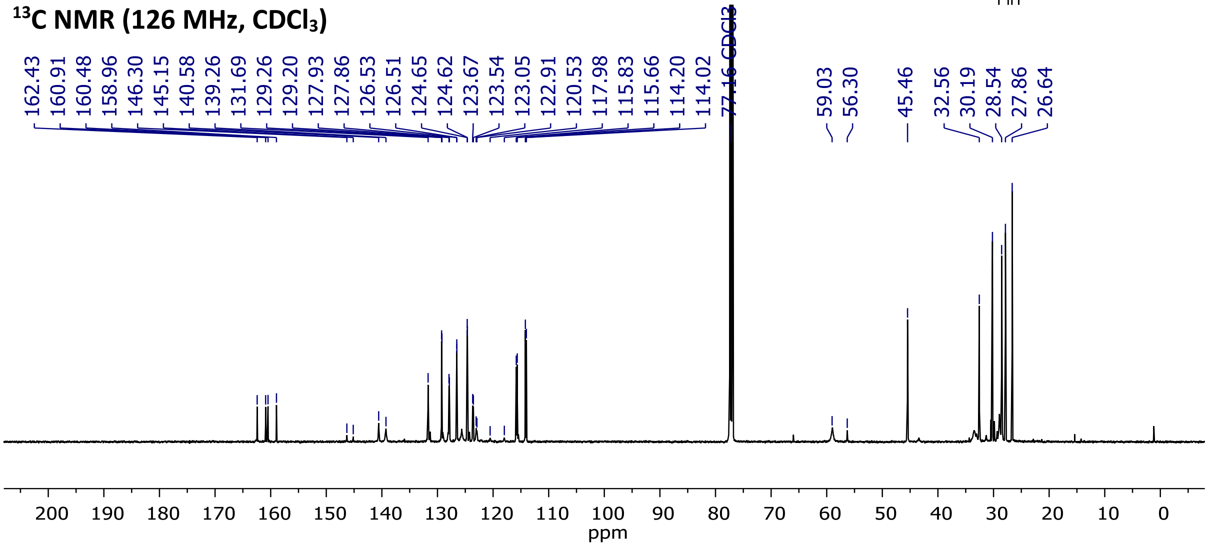

^{1}H NMR (500 MHz, CDCl$_3$)

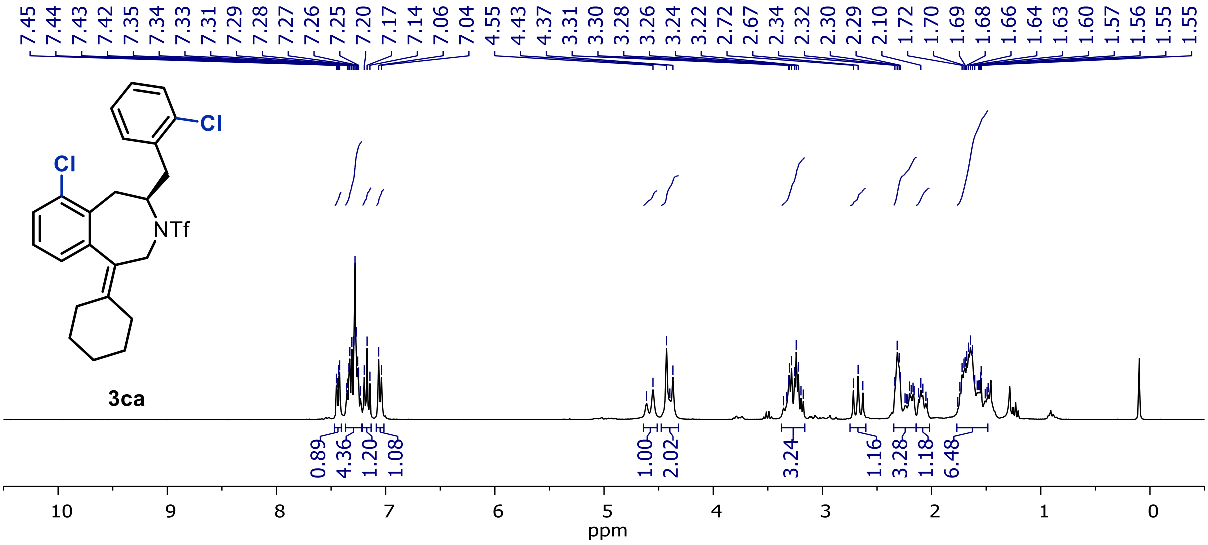

DEPT-135

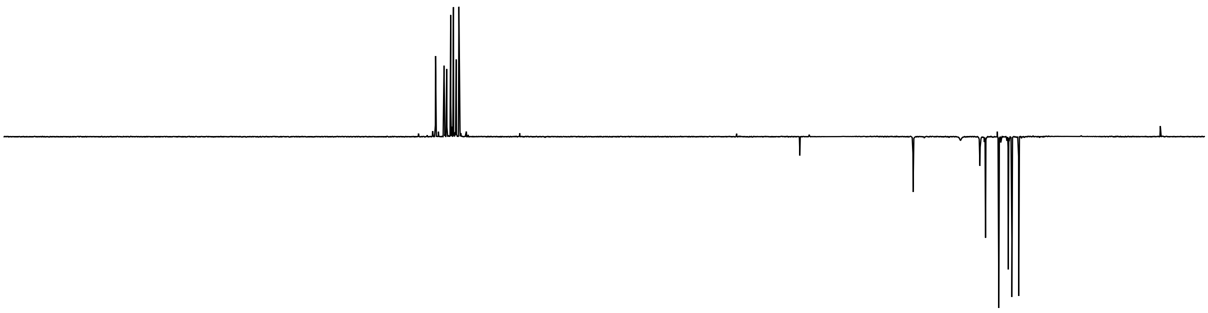

^{13}C NMR (126 MHz, CDCl$_3$)

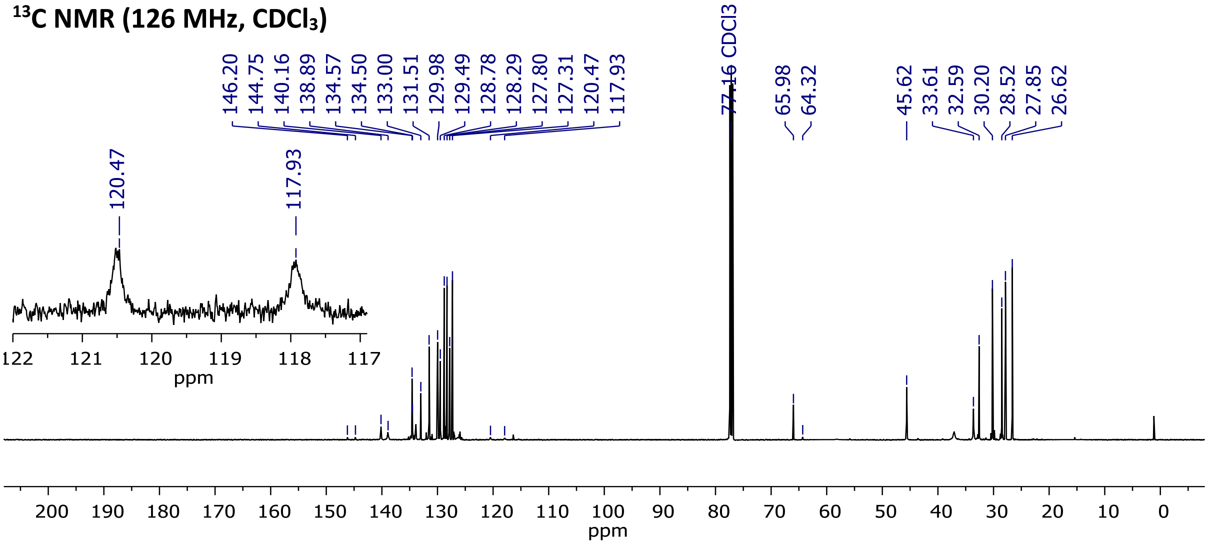

^{1}H NMR (500 MHz, CDCl$_3$)

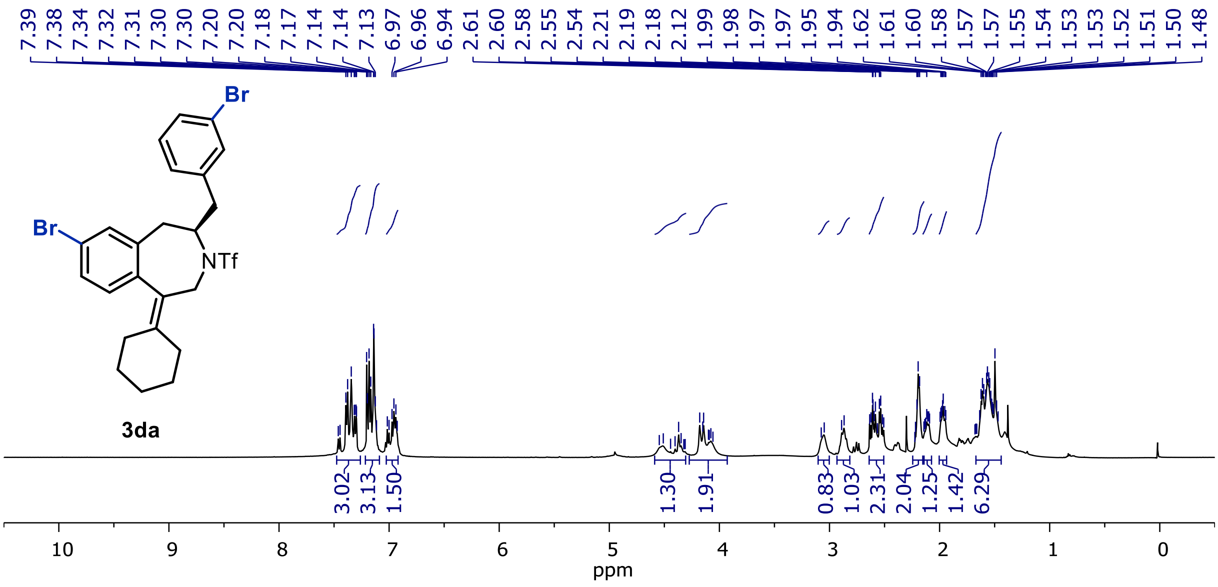

DEPT-135

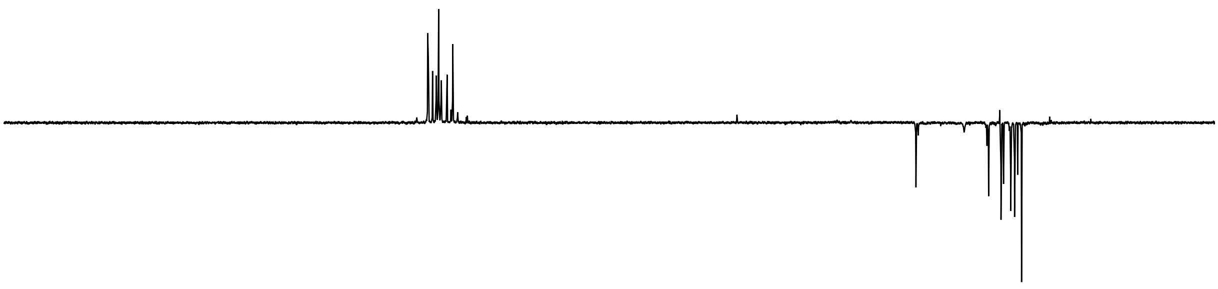

^{13}C NMR (126 MHz, CDCl$_3$)

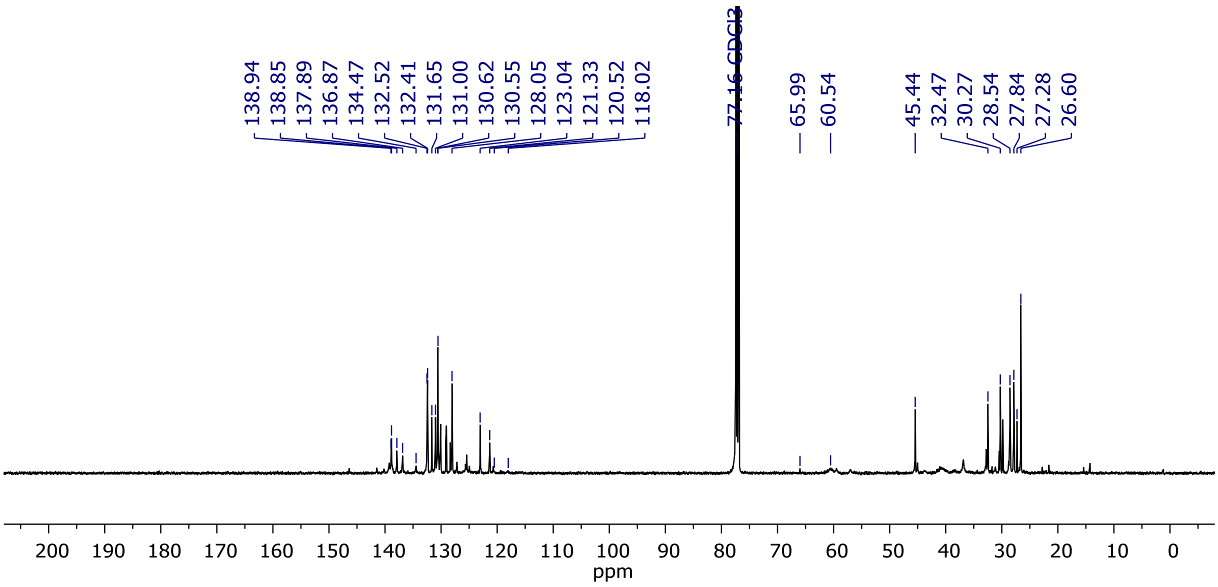

^{1}H NMR (500 MHz, $CDCl_3$)

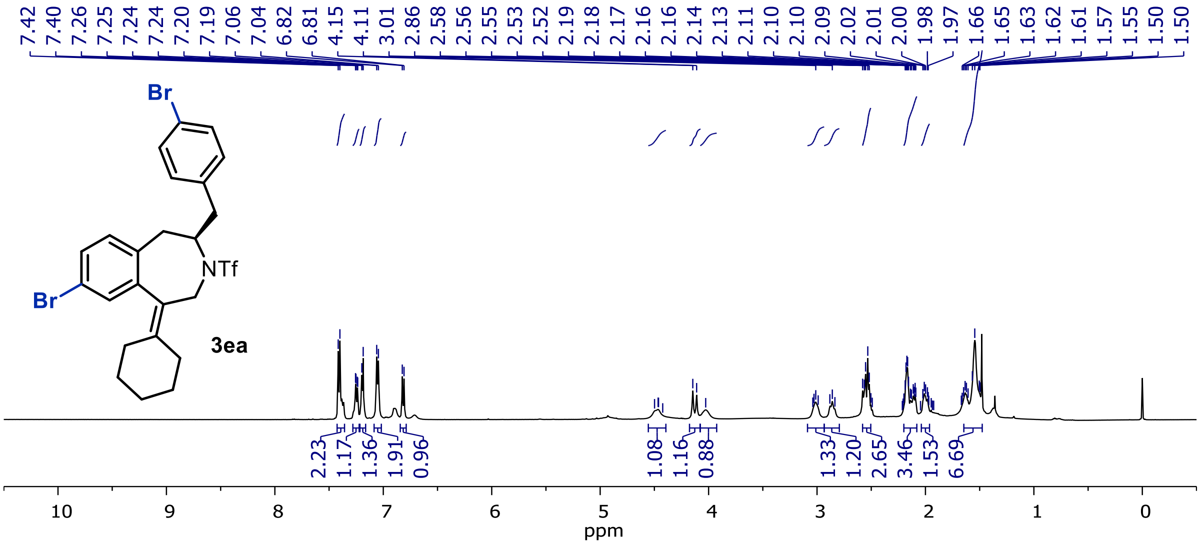

DEPT-135

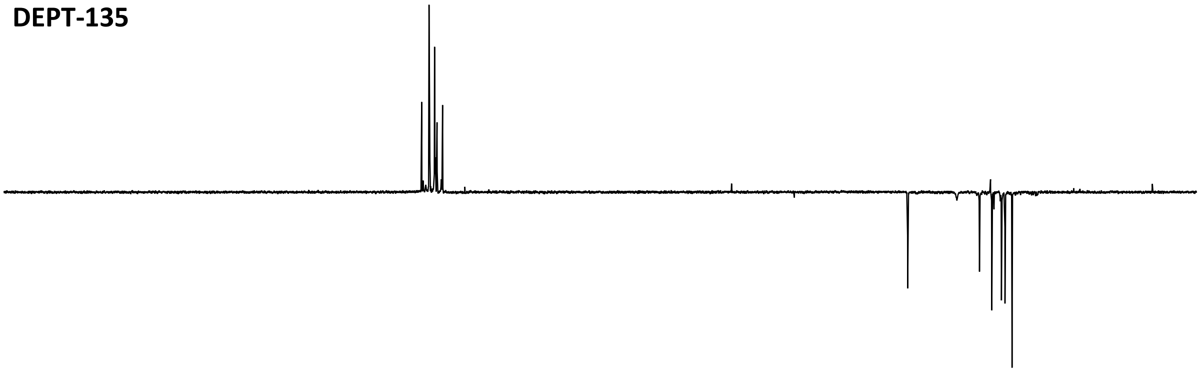

^{13}C NMR (126 MHz, $CDCl_3$)

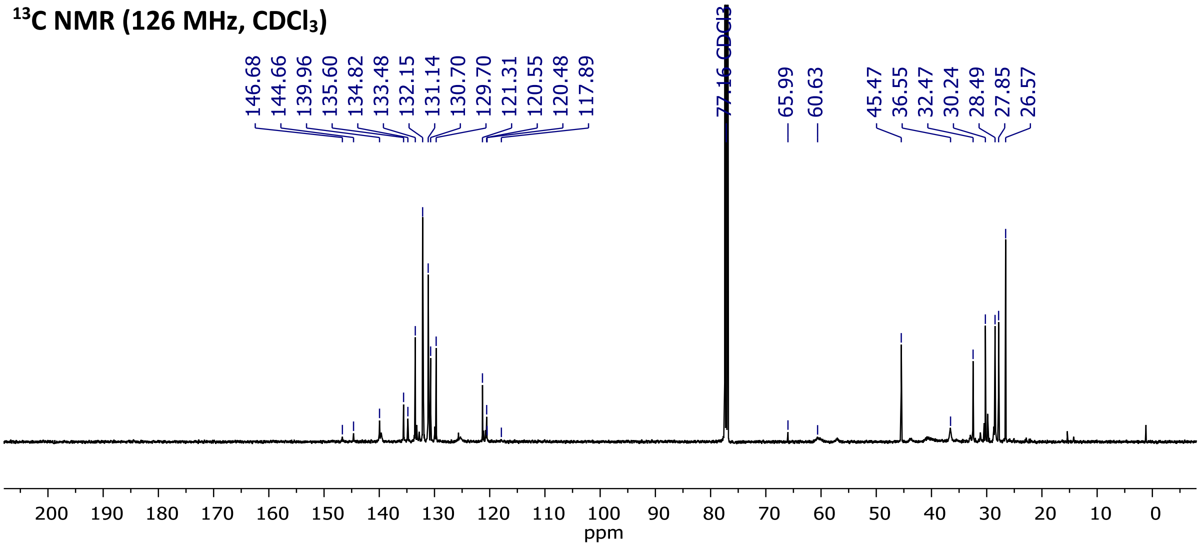

^{1}H NMR (500 MHz, CDCl$_3$)

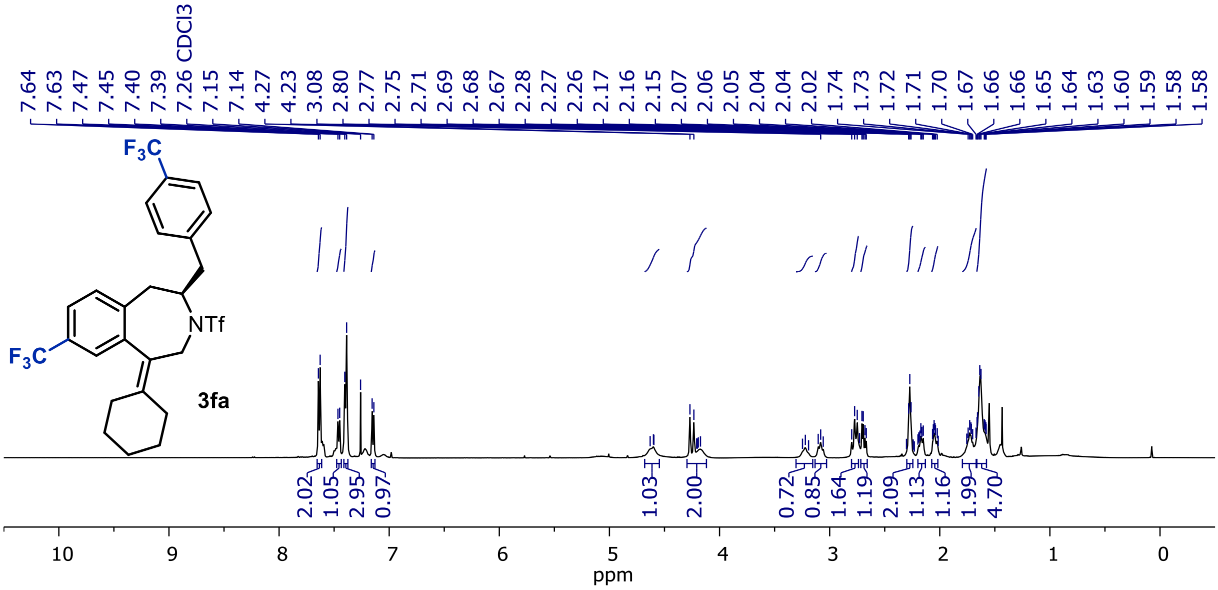

DEPT-135

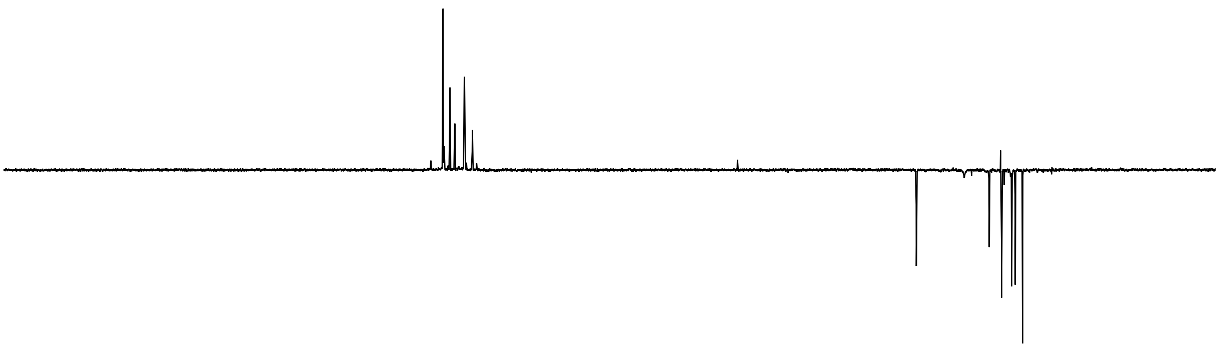

^{13}C NMR (126 MHz, CDCl$_3$)

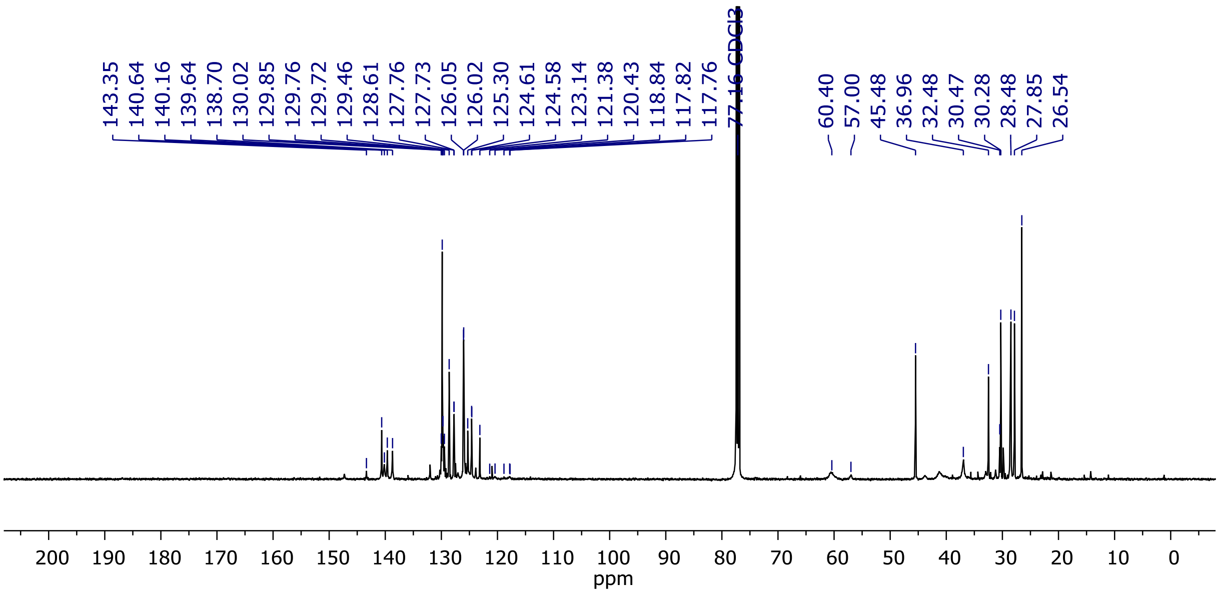

^{1}H NMR (500 MHz, $CDCl_3$)

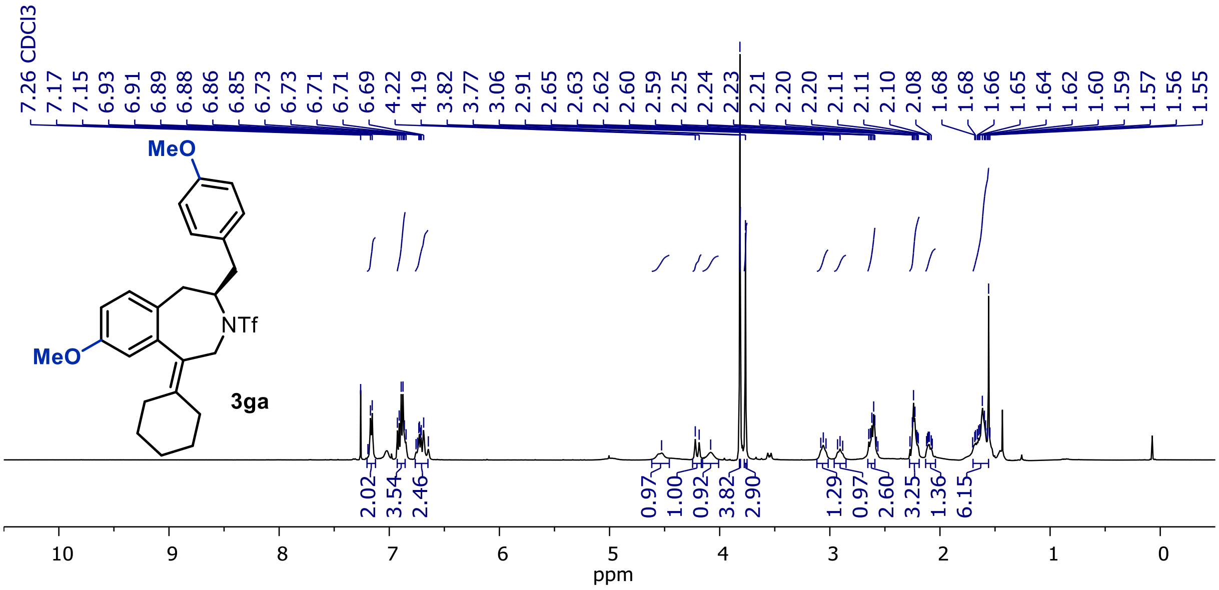

DEPT-135

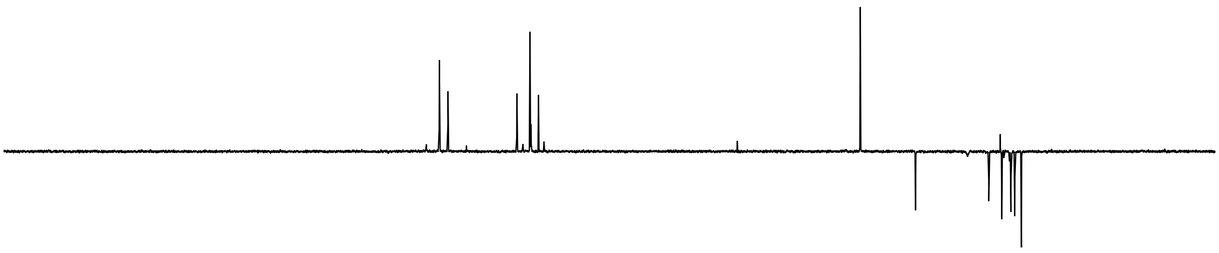

^{13}C NMR (126 MHz, $CDCl_3$)

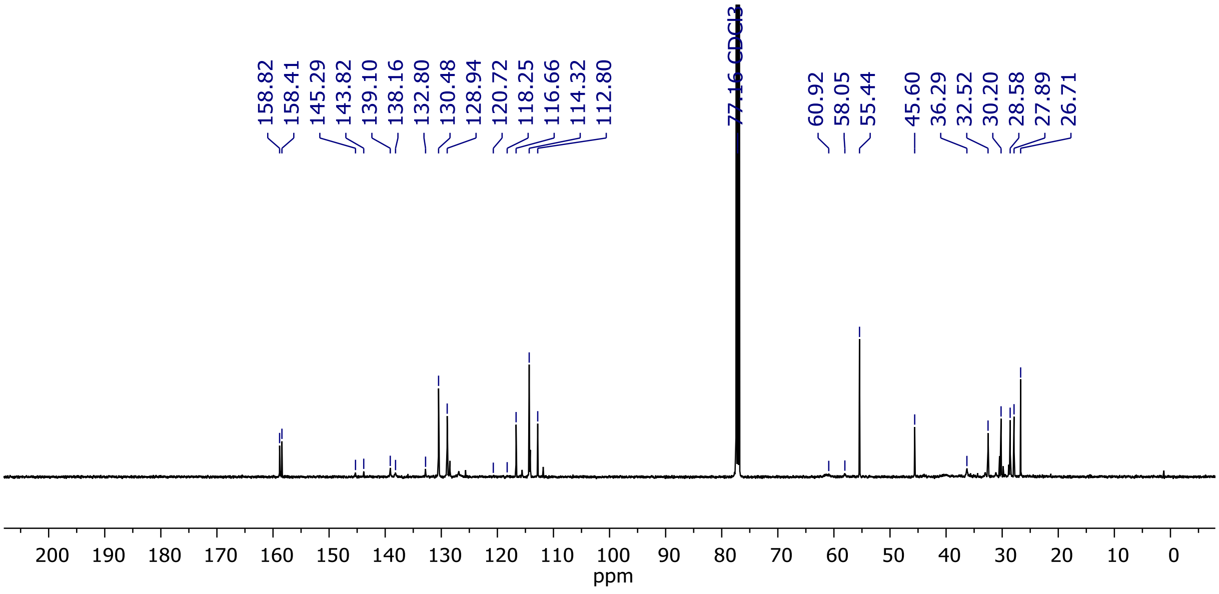

^{1}H NMR (500 MHz, $CDCl_3$)

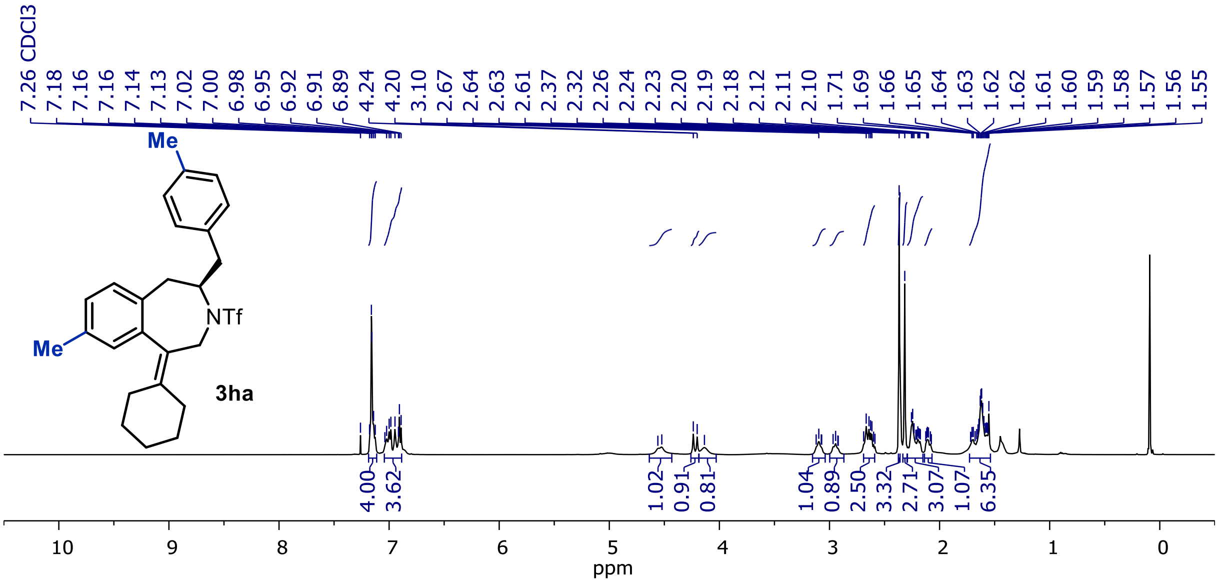

DEPT-135

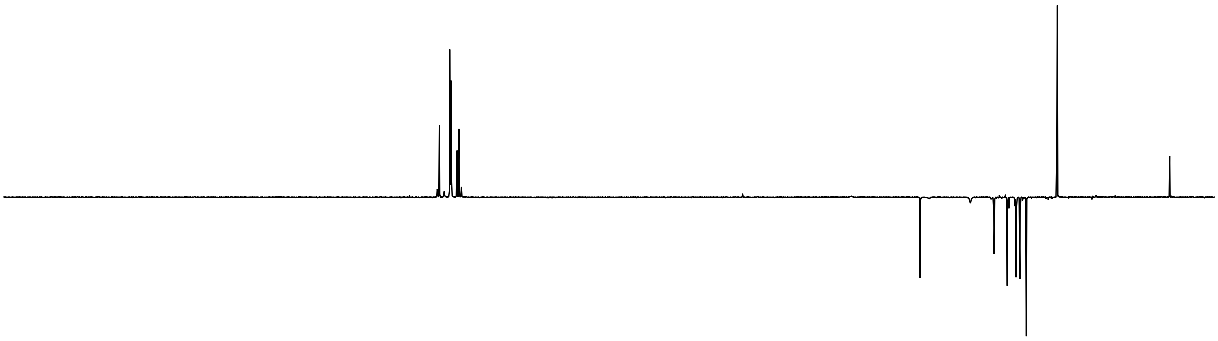

^{13}C NMR (126 MHz, $CDCl_3$)

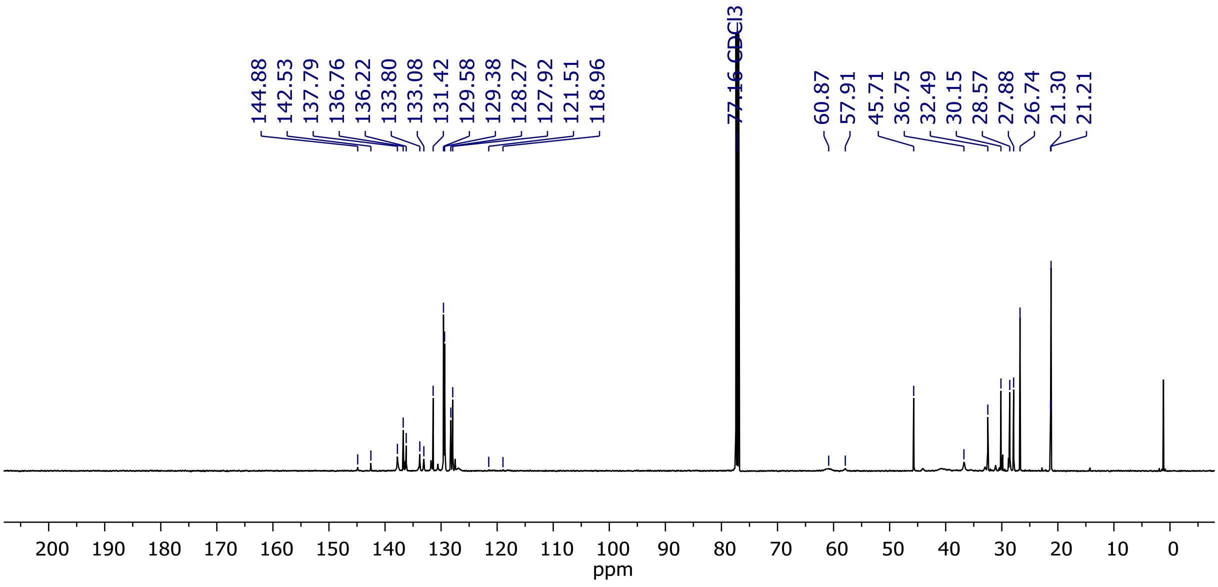

^{1}H NMR (500 MHz, $CDCl_3$)

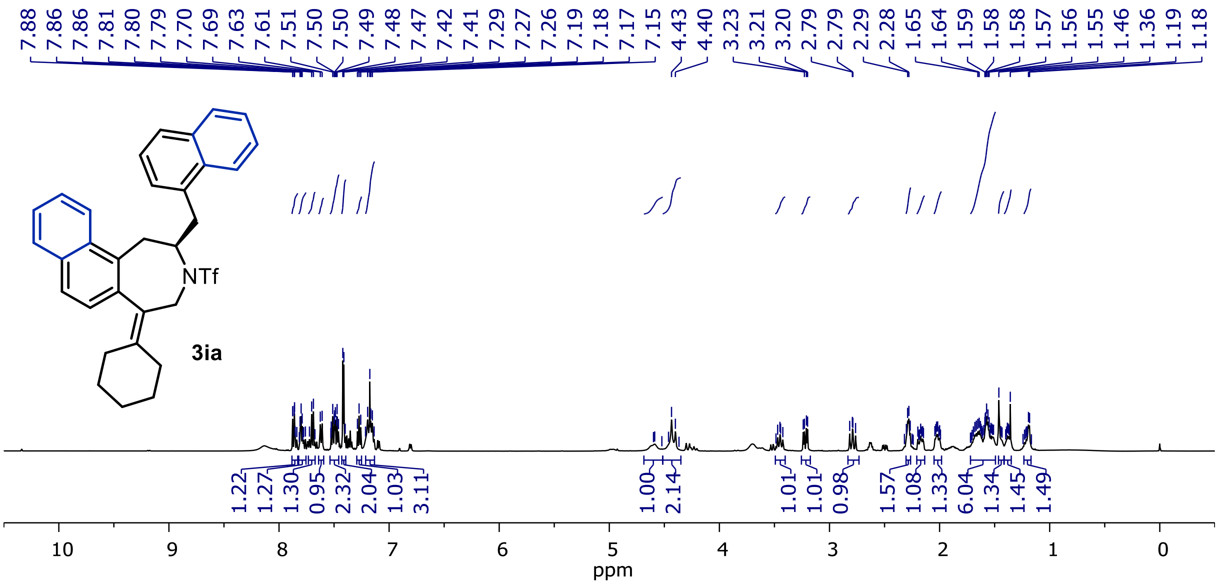

DEPT-135

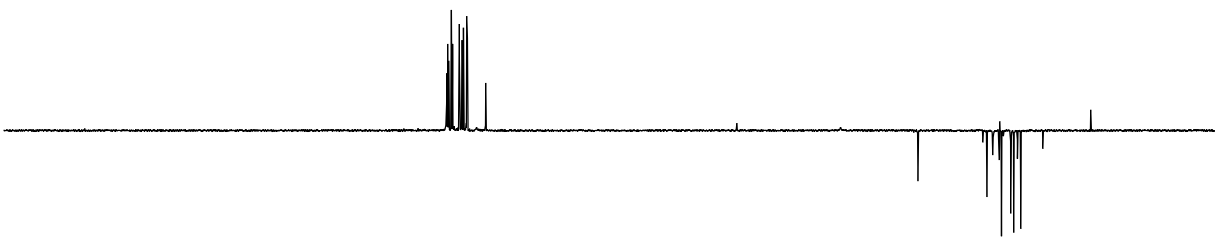

^{13}C NMR (126 MHz, $CDCl_3$)

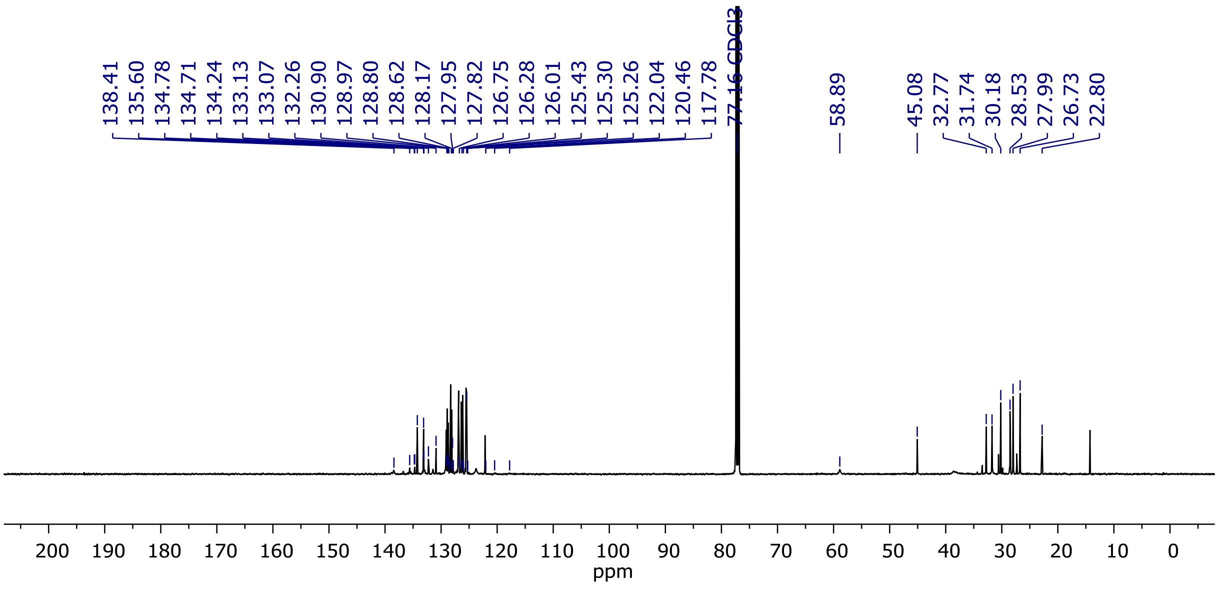

^{1}H NMR (500 MHz, $CDCl_3$)

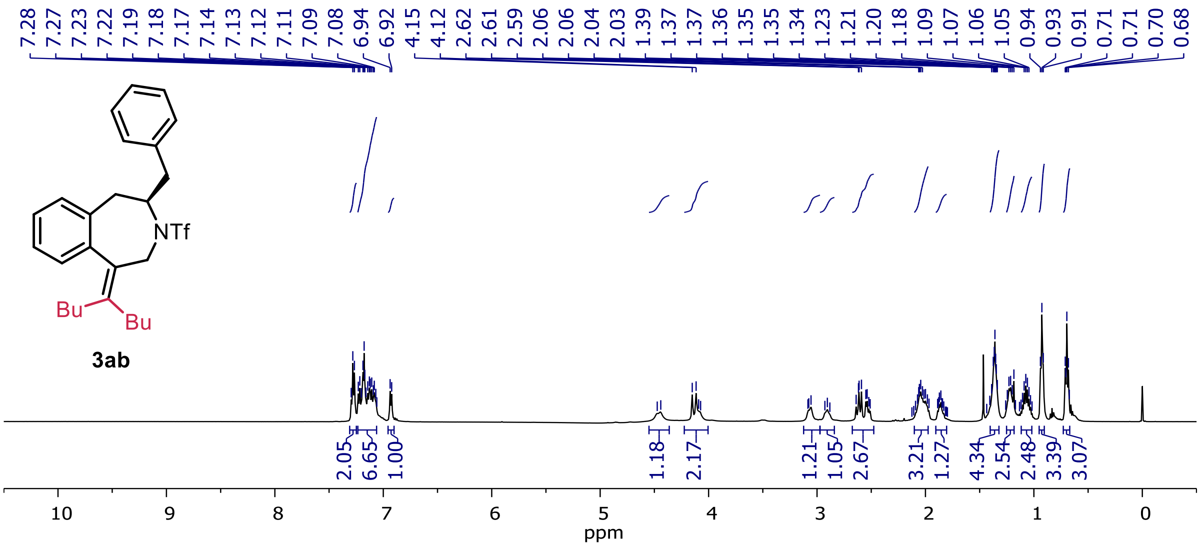

DEPT-135

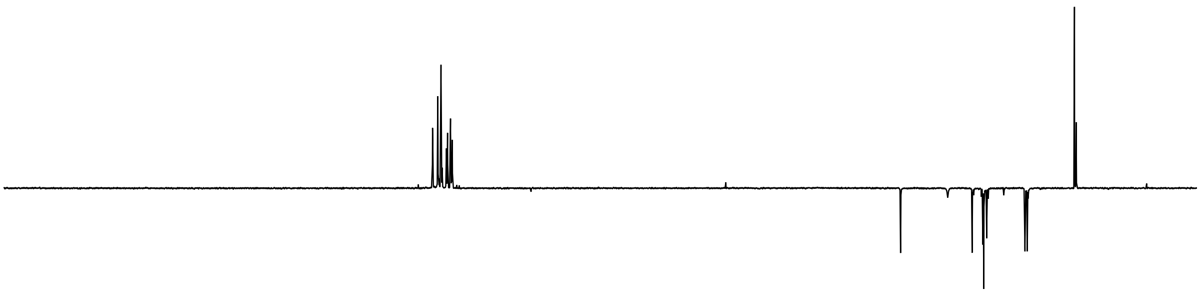

^{13}C NMR (126 MHz, $CDCl_3$)

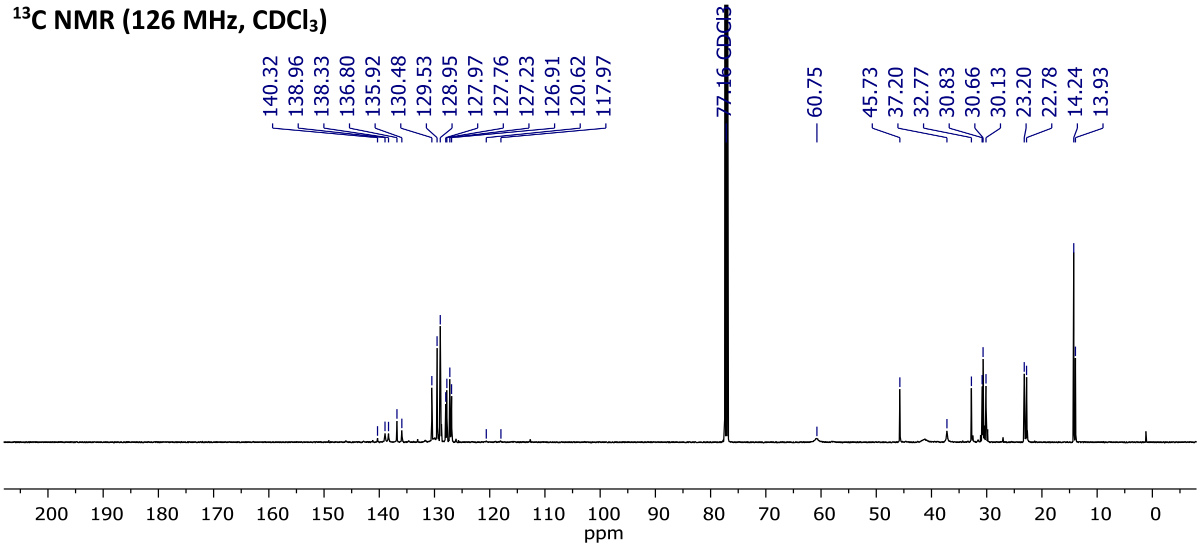

^{1}H NMR (500 MHz, $CDCl_3$)

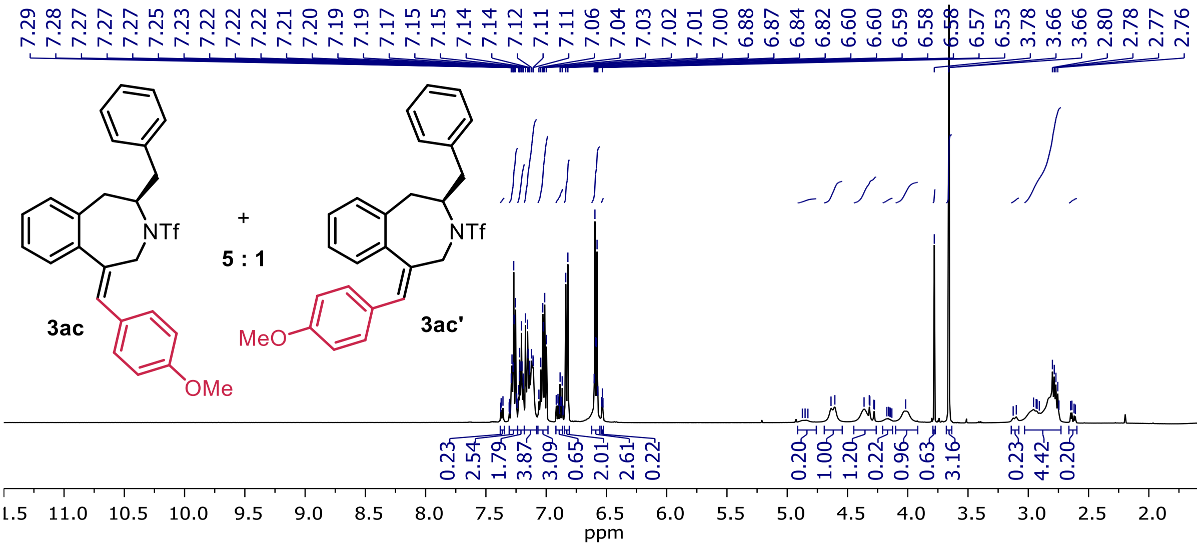

DEPT-135

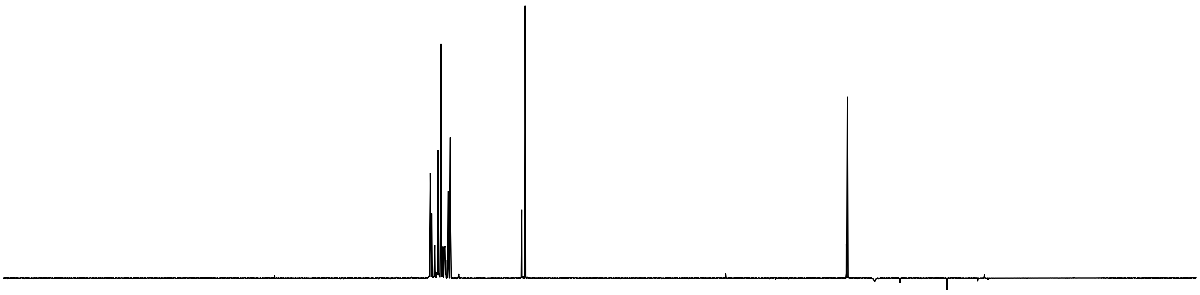

^{13}C NMR (126 MHz, $CDCl_3$)

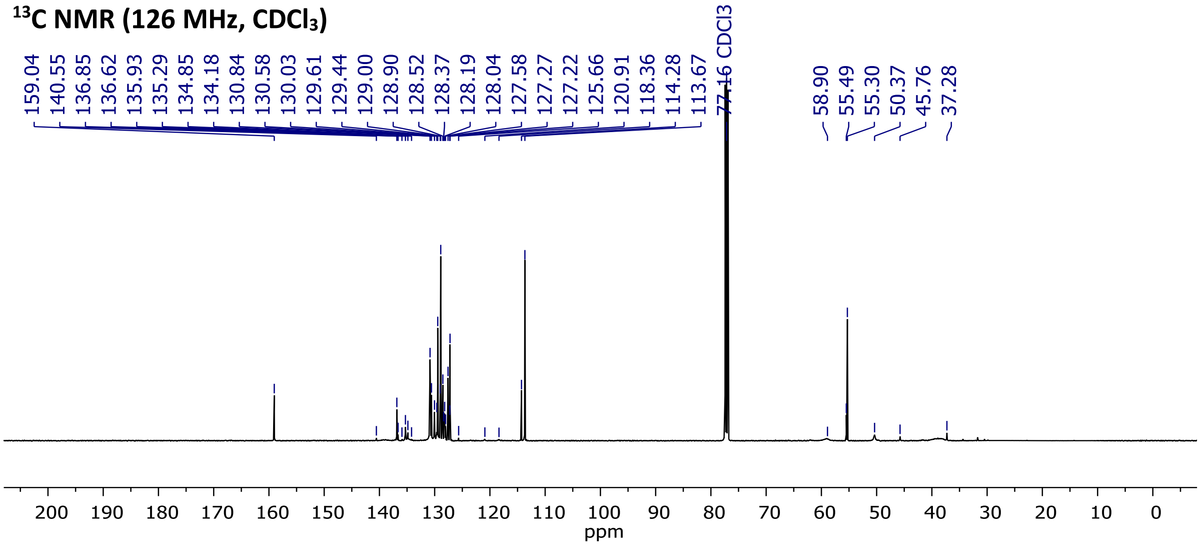

^{1}H NMR (500 MHz, CDCl$_3$)

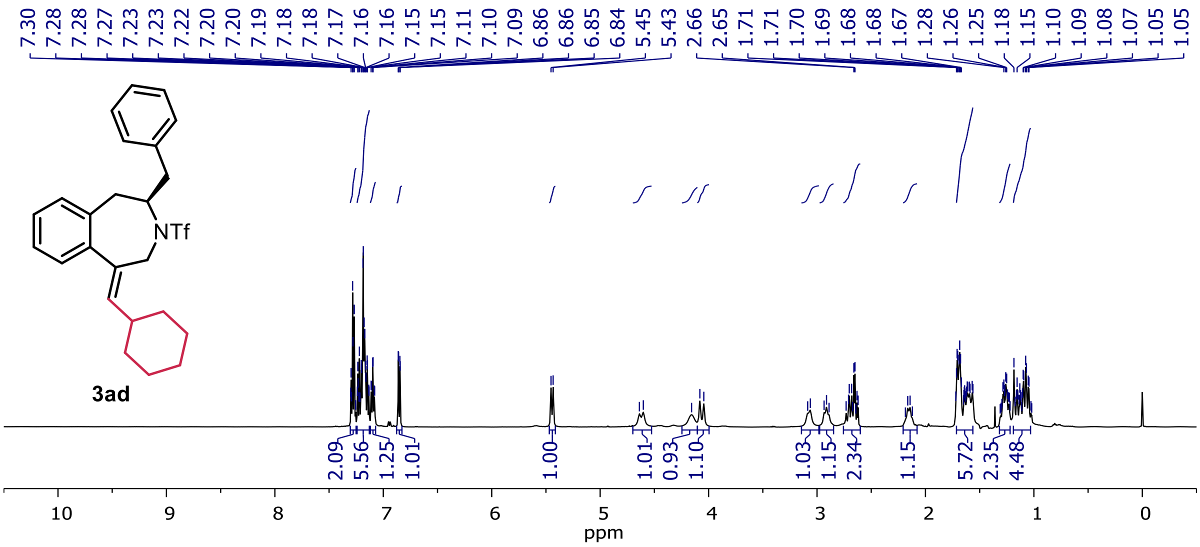

DEPT-135

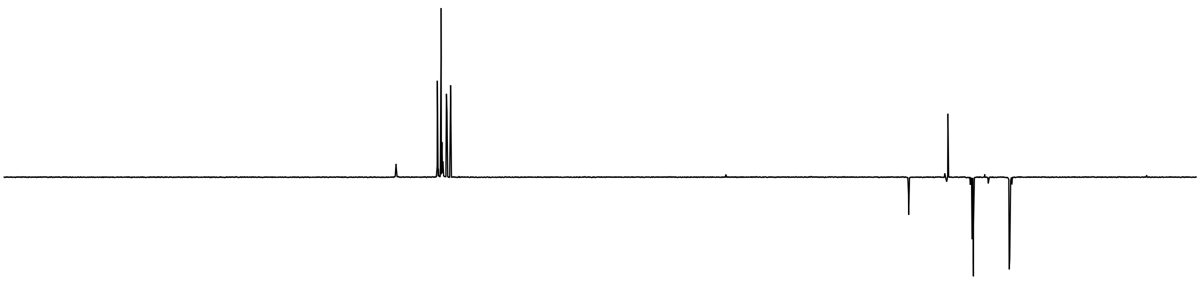

^{13}C NMR (126 MHz, CDCl$_3$)

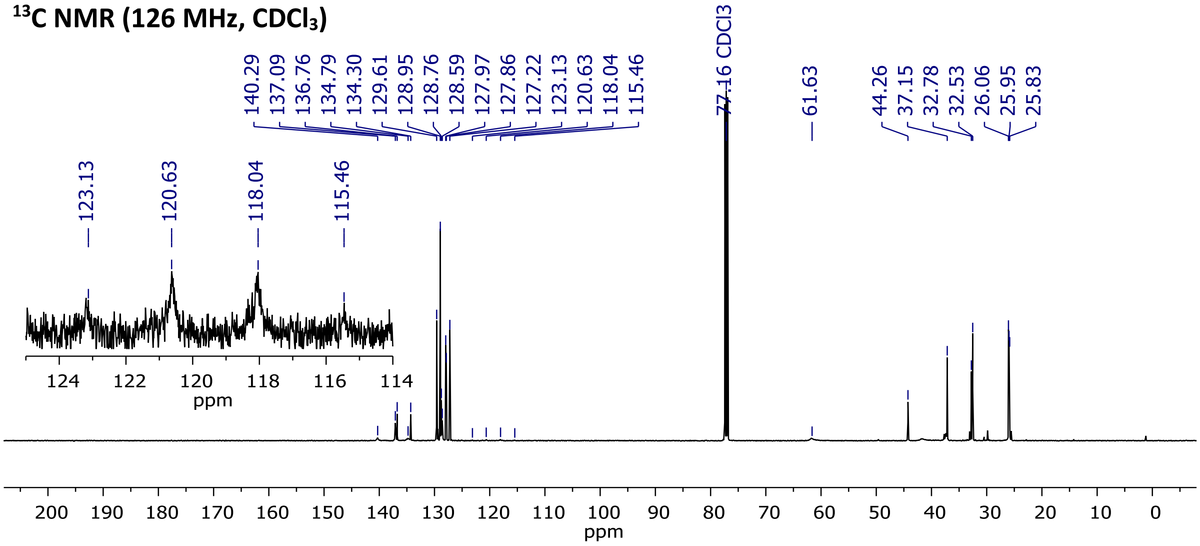

^{1}H NMR (500 MHz, $CDCl_3$)

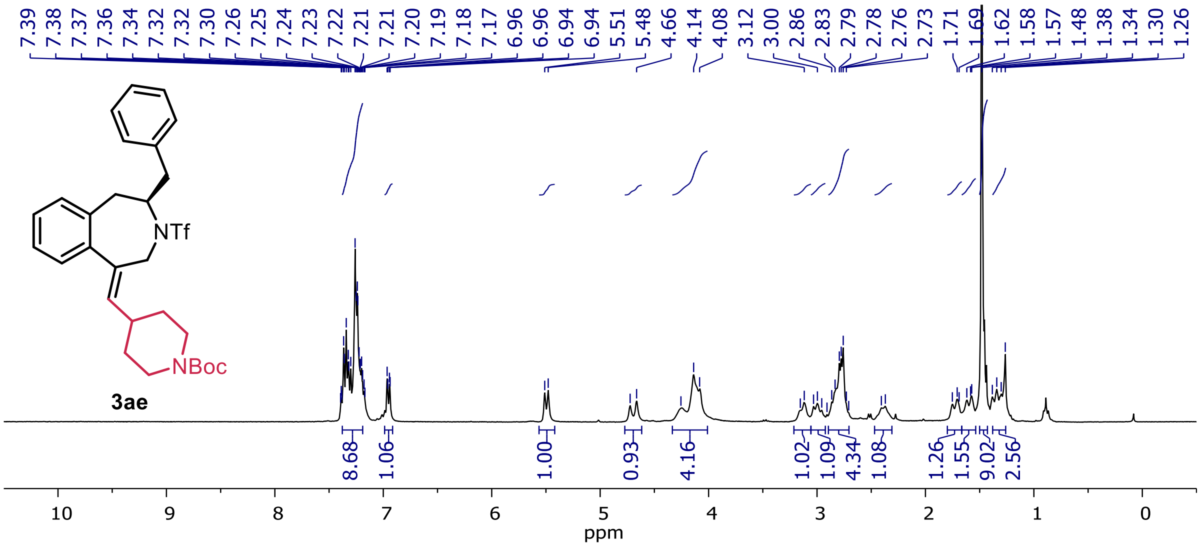

DEPT-135

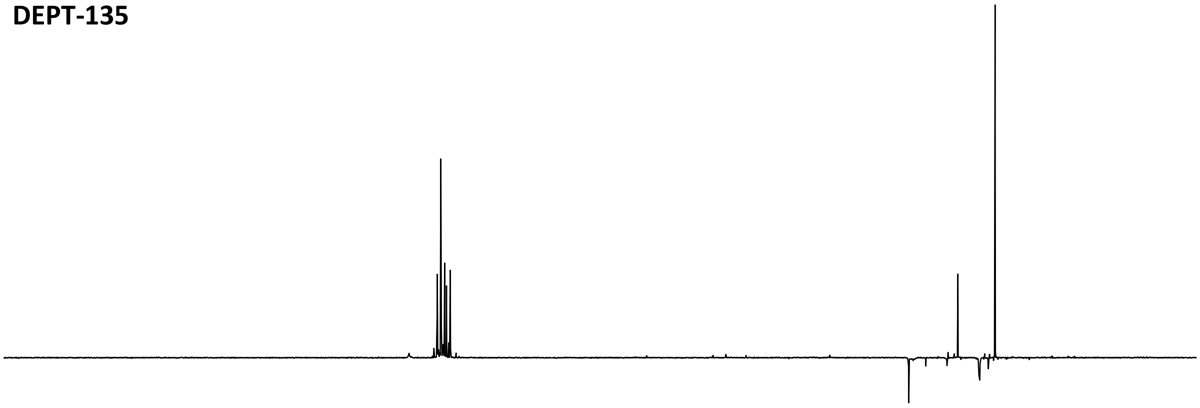

^{13}C NMR (126 MHz, $CDCl_3$)

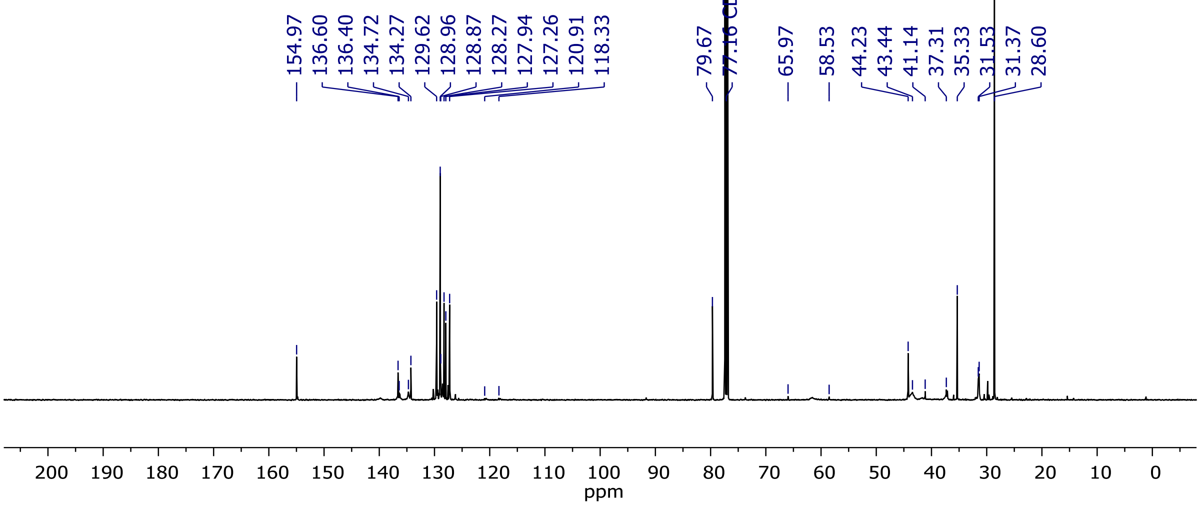

^{1}H NMR (500 MHz, $CDCl_3$)

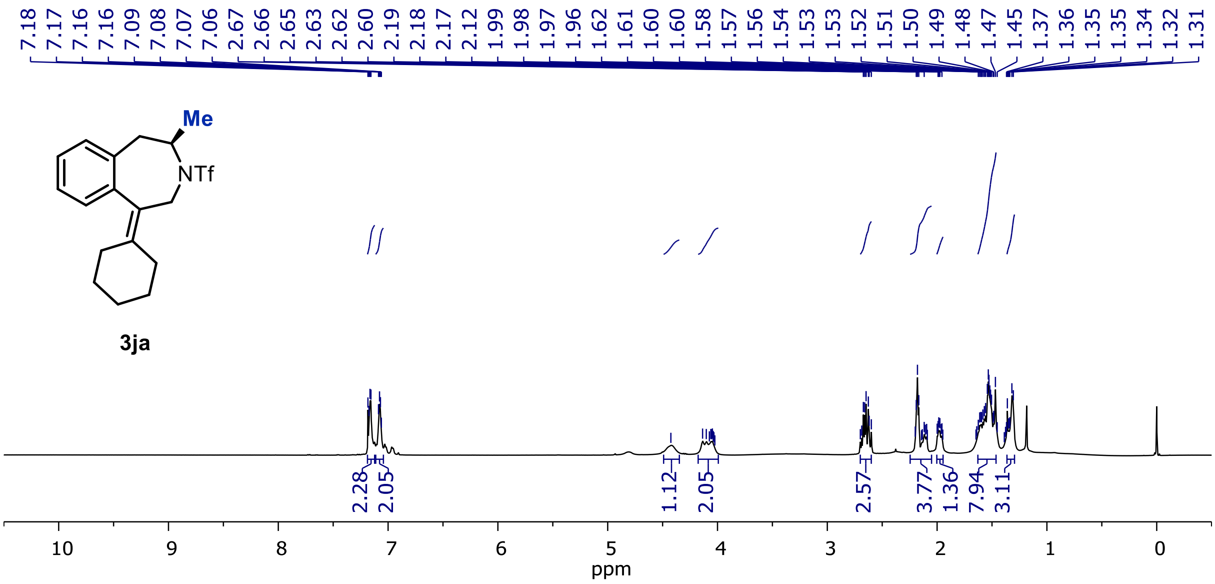

DEPT-135

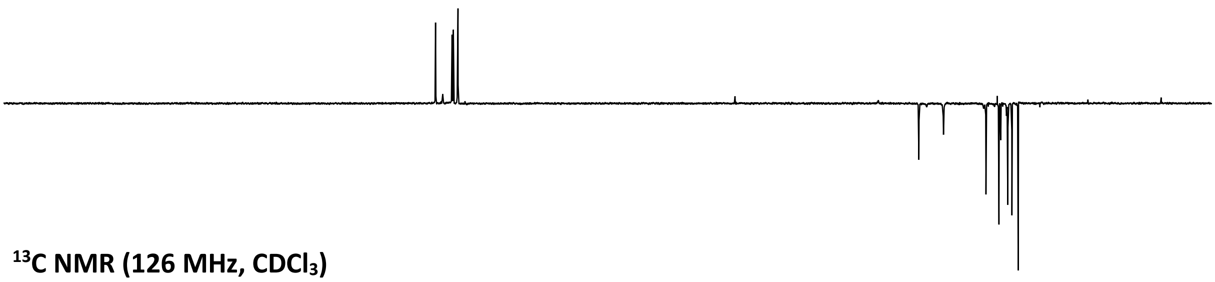

^{13}C NMR (126 MHz, $CDCl_3$)

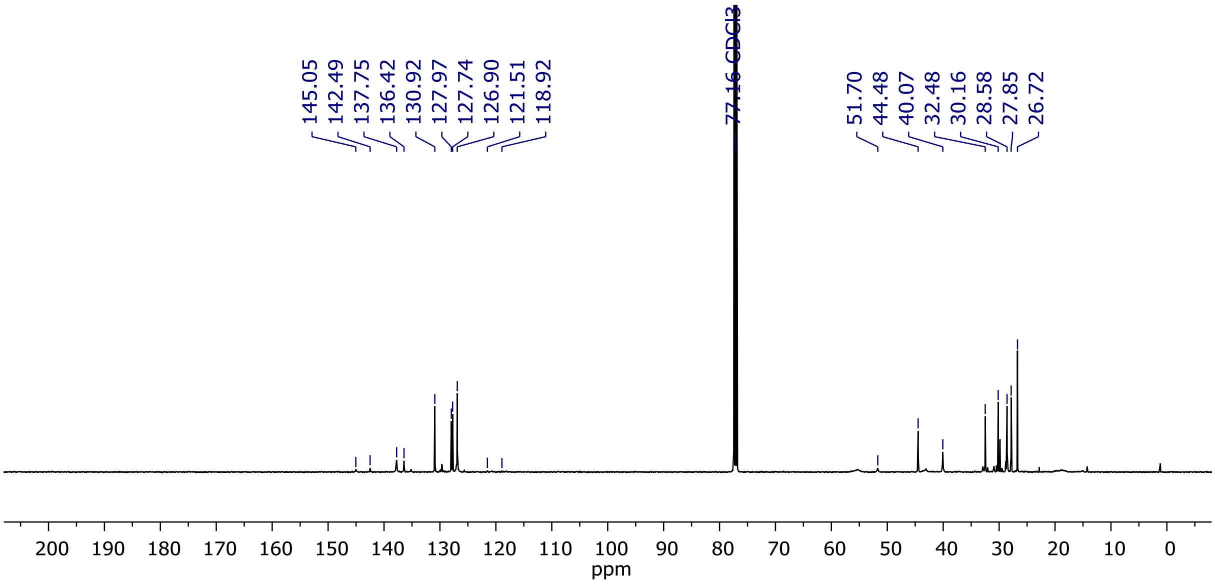

¹H NMR (500 MHz, CDCl$_3$)

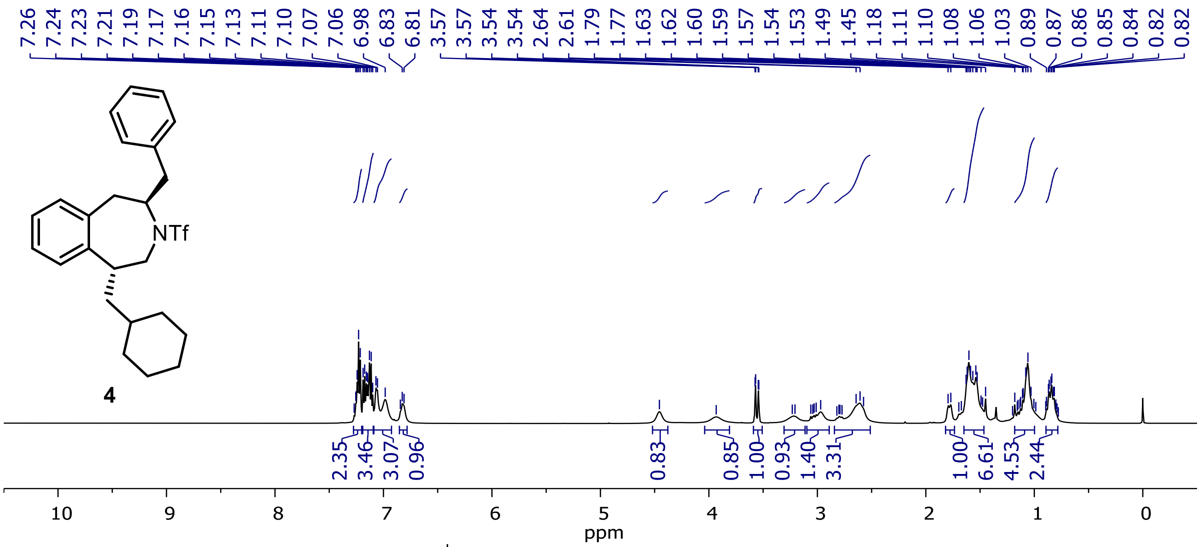

DEPT-135

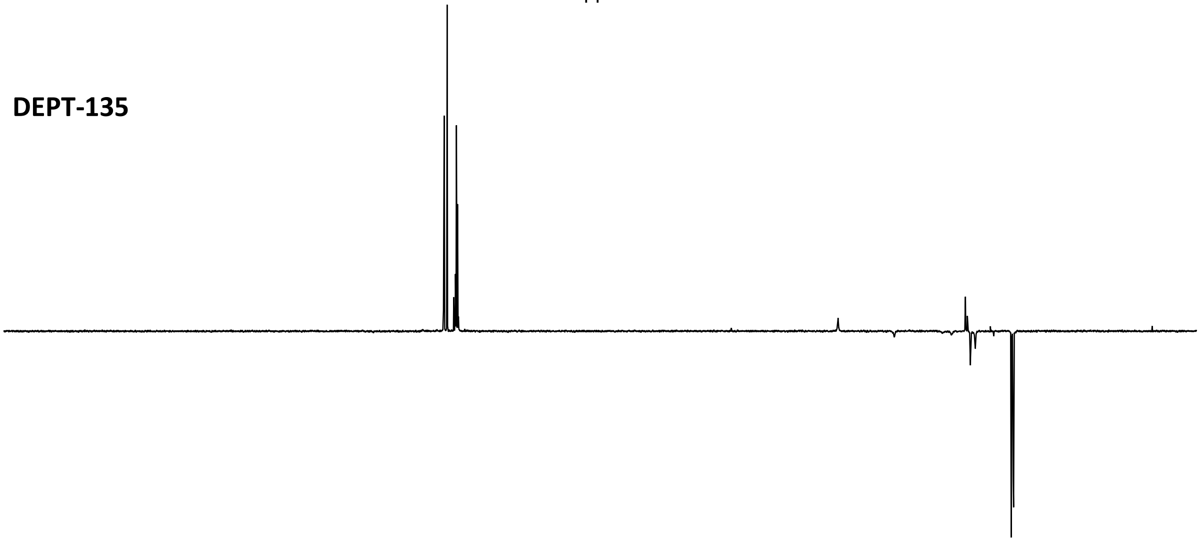

¹³C NMR (126 MHz, CDCl$_3$)

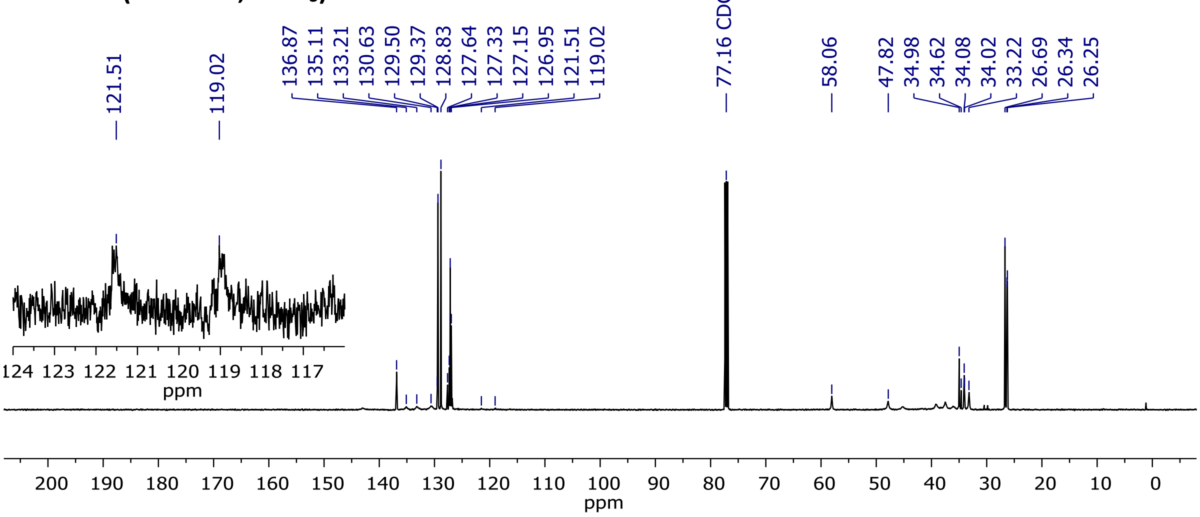

^{1}H NMR (500 MHz, $CDCl_3$)

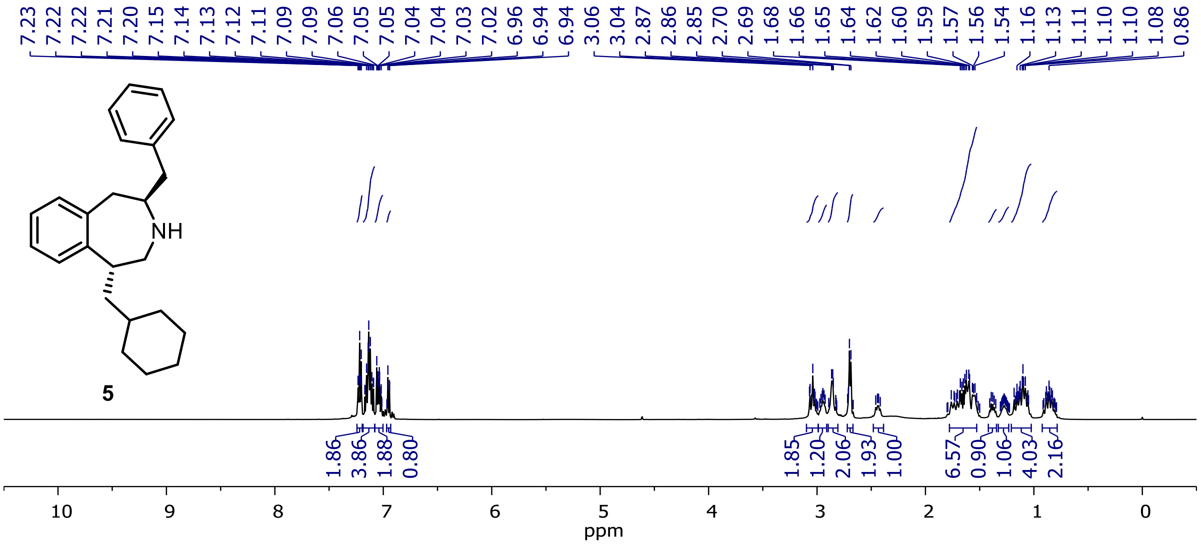

DEPT-135

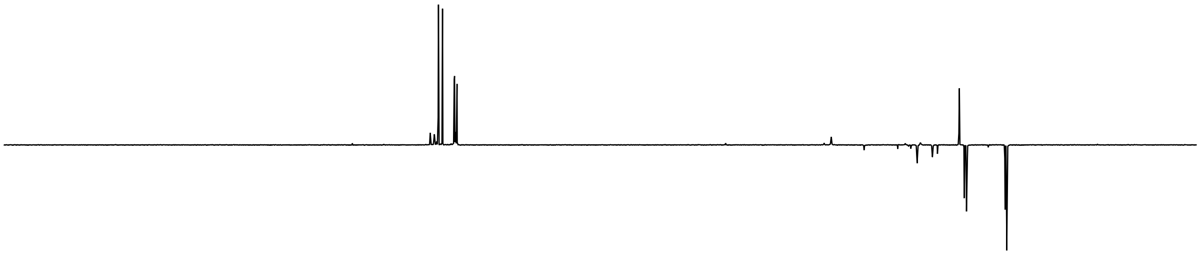

^{13}C NMR (126 MHz, $CDCl_3$)

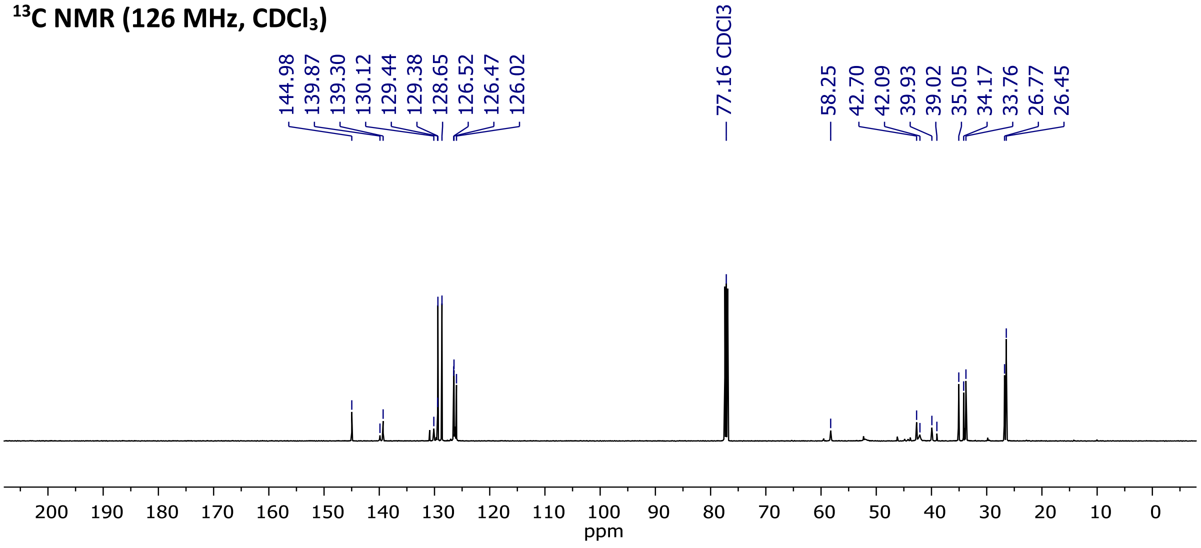

^{1}H NMR (500 MHz, CDCl$_3$)

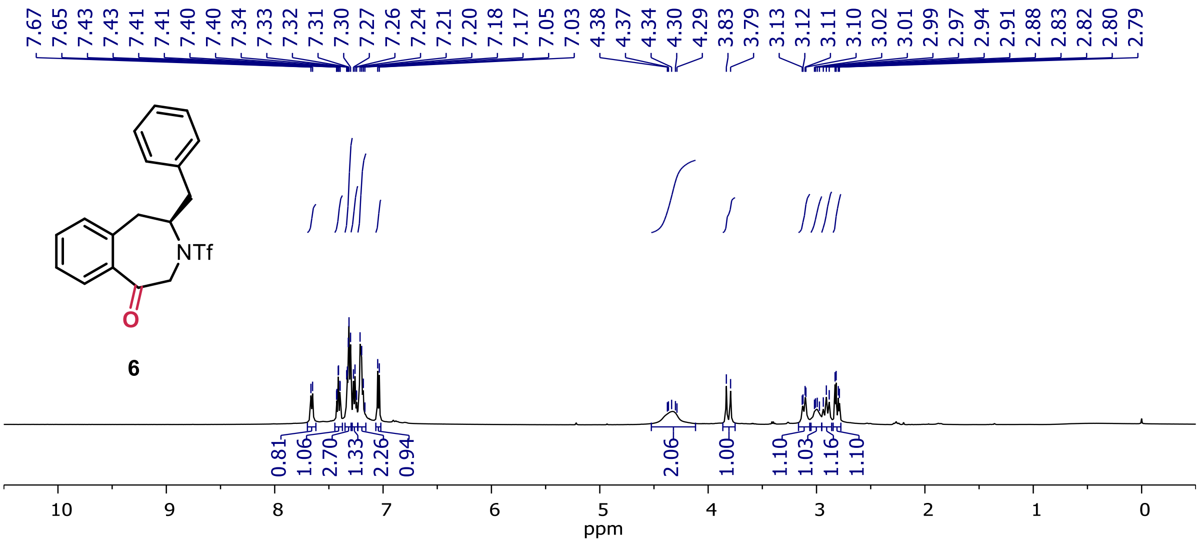

DEPT-135

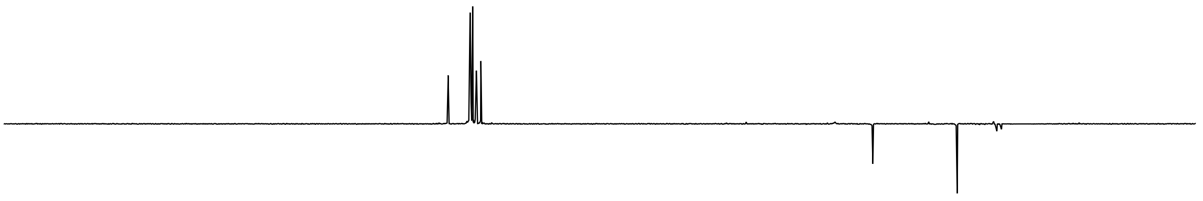

^{13}C NMR (126 MHz, CDCl$_3$)

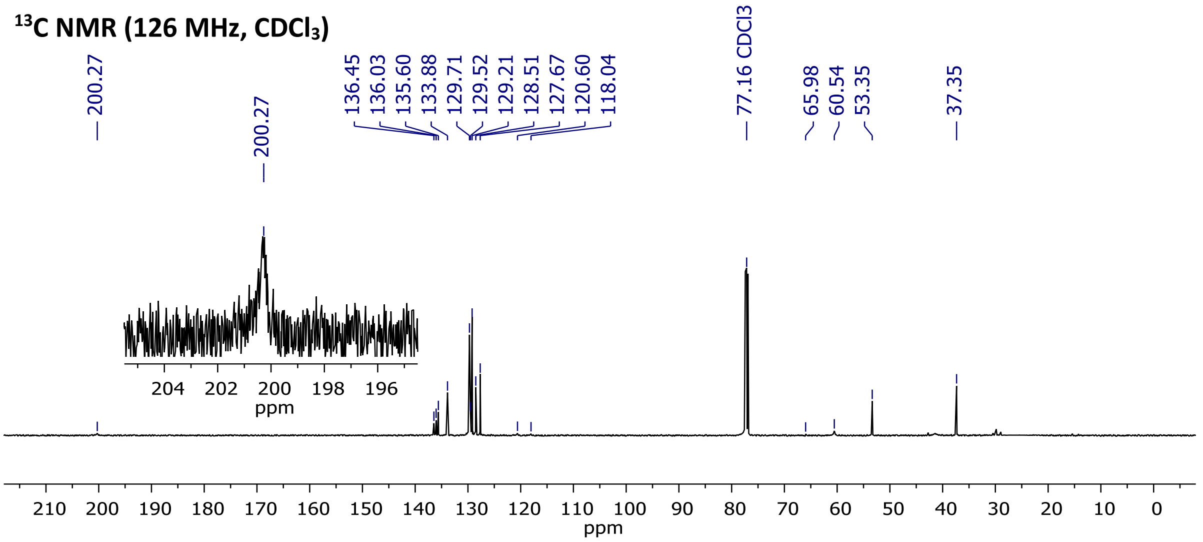

^{1}H NMR (500 MHz, $CDCl_3$)

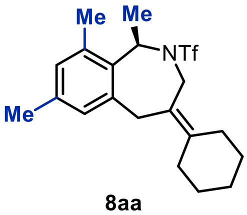

8aa

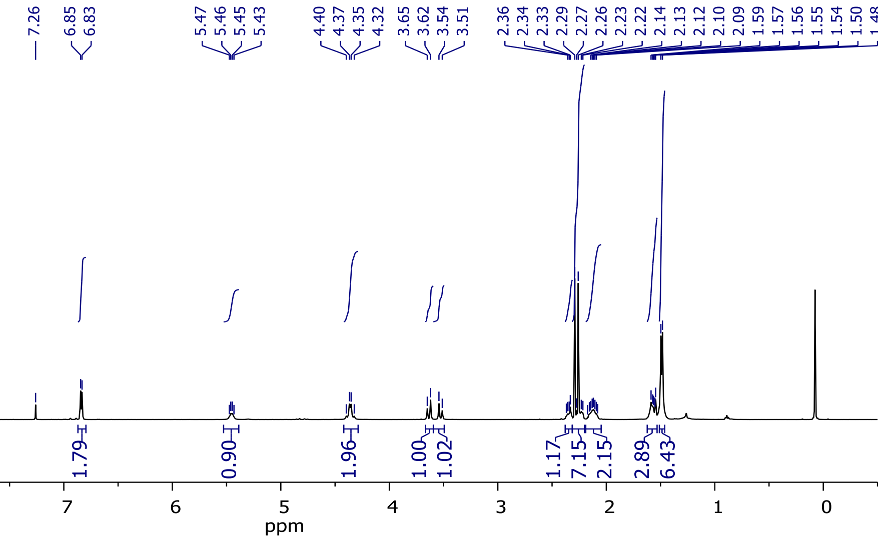

DEPT-135

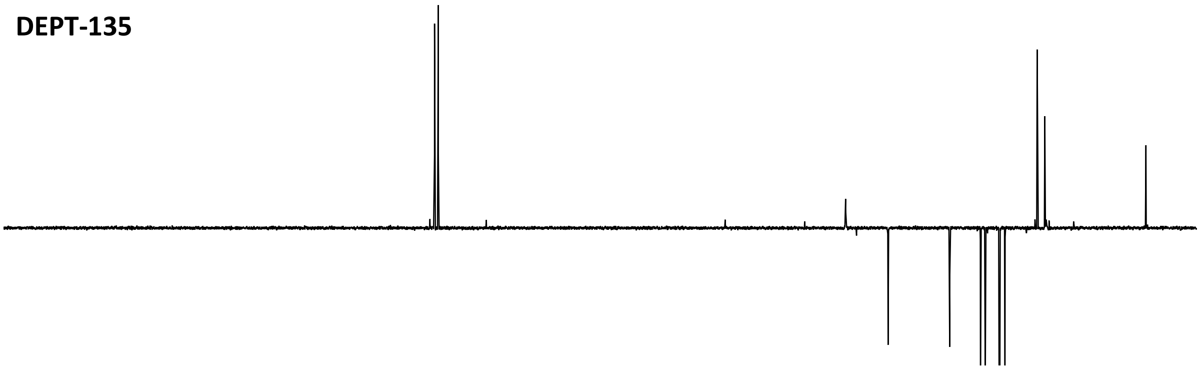

^{13}C NMR (126 MHz, $CDCl_3$)

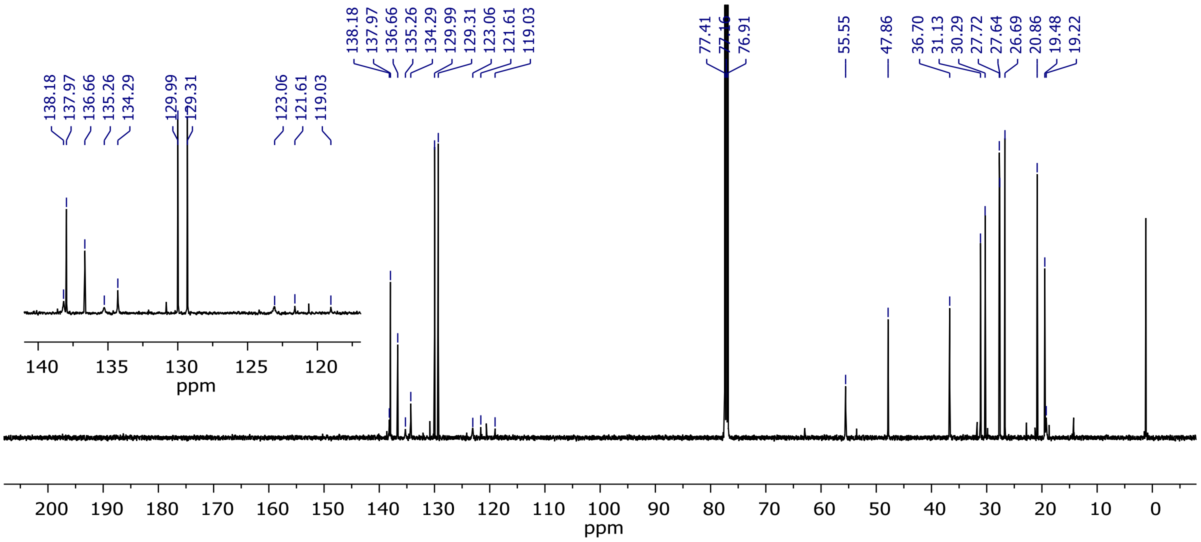

^{1}H NMR (500 MHz, $CDCl_3$)

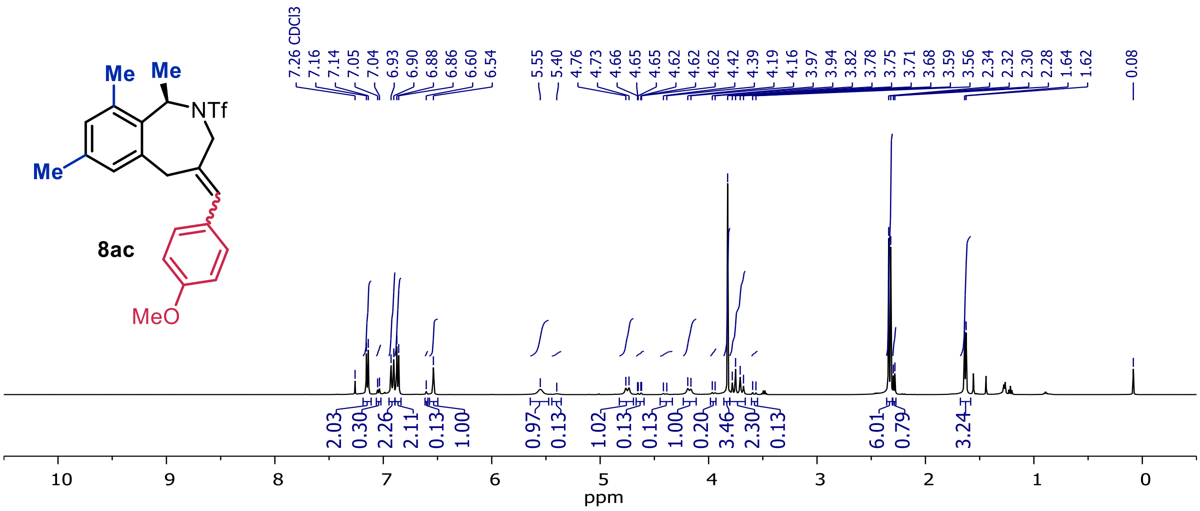

DEPT-135

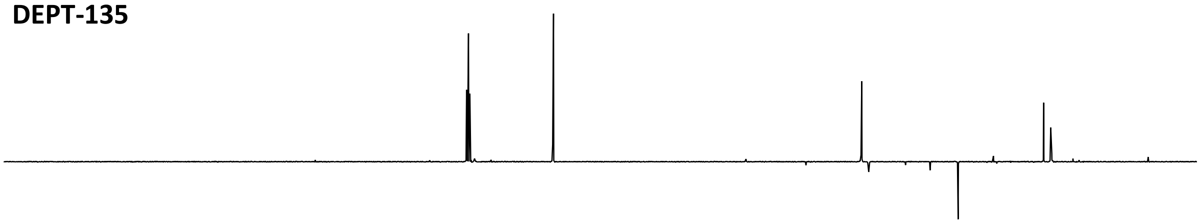

^{13}C NMR (126 MHz, $CDCl_3$)

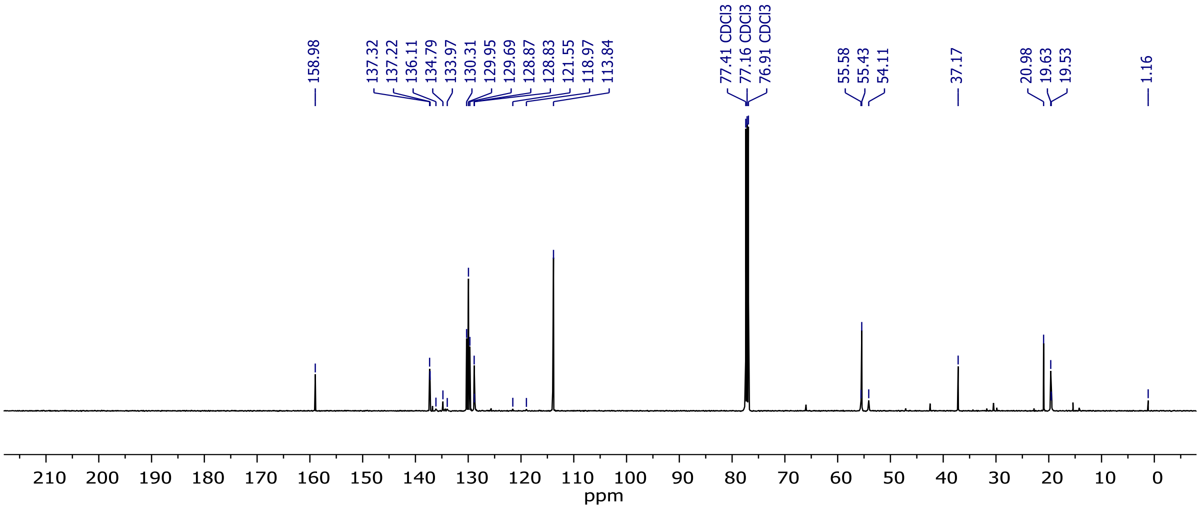

2D-NOESY

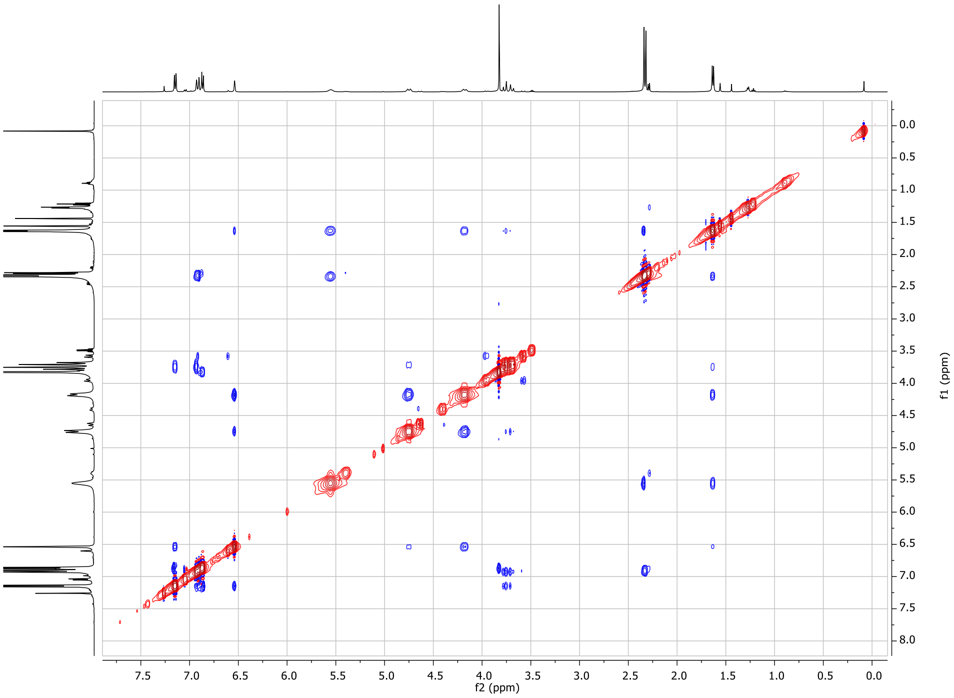

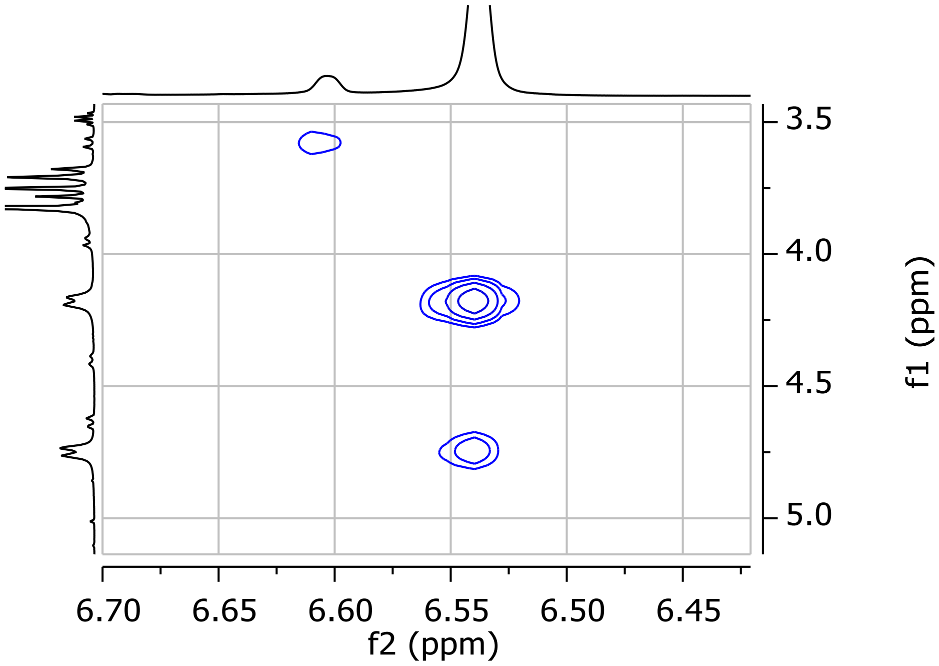

^{1}H NMR (500 MHz, $CDCl_3$)

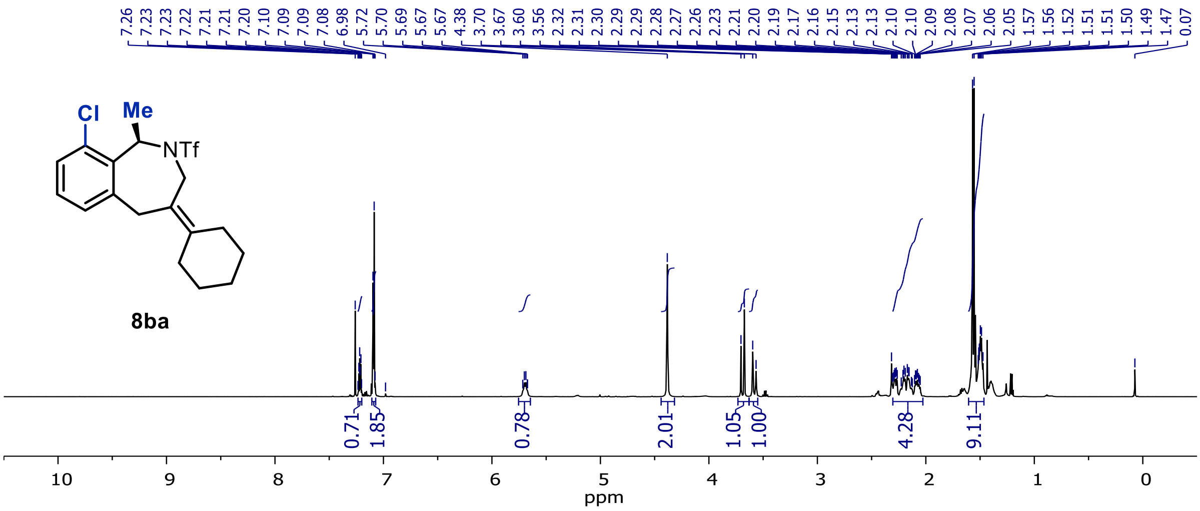

DEPT-135

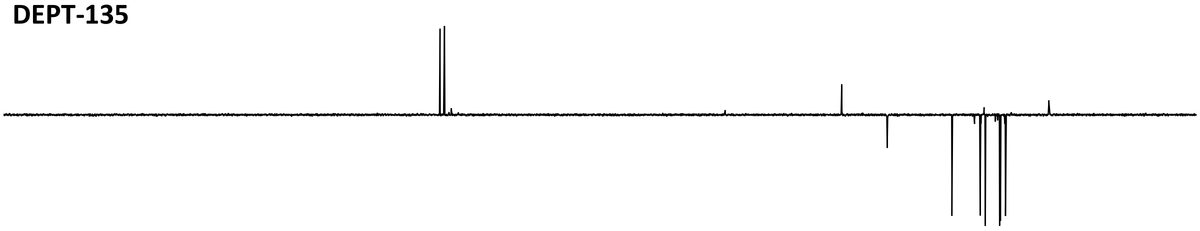

^{13}C NMR (126 MHz, $CDCl_3$)

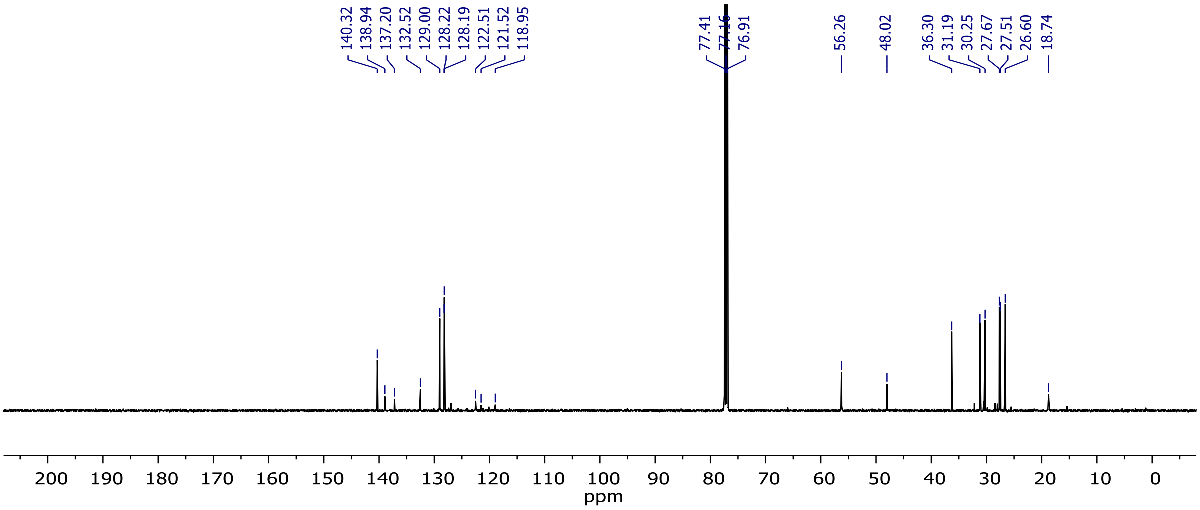

^{1}H NMR (500 MHz, CDCl$_3$)

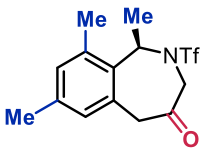

SI1

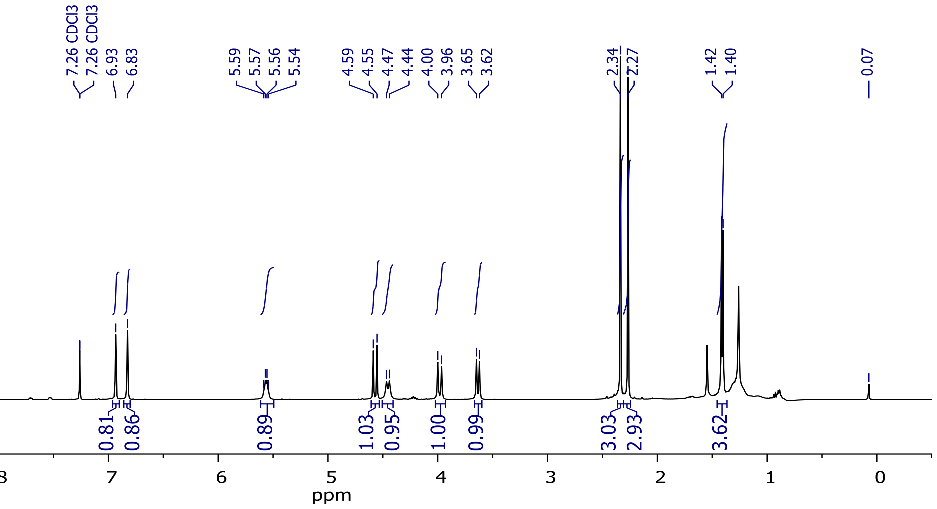

DEPT-135

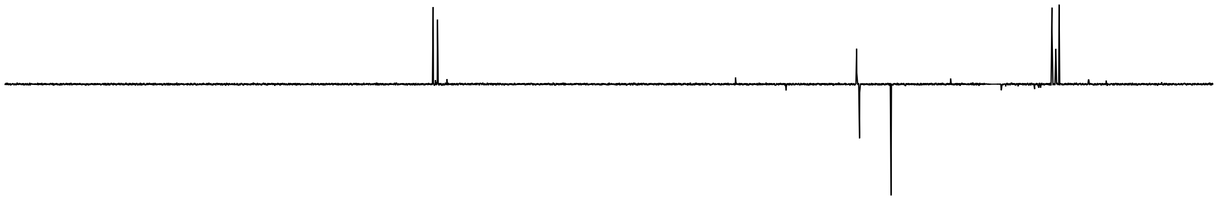

^{13}C NMR (126 MHz, CDCl$_3$)

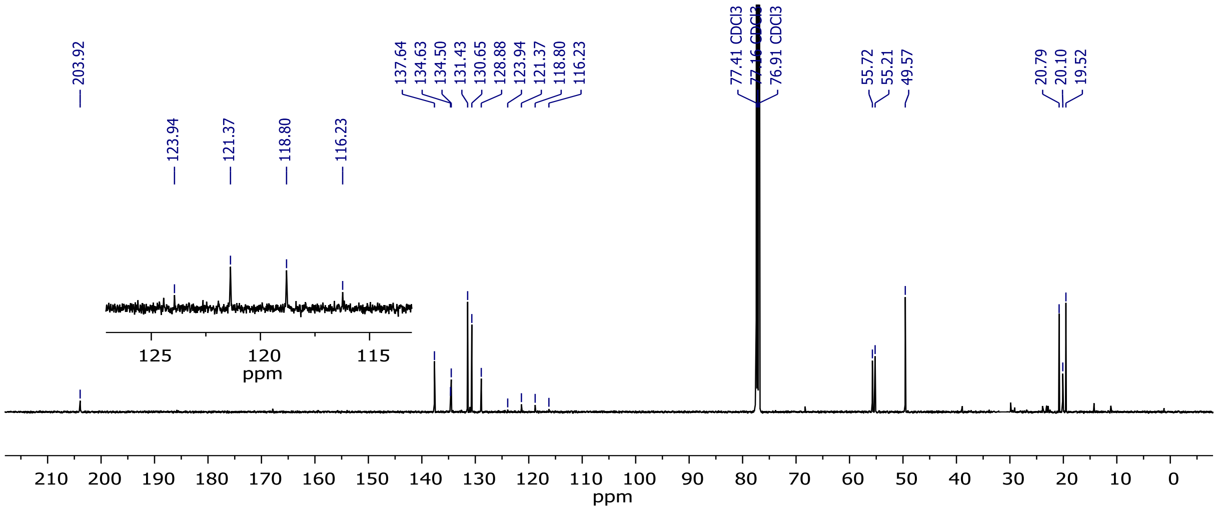

Supplement: Supplementary file 1 — ja2c09479_si_001.pdf [file ja2c09479_si_001.pdf]
